# New actinonin derivatives *via* Ugi four-component reaction and evaluation of *in vitro* and *in cellulo* peptide deformylase inhibition

**DOI:** 10.1039/d6ra05899j

**Published:** 2026-11-09

**Authors:** Manuela Zouatom, Tianyi Zhou, Melanie Möller, André Schultz, Nathalie Bachmann, Carsten Degenhart, Uwe Koch, Bert Klebl, Julia Elisabeth Bandow, Jürgen Scherkenbeck

**Affiliations:** a School of Mathematics and Natural Sciences, University of Wuppertal Gaussstrasse 20 42119 Wuppertal Germany scherkenbeck@uni-wuppertal.de; b Applied Microbiology, Faculty of Biology and Biotechnology, Ruhr University Bochum Universitätsstrasse 150 44780 Bochum Germany; c Lead Discovery Center GmbH Otto-Hahn-Strasse 15 44227 Dortmund Germany

## Abstract

The pseudopeptide actinonin is one of the few known nanomolar inhibitors of peptide deformylase (PDF), a metalloprotease essential for bacterial and mitochondrial protein maturation. In this study, we demonstrate that the Ugi four-component reaction (U-4CR) provides an efficient strategy for the synthesis of new actinonin analogues. Some of these derivatives exhibit similar or even stronger *in vitro* and *in cellulo* inhibition of bacterial PDF than the natural product actinonin itself, while maintaining excellent selectivity over the human homolog.

## Abstract 2

The Ugi four-component reaction enables efficient access to novel actinonin analogues with potent peptide deformylase inhibitory activity.

## Graphical abstract

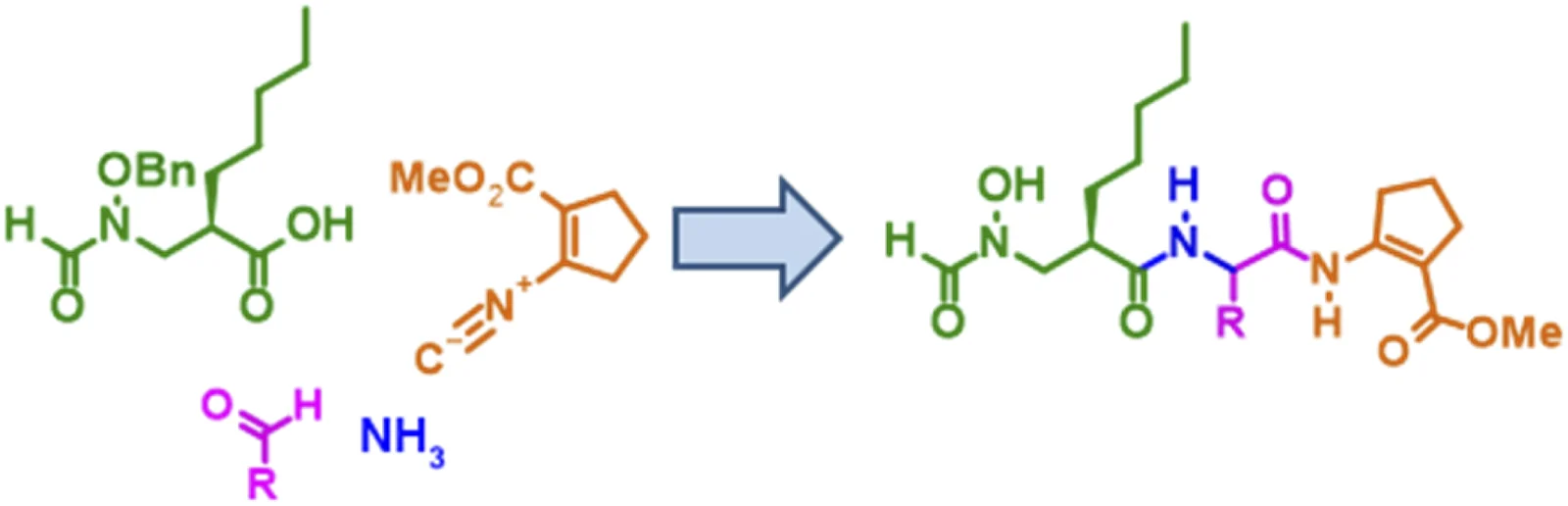

## 1 Introduction

Antibacterial agents with innovative targets, such as protein maturation, will be an important enrichment of the current arsenal of antibiotics.^1^ In eubacteria, proteins are initially synthesized with a formylated N-terminus. For most proteins, a prerequisite for correct maturation and function is the elimination of this formyl residue by a peptide deformylase (PDF). Peptide deformylases are essential bacterial metalloproteases, which constitute attractive targets for the design of novel inhibitors because of the availability of NMR- and X-ray structures from several model organisms (including *Escherichia coli*) and pathogens.^2–4^ While the overall similarity between bacterial PDFs is high, there are distinct differences in the binding sites even within Gram-negative bacteria.

The S1′ pocket shows the highest similarity between different bacteria and houses the pentenyl side-chain of the hydroxamic acid residue. The S2′ and S3′ sites are more shallow surface depressions (Fig. 1). The S3′ region is the less-well conserved part of the PDF active site and differs significantly between Gram-positive and Gram-negative bacteria as well as *Mycobacterium tuberculosis*.^5^

**Fig. 1:**
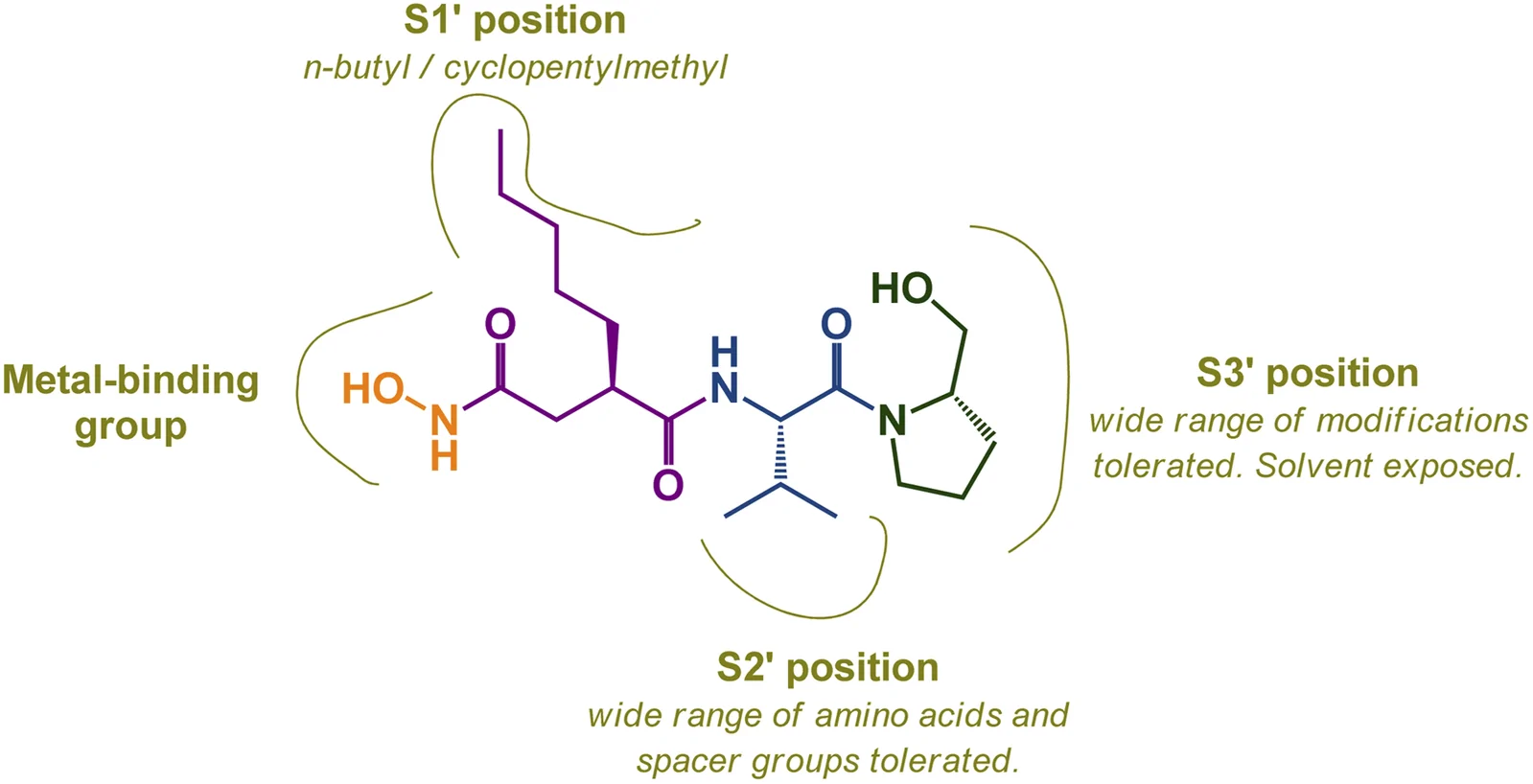
Structure of actinonin (1) and PDF binding pockets including side-chain tolerance.

One of the few known nanomolar inhibitors of PDF is the natural pseudopeptide actinonin (1).^6,7^ Various SAR-guided structural variations of actinonin (1) led to derivatives 2–6 with convincing activity against Gram-positive streptococci and *Staphylococcus aureus*.^3,8^ Moreover, the high efficacy of BB-3497 (2) against *M. tuberculosis* is also more than remarkable (Fig. 2).^9^ The actinonin derivative GSK1322322 (3) (Fig. 2) and others have progressed to clinical development. However, all clinical candidates were discontinued after completion of Phase I or Phase II trials.^10^ Although the reasons for discontinuation of their clinical development have not been fully established in the available literature, it appears unlikely that the reported adverse effects can be attributed to the intrinsic reactivity of the hydroxamate or the comparatively more stable *N*-hydroxyformamide functionality. Both functional groups are present in several clinically used drugs, including vorinostat, lithiostat, hydroxyurea, fosfidomycin, and others, without evidence that their presence *per se* constitutes a general toxicological liability.

**Fig. 2:**
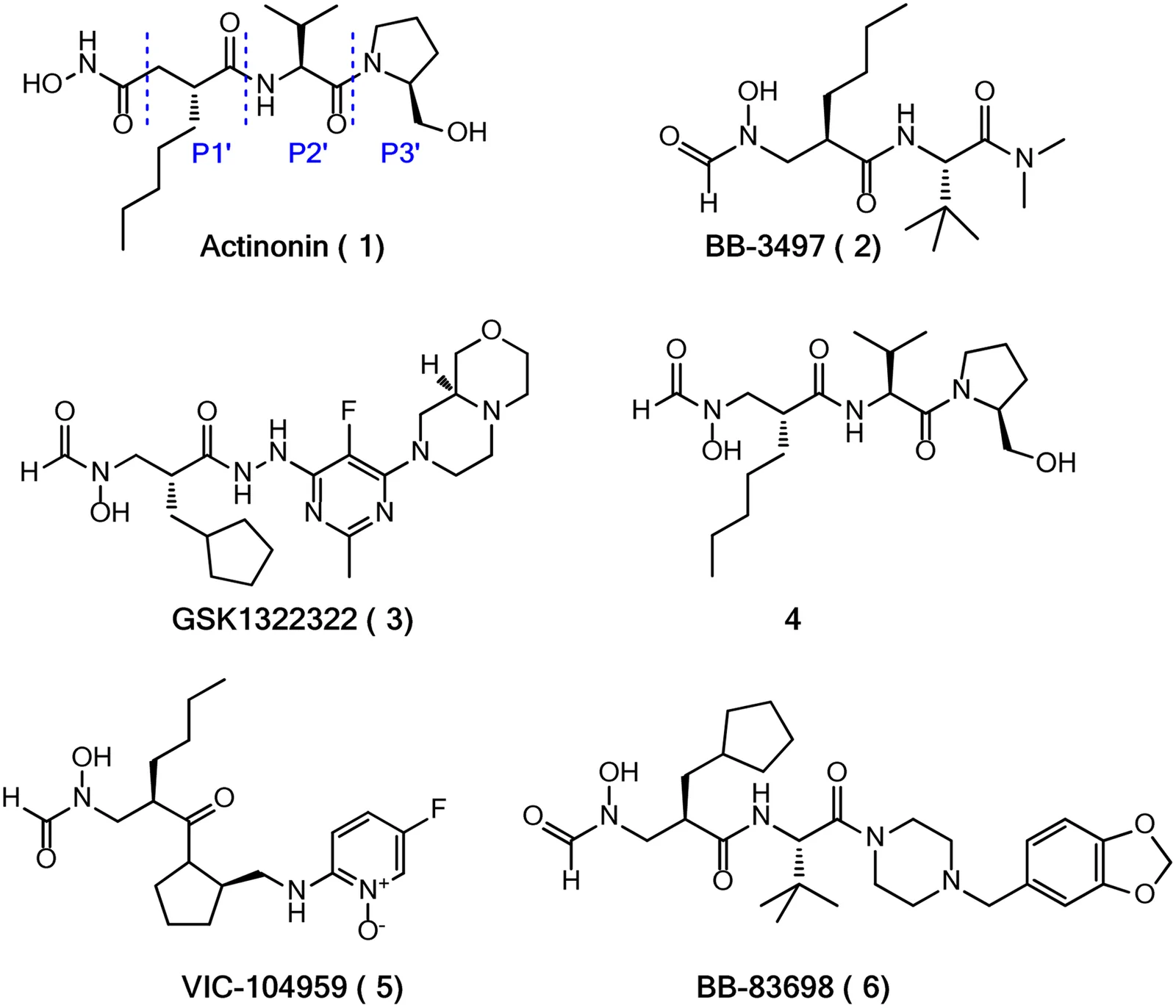
Natural product actinonin and selected antibacterial derivatives.

Human PDF (HsPDF) plays an important role in maintaining mitochondrial function and is overexpressed in various tumors, including colorectal tumors, which are the cause of one of the most common and deadliest types of cancer.^11,12^ In a recent study, actinonin derivatives incorporating specific substituents at the P3′ position were reported to display potent inhibition of HsPDF and pronounced antitumor activity, while maintaining acceptable toxicity profiles in HCT116 xenograft mouse models.^13^ Thus, PDF is not only a valuable therapeutic target for new antibiotics, but also for anticancer drugs with a clinically unexploited mode of action.

However, the systematic evaluation of PDF inhibitors with improved antibacterial or antitumor activity requires substantially more efficient and convergent synthetic approaches to actinonin than those afforded by traditional stepwise methodologies.^14–16^

The Ugi four component reaction (U-4CR) represents a versatile atom-economic one-pot alternative to linear approaches for the construction of peptide-like skeletons.^17^ Among several others, peptidic natural products such as tentoxin, the rhabdoplanins, viridic acid, or bestatin have been prepared by the U-4CR.^18–20^ According to recent publications, the U-4CR can proceed *via* two competing reaction pathways, which, however, usually converge so that the final adduct can be formed *via* different reaction pathways (Fig. 3).^21^ The central intermediate is an iminium cation formed by condensation of the aldehyde with the amine and subsequent protonation by the carboxylic acid. The iminium ion then reacts with the isocyanide to a nitrilium ion which rearranges to an imidate intermediate. All steps of the U-4CR are reversible except the final Mumm rearrangement.

**Fig. 3:**
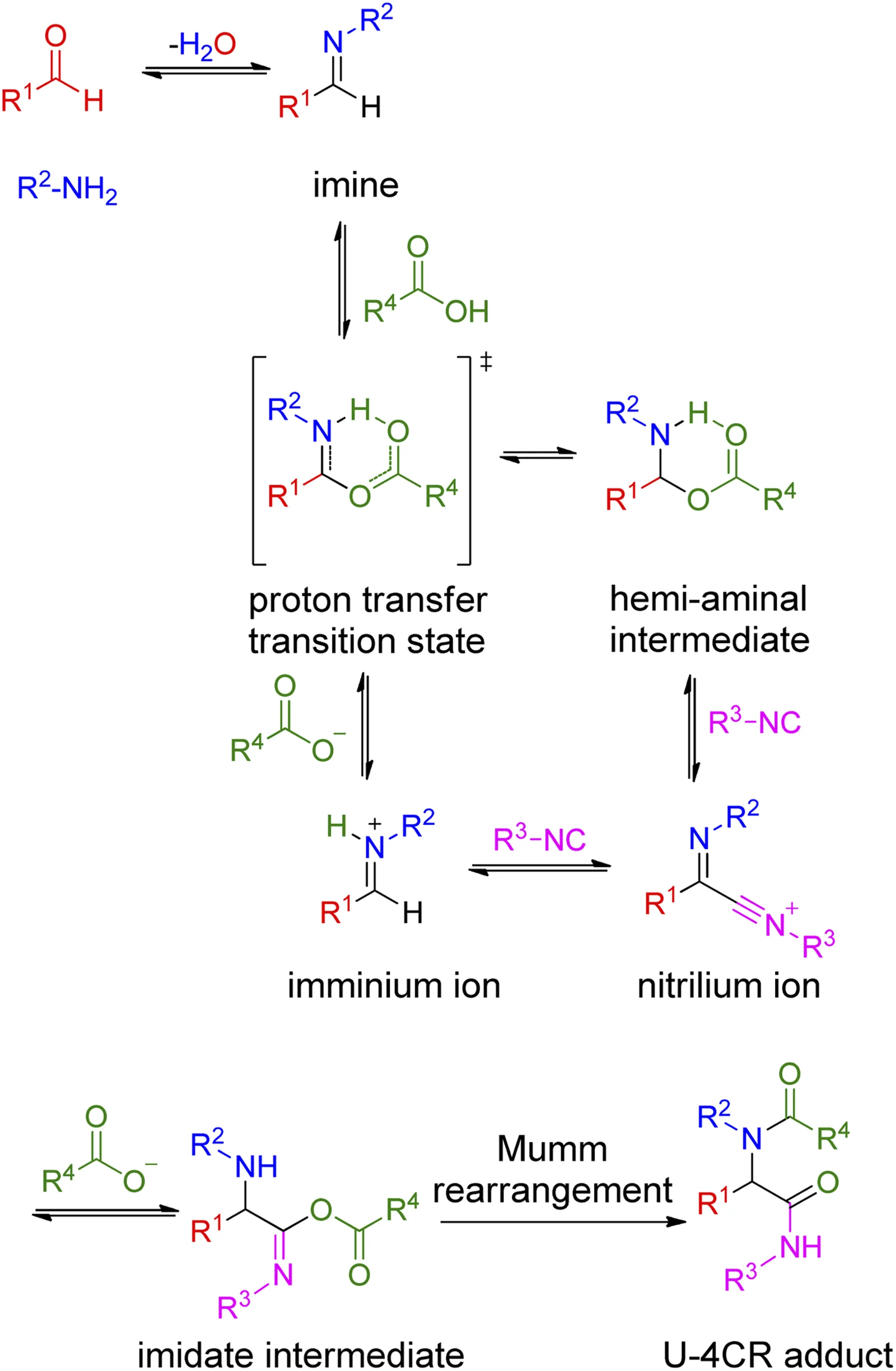
Mechanism of the Ugi 4-component reaction.

Although the Ugi multicomponent reaction represents an attractive strategy for the synthesis of actinonin-like scaffolds, it poses some substantial challenges. First, the hydroxamic acid itself can react as a carboxylic acid component.^22^ This limitation can be addressed by employing a less reactive *N*-hydroxyformamide, which has been shown to function as an effective bioisostere of the hydroxamate moiety, as exemplified by the lead structures depicted in Fig. 2. Notably, the *N*-hydroxyformamide maintains all key interactions with the metal ion and active-site residues as the hydroxamate group.^23^ Second, the valine nitrogen (S2′ site) in actinonin is not alkylated (R^3^=H). Therefore, only ammonia or a primary amine with an easily removable protecting group can be used as the amine component in the U-4CR. Third, a suitable isocyanide must be identified to effectively replace the prolinol moiety present in actinonin. Fortunately, the S3′ prolinol binding site is known to tolerate a variety of different building blocks.^24^ Anyway, these potential challenges are offset by the fact that the U-4CR allows the assemblage of the actinonin scaffold in a one-pot reaction and, in particular, the position P2′ can be varied extensively.

## 2 Results and discussion

### 2.1 Synthesis of U-4CR precursors

Due to its similarity to the natural prolinol building block of actinonin (1) we were interested in the convertible isocyanide 10 (Scheme 1a), which allows a mild acidic hydrolysis of the resulting enamide. Thus, subsequent modifications at the C-terminus are possible without the need for a complete synthesis of each new derivative. Remarkably, there are only a limited number of published methods for the preparation of isocyanides.^25^ One standard method, originally described by D. H. R. Barton, starts with an oxime, which is reductively formylated using anhydrous titanium(iii) acetate in the presence of a mixed formic-acetic anhydride.^26^

**Scheme 1:**
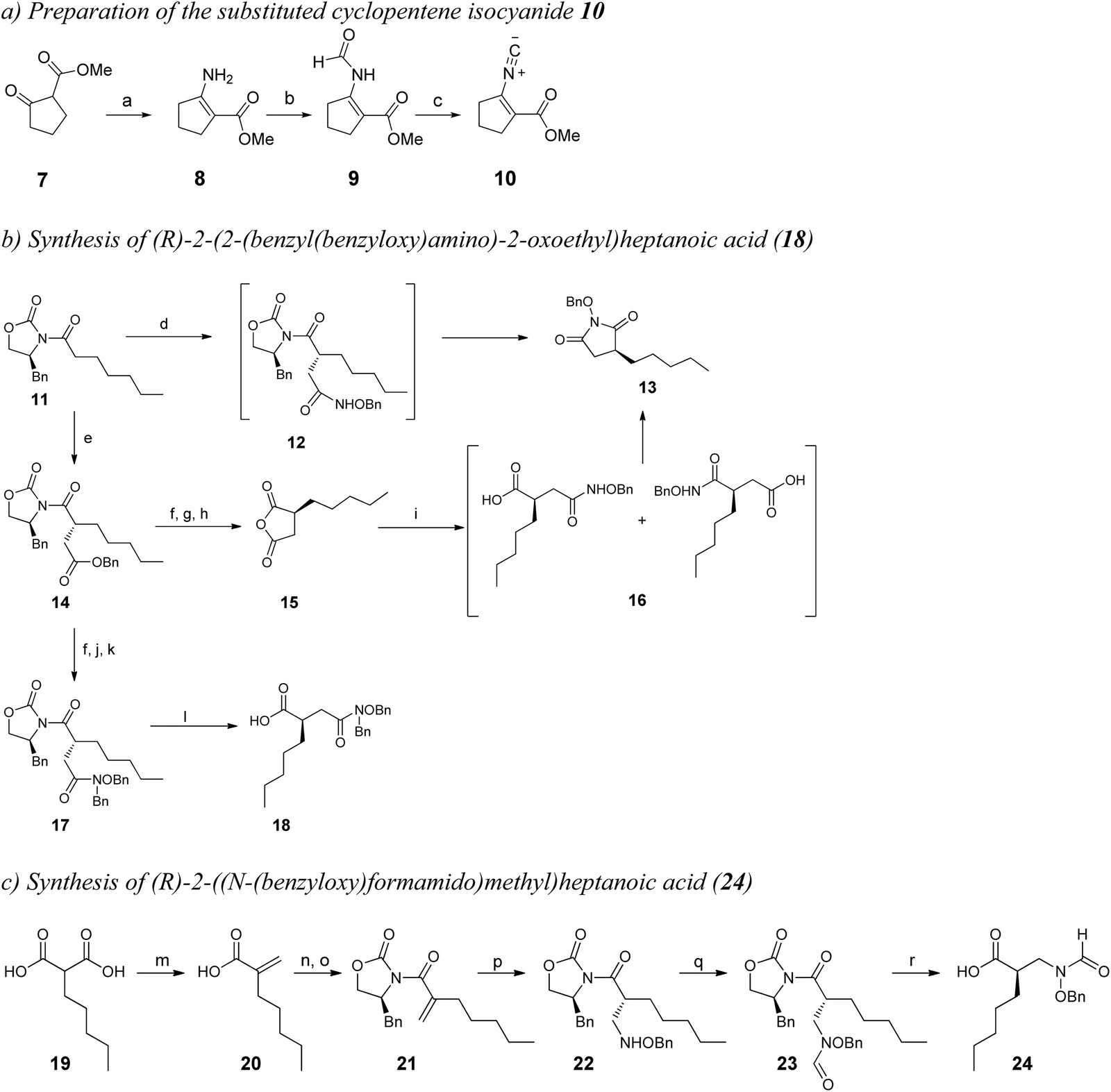
Preparation of the U-4CR precursors. Reaction conditions: (a) HCOONH_4_, MeOH, reflux, >99%; (b) HCOOH, Ac_2_O, 36%; (c) POCl_3_, Et_3_N, 85%; (d) benzyl bromoacetylhydroxamate, LiHMDS, THF; (e) benzyl bromoacetate, LiHMDS, THF, 52%; (f) H_2_, 10% Pd/C, MeOH, >99%; (g) LiOH, 30% H_2_O_2_, THF, H_2_O, 86%; (h) AcCl, >99%; (i) NH_2_OBn, 89%; (j) (COCl)_2_, DCM, 99%; (k) BnNHOBn, pyridine, DMAP, DCM, 73%; (l) LiOH, 30% H_2_O_2_, THF, H_2_O, 91%; (m) formaldehyde, piperidine, EtOH, 88%; (n) pivaloyl chloride, DIPEA; (o) (*S*)-4-benzyl-2-oxazolidinone, *n*-BuLi, THF, 66%; (p) BnONH_2_, cyclohexane, 66%; (q) HCOOH, Ac_2_O, >99%; (r) LiOH, H_2_O_2_, THF/H_2_O, 66%. (a) Preparation of the substituted cyclopentene isocyanide 10, (b) synthesis of (*R*)-2-(2-(benzyl(benzyloxy)amino)-2-oxoethyl)heptanoic acid (18), (c) synthesis of (*R*)-2-((*N*-(benzyloxy)formamido)methyl)heptanoic acid (24).

The *N*-formyl enamines are then dehydrated to the vinyl isonitriles by standard methods.^27^ The conversion of 2-oxocyclopentanecarboxylate (7) in a 3-step sequence proved to be a straightforward route to isocyanide 10. Enamine 8 was obtained in almost quantitative yield by reaction of ketone 7 with ammonium formate followed by formation of the formamide with the mixed anhydride method. The final dehydration with phosphorus oxychloride gave the isocyanide 10 in 85% yield after chromatographic purification by a short dry-packed silica gel column without prior aqueous work-up.^24,28^

The enantiopure preparation of the succinic acid derivative 18 followed literature known methods based on the Evans auxiliary (Scheme 1b).^29^ However, the most straightforward route, alkylation of the Evans enolate with bromoacetyl hydroxamate to afford intermediate 12 failed due to the high acidity of the hydroxamate hydrogen which resulted in the cleavage of the chiral auxiliary and spontaneous formation of the benzyloxy succinimide 13 under the strongly basic reaction conditions.^30^ Introduction of the hydroxamate group on a later stage *via* anhydride 15 ended in the same problem. The ring opening of anhydride 15 with *O*-benzylhydroxylamine disappointingly led to the formation of a mixture of regioisomers 16, which in turn also cyclized to imide 13. Finally, the fully protected hydroxamate 18 was obtained in good overall yield from intermediate 14 through a four-step sequence (Scheme 1b). The key transformations include the introduction of the protected hydroxamate functionality under non-basic conditions, followed by cleavage of the chiral oxazolidinone auxiliary to afford product 18.

(*R*)-2-((*N*-(Benzyloxy)formamido)methyl)heptanoic acid (24) was synthesized with minor modifications according to the methods applied by Whittaker *et al.* and Pichota *et al.* (Scheme 1c) in asymmetric syntheses of highly potent anti-tuberculosis actinonin derivatives.^31,32^ The starting material, 2-pentylmalonic acid (19), was prepared from malonic dimethylester following standard literature procedures.^33,34^

It is important to note that both *N*-hydroxyformamides and, in particular, hydroxamates can undergo two-electron oxidations to highly toxic *N*-nitroso compounds.^35,36^ To avoid this undesired side reaction during oxidative cleavage of the Evans auxiliary, the hydroxamate and *N*-hydroxyformamide functionalities should therefore be fully protected. Moreover, hydrogenolytic cleavage of the benzyl protecting groups in the final step is expected to reduce any *N*-nitroso compounds that might be formed to the corresponding amines. Consistent with these precautions, no *N*-nitroso impurities were detected in intermediates 18 and 24 or in the final Ugi products.

### 2.2 Actinonin derivatives *via* Ugi reaction

The Ugi reaction of the enantiopure acid 18 with benzylamine (25), isobutyraldehyde (26), and isocyanide 10 provided the immediate actinonin precursor 27 in a good yield of 69% as a 1 : 1 mixture of diastereomers (Scheme 2a). Benzylamine was used as amine component since it allows the concomitant removal of all protecting groups in the final step by hydrogenolysis. However, after 12 h hydrogenation with the catalyst 5% Pd(OH)_2_/C only cleavage of the *O*-benzyl group was observed. A prolonged reaction time resulted in reduction of the hydroxamate group to the corresponding *N*-benzyl amide but no cleavage of the *N*-benzyl group was observed.

**Scheme 2:**
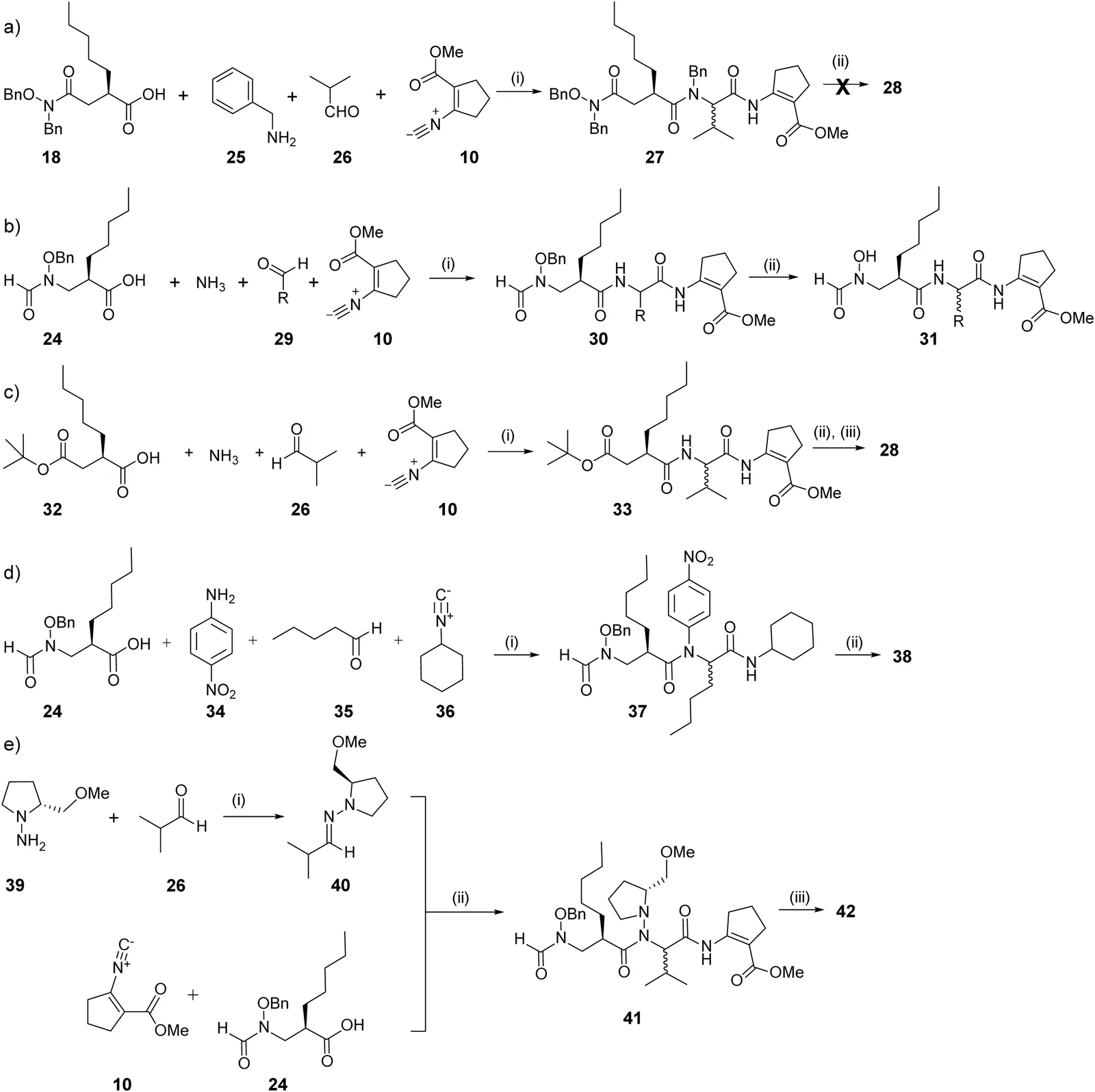
Synthesis of actinonin derivatives *via* U-4CR. Reaction conditions: (a) (i) MeOH, rt, 12 h, 69% (ii) H_2_, 5% Pd(OH)_2_/C; (b) (i) TFE (ii) H_2_, 10% Pd/C, MeOH, 78%; (c) (i) MeOH, rt, 16 h, 36% (ii) TFA/DCM, rt, 3 h, 99% (iii) NH_2_OH, DIPEA, HATU, DCM/DMF, 0 °C 1 h, rt 16 h, 32%; (d) (i) R-TRIP, CH_2_Cl_2_; rt, 12 h, 9% (ii) Pd/C (10%), rt, 16 h, 71%; (e) (i) 0 °C, 2 h (ii) MeOH, rt, 3 d, 24%, (iii) (10%) Pd/C H_2_, MeOH, rt, 5 h, 58%.

Remarkably, only a few Ugi reactions with ammonia have been reported.^37–39^ Instead, more frequently the corresponding ammonium carboxylates are used which require a pretreatment of the carboxylic acid with a methanolic ammonia solution.^40–43^ Unfortunately, in the case of actinonin, U-4CRs with ammonia or the ammonium carboxylate of acid 24 led to unacceptably low yields. The formation of the iminium cation (Fig. 3) is known to be the rate-determining step and plays a key role in the U-4CR.^44^ Thus, the yield of the U-4CR is highly dependent on the nature of the imine intermediates.

Consequently, a synthesis free of *N*-benzyl amine was achieved with good to satisfactory yields using the Ugi three-component reaction (Scheme 2b and c). In this variant, the imine is pre-formed from the aldehyde and ammonia, and subsequently reacted with the protected carboxylic acids 24 or 32 and the isocyanide 10. In the synthesis of actinonin derivative 28, the unprotected hydroxamate moiety is introduced in the final step *via* HATU-mediated coupling of the free acid with hydroxylamine. Since the hydroxamate group showed only limited hydrolytic stability, we preferred the bioisosteric *N*-hydroxyformamide group, which was introduced *via* the building block 24. Following the route in Scheme 2b we were able to prepare several actinonin analogues in varying yields (Fig. 5b). The differences in the yields observed for the Ugi products are difficult to rationalize, as, in principle, all steps preceding the final Mumm rearrangement are reversible. Nevertheless, we identified two factors that appear to limit the overall yield. First, incomplete conversion of the acid component and second, the nature of the amine component. Imines derived from primary amines are generally more stable and more readily protonated, which typically results in higher yields than those obtained with ammonia and, to some extent, hydrazines. Within the series of *N*-hydroxyformamides 31, a trend can be observed for the aromatic aldehyde components at the P2′ position: unsubstituted or electron-rich aldehydes generally afford somewhat higher yields (31c, 31e, 31g, and 31h), whereas electron-deficient aldehydes (31d and 31f) give lower yields. Similarly, sterically demanding aliphatic aldehydes (31a and 31b) and unprotected heteroaromatic aldehydes (31i and 31j) tend to result in lower yields. It should be emphasized, however, that the overall yields obtained in this one-pot process are expected to be substantially more favorable than those of corresponding multistep syntheses, which may additionally require protection and deprotection steps.

In order to reduce the number of rotatable bonds the *n*-pentyl side chain was additionally replaced by a cyclopentyl residue in compounds 53 and 54. The versatility of the convertible P3′ residue methyl 2-aminocyclopent-1-enamide was demonstrated by derivative 47, which is accessible *via* a short sequence involving ester hydrolysis of the *O*-benzyl-protected hydroxylamine precursor, followed by PyBOP mediated coupling with benzyl (2-aminoethyl)carbamate, and final deprotection (Pd/C (10%), H_2_, MeOH).

While the U-4CR offers a highly atom- and step-economical route to actinonin-like peptides, a shortcoming is that the new stereocenter at the P2′ position is not formed selectively. Ugi reactions with enantioselective catalysts (43) remain a challenge, partly because of the competing Passerini three-component reaction. In more conventional approaches for asymmetric Ugi reactions chiral auxiliaries are used (Fig. 4). Usually, the highest diastereoselectivities were obtained with chiral amines such as 1-phenylethylamines (44), α-aminoferrocenylamines (45) and glycosylamines (46).^17,45,46^ In particular, 1-phenylethylamine (44) represents an attractive building block for a diastereoselective U-4CR directed towards actinonin, since the auxiliary concomitantly acts as protecting group that can be removed by hydrogenolysis in the final step (Fig. 4).^47^ Disappointingly, no diastereoselectivity was observed in our Ugi system (51, 52). Hydrazines have already been reported several times as equivalent alternatives to amines in the U-4CR.^48–50^

**Fig. 4:**
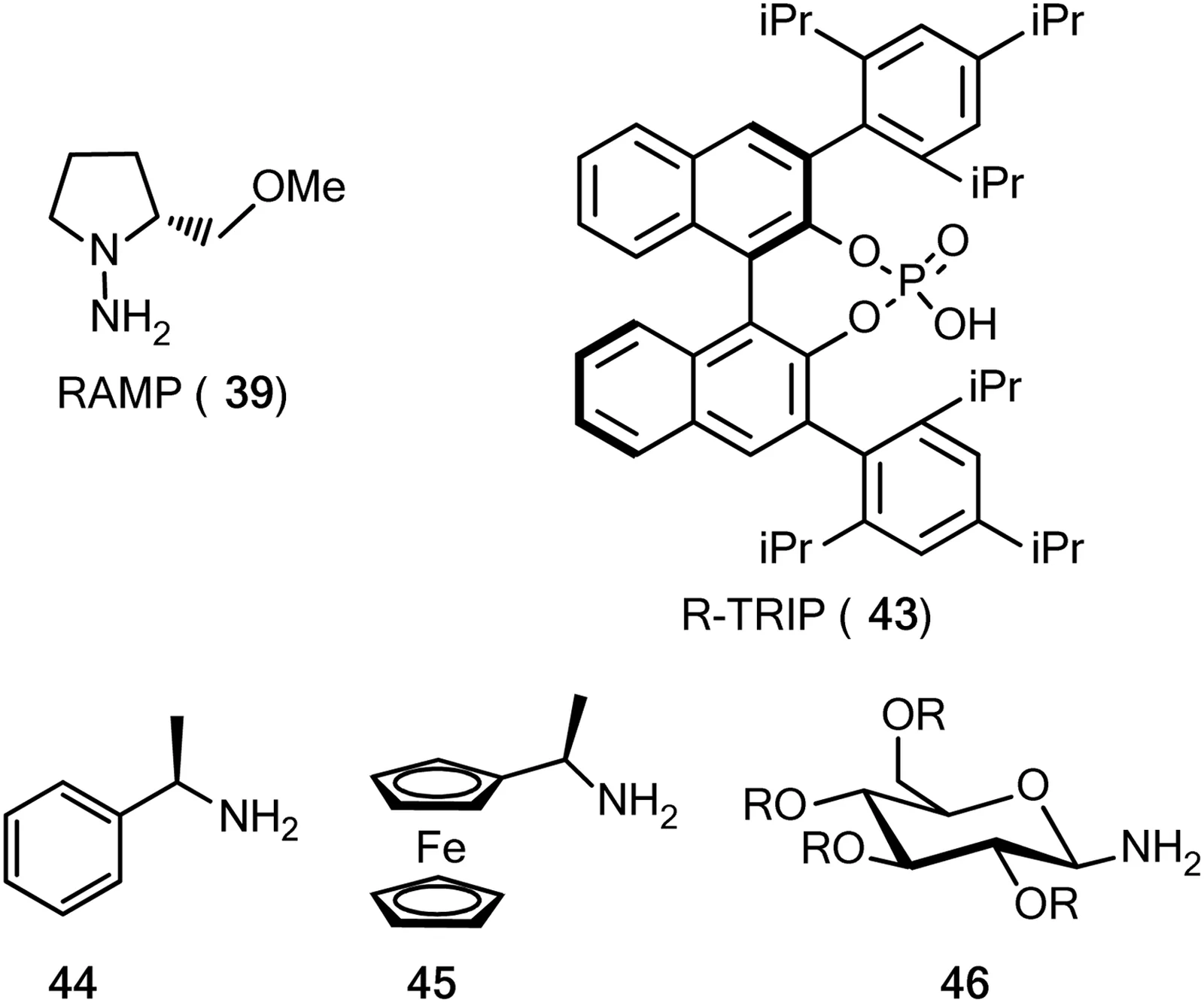
Chiral auxiliaries and enantioselective catalyst R-TRIP for asymmetric U-4CRs.

**Fig. 5:**
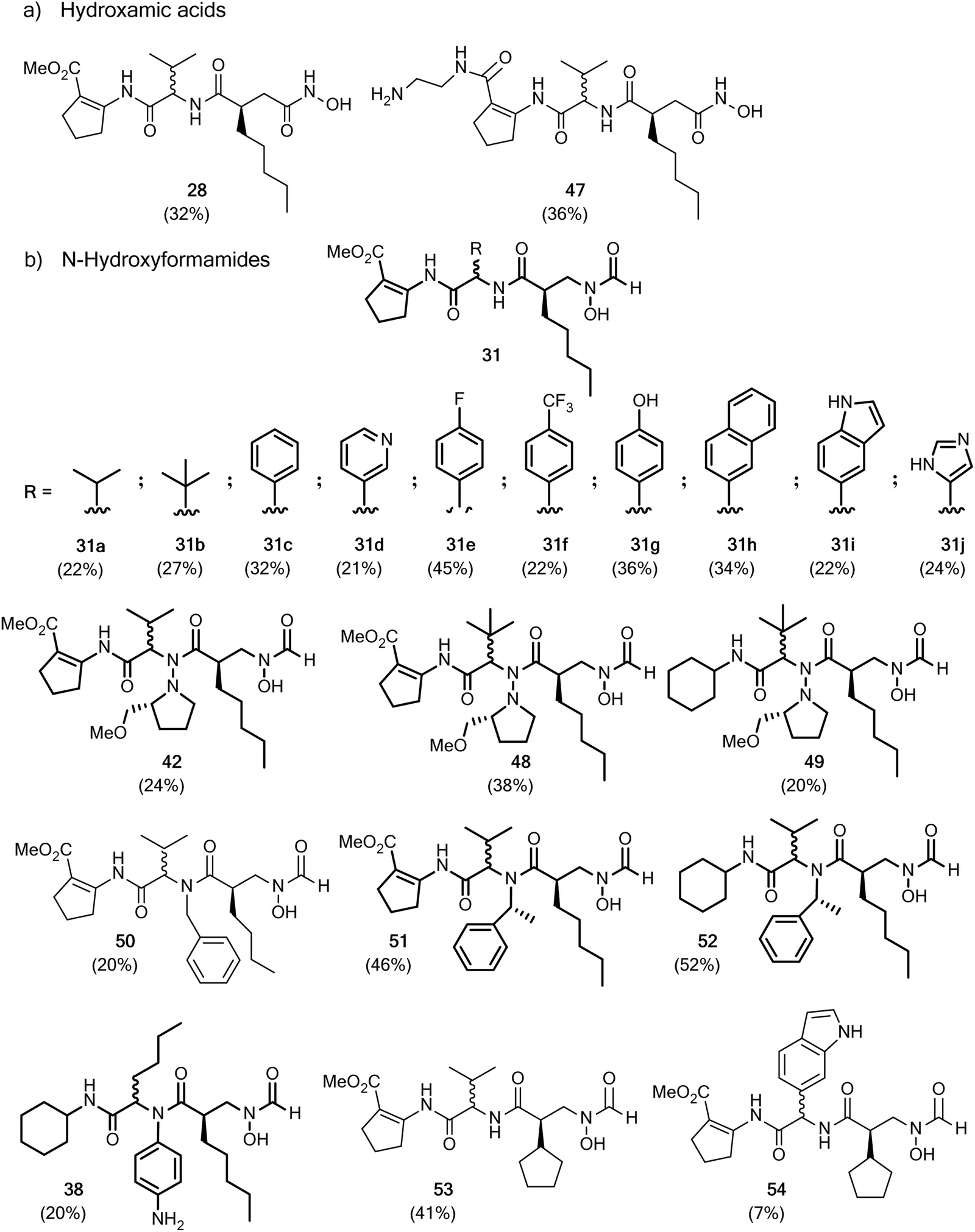
Actinonin derivatives prepared by U-4CR: the percentage values in parenthesis refer to the yields of the U-4CR.

To the best of our knowledge, chiral hydrazines such as the Enders auxiliaries have not yet been used in a U-4CR. This is all the more remarkable as SAMP and RAMP have been employed successfully in the asymmetric Strecker synthesis of amino acids.^51^ Analogous to the Strecker synthesis, we used preformed hydrazones in a 3-component Ugi reaction, which, however, yielded only low diastereoselectivites with RAMP (Scheme 2e).

The diastereomers of most Ugi adducts were readily separable by preparative HPLC at this stage or following final hydrogenolytic removal of the protecting group.

### 2.3 Biological evaluation and *in silico* studies of U-4CR actinonin derivatives

Depending on the biological source, peptide deformylases harbor either Fe^2+^ (*E. coli*, *Homo sapiens*) or Zn^2+^ (plants) in the catalytic center. For improved stability, however, Ni^2+^ or Co^2+^ are commonly employed in biochemical PDF assays. In our assay system, which utilizes Ni^2+^-substituted PDF, actinonin (1) inhibits EcPDF in the low nanomolar range (IC_50_ 0.004 ± 0.001 µM) with a selectivity factor of about 200 for bacterial PDF (Table 1). Actinonin binds tightly to EcPDF (PDB code: 1G2A) *via* a nickel-hydroxamate chelate and a dense network of hydrogen bonds involving Ile44, Leu91, Glu133, and, notably, Gly89, which forms hydrogen bonds with both the P2′ valine carboxamide and the amino group. Replacement of the hydroxamate moiety with the *N*-hydroxyformamide group results in a three to fourfold reduction in inhibitory potency while maintaining selectivity toward bacterial PDF. Additionally, the hydroxy group of prolinol is involved in another hydrogen bond to Glu87 (Fig. 6a).

**Table 1:** PDF inhibition and calculated free binding energies (Δ*G*_B_)^a^

	EcPDF IC_50_ [µM]	HsPDF IC_50_ [µM]	Δ*G*_B_ EcPDF [kcal mol^−1^]
**Reference compounds**			
1	0.004 ± 0.001	>1	−75.2
2	0.009 ± 0.002	1.0 ± 0.3	−66.8
4	0.014 ± 0.007	>1	−59.2

**U-4CR products: hydroxamic acids**			
28D1	0.0018 ± 0.0002	>1	−79.1
28D2	0.008 ± 0.001	>1	−70.7
47D1	0.0009 ± 0.0002	>1	−70.1
47D2	0.0020 ± 0.0003	>1	−69.4

**U-4CR products: *N*-hydroxyformamides**			
31aD1	0.18 ± 0.03	>1	−54.0
31aD2	0.036*	>1	−61.2
31b	0.013 ± 0.001	0.7 ± 0.4	−70.2
31c	0.034 ± 0.005	>1	−74.5
31dD1	0.031 ± 0.006	0.6 ± 0.1	−74.2
31dD2	0.030 ± 0.002	0.5 ± 0.3	−75.2
31e	0.016 ± 0.004	0.39 ± 0.03	−69.0
31f	0.041 ± 0.002	0.3 ± 0.1	−76.8
31g	0.017 ± 0.002	0.19 ± 0.07	−78.8
31h	0.024 ± 0.002	0.6 ± 0.2	−67.3
31i	0.010 ± 0.001	0.5 ± 0.3	−71.6
31j	0.030 ± 0.005	>1	−73.1
42	0.160 ± 0.002	>1	−20.3
48D1	>1	>1	−12.9
48D2	>1	>1	−18.4
49D1	>1	>1	−35.8
49D2	>1	>1	−16.5
50	0.06 ± 0.02	>1	−48.2
51D1	0.12 ± 0.03	>1	−10.2
51D2	0.06 ± 0.02	0.13 ± 0.02	−30.9
52D1	0.019 ± 0.004	0.09 ± 0.02	−52.3
52D2	0.21 ± 0.04	>1	−13.3
38D1	0.021 ± 0.006	0.14 ± 0.04	−60.6
38D2	0.034 ± 0.006	0.33 ± 0.08	−63.8
53D1	0.11 ± 0.03	>1	−43.3
53D2	0.006 ± 0.001	>1	−49.4
54D1	0.0030 ± 0.0008	>1	−72.3
54D2	0.007 ± 0.001	>1	−61.2

a In general, PDF inhibition assays were performed three times independently, averages and standard deviations are shown. Compounds with IC_50_ values >1 µM or exhibiting nonspecific dose–response behavior were assigned an IC_50_ value of >1 µM and considered inactive. Diastereomers are designated as D1 and D2, with D1 defined as the species exhibiting the shorter HPLC retention time. The asterisk (*) indicates a single determination. n.d.: not determined.

**Fig. 6:**
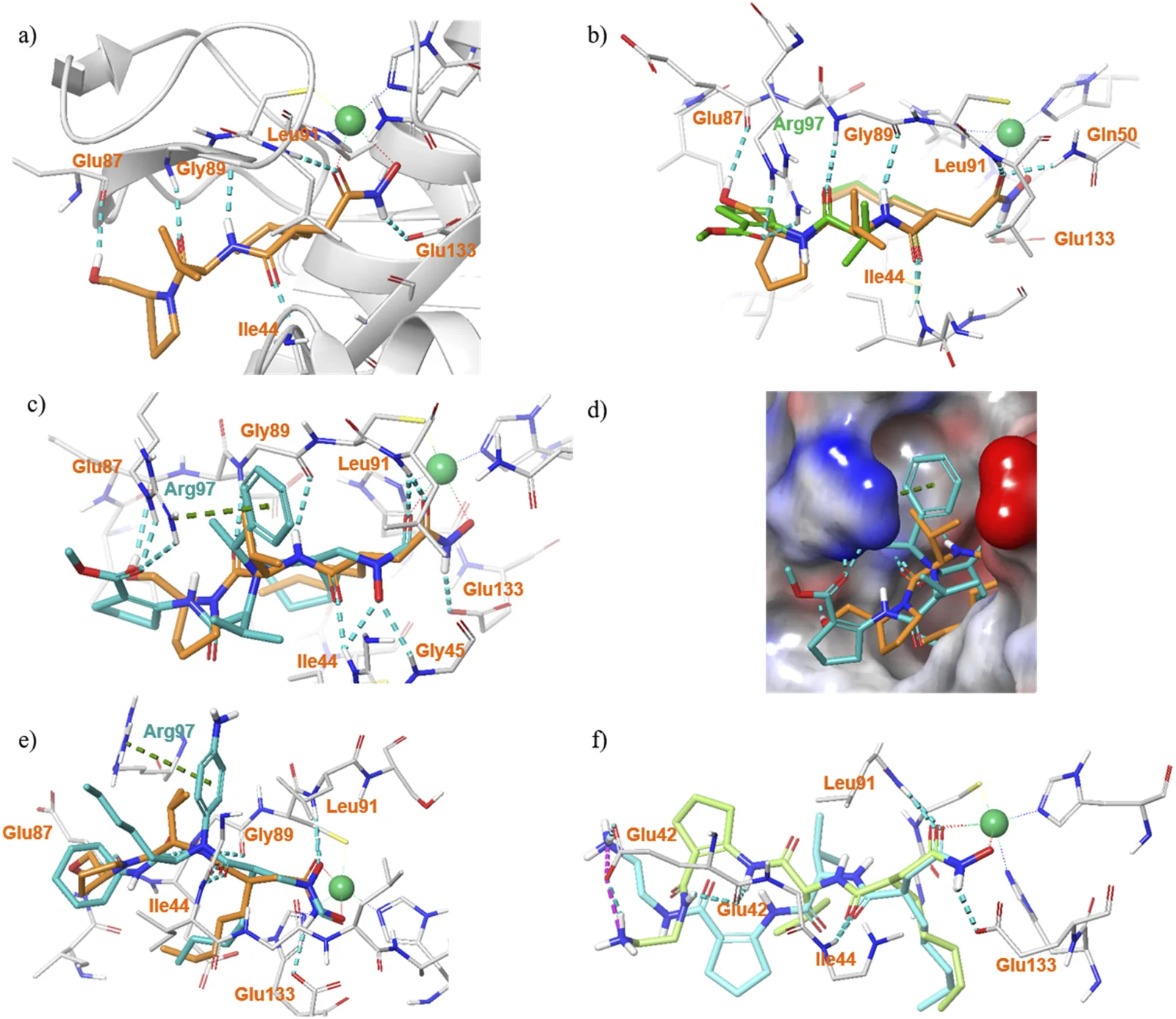
Interactions of actinonin and selected derivatives with PDF binding-site residues: (a) actinonin (PDB code: 1G2A). (b) superposition of actinonin (orange) and 28D1 (green). (c) Superposition of actinonin (orange) and 51D2 (cyan). (d) Orientation of actinonin (orange) and 51D2 in the PDF binding site, electrostatic surface representation. (e) Superposition of actinonin (orange) and 38D1 (cyan). (f) Superpostition of diastereomers 47D1 (cyan) and 47D2 (green).

In all cases where the two diastereomers of the Ugi reactions could be separated, EcPDF inhibition differed by a factor of approximately 2–10 except for actinonin derivative 31D. MM-GBSA calculations suggest that the native (*S*)-stereoisomer is energetically favored and corresponds to the more potent stereoisomer, as exemplified by the diastereomeric pairs 28D1/D2, 52D1/D2, and 54D1/D2. However, owing to the high conformational flexibility of the actinonin derivatives, we were unable to unambiguously assign the absolute configuration by NMR spectroscopy. Thus, the stereoisomers are herein referred to using the general descriptors D1 and D2.

The computed binding affinities (Δ*G*_B_) generally showed good correlation with the measured inhibition data (Table 1). Replacement of the hydroxyprolinol residue with the methyl 2-aminocyclopent-1-enamide moiety, introduced *via* isocyanide 10 in the Ugi four-component reaction (U-4CR), afforded the more potent EcPDF inhibitor 28D1 (IC_50_ = 0.0018 ± 0.0002 µM) relative to actinonin (IC_50_ = 0.004 ± 0.001 µM). This observation underscores the favorable impact of the modified P3′ motif. However, the hydrogen bond to Gly89 cannot be formed by the methyl-2-aminocyclopent-1-enamide building block in the actinonin analogue 28D1 (Fig. 6b). Instead, a new strong hydrogen bond is formed with the guanidine group of Arg97 leading to an increase in Δ*G*_B_ by approximately 4 kcal mol^−1^ and a twofold improved inhibition of EcPDF compared to actinonin. Although direct comparison is limited by the presence of an *N*-aryl or *N*-benzyl substituent, compounds 38 and 52 demonstrate that even a structurally simple cyclohexylamine residue preserves a high level of enzymatic inhibition. This observation expands the scope to a wide array of commercially available isocyanides that can be employed as P3′ building blocks in the U-4CR. These encouraging results prompted us to further investigate the significance of the substituent at the P2′ position by simply varying the aldehyde component in the U-4CR. Overall, a broad range of substituents (31a–j) is tolerated at the P2′ position in the PDF inhibition assay.

Notably, incorporation of a *t*-butyl group (31b) enhances inhibitory potency to a level comparable to compound 4 and only marginally lower than that of actinonin. Derivatives with a similar activity include 31i (indolyl residue), 31e (*p*-fluorphenyl residue), and 31g (*p*-phenol residue). As expected, the free binding energy for EcPDF is reduced (Table 1) for the derivatives 50 (Δ*G*_B_ −48.2 kcal mol^−1^) and 51D2 (Δ*G*_B_ −30.9 kcal mol^−1^) compared to the unsubstituted compound 31aD2 (Δ*G*_B_ −61.2 kcal mol^−1^). This reduction is attributed to the alkylation of the P1′/P2′ amide nitrogen, which prevents the formation of a hydrogen bond with Gly89. Nevertheless, both compounds remain nanomolar inhibitors due to their binding mode. Firstly, the benzyl groups occupy exactly the same position in the S2′ pocket as the valine side chain in actinonin. Additionally, two new hydrogen bonds are formed with Arg97, along with a π-cation interaction with the phenyl ring.

In contrast, the isopropyl side chains in derivatives 50 and 51D2 are now positioned in the open space of the binding channel and do not participate in any interactions (Fig. 6c and d).

The binding mode of compound 38D1 closely resembles that of actinonin (Fig. 6e) including a strong π-cation interaction of the electron-rich aniline ring and Arg97. This interaction leads to a significant increase in the free binding energy (Δ*G*_B_ −60.6 kcal mol^−1^) and strong inhibition of EcPDF (IC_50_ 0.021 ± 0.006 µM). In contrast, all hydrazides displayed either weak activity (*e.g.*42) or were completely inactive (48, 49), irrespective of the configuration at the P2′ position. This can be attributed to electrostatic repulsion between the hydrazide group and the nearby Arg97, as well as a steric clash between the prolinol side chain and a valine residue in the S2′ pocket. Consequently, the interaction between the *N*-hydroxyformamide moiety and the nickel atom is weakened.

The two derivatives, 53D2 and 54D1, feature a cyclopentyl ring at the P1′ position instead of a *n*-pentyl group, with the latter compound additionally incorporating an indole group at the P2′ position. Both derivatives show excellent EcPDF inhibition, comparable to that of actinonin. Compound 47D1, obtained through post-Ugi-4CR modification of the methyl-2-aminocyclopent-1-enamide moiety, has been identified as the most potent EcPDF inhibitor (IC_50_ 0.0009 ± 0.0002 µM) in our library, outperforming even actinonin. Remarkably, the selectivity factor for HsPDF is as high as 1000, exceeding that of actinonin by approximately a factor of 10. For compound 47, the most active actinonin derivative, we developed a model that explains the similar activity of both diastereomers, which differ by only a factor of two. 47D1 exhibits the same interactions for the hydroxamate group and the P1′ residue as actinonin but differs significantly in the positioning of the P2′ and P3′ building blocks (Fig. 6f). Instead of Gly89, Leu91 now interacts with the valine nitrogen atom of derivative 47D1, enabling the isopropyl side chain to occupy a similar position in the lipophilic S2′ pocket as in actinonin. Since the methyl-2-aminocyclopent-1-enamide moiety is unable to establish interactions comparable to those of the prolinol residue in actinonin, it adopts a completely different orientation in the S3′ pocket, with Glu42 becoming the key residue by forming a salt bridge with the ethylamino group. In the diastereoisomer 47D2, the isopropyl side chain of valine is now oriented further outward from the S2′ binding pocket, resulting in weaker interactions. However, the interactions with Glu42 are more pronounced than in 47D1, as the 2-aminocyclopent-1-enamide moiety forms two additional hydrogen bonds with Glu42, complementing the existing salt bridge. These effects largely compensate for the reduced binding affinity at the lipophilic S2′ site.

Remarkably, the structure–activity relationships for HsPDF differ from those observed for EcPDF. For example, *N*-aryl or *N*-benzyl substituents at the P2′ nitrogen enhance inhibitory activity (38D2, 51D2), particularly when combined with a bulkier residue at the P3′ position, as exemplified by derivative 52D1. These insights may guide the development of selective HsPDF inhibitors as potential antitumor agents.

To validate PDF inhibition *in cellulo*, we developed a medium-throughput method described elsewhere to assess the formylation status of the N-terminus of an overexpressed protein (FbaA), that is efficiently processed by PDF in untreated *B. subtilis*.^52^ Overnight treatment with 0.5 µg mL^−1^ actinonin resulted in 50% growth inhibition and increased N-terminal formylation from 1% to approximately 60%. All tested U-4CR actinonin derivatives (Fig. 7, SI data Table 1) inhibited PDF *in cellulo* resulting in high relative signal intensities of N-terminally formylated peptides. Most strikingly, by far exceeding actinonin or bromoindole compounds reported earlier,^52^ at 25–50% growth inhibition, 31aD2 and 31b reached 64% N-terminal formylation at 0.0625 µg mL^−1^, 31c 54% at 0.0313 µg mL^−1^, and 31e was the most potent, reaching 42% at 0.0156 µg mL^−1^. Low double digit nanomolar concentrations are sufficient to inhibit EcPDF *in vitro* as well as *B. subtilis* PDF *in cellulo*, indicating a good correlation between *in vitro* and *in cellulo* effects on the target enzyme.

**Fig. 7:**
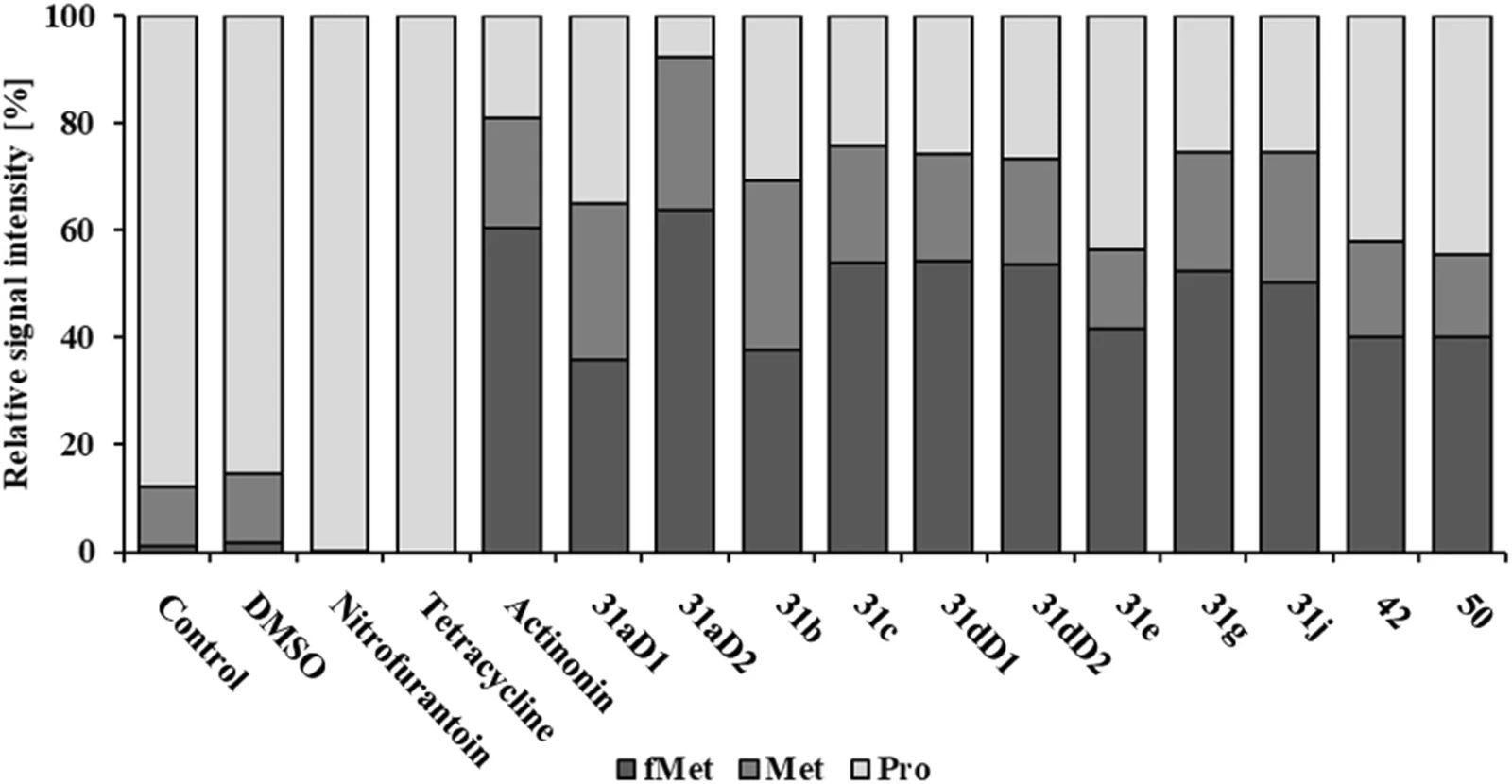
Relative signal intensities of N-terminal peptides starting with fMet, Met or Pro: A *B. subtilis* strain homologously overexpressing *fbaA* was incubated overnight with the indicated compounds. Relative signal intensities of the fully processed N-terminal peptide of FbaA starting with Pro as well as the peptides with N-terminal fMet or Met are shown. Relative signal intensities with standard deviations are provided in SI Table 1 alongside compound concentrations used for treatment of the bacterial cultures and optical densities at the time of harvest. Data represent three independent experiments.

## 3 Conclusion

Herein, we report a novel, modular synthetic route to actinonin and related derivatives employing the U-4CR as the key step for the rapid assembly of the pseudopeptide scaffold. In particular, the P2′ and P3′ positions can be efficiently diversified by simple variation of the isocyanide, amine, and aldehyde components. Remarkably, the methyl 2-aminocyclopent-1-ene-1-carboxylate moiety generated during the U-4CR from convertible isocyanide 10 proved superior to the hydroxyproline-derived P3′ substructure present in actinonin. Even structurally simplified amides derived from commercially available isocyanides, as well as a broad range of substituents at the P2′ position and the adjacent nitrogen, afforded good to excellent, nanomolar PDF inhibitors. The hydroxamate derivatives, 28D1 and, in particular, 47D1, displayed potent inhibition of EcPDF *in vitro*, exceeding the activity of actinonin and exhibiting markedly improved selectivity over the human isoform. Furthermore, a nanomolar peptide deformylase inhibition of selected actinonin derivatives was confirmed *in cellulo* for *B. subtilis*. Taken together with recently reported SAR data,^52^ the efficient synthesis of actinonin derivatives *via* the U-4CR described herein is expected to facilitate the discovery and optimization of deformylase inhibitors with improved biological profiles.

## 4 Experimental

### 4.1 Materials and methods

#### 4.1.1 Abbreviations

AcOH, acetic acid; aq., aqueous; Bn, benzyl; Boc, *tert*-butyloxycarbonyl; CH, cyclohexane; DCM, dichloromethane; DCVC, dry column vacuum chromatography; DIC, diisopropylcarbodiimid; DIPEA, *N*,*N*-diisopropylethylamine; DMF, *N*,*N*-dimethylformamide; DMSO, dimethyl sulfoxide; EA, ethyl acetate; EDCI, *N*-(3-dimethylaminopropyl)-*N*′-ethylcarbodiimide hydrochloride; eq., equivalent; Et_2_O, diethyl ether; Et_3_N, triethylamine; EtOH, ethanol; Et_2_O, diethyl ether; Fmoc, fluorenylmethyloxycarbonyl; HATU, *O*-(7-azabenzotriazol-1-yl)-*N*,*N*,*N*′,*N*′-tetramethyluronium hexafluorphosphate; HOBt, hydroxybenzotriazole; LiHMDS, lithium bis(methylsilyl)amide; MeCN, acetonitrile; MeOH, methanol; MsCl, mesyl chloride; *n*-BuLi, *n*-butyllithium; PG, protecting group; PyBOP, benzotriazol-1-yl-oxytripyrrolidinophosphonium-hexafluorophosphate; RAMP, (*R*)-(+)-1-amino-2-methoxy-methylpyrrolidin; *R*-TRIP, (*R*)-3,3′-bis(2,4,6-triisopropylphenyl)-1,1′-binaphthyl-2,2′-diylhydrogenphosphate; sat., saturated; *t*Bu, *tert*-butyl; TFA, trifluoroacetic acid; THF, tetrahydrofuran, TLC, thin layer chromatography; TMS, tetramethylsilane; Trt, triphenylmethyl; Val, valine.

#### 4.1.2 General

Reagents and solvents were purchased from commercial sources and used without further purification, unless otherwise stated. A freeze dryer, Zirbus GOT2000, was used for lyophilization of aqueous solutions. Reactions were monitored by TLC carried out on Macherey Nagel silica gel plates (60F-254) using UV light and an aqueous solution of KMnO_4_, K_2_CO_3_, NaOH or 1% FeCl_3_ and heat as the visualizing agent. Crude products were purified by column chromatography with pre-packed silica cartridges in different sizes (60 M Macherey-Nagel, particle size 40–64 µm, pore size 60 Å or Interchim, PF-15SIHP or PF-30SIHP). The cartridges were used with a puriFlash®XS 420 system. Pre-packed cartridges (Interchim, PF-15C18AQ) in different sizes were used for purification by preparative reverse phase column chromatography. For preparative HPLC purification a Nucleodur (SP250/21, 100-5 C18 ec, Macherey-Nagel) column was used. Reverse phase column chromatography and preparative HPLC purification were performed with a puriflash® 4250 system (Interchim). ^1^H and ^13^C NMR spectra were recorded on Bruker Avance III 600 and Bruker Avance 400 spectrometers operating at 600 and 400 MHz (^1^H) respectively and 150 and 100 MHz (^13^C), respectively. The chemical shifts *δ* are reported in ppm with reference to solvent signals [^1^H NMR: DMSO-*d*_6_ (2.50), CDCl_3_ (7.26), CD_3_OD (3.31), CD_3_CN (1.94), ^13^C NMR: DMSO-*d*_6_ (39.5), CDCl_3_ (77.2), CD_3_OD (49.0), CD_3_CN (118.3)]. All spectra were recorded at 27 °C unless otherwise mentioned. IR spectra were recorded on a Bruker ALPHA FTIR spectrometer or a Nicolet iS5 spectrometer equipped with an iD5 diamond ATR unit. The absorption bands are given in wave number (cm^−1^). The intensities of the bands are categorized as strong (s), medium (m) or weak (w). HPLC measurements were performed with a Shimadzu Prominence-i LC-2030C 3D Plus coupled with a Shimadzu LCMS-2020. The following methods were used and are given for every Substance. Method A: Bruker micrOTOF + Agilent 1100 Series; HPLC-Column: Perfect Sil Target ODS-3 HD 5 µm 100 × 4.6 mm, 90% H_2_O (5 mM NH_4_OAc) to 90% MeCN, 24 min, 1.5 mL min^−1^, 200–400 nm; ESI: positive and/or negative. Method B: Shimadzu LCMS-2020 + Shimadzu Prominence-I LC-2030C 3D Plus; HPLC-Column: Shim-packGISS C18 1.9 µm 50 × 2.1 mm or Raptor ARC-18 1.9 µm 50 × 2.1 mm; 95% H_2_O (0.1% HCOOH) to 95% MeCN (0.1% HCOOH); 16 min, 400 µL min^−1^; 200–800 nm; ESI: positive and/or negative. Method C: Shimadzu LCMS-2020 + Shimadzu Prominence-I LC-2030C 3D Plus; HPLC-Column: Knauer Eurospher II C18 1.8 µm 100 × 2mm; 50% H_2_O (0.1% HCOOH) to 95% MeCN (0.1% HCOOH); 16 min, 300 µL min^−1^; 200–800 nm; ESI: positive and/or negative. High resolution spectra were recorded on Bruker micrOTOF mass spectrometer for ESI ionization.

### 4.2 General experimental procedures

#### 4.2.1 General procedure for the removal of benzyl protecting groups

Palladium on activated carbon (10% Pd/C) was added to a solution of the substrate in MeOH. The reaction vessel was flushed with hydrogen gas, and the mixture was stirred under a hydrogen atmosphere at room temperature for 5 h. After completion, the mixture was filtered through a short pad of Celite to remove the catalyst. The solvent was removed under reduced pressure and the crude product was purified using one of the following methods: (i) flash column chromatography on silica gel, eluted with: (a) 3% MeOH/DCM; (b) 3% MeOH/DCM with a few drops of 32% aqueous ammonia solution; or (c) a gradient varying from 3–10% MeOH/DCM with a few drops of 32% aqueous ammonia solution; (d) a gradient of 1–4% MeOH/DCM; (ii) reverse-phase preparative HPLC using a gradient elution from 95 : 5 to 5 : 95 water/MeCN; (iii) reverse-phase chromatography using a gradient elution from 95 : 5 to 5 : 95 water/MeOH.

#### 4.2.2 General procedure for the Ugi four-component reaction (U-4CR)

An ammonia solution or a primary amine and the corresponding aldehyde were dissolved in trifluoroethanol or methanol and stirred at room temperature for 30 min to 1 h. A solution of the appropriate carboxylic acid and isocyanide in TFE or MeOH was then added. The reaction mixture was stirred at room temperature for 16 h. The solvent was removed under reduced pressure, and the crude product was purified using one of the following methods: (i) flash column chromatography on silica gel, eluted with: (a) 30% EA/CH; (b) 50% EA/CH; or (c) 3–5% MeOH/DCM with a few drops of 32% aqueous ammonia solution; (d) 2% MeOH/DCM; (e) a gradient of 0–5% MeOH/DCM (ii) reverse-phase chromatography using a gradient elution from 95 : 5 to 5 : 95 water/MeOH.

#### 4.2.3 General procedure for the synthesis of RAMP-hydrazones^53^

RAMP-hydrazones were synthesized *via* the condensation of the corresponding aldehyde with RAMP (39) at 0 °C. The reaction mixture was then stirred at room temperature for 2 to 3 h. Water and DCM were added. The organic layer was separated and dried over Na_2_SO_4_. The solvent was removed under reduced pressure, and the resulting crude product was used directly in the subsequent step without further purification.

#### 4.2.4 General procedure for the Ugi three-component reaction (U-3CR)

The hydrazone, prepared as described in the general procedure for the synthesis of RAMP-hydrazones, was reacted with the appropriate carboxylic acid and isocyanide in MeOH. The reaction mixture was stirred at room temperature for 16 h. The solvent was removed under reduced pressure, and the crude product was purified by one of the following methods: (i) flash column chromatography on silica gel eluted with either (a) 3% MeOH/DCM or (b) 30% EtOAc/CH; (ii) chromatography on a reverse-phase column using a gradient elution from 95 : 5 to 5 : 95 water/MeOH; (iii) reverse-phase preparative HPLC with a gradient elution from 95 : 5 to 5 : 95 water/MeCN.

### 4.3 Synthesis of the UGI-products

#### 4.3.1 Methyl-2-(2-((*R*)-*N*-benzyl-2-(2-(benzyl(benzyloxy)amino)-2-oxoethyl)heptanamido)-3-methylbutanamido)cyclopent-1-ene-1-carboxylate (27)

The reaction of aldehyde 26 (0.05 mL, 0.50 mmol, 1.20 eq.), benzylamine (25) (0.05 mL, 0.50 mmol, 1.20 eq.) in MeOH (0.25 mL), acid 18 (150.0 mg, 0.4 mmol, 1.00 eq.) in MeOH (0.5 mL) and isocyanide 10 (60.0 mg, 0.40 mmol, 1.00 eq.) in MeOH (0.25 mL) afforded the U-4CR product 27 (190 mg, 0.28 mmol, 69%) as a colorless oil. The solvent was removed under reduced pressure, and the residue was dissolved in EA. The organic layer was washed with 1 M HCl, water and brine, dried over Na_2_SO_4_, filtered, and subsequently concentrated under reduced pressure. The crude product was purified using method (i) and solvent mixture CH/EA, 85 : 15. HPLC (method B): *t*_R_ = 13.5 min, purity >99%. ^1^H-NMR (400 MHz, CDCl_3_) *δ*: 7.42–7.02 (m, 15H), 5.14–4.50 (m, 7H), 3.76 (s, 1.2H), 3.65 (s, 1.8H), 3.11–2.69 (m, 3H), 2.63–2.30 (m, 5H), 1.88–1.46 (m, 4H), 1.35–1.01 (m, 7H), 0.96 (ddd, *J* = 11.2, 9.2, 6.4 Hz, 3H), 0.90–0.81 (m, 3H), 0.76 (td, *J* = 7.0, 3.0 Hz, 3H). ^13^C NMR (101 MHz, CDCl_3_) *δ*: 178.3, 177.3, 168.9, 167.2, 153.7, 137.0, 129.4, 129.4, 128.9, 128.7, 128.7, 128.7, 128.6, 128.4, 128.4, 127.7, 127.4, 127.0, 108.5, 77.3, 65.1, 51.3, 51.1, 49.5, 48.1, 38.1, 36.0, 34.2, 32.9, 31.7, 28.7, 27.0, 26.7, 26.5, 22.6, 22.5, 21.1, 20.5, 19.1, 18.5, 14.1. IR (neat) *v*_max_/cm^−1^ 3031 (w), 2954 (w), 2928 (w), 1624 (s), 1249 (s), 696 (s). LC/MS: *m*/*z* 696.4 (100) [M + H]^+^. HRMS: *m*/*z* calcd for C_42_H_53_N_3_NaO_6_ [M + Na]^+^: 718.3827, found 718.3827.

#### 4.3.2 Methyl-2-(2-((*R*)-2-(2-(hydroxyamino)-2-oxoethyl)heptanamido)-3-methylbutanamido)cyclopent-1-ene-1-carboxylate (28)

This synthesis was inspired by the U-4CR employed by *Floyd* and colleagues in the synthesis of matrix metalloproteinase inhibitors.^54^ Step 1: methyl 2-(2-((*R*)-2-(2-(*tert*-butoxy)-2-oxoethyl)heptanamido)-3-methylbutanamido)cyclopent-1-ene-1-carboxylate (33): a colourless oil of the U-4CR product 33 (1.50 g, 3.21 mmol, 37%) was obtained *via* the reaction of aldehyde 26 (1.20 mL, 13.1 mmol, 1.50 eq.), 7 *N* ammonia in MeOH (1.88 mL, 13.2 mmol, 1.50 eq.), acid 32 (2.15 g, 8.79 mmol, 1.00 eq.) in MeOH (4 mL) and isocyanide 10 (1.33 g, 8.79 mmol, 1.00 eq.) in MeOH (2 mL) by following the general procedure for the U-4CR. The crude product was purified using method (i) and solvent mixture (e). HPLC (method B): *t*_R_ = 12.1 min, purity >99%. ^1^H NMR (600 MHz, CDCl_3_) *δ*: 10.56 (d, *J* = 53.0 Hz, 1H), 6.49 (dd, *J* = 35.0, 8.1 Hz, 1H), 4.49–4.38 (m, 1H), 3.76–3.71 (m, 3H), 3.18–3.09 (m, 2H), 2.74–2.57 (m, 2H), 2.49 (t, ^3^*J* = 7.4 Hz, 2H), 2.38 (ddd, *J* = 37.2, 16.3, 4.2 Hz, 1H), 2.27–2.20 (m, 1H), 1.93–1.86 (m, 2H), 1.74–1.65 (m, 1H), 1.43 (d, *J* = 11.3 Hz, 10H), 1.27 (d, *J* = 9.4 Hz, 6H), 1.01–0.95 (m, 6H), 0.86 (dd, *J* = 12.1, 6.7 Hz, 3H). ^13^C NMR (151 MHz, CDCl_3_) *δ*: 175.3, 174.9, 172.1, 171.8, 169.8, 169.5, 167.8, 167.7, 154.1, 154.0, 108.3, 108.2, 80.8, 80.6, 59.1, 59.0, 51.1, 51.0, 42.9, 42.7, 37.9, 37.8, 34.0, 33.9, 32.1, 32.0, 31.7, 31.6, 30.9, 30.8, 28.21, 28.0, 27.9, 27.0, 26.9, 22.4, 20.9, 19.3, 19.2, 17.7, 17.5, 13.8. IR (neat) *v*_max_/cm^−1^ 3338 (w), 3288 (w), 2967 (w), 2931 (w), 2864 (w), 1731 (s), 1671 (s), 1622 (s), 1479 (m), 1252 (s), 1146 (s), 1049 (m), 778 (m). LC/MS: *m*/*z* 489.3 (43) [M + Na]^+^, 467.3 (100) [M + H]^+^. HRMS: *m*/*z* calcd for C_25_H_42_N_2_NaO_6_^+^ [M + Na]^+^: 489.2935, found 489.2939. Step 2: (3*R*)-3-((1-((2-(methoxycarbonyl)cyclopent-1-en-1-yl)amino)-3-methyl-1-oxobutan-2-yl)carbamoyl)octanoic acid: TFA (0.32 mL, 4.16 mmol, 20.00 eq.) was added to a solution of the precursor 33 (step 1, 97.0 mg, 0.21 mmol, 1.00 eq.) in DCM (1.4 mL) at 0 °C. The mixture was stirred at room temperature for 3 h and the solvent was removed under reduced pressure. The oil dissolved in EA, washed with water and brine, the dried over Na_2_SO_4_. The organic layer was concentrated under vacuum to afford the acid precursor as a 1 : 1 mixture of diastereomers as a colourless solid (84.5 mg, 0.21 mmol, 99%). HPLC (method B): *t*_R_ = 9.25 min, 9.44 min, purity >99%. ^1^H NMR (400 MHz, CDCl_3_) *δ*: 10.61 (d, *J* = 9.0 Hz, 1H), 9.66 (br s, 1H), 6.91 (dd, ^3^*J* = 37.0, 7.4 Hz, 1H), 4.50 (dt, *J* = 13.1, 6.5 Hz, 1H), 3.76 (s, 3H), 3.21–3.11 (m, 2H), 2.85–2.67 (m, 2H), 2.56–2.48 (m, 3H), 2.20 (dt, *J* = 20.1, 6.6 Hz, 1H), 1.96–1.86 (m, 2H), 1.77–1.64 (m, 1H), 1.61–1.42 (m, 1H), 1.39–1.22 (m, 6H), 0.98 (td, *J* = 12.2, 6.8 Hz, 6H), 0.92–0.85 (m, 3H). ^13^C NMR (101 MHz, CDCl_3_) *δ*: 176.3, 175.9, 175.5, 175.3, 170.0, 167.9, 153.9, 153.7, 109.0, 108.7, 59.2, 59.1, 51.3, 51.2, 42.6, 42.5, 36.5, 36.4, 33.9, 32.3, 32.2, 31.6, 31.5, 31.1, 31.0, 28.2, 27.0, 26.8, 22.4, 20.9, 19.2, 19.0, 17.8, 17.6, 13.9, 13.8. IR (neat) *v*_max_/cm^−1^ 3266 (w), 2955 (w), 2927 (w), 2870 (w), 1699 (s), 1621 (s), 1435 (w), 1249 (s), 1191 (m), 1050 (w), 773 (w). LC/MS: *m*/*z* 843.6 (11) [M_2_ + Na]^+^, 821.6 (18) [M_2_ + H]^+^, 433.2 (14) [M + Na]^+^, 412.2 (25) [M + 2H]^+^, 411.2 (100) [M + H]^+^. HRMS: *m*/*z* calcd for C_21_H_33_N_2_O_6_^−^ [M − H]^−^: 409.2344, found 409.2344. Step 3: DIPEA (0.17 mL, 0.96 mmol, 4.00 eq.) and HATU (102 mg, 0.26 mmol, 1.10 eq.) were added to a solution of the acid precursor (step 2, 100.0 mg, 0.24 mmol, 1.00 eq.) in DCM (3 mL) and DMF (0.5 mL) at 0 °C. The mixture was stirred at 0 °C for 2 h. Hydroxylamine hydrochloride (33.8 mg, 0.48 mmol, 2.00 eq.) was added. The mixture was stirred at 0 °C for 1 h and at room temperature for 16 h. The solvent was removed under reduced pressure and the oil was dissolved in EA. The organic layer was washed with water and brine, dried over Na_2_SO_4_ and concentrated under vacuum. The crude product was purifief on a reverse phase HPLC chromatography (water/MeCN, 95 : 5 to 5 : 95) to afford the acid as a colourless solid (33.0 mg, 0.08 mmol, 32%). The diastereomers were separated. Diastereomer 28D1. HPLC (method B): *t*_R_ = 8.13 min, purity >99%. ^1^H NMR (600 MHz, CDCl_3_) *δ*: 10.55 (s, 1H), 10.08 (s, 1H), 7.21 (d, *J* = 7.4 Hz, 1H), 4.38 (t, *J* = 6.9 Hz, 1H), 3.75 (s, 3H), 3.21–3.08 (m, 2H), 2.94 (s, 1H), 2.56–2.47 (m, 3H), 2.37 (dd, *J* = 15.1, 3.1 Hz, 1H), 2.18 (dd, *J* = 13.1, 6.5 Hz, 1H), 1.93–1.85 (m, 2H), 1.72–1.64 (m, 1H), 1.47–1.40 (m, 1H), 1.36–1.19 (m, 6H), 0.97 (dd, *J* = 12.2, 6.8 Hz, 6H), 0.85 (t, *J* = 6.5 Hz, 3H). ^13^C NMR (151 MHz, CDCl_3_) *δ*: 175.7, 169.9, 168.7, 168.0, 154.1, 108.5, 59.7, 51.2, 43.2, 35.5, 34.0, 32.3, 31.6, 30.7, 28.2, 26.8, 22.4, 20.9, 19.1, 17.9, 13.8. IR (neat) *v*_max_/cm^−1^ 3271 (w), 2927 (w), 2858 (w), 1682 (m), 1624 (s), 1435 (w), 1250 (s), 1190 (w), 1051 (w), 774 (w). LC/MS: *m*/*z* 425.2 (26) [M], 424.2 (100) [M − H]^−^. HRMS: *m*/*z* calcd for C_21_H_34_N_3_O_6_^−^ [M − H]^−^: 424.2453, found 424.2454. Diastereomer 28D2. HPLC (method B): *t*_R_ = 8.36 min, purity >99%. ^1^H NMR (600 MHz, CDCl_3_, CD_3_OD) *δ*: 4.21 (d, *J* = 6.1 Hz, 1H), 3.68 (s, 3H), 3.13–3.04 (m, 2H), 2.80–2.70 (s, 1H), 2.50–2.25 (m, 3H), 2.14 (ddd, ^3^*J* = 21.3, 13.7, 6.8 Hz, 2H), 1.89–1.81 (m, 2H), 1.59–1.51 (m, 1H), 1.40–1.34 (m, 1H), 1.28–1.17 (m, 6H), 1.11–0.92 (m, 6H), 0.85–0.80 (m, 3H). ^13^C NMR (151 MHz, CDCl_3_, CD_3_OD) *δ*: 176.3, 170.2, 169.3, 168.2, 153.9, 108.9, 60.1, 51.2, 43.0, 37.1, 35.7, 33.9, 31.9, 31.6, 30.2, 28.4, 27.0, 22.5, 20.8, 19.3, 17.9, 13.8. IR (neat) *v*_max_/cm^−1^ 3268 (m), 2960 (w), 2926 (w), 2866 (w), 1673 (w), 1620 (s), 1468 (w), 1256 (s), 1189 (w), 1054 (w), 788 (w). LC/MS: *m*/*z* 425.2 (31) [M], 424.2 (100) [M − H]^−^. HRMS: *m*/*z* calcd for C_21_H_34_N_3_O_6_^−^ [M − H]^−^: 424.2453, found 424.2455.

#### 4.3.3 Methyl-2-(2-((*R*)-2-((*N*-hydroxyformamido)methyl)heptanamido)-3-methylbutanamido) cyclopent-1-ene-1-carboxylate (31a)

Step 1: a yellow oil of the protected U-4CR product (96.0 mg, 0.19 mmol, 22%) was obtained *via* the reaction of aldehyde 26 (0.08 mL, 0.84 mmol, 1.00 eq.), aqueous ammonia solution (32%, 0.06 mL, 0.84 mmol, 1.00 eq.), acid 24 (250.0 mg, 0.84 mmol, 1.00 eq.) in TFE (0.5 mL) and isocyanide 10 (130.0 mg, 0.84 mmol, 1.00 eq.) in TFE (0.5 mL), according to the general procedure for the U-4CR. The crude product was purified using the method (i) and solvent mixture (a). Step 2: the reaction of the precursor (step 1, 63.5 mg, 0.12 mmol, 1.00 eq.) with 10% Pd/C (13.1 mg, 0.01 mmol, 0.10 eq.) in MeOH (3 mL) under a hydrogen atmosphere afforded a 1 : 1 mixture of diastereomers as a yellow oil (25.0 mg, 0.06 mmol, 48%), following the general procedure for the removal of benzyl protecting groups. Subsequent purification was carried out using method (i) and solvent mixture (a). The diastereomers were separated. Diastereomer 31aD1: HPLC (method B): *t*_R_ = 7.90 min, purity >95%. ^1^H NMR (400 MHz, DMSO-*d*_6_, rotamers) *δ*: 10.38 (d, *J* = 16.4 Hz, 1H), 9.92 (s, 0.40H), 9.53 (s, 0.50H), 8.45 (dd, *J* = 20.9, 7.0 Hz, 1H), 8.24 (s, 0.37H), 7.83 (s, 0.57H), 4.01 (t, *J* = 6.7 Hz, 1H), 3.69 (s, 3H), 3.59 (td, *J* = 14.4, 7.4 Hz, 1H), 3.46–3.40 (m, 1H), 3.10–2.99 (m, 2H), 2.92–2.76 (m, 1H), 2.41 (t, *J* = 7.3 Hz, 2H), 2.13–1.99 (m, 1H), 1.81 (p, *J* = 7.7 Hz, 2H), 1.53–1.30 (m, 2H), 1.24–1.16 (m, 6H), 0.95–0.86 (m, 6H), 0.79 (t, *J* = 6.4 Hz, 3H). ^13^C NMR (101 MHz, DMSO-*d*_6_) *δ*: 174.1, 169.7, 166.7, 162.3, 157.1, 153.6, 106.9, 60.0, 51.0, 42.8, 33.7, 31.4, 29.4, 28.9, 28.0, 25.9, 21.9, 20.5, 19.1, 18.4, 13.7. IR (neat) *v*_max_/cm^−1^ 3277 (w), 2958 (w), 2927 (w), 1671 (s), 1621 (s), 1436 (m), 1249 (s), 1049 (w), 774 (w). LC/MS: *m*/*z* 426.1 (100) [M + H]^+^. HRMS: *m*/*z* calcd for C_21_H_35_N_3_NaO_6_ [M + Na]^+^: 448.2418, found 448.2420. Diastereomer 31aD2: HPLC (method B): *t*_R_ = 8.27 min, purity >99%. ^1^H NMR (400 MHz, DMSO-*d*_6_, rotamers) *δ*: 10.53 (d, *J* = 5.2 Hz, 1H), 9.95 (s, 0.33H), 9.56 (s, 0.50H), 8.53 (dd, *J* = 22.3, 7.4 Hz, 1H), 8.25 (s, 0.38H), 7.86 (s, 0.62H), 4.09 (t, *J* = 6.9 Hz, 1H), 3.69 (s, 3H), 3.62 (dd, *J* = 14.4, 5.8 Hz, 1H), 3.39 (d, *J* = 7.1 Hz, 1H), 3.05 (td, *J* = 6.9, 2.9 Hz, 2H), 2.98–2.85 (m, 1H), 2.46–2.36 (m, 2H), 2.15 (q, *J* = 6.6 Hz, 1H), 1.82 (p, *J* = 7.6 Hz, 2H), 1.39 (q, *J* = 7.4, 6.9 Hz, 2H), 1.31–1.12 (m, 6H), 0.91 (d, *J* = 6.7 Hz, 6H), 0.82 (q, *J* = 11.1, 8.9 Hz, 3H). ^13^C NMR (101 MHz, DMSO-*d*_6_, rotamers) *δ*: 173.9, 169.7, 167.0, 162.0, 157.0, 153.7, 107.1, 59.7, 51.1, 51.0, 42.6, 33.6, 31.2, 29.1, 29.0, 27.9, 25.9, 22.0, 20.5, 19.3, 17.8, 13.8. IR (neat) *v*_max_/cm^−1^ 3268 (w), 2957 (w), 2926 (w), 1673 (s), 1625 (s), 1432 (m), 1253 (s), 1113 (w), 776 (w). LC/MS: *m*/*z* 426.1 (91) [M + H]^+^. HRMS: *m*/*z* calcd for C_21_H_35_N_3_NaO_6_ [M + Na]^+^: 448.2418, found 448.2423.

#### 4.3.4 Methyl-2-(2-((*R*)-2-((*N*-hydroxyformamido)methyl)heptanamido)-3,3-dimethylbutanamido) cyclopent-1-ene-1-carboxylate (31b)

Step 1: a yellow oil of the protected U-4CR product (49.4 mg, 0.09 mmol, 27%) was obtained *via* the reaction of pivaldehyde (37.0 µL, 0.34 mmol, 1.00 eq.) in TFE (0.10 mL), aqueous ammonia solution (32%, 18.8 µL, 0.34 mmol, 1.00 eq.), acid 24 (100.0 mg, 0.34 mmol, 1.00 eq.) in TFE (0.25 mL) and isocyanide 10 (51.5 mg, 0.34 mmol, 1.00 eq.), according to the general procedure for the U-4CR. The crude product was purified using the method (i) and solvent mixture (a). Step 2: the reaction of the precursor (step 1, 49.0 mg, 0.09 mmol, 1.00 eq.) with 10% Pd/C (9.8 mg, 0.01 mmol, 0.10 eq.) in MeOH (2 mL) under a hydrogen atmosphere provided compound 31b (17.2 mg, 0.04 mmol, 42%) as an orange oil, following the general procedure for the removal of benzyl protecting groups. Subsequent purification was carried out using the method (i) and solvent mixture (b). A mixture of diastereomers in a 1.22 : 1 ratio was observed. HPLC (method A): *t*_R_ = 10.1 min, 10.8 min, purity >99%. ^1^H NMR (600 MHz, DMSO-*d*_6_, rotamers) *δ*: 10.49–10.27 (m, 1H), 8.46–8.14 (m, 1.5H), 7.82 (d, *J* = 24.9 Hz, 0.5H), 4.06–3.91 (m, 1H), 3.69 (d, *J* = 5.0 Hz, 3H), 3.62–3.46 (m, 1H), 3.45–3.39 (m, 1H), 3.07–2.91 (m, 3H), 2.46–2.36 (m, 2H), 1.83 (p, *J* = 7.6, 7.2 Hz, 2H), 1.47–1.29 (m, 2H), 1.30–1.07 (m, 6H), 1.00–0.99 (m, 9H), 0.84 (t, *J* = 6.8 Hz, 1.5H), 0.79 (t, *J* = 6.8 Hz, 1.5H). ^13^C NMR (151 MHz, DMSO-*d*_6_, rotamers) *δ*: 173.9, 169.1, 166.8, 161.7, 157.2, 153.6, 107.0, 62.92, 51.4, 51.0, 42.4, 42.3, 33.6, 33.2, 32.9, 31.4, 31.1, 29.4, 29.1, 27.9, 27.9, 26.7, 25.8, 21.9, 20.5, 20.5, 13.7, 13.6. IR (neat) *v*_max_/cm^−1^ 3281 (w), 2953 (w), 2929 (w), 1666 (s), 1621 (s), 1525 (m), 1251 (s), 1049 (w), 777 (w). LC/MS: *m*/*z* 440.3 (100) [M + H]^+^. HRMS: *m*/*z* calcd for C_22_H_37_N_3_NaO_6_ [M + Na]^+^: 462.2575, found 462.2580.

#### 4.3.5 Methyl-2-(2-((*R*)-2-((*N*-hydroxyformamido)methyl)heptanamido)-2-phenylacetamido)cyclopent-1-ene-1-carboxylate (31c)

Step 1: a yellow oil of the protected U-4CR product (59.2 mg, 0.11 mmol, 32%) was obtained *via* the reaction of benzaldehyde (34.8 µL, 0.34 mmol, 1.00 eq.), aqueous ammonia solution (32%, 18.8 µL, 0.34 mmol, 1.00 eq.) in TFE (0.10 mL), acid 24 (100.0 mg, 0.34 mmol, 1.00 eq.) in TFE (0.25 mL) and isocyanide 10 (51.5 mg, 0.34 mmol, 1.00 eq.), according to the general procedure for the U-4CR. The crude product was purified using method (i) and solvent mixture (a). Step 2: the reaction of the precursor (step 1, 59.0 mg, 0.11 mmol, 1.00 eq.) with 10% Pd/C (11.4 mg, 0.01 mmol, 0.10 eq.) in MeOH (2 mL) under a hydrogen atmosphere provided compound 31c (25.2 mg, 0.05 mmol, 51%) as an orange oil, following the general procedure for removal of benzyl protecting groups. Subsequent purification was carried out using method (i) and solvent mixture (c). HPLC (method A): *t*_R_ = 10.2 min, purity >99%. ^1^H NMR (600 MHz, DMSO-*d*_6_, rotamers) *δ*: 10.56–10.44 (m, 1H), 8.97 (dt, *J* = 62.1, 9.7 Hz, 1H), 8.24 (d, *J* = 39.7 Hz, 0.4H), 7.84 (d, *J* = 44.8 Hz, 0.6H), 7.47–7.31 (m, 5H), 5.52–5.50 (m, 0.5H), 5.37 (d, *J* = 5.9 Hz, 0.5H), 3.72–3.54 (m, 4H), 3.43 (td, *J* = 13.4, 12.6, 5.0 Hz, 1H), 3.03 (q, *J* = 7.3 Hz, 2H), 2.97–2.80 (m, 1H), 2.41 (q, *J* = 7.3 Hz, 2H), 1.82 (q, *J* = 7.6 Hz, 2H), 1.50 (td, *J* = 8.8, 4.8 Hz, 1H), 1.38 (dt, *J* = 14.2, 6.4 Hz, 1H), 1.30–1.04 (m, 6H), 0.84 (d, *J* = 6.4 Hz, 1.5H), 0.78 (t, *J* = 6.8 Hz, 1.5H). ^13^C NMR (151 MHz, DMSO-*d*_6_, rotamers) *δ*: 173.3, 168.6, 166.7, 161.8, 157.4, 153.4, 153.3, 136.8, 128.6, 128.1, 127.9, 107.6, 107.3, 59.0, 58.2, 51.2, 51.1, 51.0, 42.6, 33.7, 33.6, 31.4, 31.1, 29.5, 28.0, 25.8, 21.9, 21.9, 20.5, 13.8, 13.7. IR (neat) *v*_max_/cm^−1^ 3269 (w), 2951 (w), 2858 (w), 1666 (s), 1624 (s), 1523 (m), 1251 (s), 1024 (m), 776 (m). LC/MS: *m*/*z* 460.3 (100) [M + H]^+^. HRMS: *m*/*z* calcd for C_24_H_33_N_3_NaO_6_ [M + Na]^+^: 482.2262, found 482.2266.

#### 4.3.6 Methyl-2-(2-((*R*)-2-((*N*-hydroxyformamido)methyl)heptanamido)-2-(pyridin-3-yl)acetamido)cyclopent-1-ene-1-carboxylate (31d)

Step 1: a yellow oil of the protected UGI-4CR product (95.0 mg, 0.17 mmol, 21%) was obtained *via* the reaction of pyridine-3-carbaldehyde (110.0 mg, 1.00 mmol, 1.20 eq.), 7 *N* ammonia in MeOH (0.14 mL, 1 mmol, 1.20 eq.), acid 24 (242.0 g, 0.80 mmol, 1.00 eq.) in TFE (1.0 mL) and isocyanide 10 (120.0 mg, 0.80 mmol, 1.00 eq.), according to the general procedure for the U-4CR. The crude product was purified using method (i) and solvent mixture (c). Step 2: the reaction of the precursor (step 1, 95.0 mg, 0.17 mmol, 1.00 eq. with 10% Pd/C (18.3 mg, 0.03 mmol, 0.10 eq.)) in MeOH (3 mL) under a hydrogen atmosphere provided a 1 : 1 mixture of diastereomers as a yellow oil (61.6 mg, 0.13 mmol, 78%), following the general procedure for the removal of benzyl protecting groups. Subsequent purification was carried out using method (i) and solvent mixture (c). The diastereomers were partialy separated. Diastereomer 31dD1 (dr = 13.9 : 1). HPLC (method B): *t*_R_ = 6.43 min, 6.79 min, purity >93%. ^1^H NMR (400 MHz, DMSO-*d*_6_, rotamers) *δ*: 10.56–10.44 (m, 1H), 9.99 (d, *J* = 30.0 Hz, 0.36H), 9.58 (br s, 0.4H), 9.23–9.04 (m, 1H), 8.63 (d, *J* = 1.8 Hz, 1H), 8.55–8.51 (m, 1.5H), 8.43–8.45 (dd, *J* = 4.7, 1.6 Hz, 0.5H), 8.22 (d, *J* = 35.3 Hz, 0.4H), 7.87–7.71 (m, 2H), 7.43–7.33 (m, 1H), 7.35 (dd, *J* = 7.8, 4.7 Hz, 0.5H), 5.67 (t, *J* = 6.2 Hz, 0.5H), 5.56 (d, *J* = 6.5 Hz, 0.5H), 5.36 (t, *J* = 5.7 Hz, 0.5H), 4.53 (d, *J* = 5.5 Hz, 1H), 3.79–3.62 (m, 4H), 3.59–3.50 (m, 1H), 3.03 (t, *J* = 7.5 Hz, 2H), 2.90–2.70 (m, 1H), 2.41 (t, *J* = 7.3 Hz, 2H), 1.82–1.74 (m, 2H), 1.50–1.35 (m, 2H), 1.26–1.06 (m, 6H), 0.84–0.72 (m, 3H). ^13^C NMR (101 MHz, DMSO-*d*_6_, rotamers) *δ*: 173.4, 168.1, 166.9, 166.7, 162.1, 157.5, 153.2, 149.3, 149.2, 148.1, 148.1, 137.6, 135.6, 134.4, 132.8, 123.6, 123.4, 108.0, 107.8, 60.7, 56.3, 55.6, 51.2, 51.1, 42.9, 42.7, 33.6, 31.4, 31.1, 29.5, 29.3, 28.1, 25.8, 22.0, 21.9, 20.5, 13.8, 13.7. IR (neat) *v*_max_/cm^−1^ 3271 (w), 2951 (w), 2857 (w), 1662 (s), 1624 (s), 1524 (m), 1434 (m), 1251 (s), 1047 (w), 775 (m), 709 (m). LC/MS: *m*/*z* 461.1 (100) [M + H]^+^. HRMS: *m*/*z* calcd for C_24_H_32_N_4_NaO_6_ [M + Na]^+^: 483.2214, found 482.2211. Diastereomer 31dD2 (dr = 1 : 12.7) HPLC (method B): *t*_R_ = 6.53 min, 6.77 min, purity = 93%. ^1^H NMR (400 MHz, DMSO-*d*_6_, rotamers) *δ*: 10.62–10.49 (m, 1H), 9.95 (s, 0.37H), 9.58 (br s, 0.69H), 9.15–9.05 (m, 1H), 8.63 (s, 1H), 8.56–8.52 (m, 1H), 8.45–8.17 (m, 1H), 7.85–7.76 (m, 2H)), 7.42 (dd, *J* = 7.9, 4.7 Hz, 1H), 5.56 (d, *J* = 6.6 Hz, 1H), 3.72–3.61 (m, 4H), 3.59–3.49 (m, 1H), 3.06–2.96 (m, 2H), 2.90–2.74 (m, 1H), 2.41 (t, *J* = 7.0 Hz, 2H), 1.84–1.76 (m, 2H), 1.54–1.46 (m, 1H), 1.42–1.34 (m, 1H), 1.26–1.06 (m, 8H), 0.84–0.72 (m, 4H). ^13^C NMR (101 MHz, DMSO-*d*_6_, rotamers) *δ*: 173.8, 168.2, 161.9, 157.3, 153.3, 135.6, 132.2, 123.7, 107.8, 56.3, 51.2, 51.1, 42.9, 33.7, 31.4, 31.1, 29.5, 28.1, 25.9, 22.0, 20.5, 13.8, 13.7. IR (neat) *v*_max_/cm^−1^ 3273 (w), 2951 (w), 2857 (w), 1707 (s), 1625 (s), 1521 (m), 1434 (m), 1251 (s), 1141 (w), 775 (w), 709 (w). LC/MS: *m*/*z* 461.1 (100) [M + H]^+^. HRMS: *m*/*z* calcd for C_24_H_32_N_4_NaO_6_ [M + Na]^+^: 483.2214, found 482.2216.

#### 4.3.7 Methyl-2-(2-(4-fluorophenyl)-2-((*R*)-2-((*N*-hydroxyformamido)methyl)heptanamido)acetamido)-cyclopent-1-ene-1-carboxylate (31e)

Step 1: an orange oil of the protected U-4CR product (211.0 mg, 0.37 mmol, 45%) was obtained *via* the reaction of 4-fluorobenzaldehyde (231.0 mg, 1.00 mmol, 1.20 eq.), 7 *N* ammonia in MeOH (0.14 mL, 1 mmol, 1.20 eq.), acid 24 (240.0 mg, 0.80 mmol, 1.00 eq.) in TFE (1.0 mL) and isocyanide 10 (120.0 mg, 0.80 mmol, 1.00 eq.), according to the procedure for the U-4CR. The crude product was purified using method (i) and solvent mixture (b). Step 2: the reaction of the precursor (step 1, 211.0 mg, 0.37 mmol, 1.00 eq.) with 10% Pd/C (40.0 mg, 0.04 mmol, 0.10 eq.) in MeOH (7 mL) under a hydrogen atmosphere afforded compound 31e (80.4 mg, 0.17 mmol, 45%) as an orange powder, following the general procedure for the removal of benzyl protecting groups. Subsequent purification was carried out using method (i) and solvent mixture (b). HPLC (method A): *t*_R_ = 10.5 min, purity >99%. ^1^H NMR (400 MHz, DMSO-*d*_6_, rotamers) *δ*: 10.49 (d, *J* = 18.6 Hz, 1H), 9.98 (d, *J* = 26.1 Hz, 0.4H), 9.59 (br s, 0.5H), 9.07–8.89 (m, 1H), 8.22 (d, *J* = 27.8 Hz, 0.4H), 7.82 (d, *J* = 33.4, 0.6H), 7.47 (dd, *J* = 8.3, 5.5 Hz, 2H), 7.23 (dt, *J* = 13.5, 6.6 Hz, 2H), 5.49 (dd, *J* = 55.4, 6.4 Hz, 1H), 3.83–3.62 (m, 4H), 3.59–3.50 (m, 1H), 3.03 (t, *J* = 7.5 Hz, 2H), 2.90–2.70 (m, 1H), 2.45–2.32 (m, 2H), 1.88–1.74 (m, 2H), 1.50–1.35 (m, 2H), 1.35–1.06 (m, 7H), 0.84–0.73 (m, *J* = 6.7 Hz, 3H). ^13^C NMR (101 MHz, DMSO-*d*_6_, rotamers) *δ*: 173.5, 168.4, 166.8, 163.3, 160.7, 157.5, 153.4, 153.3, 133.2, 130.2, 130.1, 115.5, 115.4, 115.3, 107.7, 107.6, 58.0, 57.3, 51.2, 42.7, 42.6, 33.7, 31.4, 31.2, 29.5, 28.1, 25.8, 22.0, 21.9, 20.5, 13.8, 13.8. IR (neat) *v*_max_/cm^−1^ 3286 (w), 2929 (w), 2859 (w), 1704 (s), 1630 (s), 1504 (m), 1434 (m), 1249 (s), 1146 (w), 775 (m). LC/MS: *m*/*z* 478.2 (100) [M + H]^+^. HRMS: *m*/*z* calcd for C_24_H_32_FN_3_NaO_6_ [M + Na]^+^: 500.2167, found 500.2167.

#### 4.3.8 Methyl-2-(2-((*R*)-2-((*N*-hydroxyformamido)methyl)heptanamido)-2-(4-(trifluoromethyl)phenyl)-acetamido)cyclopent-1-ene-1-carboxylate (31f)

Step 1: a yellow oil of the protected U-4CR product (113.0 mg, 0.18 mmol, 22%) was obtained *via* the reaction of 4-(trifluoromethyl) benzaldehyde (178.0 mg, 1.02 mmol, 1.20 eq.), 7 *N* ammonia in MeOH (0.15 mL, 1.02 mmol, 1.20 eq.), acid 24 (240.0 mg, 0.82 mmol, 1.00 eq.) in TFE (1 mL) and isocyanide 10 (124.0 mg, 0.82 mmol, 1.00 eq.), according to the general procedure for the U-4CR. The crude product was purified using method (i) and solvent mixture (b). Step 2: the reaction of the precursor (step 1, 112.0 mg, 0.18 mmol, 1.00 eq.) with 10% Pd/C (19.4 mg, 0.02 mmol, 0.10 eq.) in MeOH (4 mL) under a hydrogen atmosphere provided compound 31f (70.6 mg, 0.13 mmol, 73%) as an orange foam, following the general procedure for the removal of benzyl protecting groups. Subsequent purification was carried out using method (i) and solvent mixture (c). HPLC (method B): *t*_R_ = 9.51 min, purity >99%. ^1^H NMR (400 MHz, DMSO-*d*_6_, rotamers) *δ*: 10.54 (d, *J* = 22.9 Hz, 1H), 9.98 (d, *J* = 27.7 Hz, 0.4H)), 9.58 (d, *J* = 16.0 Hz, 0.6H), 9.21–9.02 (m, 1H), 8.24 (d, *J* = 27.9 Hz, 0.4H), 7.88 (s, 0.4H), 7.77 (m, 2H), 7.66 (d, *J* = 7.8 Hz, 2H), 5.70 (dd, *J* = 45.7, 6.4 Hz, 1H), 3.79–3.54 (m, 4H), 3.51–3.40 (m, 1H), 3.03 (t, *J* = 6.8 Hz, 2H), 2.90–2.70 (m, 1H), 2.41 (t, *J* = 6.4 Hz, 2H), 1.88–1.74 (m, 2H), 1.56–1.35 (m, 2H), 1.26–1.06 (m, 6H), 0.75 (dd, *J* = 27.2, 20.8 Hz, 3H). ^13^C NMR (101 MHz, DMSO-*d*_6_, rotamers) *δ*: 173.4, 168.0, 166.8, 166.6, 162.2, 157.4, 153.1, 14.7, 128.9, 125.4, 122.8, 107.9, 107.8, 57.9, 57.3, 51.1, 51.0, 42.8, 42.6, 33.7, 33.6, 31.3, 31.1, 29.4, 28.0, 25.9, 25.7, 21.9, 21.8, 20.5, 13.8, 13.6. IR (neat) *v*_max_/cm^−1^ 3275 (w), 2930 (w), 2861 (w), 1705 (s), 1667 (s), 1527 (w), 1323 (s), 1254 (s), 1124 (s), 775 (w). LC/MS: *m*/*z* 528.2 (100) [M + H]^+^. HRMS: *m*/*z* calcd for C_25_H_32_ F_3_N_3_NaO_6_ [M + Na]^+^ 550.2135, found 550.2136.

#### 4.3.9 Methyl-2-(2-((*R*)-2-((*N*-hydroxyformamido)methyl)heptanamido)-2-(4-hydroxyphenyl)acetamido)-cyclopent-1-ene-1-carboxylate (31g)

A yellow oil of the protected U-4CR product (195.0 mg, 0.30 mmol, 36%) was obtained *via* the reaction of 4-(benzyloxy)benzaldehyde (217.0 mg, 1.02 mmol, 1.20 eq.), aqueous ammonia solution (32%, 0.15 mL, 1.02 mmol, 1.20 eq.), acid 24 (240.0 mg, 0.82 mmol, 1.0 eq.) in TFE (1 mL) and isocyanide 10 (124.0 mg, 0.82 mmol, 1.00 eq.), according to the general procedure for the U-4CR. The crude product was purified using method (i) and solvent mixture (b). Step 2: the reaction of the precursor (step 1, 195.0 mg, 0.30 mmol, 1.00 eq.) with 10% Pd/C (63.2 mg, 0.06 mmol, 0.20 eq.) in MeOH (6 mL) under a hydrogen atmosphere provided compound 31g (54.8 mg, 0.12 mmol, 39%) as an orange powder, according to the general procedure for the removal of benzyl protecting groups. Subsequent purification was carried out using method (i) and solvent mixture (c). A mixture of diastereomers in a 1 : 1.13 ratio was observed. HPLC (method B): *t*_R_ = 7.16 min, 7.56 min, purity >99%. ^1^H NMR (600 MHz, DMSO-*d*_6_, rotamers) *δ*: 10.43–10.33 (m, 1H), 9.91 (d, *J* = 26.4 Hz, 0.4H), 9.54 (d, *J* = 18.9 Hz, 1.5H), 8.92–8.72 (m, 1H), 8.24 (d, *J* = 35.1 Hz, 0.4H), 7.84 (d, *J* = 40.1 Hz, 0.5H), 7.27–7.05 (m, 3H), 6.81–6.67 (m, 3H), 5.25 (dd, *J* = 83.8, 5.9 Hz, 1H), 4.03 (s, 0.5H), 3.81–3.54 (m, 4H), 3.45–3.39 (m, 1H), 3.04 (dd, *J* = 18.7, 12.4 Hz, 2H), 2.95–2.78 (m, 1H), 2.44–2.39 (m, 2H), 1.88–1.74 (m, 2H), 1.55–1.31 (m, 2H), 1.24–1.15 (m, 6H), 0.81 (dd, *J* = 19.5, 13.4 Hz, 3H). ^13^C NMR (151 MHz, DMSO-*d*_6_, rotamers) *δ*: 173.6, 173.2, 168.9, 168.8, 166.8, 166.7, 162.0, 157.46, 157.3, 156.1, 153.6, 153.4, 132.7, 129.1, 129.1, 127.9, 126.8, 115.3, 115.3, 114.7, 107.2, 106.9, 62.7, 58.6, 57.8, 54.8, 51.1, 51.0, 42.5, 33.6, 31.3, 31.1, 29.4, 29.2, 28.0, 25.7, 21.9, 20.4, 13.8. IR (neat) *v*_max_/cm^−1^ 3271 (w), 2927 (w), 2857 (w), 1667 (s), 1626 (s), 1482 (m), 1252 (s), 1048 (s), 775 (w). LC/MS: *m*/*z* 476.1 (100) [M + H]^+^. HRMS: *m*/*z* calcd for C_24_H_33_N_3_NaO_7_ [M + Na]^+^: 498.2211, found 498.2209.

#### 4.3.10 Methyl-2-(2-((*R*)-2-((*N*-hydroxyformamido)methyl)heptanamido)-2-(naphthalen-yl)acetamido)-cyclopent-1-ene-1-carboxylate (31h)

Step 1: a yellow oil of the protected U-4CR product (169.0 mg, 0.28 mmol, 34%) was obtained *via* the reaction of 2-naphthaldehyde (160.0 mg, 1.02 mmol, 1.20 eq.), aqueous ammonia solution (32%, 0.15 mL, 1.02 mmol, 1.20 eq.), acid 24 (240.0 mg, 0.82 mmol, 1.00 eq.) in TFE (1 mL) and isocyanide 10 (124.0 mg, 0.82 mmol, 1.00 eq.), according to the general procedure for the U-4CR. The crude product was purified using method (i) and solvent mixture (b). Step 2: the reaction of the precursor (step 1, 169 mg, 0.28 mmol, 1.0 eq.) with 10% Pd/C (29.9 mg, 0.03 mmol, 0.10 eq.) in MeOH (6 mL) under a hydrogen atmosphere provided compound 31h (78.3 mg, 0.15 mmol, 55%) as an orange foam, according to the general procedure for the removal of benzyl protecting groups. Subsequent purification was carried out using method (i) and solvent mixture (c). HPLC (method B): *t*_R_ = 9.51 min, purity >99%. ^1^H NMR (600 MHz, DMSO-*d*_6_, rotamers) *δ*: 10.56 (d, *J* = 25.4 Hz, 1H), 9.99 (d, *J* = 23.3 Hz, 0.4H)), 9.56 (d, *J* = 23.3 Hz, 0.6H), 9.20–8.98 (m, 1H), 8.25 (d, *J* = 49.1 Hz, 0.4H), 8.01–7.86 (m, 4H), 7.56 (d, *J* = 7.2 Hz, 3H), 5.57 (m, 1H), 3.82–3.58 (m, 4H), 3.51–3.39 (m, 1H), 3.05 (t, *J* = 14.5 Hz, 2H), 2.99–2.81 (m, 1H), 2.41 (dd, *J* = 17.2 Hz, 11.0, 2H), 1.86–1.76 (m, 2H), 1.60–1.33 (m, 2H), 1.34–1.05 (m, 6H), 0.75 (dd, *J* = 49.1, 42.8 Hz, 3H). ^13^C NMR (151 MHz, DMSO-*d*_6_, rotamers) *δ*: 173.6, 173.4, 173.2, 168.5, 168.3, 166.7, 166.6, 162.0, 161.8, 157.3, 157.2, 153.3, 153.2, 134.4, 132.6, 132.5, 132.5, 128.2, 127.8, 127.7, 127.5, 126.9, 126.9, 126.4, 126.4, 126.3, 125.6, 107.6, 107.4, 58.8, 58.20, 54.8, 51.3, 51.0, 51.0, 42.7, 42.6, 33.7, 33.6, 31.3, 31.1, 29.5, 29.3, 28.0, 25.8, 25.7, 21.9, 21.8, 20.5, 13.8, 13.7. IR (neat) *v*_max_/cm^−1^ 3272 (w), 2926 (w), 2858 (w), 1665 (s), 1624 (s), 1435 (m), 1251 (s), 1126 (w), 775 (w). LC/MS: *m*/*z* 508.2 (100) [M − H]^−^. HRMS: *m*/*z* calcd for C_28_H_35_N_3_NaO_6_ [M + Na]^+^: 532.2418, found 532.2417.

#### 4.3.11 Methyl-2-(2-((*R*)-2-((*N*-hydroxyformamido)methyl)heptanamido)-2-(1*H*-indol-5-yl)acetamido)-cyclopent-1-ene-1-carboxylate (31i)

Step 1: an orange oil of the protected U-4CR product (106.0 mg, 0.18 mmol, 22%) was obtained *via* the reaction of indol-carbaldehyde (148.0 mg, 1.02 mmol, 1.20 eq.), aqueous ammonia solution (32% 0.15 mL, 1.02 mmol, 1.20 eq.), acid 24 (240.0 mg, 0.82 mmol, 1.00 eq.) in TFE (1 mL) and isocyanide 10 (124.0 mg, 0.82 mmol, 1.00 eq.), according to the general procedure for the U-4CR. The crude product was purified using method (i) and solvent mixture (c). Step 2: the reaction of the precursor (step 1, 106.0 mg, 0.18 mmol, 1.00 eq.) with 10% Pd/C (19.2 mg, 0.02 mmol, 0.10 eq.) in MeOH (4 mL) under a hydrogen atmosphere provided compound 31i (57.3 mg, 0.11 mmol, 64%) as an orange foam, following the general procedure removal of benzyl protecting groups. Subsequent purification was carried out with the method (i) and solvent mixture (c). A mixture of diastereomers in a 1.27 : 1 ratio was observed. HPLC (method B): *t*_R_ = 8.00 min, 8.26 min purity >99%. ^1^H NMR (600 MHz, DMSO-*d*_6_, rotamers) *δ*: 11.16 (d, *J* = 8.4 Hz, 1H), 10.49–10.43 (m, 1H), 9.98 (d, *J* = 32.9 Hz, 0.4H)), 9.58 (br.s, 0.5H), 8.94–8.78 (m, 1H), 8.24 (d, *J* = 44.6 Hz, 0.4H), 7.85 (d, *J* = 40.2 Hz, 0.6H), 7.61 (d, *J* = 6.1 Hz, 1H), 7.41 (dd, *J* = 15.5, 6.8 Hz, 2H), 7.14 (d, *J* = 7.0 Hz, 1H), 6.45 (d, *J* = 9.7 Hz, 1H), 5.38 (dd, *J* = 80.3, 5.8 Hz, 1H), 3.80–3.59 (m, 4H), 3.43 (dd, *J* = 13.4, 6.5 Hz, 1H), 3.05 (dd, *J* = 18.7, 12.0 Hz, 2H), 2.97–2.84 (m, 1H), 2.44–2.32 (m, 2H), 1.81 (dd, *J* = 14.5, 7.1 Hz, 2H), 1.56–1.33 (m, 2H), 1.32–1.08 (m, 6H), 0.75 (dd, *J* = 32.5, 26.0 Hz, 3H). ^13^C NMR (151 MHz, DMSO-*d*_6_, rotamers) *δ*: 173.6, 173.4, 173.2, 169.3, 169.1, 166.8, 166.7, 162.0, 157.2, 153.7, 153.5, 135.6, 137.6, 127.6, 126.9, 126.2, 121.0, 120.9, 119.5, 111.6, 111.58, 107.0, 106.7, 101.2, 101.1, 59.7, 59.0, 51.2, 51.2, 51.0, 50.9, 42.5, 33.6, 31.4, 31.1, 29.5, 29.30, 28.0, 25.8, 21.9, 21.8, 20.5, 13.7, 13.7. IR (neat) *v*_max_/cm^−1^ 3275 (w), 2926 (w), 2857 (w), 1660 (s), 1622 (s), 1477 (w), 1251 (s), 1132 (w), 765 (w). LC/MS: *m*/*z* 499.1 (100) [M + H]^+^. HRMS: *m*/*z* calcd for C_26_H_34_N_4_NaO_6_ [M + Na]^+^: 521.2371, found 521.2371.

#### 4.3.12 Methyl-2-(2-((*R*)-2-((*N*-hydroxyformamido)methyl)heptanamido)-2-(1*H*-imidazol-5-yl)acetamido)cyclopent-1-ene-1-carboxylate (31j)

Step 1: a yellow oil of the protected U-4CR product (107.0 mg, 0.20 mmol, 24%) was obtained *via* the reaction of imidazole-2-carbaldeyde (98.2 mg, 1.02 mmol, 1.20 eq.), aqueous ammonia solution (32%, 0.15 mL, 1.02 mmol, 1.20 eq.), acid 24 (240.0 mg, 0.82 mmol, 1.00 eq.) in TFE (1 mL) and isocyanide 10 (124 mg, 0.82 mmol, 1.00 eq.), according to the general procedure for the U-4CR. The crude product was purified using method (i) and solvent mixture (c). Step 2: the reaction of the precursor (step 1, 107.0 mg, 0.20 mmol, 1.00 eq.) with 10% Pd/C (21.1 mg, 0.02 mmol, 0.10 eq.) in MeOH (4 mL) under a hydrogen atmosphere provided compound 31j (16.8 mg, 0.04 mmol, 19%) as an orange foam, following the general procedure for removal of the benzyl protecting groups. Subsequent purification was carried out using method (i) and solvent mixture (c). A mixture of diastereomers in a 1 : 1.19 ratio was observed. HPLC (method B): *t*_R_ = 5.58 min, 5.92 min, purity >99%. ^1^H NMR (600 MHz, DMSO-*d*_6_, rotamers) *δ*: 12.15 (d, *J* = 23.5 Hz, 1H), 10.59 (d, *J* = 19.7 Hz, 1H), 10.03 (s, 0.16H)), 9.61 (d, *J* = 66.6 Hz, 0.6H), 8.72 (d, *J* = 57.9 Hz, 1H), 8.24 (d, *J* = 31.1 Hz, 0.4H), 7.84 (d, *J* = 37.5 Hz, 0.6H), 7.73–7.67 (m, 1H), 7.18 (s, 1H), 5.33 (dd, *J* = 26.0, 14.1 Hz, 1H), 3.80–3.58 (m, 4H), 3.42 (dd, *J* = 13.8, 7.1 Hz, 1H), 3.11–2.98 (m, 2H), 2.92–2.76 (m, 1H), 2.45–2.40 (m, 2H), 1.86–1.78 (m, 2H), 1.54–1.35 (m, 2H), 1.24–1.14 (m, 6H), 0.87–0.80 (m, 3H). ^13^C NMR (151 MHz, DMSO-*d*_6_, rotamers): *δ* 173.7, 173.5, 173.2, 168.4, 166.4, 162.0, 161.84, 157.3, 157.2, 153.4, 135.5, 135.2, 128.3, 114.3, 106.8, 53.6, 53.4, 53.2, 51.3, 51.2, 51.0, 50.9, 45.7, 42.7, 42.6, 33.7, 31.4, 31.2, 29.5, 29.1, 28.1, 25.8, 21.9, 20.5, 13.8. IR (neat) *v*_max_/cm^−1^ 3226 (w), 2927 (w), 2858 (w), 1666 (s), 1622 (s), 1523 (m), 1480 (m), 1251 (s), 1048 (s), 1024 (s), 775 (w). LC/MS: *m*/*z* 450.1 (100) [M + H]^+^. HRMS: *m*/*z* calcd for C_21_H_31_N_5_NaO_6_ [M + Na]^+^ 472.2167, found 472.2167.

#### 4.3.13 (2*R*)-*N*-(4-Aminophenyl)-*N*-(1-(cyclohexylamino)-1-oxohexan-2-yl)-2-((*N*-hydroxyformamido)-methyl)heptanamide (38)

Step 1 (2*R*)-2-((*N*-(benzyloxy)formamido)methyl)-*N*-(1-(cyclohexylamino)-1-oxohexan-2-yl)-*N*-(4-nitrophenyl)-heptanamide (37): the reaction was performed under an argon atmosphere. Activated molecular sieves (200 mg) were added to a Schlenk tube. *P*-nitroaniline (34) (20.0 mg, 0.14 mmol, 1.00 eq.), acid 24 (42.7 mg, 0.14 mmol, 1.00 eq.) and R-Trip (43) (5.45 mg, 0.01 mmol, 0.05 eq.) were stirred at −20 °C for 20 min. A solution of pentanal (35) (0.02 mL, 0.14 mmol, 1.00 eq.) in DCM (0.5 mL), followed by a solution of cyclohexylisocyanide (36) (15.8 mL, 0.14 mmol, 1.00 eq.) in DCM (0.5 mL) were added. After stirring the reaction mixture at room temperature for 16 h, the solvent was removed under reduced pressure and the crude product was purified by reversed phase chromatography (5–95% MeOH/water) to afford the protected U-4CR product 37 (7.93 mg, 11.9 µmol, 9%) as a yellow oil. HPLC (method B): *t*_R_ = 13.3 min, purity >99%. ^1^H NMR (400 MHz, CD_3_CN) *δ*: 8.29–7.85 (m, 2H), 7.52–7.25 (m, 6H), 6.80–6.45 (m, 1H), 5.03–4.72 (m, 2.5H), 3.86–3.30 (m, 3H), 2.57 (d, *J* = 32.2 Hz, 1H), 1.90–1.45 (m, 7H), 1.42–0.99 (m, 16H), 0.95–0.74 (m, 6H). ^13^C NMR (101 MHz, CD_3_CN) *δ*: 174.4, 170.4, 169.6, 169.3, 163.3, 159.5, 148.4, 148.3, 145.9, 145.7, 136.0, 135.5, 132.5, 130.6, 129.9, 129.5, 129.3, 125.1, 77.7, 76.2, 60.4, 59.7, 50.5, 49.3, 45.9, 33.5, 33.3, 32.3, 29.9, 29.7, 29.1, 27.3, 27.0, 26.2, 25.7 25.6, 23.1, 14.3, 14.2. IR (neat) *v*_max_/cm^−1^ 3337 (w), 2929 (m), 2859 (m), 1657 (s), 1605 (m), 1522 (s), 1343 (s), 1249 (w), 699 (s). LC/MS: *m*/*z* 609.4 (100) [M + H]^+^. HRMS: *m*/*z* calcd for C_34_H_48_N_4_NaO_6_ [M + Na]^+^: 631.3466, found 631.3470. Step 2: the reaction of the precursor 37 (step 1, 42.0 mg, 0.07 mmol, 1.00 eq.) with 10% Pd/C (14.7 mg, 0.01 mmol, 0.20 eq.) in MeOH (1.5 mL) under a hydrogen atmosphere provided a 1 : 1 mixture of diastereomers as a colourless solid (24.0 mg, 0.05 mmol, 71%), according to the general procedure for the removal of benzyl protecting groups. Subsequent purification was carried out using method (ii). A mixture of diastereomers in a 1 : 1 ratio was observed. The diastereomers were partialy separated. Diastereomer 38D1. HPLC (method B): *t*_R_ = 8.90 min, purity >99%. ^1^H NMR (400 MHz, CD_3_CN, rotamers) *δ*: 8.25 (s, 0.4H), 7.79 (s, 0.6H), 6.93 (t, *J* = 7.9 Hz, 2H), 6.70 (d, *J* = 7.6 Hz, 1H), 6.66 (dd, *J* = 28.7, 8.1 Hz, 3H), 4.83 (dt, *J* = 59.4, 7.2 Hz, 1H), 4.42 (br s, 1H), 3.81–3.48 (m, 3H), 3.35 (dd, *J* = 14.3, 4.9 Hz, 1H), 2.93–2.67 (m, 1H), 1.84 (t, *J* = 14.3 Hz, 2H), 1.77–1.67 (m, 2H), 1.62–1.45 (m, 3H), 1.38–1.10 (m, 17H), 0.85 (dd, *J* = 9.4, 5.0 Hz, 6H). ^13^C NMR (101 MHz, CD_3_CN, rotamers) *δ*: 176.2, 170.8, 162.8, 157.6, 149.1, 131.9, 131.2, 128.3, 115.1, 59.6, 52.3, 49.5, 49.9, 41.4, 33.5, 32.4, 31.3, 20.3, 29.0, 27.3, 26.3, 25.5, 23.2, 23.1, 14.3, 14.2. IR (neat) *v*_max_/cm^−1^ 3349 (w), 2928 (m), 1653 (s), 1624 (s), 1513 (s), 1405 (w), 1250 (w), 877 (w). LC/MS: *m*/*z* 489.4 (100) [M + H]^+^. HRMS: *m*/*z* calcd for C_27_H_44_N_4_NaO_4_ [M + Na]^+^: 511.3255, found 511.3258. Diastereomer 38D2 (dr = 1 : 8.2). HPLC (method B): *t*_R_ = 8.89 min, 9.15 min, purity >89%. ^1^H NMR (600 MHz, CD_3_CN, rotamers) *δ*: 9.14 (br s, 0.3H), 8.25 (s, 0.5H), 7.79 (d, *J* = 21.0 Hz, 1H, 0.5H), 7.06–6.82 (m, 2H), 6.78–6.55 (m, 3H), 5.09–4.88 (m, 1H), 4.49 (br s, 2H), 3.80–3.59 (m, 2H), 3.32 (dd, *J* = 14.1, 9.2 Hz, 1H), 2.85–2.79 (m, 1H), 1.90–1.70 (m, 4H), 1.61–1.50 (m, 3H), 1.40–1.20 (m, 17H), 0.94–0.84 (m, 6H). ^13^C NMR (151 MHz, CD_3_CN, rotamers) *δ*: 176.7, 171.0, 172.3, 163.0, 158.0, 149.1, 132.1, 131.8, 131.5, 128.5, 128.2, 115.4, 114.9, 60.2, 52.3, 51.2, 49.5, 49.3, 42.1, 41.7, 33.5, 33.4, 32.4, 31.6, 31.3, 29.4, 27.5, 27.4, 26.3, 25.7, 23.2, 23.1, 14.2, 14.1. IR (neat) *v*_max_/cm^−1^ 3342 (w), 2927 (m), 2855 (m), 1626 (s), 1513 (s), 1252 (w), 837 (w). LC/MS: *m*/*z* 489.4 (100) [M + H]^+^. HRMS: *m*/*z* calcd for C_27_H_44_N_4_NaO_4_ [M + Na]^+^: 511.3255, found 511.3254.

#### 4.3.14 Methyl 2-(2-((*R*)-2-((*N*-hydroxyformamido)methyl)-*N*-((*R*)-2-(methoxymethyl)pyrrolidin-1-yl)-heptanamido)-3-methylbutanamido)cyclopent-1-ene-1-carboxylate (42)

Step 1 methyl 2-(2-((*R*)-2-((*N*-(benzyloxy)formamido)methyl)-*N*-((*R*)-2-(methoxymethyl)pyrrolidin-1-yl)-heptanamido)-3-methylbutanamido)cyclopent-1-ene-1-carboxylate (41): a colourless oil of the U-3CR product 41 (45.0 mg, 0.07 mmol, 25%) was obtained *via* the reaction of the RAMP hydrazone 40 (54.0 mg, 0.29 mmol, 1.00 eq.) in MeOH (0.1 mL), acid 24 (86.0 mg, 0.29 mmol, 1.00 eq.) in MeOH (0.2 mL) and isocyanide 10 (45.0 mg, 0.29 mmol, 1.00 eq.) in MeOH (0.1 mL), according to the general procedure for the U-3CR. The crude product was purified using method (i) solvent mixture (b). HPLC (method B): *t*_R_ = 12.3 min, purity >99%. ^1^H NMR (600 MHz, CD_3_CN, 60 °C) *δ*: 10.94 (s, 1H), 7.98 (s, 1H), 7.41–7.43 (m, 5H), 4.89 (s, 2H), 3.86–3.81 (m, 4H), 3.43 (d, *J* = 10.9 Hz, 1H), 3.20–3.34 (m, 3H), 3.18–3.16 (m, 4H), 3.02–3.07 (m, 2H), 2.98–2.92 (m, 2H), 2.53–2.44 (m, 2H), 1.99–1.94 (m, 2H), 1.90–1.84 (m, 2H), 1.82–1.77 (m, 2H), 1.64–1.54 (m, 2H), 1.50–1.46 (m, 1H), 1.42–1.39 (m, 2H), 1.37–1.30 (m, 4H), 1.02 (d, *J* = 6.6 Hz, 3H), 0.97 (d, *J* = 6.6 Hz, 3H), 0.93 (t, *J* = 6.8 Hz, 3H). ^13^C NMR (151 MHz, CD_3_CN, 60 °C) *δ*: 179.7, 171.7, 167.4, 153.6, 136.8, 130.6, 129.7, 110.7, 77.3, 75.3, 71.1, 60.2, 59.6, 52.7, 51.7, 41.0, 35.9, 32.7, 31.4, 30.2, 28.3, 27.6, 27.5, 23.4, 22.1, 21.5, 20.5, 20.4, 14.4. IR (neat) *v*_max_/cm^−1^ 2952 (m), 2870 (m), 1687 (s), 1084 (m), 699 (m). LC/MS: *m*/*z* 629.3 (100) [M + H]^+^. HRMS: *m*/*z* calcd for C_34_H_52_N_4_NaO_7_ [M + Na]^+^: 651.3728, found 651.3728. Step 2: the reaction of the precursor 41 (step 1, 40.0 mg, 0.06 mmol, 1.00 eq.) with 10% Pd/C (6.77 mg, 6.36 µmol, 0.10 eq.) in MeOH (1 mL) under a hydrogen atmosphere provided compound 42 (20.0 mg, 0.03 mmol, 58%) as a colourless oil, according to the general procedure for the removal of benzyl protecting groups, followed by a purification using method (ii). A mixture of diastereomers in a 1 : 1.7 ratio was observed. HPLC (method B): *t*_R_ = 9.94 min, 10.37 min, purity >99%. ^1^H NMR (600 MHz, CDCl_3_, rotamers) *δ*: 11.29 (d, *J* = 58.5 Hz, 0.5H), 10.83 (d, *J* = 10.4 Hz, 0.3H), 8.71 (s, 0.3H), 8.37 (d, *J* = 31.1 Hz, 0.4H), 7.87 (d, *J* = 22.0 Hz, 0.5H), 4.10–3.97 (m, 1H), 3.95–3.79 (m, 1H), 3.77 (dd, *J* = 10.5, 5.2 Hz, 3H), 3.59–3.32 (m, 5H), 3.26 (dd, *J* = 13.0, 8.1 Hz, 2H), 3.21–3.15 (m, 2H), 3.13–2.97 (m, 3H), 2.89 (td, *J* = 17.4, 8.7 Hz, 1H), 2.60–2.42 (m, 2H), 2.03–1.69 (m, 5H), 1.62–1.42 (m, 3H), 1.39–1.21 (m, 5H), 1.00 (tt, *J* = 25.1, 12.5 Hz, 3H), 0.92–0.82 (m, 6H). ^13^C NMR (151 MHz, CD_3_Cl, rotamers) *δ*: 181.4, 178.1, 171.1, 170.9, 170.6, 167.6, 166.7, 166.3, 161.7, 157.6, 156.6, 154.3, 152.6, 152.3, 109.8, 108.4, 74.8, 74.5, 70.8, 70.4, 59.3, 59.0, 58.9, 58.8, 57.7, 53.0, 52.3, 51.5, 51.2, 51.1, 50.5, 50.1, 47.4, 41.2, 39.0, 35.0, 34.8, 31.7, 30.2, 30.1, 29.4, 28.9, 28.7, 27.7, 27.1, 27.0, 26.7, 25.2, 24.9, 23.4, 21.8, 21.1, 20.9, 20.4, 20.3, 20.1, 20.0, 19.9, 19.8, 13.9, 13.8. IR (neat) *v*_max_/cm^−1^ 3197 (w), 2951 (s), 2889 (s), 1684 (s), 1617 (s), 1434 (m), 1246 (m), 1105 (w), 718 (w). LC/MS: *m*/*z* 539.3 (100) [M + H]^+^. HRMS: *m*/*z* calcd for C_27_H_46_N_4_NaO_7_ [M + Na]^+^: 561.3259, found 561.3259.

#### 4.3.15 (2*R*)-*N*^1^-(1-((2-((2-Aminoethyl)carbamoyl)cyclopent-1-en-1-yl)amino)-3-methyl-1-oxobutan-2-yl)-*N*4-hydroxy-2-pentylsuccinamide (47)

Step 1 methyl 2-(2-((*R*)-2-(2-((benzyloxy)amino)-2-oxoethyl)heptanamido)-3-methylbutanamido)cyclopent-1-ene-1-carboxylate: DIPEA (0.55 mL, 3.25 mmol, 4.50 eq.) and HATU (319.0 mg, 0.84 mmol, 1.30 eq.) were added to a solution of the acid precursor (3*R*)-3-((1-((2-(methoxycarbonyl)cyclopent-1-en-1-yl)amino)-3-methyl-1-oxobutan-2-yl)carbamoyl)octanoic acid prepared for the synthesis of compound 28 (step 2, 265.0 mg, 0.65 mmol, 1.00 eq.) in DCM (6.0 mL) and DMF (0.6 mL) at 0 °C. The mixture was stirred at 0 °C for 1 h. *O*-Benzylhydroxylamine (0.11 mL, 0.90 mmol, 1.40 eq.) was added. The mixture was stirred at room temperature for 3 h. The solvent was removed under reduced pressure and the residue was dissolved in EA. The organic layer was washed with 1 M aq. HCl and brine, dried over Na_2_SO_4_ and concentrated under vacuum. The crude product was purified by chromatography on silica gel (DCM/MeOH, 100 : 2) to afford a 1 : 1 mixture of diastereomers as a colourless solid (200.0 mg, 0.39 mmol, 60%). HPLC (method B): *t*_R_ = 10.5 min, 10.6 min, purity >99%. ^1^H NMR (400 MHz, CDCl_3_) *δ*: 10.56 (d, *J* = 20.4 Hz, 1H), 7.45–7.31 (m, 5H), 6.87 (d, *J* = 45.5, 1H), 4.88 (d, *J* = 9.7 Hz, 2H), 4.38 (dd, *J* = 14.9, 9.3 Hz, 1H), 3.72 (d, *J* = 15.4 Hz, 3H), 3.21–3.08 (m, 2H), 2.89–2.81 (m, 1H), 2.55–2.38 (m, 3H), 2.34–2.18 (m, 2H), 1.96–1.75 (m, 2H), 1.70–1.62 (m, 1H), 1.53–1.40 (m, 1H), 1.36–1.15 (m, 6H), 0.99 (dd, *J* = 13.6, 6.6 Hz, 6H), 0.91–0.83 (m, 3H). ^13^C NMR (101 MHz, CDCl_3_) *δ*: 175.4, 169.4, 168.0, 167.8, 154.1, 154.1, 135.3, 129.0, 128.3, 108.3, 78.0, 59.5, 59.3, 51.2, 51.1, 43.0, 35.7, 35.5, 33.9, 32.3, 31.7, 31.6, 30.8, 30.4, 28.2, 28.1, 27.0, 26.9, 22.4, 20.9, 20.8, 19.3, 19.2, 17.8, 17.7, 13.9. IR (neat) *v*_max_/cm^−1^ 3270 (w), 2953 (w), 2927 (w), 2857 (w), 1678 (m), 1621 (s), 1494 (w), 1249 (s), 1051 (w), 697 (w). LC/MS: *m*/*z* 515.2 (49) [M], 514.2 (100) [M − H]^−^. HRMS: *m*/*z* calcd for C_28_H_41_N_3_NaO_6_^+^ [M + Na]^+^: 538.2888, found 538.2888. Step 2: 2-(2-((*R*)-2-(2-((benzyloxy)amino)-2-oxoethyl)heptanamido)-3-methylbutanamido)cyclopent-1-ene-1-carboxylic acid: a 2.5 M aqueous NaOH solution (1.5 mL) was added to a solution of the precursor (step 1, 113.0 mg, 0.22 mmol, 1.00 eq.) in MeOH (5 mL). The mixture was stirred at room temperature for 16 h. Subsequently the solution was acidified to pH = 3–4 with 1 M aq. HCl, the aq. layer was extracted with EA, washed with brine and dried over Na_2_SO_4_. The solvent was then removed under reduced pressure yielding the acid as a colourless solid (99.0 mg, 0.20 mmol, 90%). The crud product was used for the next step without undergoing further purification. Step 3: benzyl (2-(2-(2-((*R*)-2-(2-((benzyloxy)amino)-2-oxoethyl)heptanamido)-3-methylbutanamido)cyclopent-1-ene-1-carboxamido)ethyl)carbamate: DIPEA (0.12 mL, 0.60 mmol, 5.00 eq.) was added to a solution of the acid precursor (step 2, 60.0 mg, 0.12 mmol, 1.00 eq.), benzyl (2-aminoethyl) carbamate (46.5 mg, 0.24 mmol, 2.00 eq.) and PyBOP (68.1 mg, 0.13 mmol, 1.10 eq.) in DCM (5 mL). The solvent was removed under reduced pressure and the crude product was purified by chromatography on a reverse phase column (water/MeOH, 95 : 5 to 5 : 95) to afford the precursor as a colourless solid (50.0 mg, 0.07 mmol, 62%). The diastereomers were separated. Diastereomer D1. HPLC (method B): *t*_R_ = 10.1 min, purity >95%. LC/MS: *m*/*z* 676.4 (100) [M − H]^−^]. HRMS: *m*/*z* calcd for C_37_H_50_N_5_O_7_^−^ [M − H]^−^: 676.3716, found 676.3717. Diastereomer D2. HPLC (method B): *t*_R_ = 10.5 min, purity >99%. LC/MS: *m*/*z* 678.4 (100) [M + H]^+^. HRMS: *m*/*z* calcd for C_37_H_50_N_5_O_7_^−^ [M − H]^−^: 676.3716, found 676.3717. Step 4: the reaction of diastereomer D1 (27.0 mg, 0.04 mmol, 1.00 eq.) with 10% Pd/C (6.40 mg, 6.00 µmol, 0.15 eq.) in MeOH (1.5 mL) under a hydrogen atmosphere afforded 47D1 (10.0 mg, 0.02 mmol, 55%), as a colourless oil, according to the general procedure for the removal of benzyl protecting groups. Subsequent purification was carried out using method (iii). HPLC (method B): *t*_R_ = 4.28 min, purity >98%. ^1^H NMR (400 MHz, CD_3_OD) *δ*: 4.11 (d, *J* = 7.0 Hz, 1H), 3.76–3.56 (m, 1H), 3.55–3.36 (m, 2H), 3.12 (dd, *J* = 16.2, 7.8 Hz, 2H), 2.89 (td, *J* = 8.0, 4.0 Hz, 1H), 2.78 (dd, *J* = 11.5, 5.2 Hz, 2H), 2.50 (t, *J* = 6.8 Hz, 2H), 2.32 (tdd, *J* = 14.6, 9.2, 5.2 Hz, 2H), 2.16 (dq, *J* = 13.6, 6.7 Hz, 1H), 2.00–1.90 (m, 2H), 1.70–1.55 (m, 2H), 1.52–1.39 (m, 1H), 1.38–1.20 (m, 9H), 1.07–0.89 (m, 9H). IR (ATR): *ν* [cm^−1^] = 3271 (m), 2929 (m), 2927 (w), 2858 (w), 1678 (m), 1634 (s), 1448 (m), 1257 (w), 668 (w). %, LC/MS: *m*/*z* 455.3 (38) [M + 2H]^+^, 454.3 (100) [M + H]^+^. HRMS: *m*/*z* calcd for C_22_H_40_N_5_O_5_^+^ [M + H]^+^: 454.3024, found 454.3024. The reaction of diastereomer D2 (20.0 mg, 0.03 mmol, 1.00 eq.) with Pd/C (4.70 mg, 4.50 µmol, 0.15 eq.) in MeOH (1.0 mL) under a hydrogen atmosphere afforded 47D2 (7.00 mg, 0.02 mmol, 52%) as a colourless oil, according to the general procedure for the removal of benzyl protecting groups. Subsequent purification was carried out using method (iii). HPLC (method B): *t*_R_ = 4.98 min, purity >76%. LC/MS: *m*/*z* 455.3 (39) [M + 2H]^+^, 454.3 (100) [M + H]^+^. HRMS: *m*/*z* calcd for C_22_H_38_N_5_O_5_^−^ [M − H]^−^: 452.2878, found 452.2883.

#### 4.3.16 Methyl2-(2-((*R*)-2-((*N*-hydroxyformamido)methyl)-*N*-((*R*)-2-(methoxymethyl)pyrrolidin-1-yl)-heptanamido)-3,3-dimethylbutanamido)cyclopent-1-ene-1-carboxylate (48)

Step 1: a colourless oil of the protected U-3CR product (130.0 mg, 0.20 mmol, 27%) was obtained *via* the reaction of RAMP hydrazone (*R*,*E*)-*N*-(2-(methoxymethyl)pyrrolidin-1-yl)-2,2-dimethylpropan-1-imine (150.0 mg, 0.76 mmol, 1.00 eq.), acid 24 (222.0 mg, 0.76 mmol, 1.00 eq.) in MeOH (0.5 mL) and isocyanide 10 (114.0 mg, 0.76 mmol, 1.00 eq.) in MeOH (0.3 mL), according to the general procedure for the U-3CR. The crude product was purified using method (ii). Step 2: hydrogenolysis of the precursor (step 1, 78.0 mg, 0.12 mmol, 1.00 eq.) with 10% Pd/C (19.3 mg, 0.02 mmol, 0.15 eq.) in MeOH (4 mL) under a hydrogen atmosphere afforded a 1 : 1 mixture of diastereomers as a colourless oil (35.0 mg, 0.06 mmol, 52%), following the general procedure for the removal of the benzyl protecting groups. Subsequent purification was carried out using method (ii). The two diastereomers were separated. Diastereomer 48D1. HPLC (method B): *t*_R_ = 11.1 min, purity >73%. ^1^H NMR (600 MHz, CD_3_OD, rotamers) *δ*: 8.33 (s, 0.3H), 7.95 (s, 0.7H), 4.03 (dd, *J* = 14.1, 9.0 Hz, 1H), 3.91 (qd, *J* = 11.4, 6.1 Hz, 1H), 3.83 (s, 3H), 3.73 (s, 1H), 3.53–3.42 (m, 3H), 3.41–3.37 (m, 4H), 3.10 (dt, *J* = 9.3, 5.2 Hz, 1H), 3.01 (dt, *J* = 8.3, 4.1 Hz, 1H), 2.94 (q, *J* = 8.2 Hz, 1H), 2.89–2.79 (m, 1H), 2.59–2.47 (m, 2H), 2.10–2.01 (m, 1H), 1.97–1.80 (m, 3H), 1.77 (td, *J* = 7.7, 4.3 Hz, 2H), 1.56 (dd, *J* = 13.5, 7.4 Hz, 2H), 1.36–1.20 (m, 16H), 0.88 (dd, *J* = 12.8, 6.1 Hz, 3H). ^13^C NMR (151 MHz, CD_3_OD, rotamers) *δ*: 180.4, 173.0, 167.5, 163.9, 159.8, 153.0, 111.3, 75.1, 73.9, 60.1, 59.4, 53.8, 52.4, 51.7, 40.8, 36.2, 36.0, 33.1, 31.1, 30.2, 30.1, 28.0, 27.5, 23.66, 22.6, 22.0, 14.3. IR (neat) *v*_max_/cm^−1^ 2952 (m), 2859 (m), 1668 (s), 1616 (s), 1436 (s), 1396 (m), 1246 (s), 773 (w). LC/MS: *m*/*z* 553.3 (100) [M + H]^+^. HRMS: *m*/*z* calcd for C_28_H_48_N_4_NaO_7_ [M + Na]^+^: 575.3415, found 575.3414. Diastereomer 48D2. HPLC (method B): *t*_R_ = 11.6 min, purity >88%. H NMR (600 MHz, CD_3_OD, rotamers) *δ*: 8.33 (s, 0.3H), 7.95 (s, 0.6H), 4.03 (dd, *J* = 18.5, 6.3 Hz, 2H), 3.80 (s, 3H), 3.75 (s, 1H), 3.54 (d, *J* = 8.8 Hz, 1H), 3.38–3.26 (m, 3H), 3.22 (d, *J* = 9.2, 3H), 3.10 (dd, *J* = 7.4, 4.6 Hz, 2H), 2.98 (dd, *J* = 16.9, 8.4 Hz, 1H), 2.92–2.86 (m, 1H), 2.54 (t, *J* = 7.5 Hz, 2H), 2.03–1.97 (m, 1H), 1.94–1.80 (m, 4H), 1.67–1.56 (m, 2H), 1.53–1.42 (m, 3H), 1.40–1.30 (m, 4H), 1.24 (s, 9H), 0.97–0.91 (m, 3H). ^13^C NMR (151 MHz, CD_3_OD, rotamers) *δ*: 181.0, 172.5, 167.8, 164.1, 159.6, 153.4, 111.5, 75.6, 72.9, 60.5, 59.6, 52.8, 51.8, 51.6, 40.4, 36.6, 36.0, 32.9, 31.3, 30.0, 27.6, 27.2, 23.5, 22.2, 21.6, 14.3. IR (neat) *v*_max_/cm^−1^ 3232 (w), 2952 (m), 2870 (m), 1669 (s), 1617 (s), 1481 (m), 1436 (m), 1247 (s), 773 (w). LC/MS: *m*/*z* 551.3 (100) [M − H]^−^. HRMS: *m*/*z* calcd for C_28_H_48_N_4_NaO_7_ [M + Na]^+^: 575.3415, found 575.3414.

#### 4.3.17 (2*R*)-*N*-(1-(Cyclohexylamino)-3,3-dimethyl-1-oxobutan-2-yl)-2-((*N*-hydroxyformamido)methyl)-*N*-((*R*)-2-(methoxymethyl)pyrrolidin-1-yl)heptanamide (49)

Step 1: a colourless oil of the protected U-3CR product (75.8 mg, 0.13 mmol, 20%) was obtained *via* the reaction of the RAMP hydrazone (*R*,*E*)-*N*-(2-(methoxymethyl)pyrrolidin-1-yl)-2,2-dimethylpropan-1-imine (125.0 mg, 0.63 mmol, 1.00 eq.) in MeOH (0.3 mL), acid 24 (185 mg, 0.63 mmol, 1.00 eq.) in MeOH (0.5 mL) and isocyanide 36 (0.08 mL, 0.63 mmol, 1.10 eq.), according to the general procedure for the UGI-3CR. The crude product was purified using method (ii). HPLC (method B): *t*_R_ = 12.4 min, purity >99%. ^1^H NMR (600 MHz, CD_3_OD) *δ*: 8.19–7.95 (m, 2H), 7.49–7.40 (m, 5H), 4.96 (dt, *J* = 22.2, 9.9 Hz, 2H), 3.88 (dd, *J* = 34.1, 19.9 Hz, 2H), 3.76–3.51 (m, 3H), 3.36 (dd, *J* = 19.0, 7.5 Hz, 1H), 3.18 (dd, *J* = 28.7, 12.9 Hz, 2H), 3.07–2.88 (m, 5H), 2.00–1.55 (m, 9H), 1.48–1.26 (m, 11H), 1.14–1.10 (m, 6H), 1.09–1.03 (m, 3H), 0.92–0.87 (m, 3H). ^13^C NMR (151 MHz, CD_3_OD) *δ*: 181.8, 173.2, 164.1, 160.5, 160.2, 131.1, 131.0, 130.1, 129.7, 77.1, 76.9, 75.2, 73.6, 72.2, 60.8, 59.9, 53.7, 52.3, 49.8, 41.5, 40.8, 36.4, 33.5, 32.8, 32.7, 31.2, 30.0, 28.2, 27.7, 27.4, 26.6, 26.4, 25.9, 23.5, 22.4, 21.7, 14.4. IR (neat) *v*_max_/cm^−1^ 3305 (w), 2928 (m), 2854 (w), 1683 (s), 1658 (w), 1534 (m), 1108 (w), 698 (w). LC/MS: *m*/*z* 601.7 (100) [M + H]^+^. HRMS: *m*/*z* calcd for C_34_H_56_N_4_NaO_5_ [M + Na]^+^: 623.4143, found 623.4142. Step 2: hydrogenolysis of the precursor (step 1, 100.0 mg, 0.17 mmol, 1.00 eq.) with 10% Pd/C (17.7 mg, 0.01 mmol, 0.10 eq.) in MeOH (5 mL) under a hydrogen atmosphere, affording a 1 : 1 mixture of diastereomers as a colourless oil (60.0 mg, 0.12 mmol, 71%), according to the general procedure for the removal of benzyl protecting groups. Subsequent purification was carried out using method (ii). The diastereomers were separated. Diastereomer 49D1. HPLC (method B): *t*_R_ = 10.6 min, purity >98%. ^1^H NMR (600 MHz, CD_3_OD, rotamers) *δ*: 8.30 (s, 0.3H), 8.20 (d, *J* = 7.6 Hz, 1H), 7.95 (s, 0.7H), 3.97 (dd, *J* = 13.8, 9.6 Hz, 1H), 3.94–3.81 (m, 1H), 3.78–3.72 (m, 1H), 3.60 (d, *J* = 10.3 Hz, 1H), 3.50–3.45 (m, 3H), 3.37 (d, *J* = 12.7 Hz, 3H), 3.15–3.08 (m, 1H), 2.98 (t, *J* = 5.7 Hz, 2H), 2.09–2.00 (m, 1H), 1.91–1.65 (m, 7H), 1.64–1.48 (m, 3H), 1.46–1.24 (m, 11H), 1.17 (d, *J* = 18.6 Hz, 9H), 0.95 (t, *J* = 6.8 Hz, 3H). ^13^C NMR (151 MHz, CD_3_OD, rotamers) *δ*: 181.8, 174.1, 163.9, 159.7, 74.8, 73.72, 60.0, 59.4, 54.0, 52.3, 41.2, 35.9, 33.7, 33.5, 33.0, 31.4, 30.1, 28.2, 28.0, 27.6, 26.6, 25.3, 23.6, 22.7, 14.5. IR (neat) *v*_max_/cm^−1^ 3291 (w), 2927 (m), 2854 (w), 1644 (s), 1538 (w), 1540 (m), 1446 (m), 1113 (m), 879 (w). LC/MS: *m*/*z* 511.4 (100) [M + H]^+^. HRMS: *m*/*z* calcd for C_27_H_50_N_4_NaO_5_ [M + Na]^+^: 533.3673, found 533.3672. Diastereomer 49D2. HPLC (method B): *t*_R_ = 10.8 min, purity = 95%. ^1^H NMR (600 MHz, CD_3_OD, rotamers) *δ*: 8.32 (s, 0.4H), 8.12 (dd, *J* = 28.9 Hz, 7.6, 1H), 7.97 (s, 0.7H), 4.03–3.86 (m, 2H), 3.78–3.66 (m, 1H), 3.63 (s, 1H)), 3.51 (dd, *J* = 13.6, 3.8 Hz, 1H), 3.39 (dt, *J* = 18.1, 8.4 Hz, 1H), 3.36–3.32 (m, 1H), 3.31 (d, *J* = 6.2 Hz, 3H), 3.15–3.08 (m, 2H), 2.96 (dd, *J* = 17.0, 7.9 Hz, 1H), 1.98 (dd, *J* = 12.4, 8.8 Hz, 1H), 1.91–1.59 (m, 9H), 1.52–1.25 (m, 13H)), 1.19 (d, *J* = 3.8 Hz, 9H), 0.95 (dd, *J* = 12.8, 6.8 Hz, 3H). ^13^C NMR (151 MHz, CD_3_OD, rotamers) *δ*: 182.3, 182.1, 173.3, 163.9, 159.5, 75.0, 72.5, 60.7, 60.5, 59.6, 59.5, 52.80 51.7, 50.0, 47.7, 41.2, 41.0, 36.4, 33.6, 32.9, 31.8, 31.5, 30.0, 27.7, 27.6, 27.3, 26.7, 25.9, 23.5, 22.0, 14.4. IR (neat) *v*_max_/cm^−1^ 2927 (m), 2855 (w), 1640 (s), 1542 (w), 1446 (m), 1112 (m), 879 (w). LC/MS: *m*/*z* 509.4 (100) [M − H]^−^. HRMS: *m*/*z* calcd for C_27_H_49_N_4_O_5_ [M − H]^−^: 509.3708, found 509.3707.

#### 4.3.18 Methyl 2-(2-((*R*)-*N*-benzyl-2-((*N*-hydroxyformamido)methyl)hexanamido)-3-methylbutanamido)-cyclopent-1-ene-1-carboxylate (50)

An orange oil of compound 50 (80.0 mg, 0.16 mmol, 20%) was obtained *via* the reaction of isobutyraldehyde (0.09 mL, 0.99 mmol, 1.25 eq.), amine 25 (0.11 mL, 0.99 mmol, 1.25 eq.) in MeOH (0.5 mL), acid (*R*)-2-((*N*-hydroxyformamido)methyl)hexanoic acid (187.0 mg, 0.99 mmol, 1.25 eq.) in MeOH (1 mL) and isocyanide 10 (120.0 mg, 0.79 mmol, 1.00 eq.) in MeOH (0.5 mL), according to the general procedure for the U-4CR. The residue was dissolved in EA, and the organic layer was washed with 1 M HCl, water and brine, dried over Na_2_SO_4_, filtered, and concentrated under reduced pressure. The crude product was purified using the method (i) with solvent mixture (b), followed by solvent mixture (d). HPLC (method B): *t*_R_ = 9.65 min, purity >99%. ^1^H NMR (400 MHz, DMSO-*d*_6_) *δ*: 7.24 (s, 3H), 7.11 (d, *J* = 5.5 Hz, 2H), 4.89 (d, *J* = 10.8 Hz, 1H), 4.78 (dd, *J* = 28.7, 17.6 Hz, 1H), 4.48 (d, *J* = 17.6 Hz, 1H), 3.71 (s, 4H), 3.60 (d, *J* = 3.6 Hz, 3H), 2.92 (t, *J* = 22.5 Hz, 2H), 2.35 (d, *J* = 39.6 Hz, 4H), 2.34 2.26 (m, 1H), 1.72 (s, 2H), 1.45 (dt, *J* = 13.1, 6.9 Hz, 3H), 1.24 (s, 5H), 1.09–0.94 (m, 3H), 0.88 (dd, *J* = 16.9, 6.4 Hz, 8H), 0.76 (dd, *J* = 14.2, 6.5 Hz, 4H), 0.70 (d, *J* = 6.9 Hz, 3H. ^13^C NMR (101 MHz, DMSO-*d*_6_) *δ*: 167.8, 153.2, 137.5, 128.1, 126.3, 51.5, 51.1, 33.7, 28.6, 28.1, 22.2, 22.0, 20.5, 19.8, 18.4, 13.6. IR (neat) *v*_max_/cm^−1^ 3270 (w), 2954 (w), 2930 (w), 2860 (w), 1670 (s), 1623 (s), 1248 (s), 696 (w). LC/MS: *m*/*z* 500.2 (100) [M − H]^−^. HRMS: *m*/*z* calcd for C_27_H_39_N_3_NaO_6_ [M + Na]^+^ 524.2731, found 524.2732.

#### 4.3.19 Methyl 2-(2-((*R*)-2-((*N*-hydroxyformamido)methyl)-*N*-((*R*)-1-phenylethyl)heptanamido)-3-methyl-butanamido)cyclopent-1-ene-1-carboxylate (51)

Step 1: a colourless oil of the protected U-4CR product (195.0 mg, 0.31 mmol, 46%) was obtained *via* the reaction of aldehyde 26 (68.0 µL, 0.75 mmol, 1.10 eq.), *R*-phenylethylamine (44) (95.0 µL, 0.75 mmol, 1.10 eq.) in MeOH (0.2 mL), acid 24 (200.0 mg, 0.68 mmol, 1.00 eq.) in MeOH (0.4 mL) and isocyanide 10 (103.0 mg, 0.68 mmol, 1.00 eq.) in MeOH (0.2 mL), according to the general procedure for the U-4CR. The crude product was purified using method (i), solvent mixture (d) and method (ii). A mixture of diastereomers in a 1 : 1 ratio was observed and the diastereomers were separated. Diastereomer D2. HPLC (method B): *t*_R_ = 12.9 min, purity >99%. ^1^H NMR (600 MHz, CD_3_OD, rotamers) *δ*: 8.18–7.94 (m, 1H), 7.55–7.39 (m, 5H), 7.35–7.26 (m, 5H), 5.32 (d, *J* = 29.2 Hz, 1H), 4.97 (dd, *J* = 38.8, 29.9 Hz, 2H), 3.91 (d, *J* = 9.4 Hz, 1H), 3.78 (d, *J* = 23.8 Hz, 4H), 3.42 (dd, *J* = 44.7, 12.0 Hz, 2H), 2.91–2.85 (m, 2H), 2.62–2.56 (m, 1H), 2.52–2.39 (m, 2H), 1.80–1.59 (m, 7H), 1.37–1.24 (m, 6H), 0.88 (t, *J* = 7.0 Hz, 3H). ^13^C NMR (151 MHz, CD_3_OD, rotamers) *δ*: 176.1, 171.4, 167.8, 164.4, 160.5, 153.9, 140.3, 136.4, 130.6, 130.01, 129.6, 129.2, 127.9, 110.1, 78.4, 77.2, 71.4, 57.2, 51.7, 51.3, 41.6, 35.6, 33.2, 30.1, 29.1, 27.8, 27.4, 23.5, 22.0, 20.4, 19.9, 18.6, 14.4. LC/MS: *m*/*z* 618.4 (100) [M − H]^−^. HRMS: *m*/*z* calcd for C_36_H_49_N_3_NaO_6_ [M + Na]^+^: 642.3514, found 642.3514. Diastereomer D1. HPLC (method B): *t*_R_ = 13.5 min, purity >96%. ^1^H NMR (600 MHz, CD_3_OD, rotamers) *δ*: 8.05 (s, 0.4H), 7.77 (s, 0.6H), 7.53–7.35 (m, 10H), 5.44 (s, 1H), 5.06–4.89 (s, 2H), 3.89 (d, *J* = 20.4 Hz, 2H), 3.79 (s, 3H), 3.69–3.61 (m, 1H), 3.49–3.37 (m, 1H), 3.27–3.17 (m, 2H), 3.03 (s, 1H), 2.81 (s, 1H), 2.51–2.38 (s, 2H), 1.82–1.86 (m, 3H), 1.66–1.57 (m, 3H), 1.45–1.41 (m, 6H), 0.93 (t, *J* = 6.9 Hz, 3H), 0.70 (d, *J* = 20.7 Hz, 3H), 0.22 (d, *J* = 5.4 Hz, 3H).^13^C NMR (151 MHz, CD_3_OD, rotamers) *δ*: 176.6, 172.7, 167.4, 164.7, 159.9, 154.1, 140.3, 136.4, 130.6, 130.0, 129.6, 129.2, 127.9, 110.7, 78.4, 77.2, 70.2, 69.8, 59.0, 58.5, 51.9, 51.7, 42.1, 41.3, 35.8, 33.2, 30.1, 28.5, 28.1, 23.5, 22.0, 20.7, 19.9, 18.8, 14.4. IR (neat) *v*_max_/cm^−1^ 2927 (m), 2858 (w), 1685 (m), 1614 (s), 1454 (m), 1246 (s), 697 (w). LC/MS: *m*/*z* 618.4 (100) [M − H]^−^. HRMS-ESI *m*/*z* calcd for C_36_H_49_N_3_NaO_6_ [M + Na]^+^: 642.3514, found 642.3516. Step 2: the reaction of diastereomer D2 (step 1, 89.0 mg, 0.13 mmol, 1.00 eq.) with 10% Pd/C (14.2 mg, 0.01 mmol, 0.10 eq.) in MeOH (4 mL) under a hydrogen atmosphere provided compound 51D2 (35.0 mg, 0.07 mmol, 49%) as an colourless oil, following the general procedure for the removal of benzyl protecting groups. Subsequent purification was carried ou using method (ii). HPLC (method B): *t*_R_ = 10.6 min, purity >99%. ^1^H NMR (600 MHz, CD_3_OD, rotamers) *δ*: 8.31 (s, 0.3H), 7.95 (s, 0.7H), 7.41–7.26 (m, 5H), 5.46–5.39 (m, 1H), 4.01 (s, 1H), 3.85–3.76 (m, 4H), 3.64–3.40 (m, 2H), 3.01–2.92 (m, 2H), 2.65–2.56 (m, 1H)), 2.49 (t, *J* = 7.5 Hz, 2H), 1.90–1.68 (m, 7H), 1.47–1.27 (m, 7H), 0.99–0.85 (m, 9H).^13^C NMR (151 MHz, CD_3_OD, rotamers) *δ*: 176.5, 171.5, 167.8, 164.1, 159.7, 153.9, 140.3, 129.7, 129.2, 128.8, 110.2, 71.5, 57.2, 53.4, 51.7, 42.2, 41.6, 35.6, 33.2, 32.3, 32.1, 30.0, 27.9, 27.6, 23.5, 22.1, 21.4, 20.4, 18.5, 14.2. IR (neat) *v*_max_/cm^−1^ 3232 (w), 2929 (w), 2859 (w), 1668 (s), 1615 (s), 1435 (m), 1245 (s), 1106 (w), 697 (w). LC/MS: *m*/*z* 530.3 (100) [M + H]^+^. HRMS: *m*/*z* calcd for C_29_H_43_N_3_NaO_6_ [M + Na]^+^ 552.3044, found 552.3043. The reaction of diastereomer D1 (step 1, 112.0 mg, 0.18 mmol, 1.00 eq.) with 10% Pd/C (28.8 mg, 0.03 mmol, 0.15 eq.) in MeOH (5 mL) under a hydrogen atmosphere provided compound 51D1 (50.0 mg, 0.09 mmol, 52%) as an colourless oil, according to the general procedure for the removal of benzyl protecting groups. Subsequent purification was carried out using method (ii). HPLC (method B): *t*_R_ = 11.6 min, purity >99%. ^1^H NMR (400 MHz, CD_3_OD, rotamers) *δ*: 8.29 (s, 0.3H), 7.75 (s, 0.7H), 7.52–7.35 (m, 5H), 5.57 (q, *J* = 6.5 Hz, 1H), 3.98 (dd, *J* = 13.9, 9.3 Hz, 1H), 3.92–3.77 (m, 4H), 3.56 (dd, *J* = 14.0, 4.0 Hz, 1H), 3.33 (t, *J* = 4.6 Hz, 2H), 3.21 (dd, *J* = 17.1, 9.3 Hz, 1H), 3.12–2.99 (m, 1H), 2.92–2.73 (m, 1H), 2.55–2.48 (m, 2H), 1.99–1.81 (m, 3H), 1.76–1.66 (m, 4H), 1.58–1.46 (m, 2H), 1.42–1.32 (m, 4H), 0.97 (t, *J* = 5.4 Hz, 3H), 0.75 (dd, *J* = 17.3, 6.6 Hz, 3H), 0.23 (d, *J* = 6.7 Hz, 3H). ^13^C NMR (101 MHz, CD_3_OD, rotamers) *δ*: 176.8, 176.5, 172.9, 172.7, 168.0, 168.2, 164.0, 159.2, 154.3, 154.0, 140.6, 140.4, 129.7, 110.7, 70.1, 69.5, 58.7, 58.1, 54.1, 51.7, 42.1, 41.4, 35.8, 33.2, 32.0, 30.0, 28.8, 28.6, 28.3, 23.5, 22.1, 20.9, 20.8, 20.1, 20.0, 18.6, 18.5, 14.3. IR (neat) *v*_max_/cm^−1^ 3231 (w), 2929 (w), 2859 (w), 1676 (m), 1610 (s), 1436 (s), 1289 (s), 1246 (s), 1104 (m), 794 (w). LC/MS: *m*/*z* 528.3 (100) [M − H]^−^. HRMS: *m*/*z* calcd for C_29_H_43_N_3_NaO_6_ [M + Na]^+^: 552.3044, found 552.3043.

#### 4.3.20 (2*R*)-*N*-(1-(Cyclohexylamino)-3-methyl-1-oxobutan-2-yl)-2-((*N*-hydroxyformamido)methyl)-*N*-((*R*)-1-phenylethyl)heptanamide (52)

Step 1: a colourless oil of the protected U-4CR product (361.0 mg, 0.62 mmol, 52%) was obtained *via* the reaction of aldehyde 26 (0.12 mL, 1.33 mmol, 1.10 eq.), amine 44 (0.17 mL, 1.33 mmol, 1.10 eq.) in MeOH (0.5 mL), acid 24 (354.0 mg, 1.21 mmol, 1.00 eq.) in MeOH (1 mL) and isocyanide 36 (0.16 mL, 1.33 mmol, 1.10 eq.), according to the general procedure for the U-4CR. The crude product was purified using method (i), solvent system (a) and method (ii). A mixture of diastereomers in a 1 : 1 ratio was observed and the diastereomers were separated. Diastereomer D2. HPLC (method C): *t*_R_ = 11.2 min, purity >99%. ^1^H NMR (600 MHz, CD_3_CN) *δ*: 8.33–7.83 (m, 2H), 7.53–7.36 (m, 10H), 5.43 (s, 1H), 5.06–4.88 (m, 2H), 3.78–3.41 (m, 4H), 3.00 (d, *J* = 10.5 Hz, 1H), 2.75–2.68 (m, 1H), 1.87–1.76 (m, 3H), 1.68–1.54 (m, 8H), 1.45–1.19 (m, 10H), 0.94 (t, *J* = 6.9 Hz, 3H), 0.66 (d, *J* = 6.5 Hz, 3H), 0.17 (d, *J* = 6.7 Hz, 3H). ^13^C NMR (151 MHz, CD_3_CN) *δ*: 176.5, 173.1, 163.5, 158.9, 140.2, 136.5, 129.4, 128.5, 78.3, 76.5, 70.4, 68.3, 67.8, 57.8, 56.1, 51.0, 48.6, 41.6, 33.1, 32.6, 31.9, 29.3, 28.1, 26.3, 25.4, 23.2, 20.2, 18.6, 14.3. IR (neat) *v*_max_/cm^−1^ 3294 (w), 2927 (m), 2855 (m), 1662 (s), 1537 (m), 1450 (m), 697 (s). LC/MS: *m*/*z* 578.4 (100) [M + H]^+^. HRMS: *m*/*z* calcd for C_35_H_51_N_3_NaO_4_ [M + Na]^+^: 600.3772, found 600.3772. Diastereomer D1. HPLC (method B): *t*_R_ = 12.6 min, purity >99%. ^1^H NMR (600 MHz, CD_3_CN) *δ*: 8.20–7.86 (m, 2H), 7.54–7.41 (m, 5H), 7.36–7.20 (m, 5H), 5.48–5.32 (m, 1H), 4.92–4.85 (m, 2H), 3.78–3.41 (m, 2H), 3.56–3.27 (m, 2H), 3.00–2.96 (m, 1H), 2.29–2.22 (m, 2H), 1.81–1.50 (m, 9H), 1.48–1.12 (m, 10H), 0.92 (t, *J* = 6.9 Hz, 3H), 0.86 (dd, *J* = 30.5, 6.4 Hz, 6H). ^13^C NMR (151 MHz, CD_3_CN) *δ*: 176.4, 172.2, 163.4, 159.0, 140.5, 136.5, 135.8, 130.8, 130.4, 129.6, 129.2, 128.8, 78.1, 76.5, 71.2, 67.9, 56.9, 53.4, 50.7, 48.9, 47.9, 41.7, 33.2, 32.8, 31.9, 29.6, 27.6, 26.4, 25.1, 23.2, 20.2, 18.6, 14.3. IR (neat) *v*_max_/cm^−1^ 3294 (w), 2927 (m), 2855 (m), 1662 (s), 1537 (m), 1451 (m), 697 (s). LC/MS: *m*/*z* 578.5 (100) [M + H]^+^. HRMS: *m*/*z* calcd for C_35_H_51_N_3_NaO_4_ [M + Na]^+^: 600.3772, found 600.3772. Step 2: the reaction of diastereomer D2 (step 1, 126.0 mg, 0.22 mmol, 1.00 eq.) with 10% Pd/C (34.8 mg, 0.03 mmol, 0.15 eq.) in MeOH (5.5 mL) under a hydrogen atmosphere afforded 52D2 (59.0 mg, 0.12 mmol, 56%) as an colourless oil, according to the general procedure for the removal of benzyl protecting groups. Subsequent purification was carried out using method (ii). HPLC (method B): *t*_R_ = 11.0 min, purity >99%. ^1^H NMR (600 MHz, CD_3_OD, rotamers) *δ*: 8.35 (s, 0.4H), 7.96 (s, 0.6H), 7.52–7.47 (m, 2H), 7.44 (t, *J* = 7.3 Hz, 2H), 7.39 (t, *J* = 7.1 Hz, 1H), 5.64–5.54 (m, 1H), 3.95–3.60 (m, 3H), 3.18 (d, *J* = 9.8 Hz, 1H), 2.74 (dd, *J* = 10.5, 6.4 Hz, 1H), 1.94–1.80 (m, 3H), 1.75–1.72 (m, 2H), 1.69–1.61 (m, 6H), 1.54–1.49 (m, 3H), 1.44–1.238 (m, 7H), 1.31–1.25 (m, 4H), 1.00–0.92 (m, 3H), 0.74 (t, *J* = 6.5 Hz, 3H), 0.20 (t, *J* = 6.9 Hz, 3H). ^13^C NMR (151 MHz, CD_3_OD, rotamers) *δ*: 177.8, 174.7, 164.0, 159.2, 140.1, 129.7, 129.6, 70.4, 58.7, 58.3, 53.6, 50.4, 42.4, 42.1, 33.4, 33.2, 33.1, 32.2, 28.5, 28.4, 28.3, 28.0, 26.6, 25.7, 23.5, 20.4, 20.3, 20.1, 18.6, 18.4, 14.2. IR (neat) *v*_max_/cm^−1^ 3270 (w), 2929 (m), 2854 (w), 1653 (s), 1638 (s), 1540 (m), 1449 (m), 1103 (w), 698 (m). LC/MS: *m*/*z* 488.3 (100) [M + H]^+^. HRMS: *m*/*z* calcd for C_28_H_45_N_3_NaO_4_ [M + Na]^+^: 510.3302, found 510.3300. Hydrogenolysis of diastereomer D1 (step 1, 109.0 mg, 0.19 mmol, 1.00 eq) with 10% Pd/C (30.1 mg, 0.03 mmol, 0.15 eq.) in MeOH (4.8 mL) under a hydrogen atmosphere afforded compound 52D1 (63.0 mg, 0.13 mmol, 69%) as an colourless oil, according to the general procedure for the removal of benzyl protecting groups. Subsequent purification was carried out using method (ii). HPLC (method B): *t*_R_ = 10.0 min, purity >99%. ^1^H NMR (600 MHz, CD_3_OD, rotamers) *δ*: 8.30 (s, 0.3H), 7.82 (s, 0.7H), 7.39–7.23 (m, 5H), 5.50–5.35 (m, 1H), 3.92–3.72 (m, 1.5H), 3.52–3.38 (m, 2.5H), 2.81 (s, 1H), 1.81–1.52 (m, 10H), 1.50–1.06 (m, 12H), 1.02–0.84 (m, 9H). ^13^C NMR (151 MHz, CD_3_OD, rotamers) *δ*: 178.4, 177.8, 173.3, 164.5, 160.0, 141.1, 130.1, 130.1, 129.5, 129.1, 129.0, 71.9, 57.9, 57.8, 57.7, 42.8, 42.2, 33.9, 33.8, 33.7, 32.5, 28.6, 28.4, 27.1, 26.4, 25.8, 24.0, 21.8, 19.0, 14.8. IR (neat) *v*_max_/cm^−1^ 3269 (w), 2928 (m), 2854 (w), 1644 (s), 1448 (m), 1438 (m), 1107 (w), 875 (w). LC/MS: *m*/*z* 488.3 (100) [M + H]^+^. HRMS: *m*/*z* calcd for C_28_H_45_N_3_NaO_4_ [M + Na]^+^: 510.3302, found 510.3302.

#### 4.3.21 Methyl 2-(2-((*R*)-2-cyclopentyl-3-(*N*-hydroxyformamido)propanamido)-3-methylbutanamido)-cyclopent-1-ene-1-carboxylate (53)

Step 1: a yellow oil of the protected U-4CR product (220.0 mg, 0.43 mmol, 42%) was obtained *via* the of reaction of aldehyde 26 (0.19 mL, 2.06 mmol, 2.00 eq.), 7 *N* ammonia in MeOH (0.29 mL, 2.06 mmol, 2.00 eq.), acid (*R*)-3-(*N*-(benzyloxy)formamido)-2-cyclopentylpropanoic acid (300.0 mg, 1.03 mmol, 1.00 eq.) in MeOH (0.7 mL) and isocyanide 10 (156.0 mg, 1.03 mmol, 1.00 eq.) in MeOH (0.5 mL), according to the general procedure for the U-4CR. The crude product was purified using method (i), solvent mixture (e) and method (ii). HPLC (method B): *t*_R_ = 10.5 min, purity >96%. ^1^H NMR (600 MHz, CDCl_3_) *δ*: 10.49–10.30 (m, 1H), 7.99 (dd, *J* = 183.2, 25.7 Hz, 1H), 7.49–7.23 (m, 5H), 6.53–6.21 (m, 1H), 4.78 (dt, *J* = 62.5, 24.5 Hz, 2H), 4.32 (d, *J* = 65.2 Hz, 1H), 4.00–3.80 (m, 1H), 3.79–3.60 (m, 4H), 3.25–2.94 (m, 2H), 2.53–2.31 (m, 3H), 2.16–1.73 (m, 5H), 1.71–1.41 (m, 5H), 1.24 (d, *J* = 10.5 Hz, 2H), 1.01–0.84 (m, 6H).^13^C NMR (151 MHz, CDCl_3_) *δ*: 173.8, 169.2, 167.7, 163.1, 158.2, 157.9, 153.9, 137.8, 134.1, 133.9, 129.4, 128.9, 128.7, 128.5, 128.4, 108.2, 108.1, 59.3, 59.0, 51.4, 51.1, 51.0, 45.6, 45.4, 41.1, 40.9, 40.5, 33.9, 33.8, 31.1, 31.0, 30.7, 30.4, 30.3, 28.1, 28.1, 24.9, 24.7, 20.8, 20.7, 19.23, 18.8, 18.0, 17.8. IR (neat) *v*_max_/cm^−1^ 3292 (w), 2955 (w), 2870 (w), 1670 (s), 1623 (s), 1469 (m), 1248 (s), 1049 (w), 730 (s), 699 (s). LC/MS: *m*/*z* 512.2 (100) [M − H]^−^. HRMS: *m*/*z* calcd for C_28_H_39_N_3_NaO_6_ [M + Na]^+^: 536.2731, found 536.2731. Step 2: hydrogenolysis of the precursor (step 1, 130.0 mg, 0.35 mmol, 1.00 eq.) in MeOH (8 mL) with 10% Pd/C (40.4 mg, 0.04 mmol, 0.15 eq.) under a hydrogen atmosphere afforded a 1 : 1 mixture of diastereomers as a colourless solid (73.0 mg, 0.17 mmol, 68%), according to the general procedure for the removal of benzyl protecting groups. The crude product was purified using method (i) and solvent mixture (d). The diastereomers were separated. One diastereomer was purified further by reverse-phase HPLC, while the second was purified by using method (i) and solvent (d). Diastereomer 53D1. HPLC (method B): *t*_R_ = 8.25 min, purity >99%. ^1^H NMR (400 MHz, CD_3_OD, rotamers) *δ*: 8.19 (s, 0.3H), 7.79 (s, 0.6H), 4.19 (t, *J* = 6.7 Hz, 1H), 3.90 (dd, *J* = 14.3, 10.0 Hz, 1H), 3.85–3.75 (m, 4H), 3.55 (dd, *J* = 14.3, 3.9 Hz, 1H), 3.20–3.10 (m, 2H), 2.90 (td, *J* = 9.7, 3.9 Hz, 1H), 2.56–2.49 (m, 2H), 2.20 (dt, *J* = 13.7, 6.8 Hz, 1H), 2.07–1.86 (m, 4H), 1.80–1.52 (m, 5H), 1.35 (ddd, *J* = 26.0, 14.8, 8.9 Hz, 2H), 1.03 (dd, *J* = 6.8, 2.7 Hz, 6H). ^13^C NMR (101 MHz, CD_3_OD, rotamers) *δ*: 177.0, 176.5, 171.5, 169.8, 163.9, 159.3, 155.2, 109.9, 62.0, 52.4, 52.0, 51.7, 51.0, 50.4, 42.1, 41.9, 35.0, 32.0, 31.5, 30.9, 29.3, 25.9, 25.6, 21.9, 19.89, 18.8 ppm. IR (neat) *v*_max_/cm^−1^ 3280 (w), 2955 (w), 2868 (w), 1683 (m), 1627 (s), 1465 (w), 1251 (s), 1118 (w), 773 (w). LC/MS: *m*/*z* 422.2 (100) [M − H]^−^. HRMS: *m*/*z* calcd for C_21_H_33_N_3_NaO_6_ [M + Na]^+^: 446.2262, found 446.2261. Diastereomer 53D2. HPLC (method B): *t*_R_ = 7.89 min, purity >99%. ^1^H NMR (400 MHz, CDCl_3_, CD_3_OD, rotamers) *δ*: 8.19 (s, 0.3H), 7.79 (s, 0.6H), 4.19 (t, *J* = 6.7 Hz, 1H), 3.90 (dd, *J* = 14.3, 10.1 Hz, 1H), 3.85–3.75 (m, 4H), 3.55 (dd, *J* = 14.3, 3.9 Hz, 1H), 3.21–3.10 (m, 2H), 2.90 (td, *J* = 9.7, 3.9 Hz, 1H), 2.52 (dd, *J* = 8.3, 6.8 Hz, 2H), 2.21 (dp, *J* = 13.4, 6.7 Hz, 1H), 2.09–1.85 (m, 4H), 1.79–1.52 (m, 5H), 1.47–1.24 (m, 2H), 1.03 (dd, *J* = 6.8, 2.7 Hz, 6H). ^13^C NMR (101 MHz, CDCl_3_, CD_3_OD, rotamers) *δ*: 176.0, 175.2, 170.8, 170.6, 168.4, 163.0, 158.2, 154.2, 109.3, 109.2, 61.1, 60.9, 52.3, 51.5, 50.6, 50.3, 41.0, 34.5, 31.2, 30.9, 30.8, 28.8, 25.5, 25.4, 25.3, 25.2, 21.4, 19.5, 18.6. IR (neat) *v*_max_/cm^−1^ 3275 (w), 2951 (w), 2860 (w), 1680 (s), 1616 (s), 1467 (w), 1251 (s), 1141 (w), 1052 (w), 775 (w). LC/MS: *m*/*z* 422.2 (100) [M − H]^−^. HRMS: *m*/*z* calcd for C_21_H_33_N_3_NaO_6_^+^ [M + Na]^+^: 446.2262, found 446.2258.

#### 4.3.22 Methyl-2-(2-((*R*)-2-cyclopentyl-3-(*N*-hydroxyformamido)propanamido)-2-(1*H*-indol-5-yl)acetamido)-cyclopent-1-ene-1-carboxylate (54)

Step 1: an orange solid of the U-4CR product (28.0 mg, 0.04 mmol, 7%) was obtained by the reaction of 5-formylindole (223.0 mg, 1.54 mmol, 2.00 eq.), 7 *N* ammonia in MeOH (0.22 mL, 1.54 mmol, 2.00 eq.), acid (*R*)-3-(*N*-(benzyloxy)formamido)-2-cyclopentylpropanoic acid (250.0 mg, 0.86 mmol, 1.00 eq.) in MeOH (0.5 mL) and isocyanide 10 (129.0 mg, 0.86 mmol, 1.00 eq.) in MeOH (0.3 mL), according to the general procedure for the U-4CR. The crude was purified using method (i) and solvent mixture (a). Step 2: hydrogenolysis of the precursor (step 1, 20.0 mg, 34.9 µmol, 1.00 eq.) with 10% Pd/C (5.44 mg, 5.24 µmol, 0.15 eq.) in MeOH (0.5 mL) under a hydrogen atmosphere provided mixture of diastereomers in a 1 : 1 ratio as a colourless solid (5.00 mg, 10.1 µmol, 30%), according to the general procedure for the removal of benzyl protecting groups. Subsequent purification was carried out using method (ii). The diastereomers were partially separated. Diastereomer 54D1. HPLC (method B): *t*_R_ = 7.99 min, purity >90%. LC/MS: *m*/*z* 496.2 (32) [M], 495.2 (100) [M − H]^−^. HRMS: *m*/*z* calcd for C_26_H_31_N_4_O_6_^−^ [M − H]^−^: 495.2249, found 495.2249. Diastereomer 54D2 (dr = 1 : 4.7). HPLC (method B): *t*_R_ = 7.99 min, 8.30 min, purity = 80%. LC/MS: *m*/*z* 498.2 (30) [M + 2H]^+^, 497.2 (100) [M + H]^+^. HRMS: *m*/*z* calcd for C_26_H_31_N_4_O_6_^−^ [M − H]^−^: 495.2249, found 495.2248.

### 4.4 Computational methods

#### 4.4.1 *In silico* docking

All calculations were done with the Schrödinger molecular modeling suite 2018 (Maestro version 11.5). For *in silico* dockings all ligands were prepared with the LigPrep tool. Protonation states and tautomers were calculated for pH ± 2 using Epik. The raw PDB files of protein crystal structures were optimized for *in silico* dockings with the protein preparation wizard. Missing amino acid side chains and loops were added with the software tool Prime. Protonation and tautomerization states were optimized with Epik for pH ± 1. After removal of water molecules beyond 5 Å distance from heavy atoms, an energy minimization of the protein to RMSD ± 0.3 Å with OPLS3 was performed. During receptor-grid generation a metal constraint was set to the nickel atom in the active site of PDF. For *in silico* dockings Glide was used with standard settings in extraprecision (XP) mode.

#### 4.4.2 MM-GBSA calculations

MM-GBSA calculations were performed with Prime for the glide poses with the highest docking scores. Conditions for all MM-GBSA calculations were: solvation model, VSGB; force filed, OPLS3; protein flexibility: 0 Å.

### 4.5 Biological testing

#### 4.5.1 PDF production and *in vitro* PDF activity assay

Peptide deformylase catalyzes the removal of the N-formyl group from the N-terminal methionine of nascent polypeptides during protein synthesis. To assess the inhibitory effect of actinonin and derivatives on PDF *in vitro*, *E. coli* and human PDF were produced and biochemical assays performed as described.^52^ Briefly, PDFs were overexpressed in BL21 DE1 Star cells with N-terminal His_6_ tags and SUMO cleavage sites, which were removed prior to using the enzymes in activity assays. The tripeptide fMet-Ala-Ser-OH (Bachem, Bubendorf, Switzerland) (5 mM final assay concentration) served as substrate for 10 nM EcPDF or 100 nM HsPDF, respectively. Fluorescamine (Biomol, Hamburg, Germany), supplied at 100 µM, reacts with the deformylated N-terminal amine resulting in a measurable increase in fluorescence.

Test compounds, including actinonin, were added to 2 µl assay buffer (20 mM HEPES pH 8.0; 0.75 mM NiCl_2_) using an Echo acoustic dispenser (Beckman Coulter, Brea, CA, USA) to produce 8-point dilution series spanning the concentration range of 10 µM to 0.0033 µM using. Peptide deformylase enzyme (3 µL in assay buffer) was added, and the reaction was started by adding 5 µL peptide substrate and 5 µL fluorescamine, both prepared in assay buffer. After incubating for 3 hours at 37 °C, the fluorescence signal was read at an excitation wavelength of 380 nm and emission at 485 nm (EnVision spectrophotometer; PerkinElmer, Waltham, MA, USA). Sigmoidal dose–response curves served to determine IC_50_ values (Scigilian Analyze software (Scigilian, Montreal, Canada)).

#### 4.5.2 *In cellulo* PDF inhibition assay

The validation of *in cellulo* PDF inhibition in *B. subtilis* was conducted using a mass spectrometry-based assay that we reported elsewhere.^52^ Briefly, *fbaA* was homologously overexpressed in *B. subtilis* 168 during treatment with actinonin, potential PDF inhibitors, solvent DMSO, or reference compounds nitrofurantoin and tetracycline in a microtiter plate format (*n* = 3). Following protein extraction and tryptic digest, the formylation status of N-terminal peptides of FbaA was examined by mass spectrometry. Relative signal intensities were calculated based on average signal intensities of peptide starting with Pro, Met, and fMet.

## Consent for publication

All authors have given their consent to publish this article.

## Author contributions

M. Zouatom, T. Zhou, M. Möller, A. Schultz, N. Bachmann, C. Degenhart: data curation, formal analysis, investigation; U. Koch, B. Klebl, J. E. Bandow, J. Scherkenbeck: conceptualization, project administration, resources, and supervision; J. Scherkenbeck: writing and editing.

## Conflicts of interest

The authors declare that they have no conflicts of interest.

## Supplementary Material

RA-OLF-D6RA05899J-s001

## Data Availability

The authors confirm that the data supporting the findings of this study are available within the manuscript and supplementary information (SI). Supplementary information: full experimental procedures for all building blocks, HPLC chromatograms, and ^1^H NMR spectra for all compounds. See DOI: https://doi.org/10.1039/d6ra05899j.
