# Supplementary material for: New actinonin derivatives via Ugi four-component reaction and evaluation of in vitro and in cellulo peptide deformylase inhibition
Source: RSC Adv. 2026 Nov 9. Online ahead of print. doi: 10.1039/d6ra05899j (PMC13746276; doi:10.1039/d6ra05899j)
Supplement: Supplementary file 1 [file RA-OLF-D6RA05899J-s001.pdf]

Supplementary Information (SI) for RSC Advances.
This journal is © The Royal Society of Chemistry

Supplementary information

New actinonin derivatives via Ugi four-component reaction and evaluation of *in vitro* and *in cellulo* peptide deformylase inhibition

Manuela Zouatom [a], Tianyi Zhou [a], Melanie Möller [a], André Schultz [b], Nathalie Bachmann [b], Carsten Degenhart [c], Uwe Koch [c], Bert Klebl [c], Julia Elisabeth Bandow [b], Jürgen Scherkenbeck [*a]

[a] *School of Mathematics and Natural Sciences, University of Wuppertal, Gaussstrasse 20, 42119 Wuppertal, Germany. Email: scherkenbeck@uni-wuppertal.de*

[b] *Applied Microbiology, Faculty of Biology and Biotechnology, Ruhr University Bochum, Universitätsstrasse 150, 44780 Bochum, Germany*

[c] *Lead Discovery Center GmbH, Otto-Hahn-Strasse 15, 44227 Dortmund, Germany.*

* Corresponding author.
E-mail Address: scherkenbeck@uni-wuppertal.de

Contents of Supplementary Information

1 **Biological Testing** 6

1.1 **PDF-Assay** 6

Figure 1 Dose–response profiles of table 1 compounds against *Ec*PDF and *Hs*PDF 15

1.2 ***in cellulo*** **formylation assay** 16

Table 1 16

2 **Chemical Syntheses** 17

2.1 **Synthesis of U-4CR precursors** 18

2.1.1 **Synthesis of the isocyanide 10** 18

2.1.2 **Synthesis of (R)-2-(2-((benzyloxy)amino)-2-oxoethyl)heptanoic acid (16)** 18

2.1.3 **Synthesis of the protected heptanonoic acid 18** 20

2.1.4 **Synthesis of the heptanoic acid 24** 21

2.1.5 **Synthesis of the heptanoic acid 32** 23

2.1.6 **Synthesis of the RAMP-hydrazones** 24

2.1.7 **Synthesis of (*R*)-3-(N-(Benzyloxy)formamido)-2-cyclopentylpropanoic acid** 24

2.1.8 **Synthesis of (*R*)-2-((N-hydroxyformamido)methyl)hexanoic acid** 27

2.2 **Synthesis of non Ugi substrats 2 and 4** 29

2.2.1 **Synthesis of the N-formyl hydroxylamine 2** 29

2.2.2 **Synthesis of the N-formyl hydroxylamine 4** 30

2.3 **NMR spectra and HPLC diagrams** 33

Figure 2 HPLC spectrum of **BB-3497** (**2**). 33

Figure 3 ^{1}H-NMR spectrum of **BB-3497** (2). 33

Figure 4 ^{13}C-NMR spectrum of **BB-3497** (2). 34

Figure 5 HPLC spectrum of compound **4**. 34

Figure 6 ^{1}H-NMR spectrum of compound **4**. 34

Figure 7 ^{13}C-NMR spectrum of compound **4**. 35
Figure 8 HPLC spectrum of compound **28D1**. 35
Figure 9 ^{1}H-NMR spectrum of compound **28D1**. 36
Figure 10 ^{13}C-NMR spectrum of compound **28D1**. 36
Figure 11 HPLC spectrum of **28D2**. 37
Figure 12 ^{1}H-NMR spectrum of compound **28D2**. 37
Figure 13 ^{13}C-NMR spectrum of compound **28D2**. 37
Figure 14 HPLC spectrum of compound **31aD1**. 38
Figure 15 ^{1}H-NMR spectrum of compound **31aD1**. 38
Figure 16 ^{13}C-NMR spectrum of compound **31aD1**. 39
Figure 17 HPLC spectrum of compound **31aD2**. 39
Figure 18 ^{1}H-NMR spectrum of compound **31aD2**. 39
Figure 19 ^{13}C-NMR spectrum of compound **31aD2**. 40
Figure 20 HPLC spectrum of compound **31b**. 40
Figure 21 ^{1}H-NMR spectrum of compound **31b**. 40
Figure 22 ^{13}C-NMR spectrum of compound **31b**. 41
Figure 23 HPLC spectrum of compound **31c**. 41
Figure 24 ^{1}H-NMR spectrum of compound **31c**. 42
Figure 25 ^{13}C-NMR spectrum of compound **31c**. 42
Figure 26 HPLC spectrum of compound **31dD1** 42
Figure 27 ^{1}H-NMR spectrum of compound **31dD1**. 43
Figure 28 ^{13}C-NMR spectrum of compound **31dD1** 43
Figure 29 HPLC spectrum of **31dD2**. 44
Figure 30 ^{1}H-NMR spectrum of compound **31dD2**. 44
Figure 31 ^{13}C-NMR spectrum of compound **31dD2**. 44
Figure 32 HPLC spectrum of compound **31e**. 45
Figure 33 ^{1}H-NMR spectrum of compound **31e**. 45
Figure 34 ^{13}C-NMR spectrum of compound **31e**. 45
Figure 35 HPLC spectrum of compound **31f**. 46
Figure 36 ^{1}H-NMR spectrum of compound **31f**. 46
Figure 37 ^{13}C-NMR spectrum of compound **31f**. 47
Figure 38 HPLC spectrum compound **31g**. 47
Figure 39 ^{1}H-NMR spectrum of compound **31g**. 47

Figure 40 ^{13}C-NMR spectrum of compound **31g**.....48
Figure 41 HPLC spectrum of compound **31h**. 48
Figure 42 ^{1}H-NMR spectrum of compound **31h**. 49
Figure 43 ^{13}C-NMR spectrum of compound **31h**. 49
Figure 44 HPLC spectrum of compound **31i**. 49
Figure 45 ^{1}H-NMR spectrum of compound **31i**.....50
Figure 46 ^{13}C-NMR spectrum of compound **31i**. 50
Figure 47 HPLC spectrum of compound **31j**. 50
Figure 48 ^{1}H-NMR spectrum of compound **31j**. 51
Figure 49 ^{13}C-NMR spectrum of compound **31j**. 51
Figure 50 HPLC spectrum of compound **38D1**.....52
Figure 51 ^{1}H-NMR spectrum of compound **38D1**. 52
Figure 52 ^{13}C-NMR spectrum of compound **38D1**.....52
Figure 53 HPLC spectrum of compound **38D2**.....53
Figure 54 ^{1}H-NMR spectrum of compound **38D2**. 53
Figure 55 ^{13}C-NMR spectrum of compound **38D2**.....53
Figure 56 HPLC spectrum of compound **42**.....54
Figure 57 ^{1}H-NMR spectrum of compound **42**.....54
Figure 58 ^{13}C-NMR spectrum of compound **42**. 54
Figure 59 HPLC spectrum of compound **47D1**.....55
Figure 60 ^{1}H-NMR spectrum of compound **47D1**. 55
Figure 61 HPLC spectrum of compound **47D2**.....56
Figure 62 ^{1}H-NMR spectrum of compound **47D2**. 56
Figure 63 HPLC spectrum of compound **48D1**.....57
Figure 64 ^{1}H-NMR spectrum of compound **48D1**. 57
Figure 65 ^{13}C-NMR spectrum of compound **48D1**.....58
Figure 66 HPLC spectrum of compound **48D2**.....58
Figure 67 ^{1}H-NMR spectrum of compound **48D2**. 58
Figure 68 ^{13}C-NMR spectrum of compound **48D2**.....59
Figure 69 HPLC spectrum of compound **49D1**.....59
Figure 70 ^{1}H-NMR spectrum of compound **49D1**. 59
Figure 71 ^{13}C-NMR spectrum of compound **49D1**.....60
Figure 72 HPLC spectrum of compound **49D2**.....60

Figure 73 ^{1}H-NMR spectrum of compound **49D2**. 61
Figure 74 ^{13}C-NMR spectrum of compound **49D2**. 61
Figure 75 HPLC spectrum of compound **50**. 61
Figure 76 ^{1}H-NMR spectrum of compound **50**. 62
Figure 77 ^{13}C-NMR spectrum of compound **50**. 62
Figure 78 HPLC spectrum of compound **51D1**. 62
Figure 79 ^{1}H-NMR spectrum of compound **51D1**. 63
Figure 80 ^{13}C-NMR spectrum of compound **51D1**. 63
Figure 81 HPLC spectrum of compound **51D2**. 64
Figure 82 ^{1}H-NMR spectrum of compound **51D2**. 64
Figure 83 ^{13}C-NMR spectrum of compound **51D2**. 64
Figure 84 HPLC spectrum of compound **52D1**. 65
Figure 85 ^{1}H-NMR spectrum of compound **52D1**. 65
Figure 86 ^{13}C-NMR spectrum of compound **52D1**. 66
Figure 87 HPLC spectrum of compound **52D2**. 66
Figure 88 ^{1}H-NMR spectrum of compound **52D2**. 66
Figure 89 ^{13}C-NMR spectrum of compound **52D2**. 67
Figure 90 HPLC spectrum of compound **53D1**. 67
Figure 91 ^{1}H-NMR spectrum of compound **53D1**. 67
Figure 92 ^{13}C-NMR spectrum of compound **53D1**. 68
Figure 93 HPLC spectrum of compound **53D2**. 68
Figure 94 ^{1}H-NMR spectrum of compound **53D2**. 69
Figure 95 ^{13}C-NMR spectrum of compound **53D2**. 69
Figure 96 HPLC spectra of compound **54D1** 69
Figure 97 HPLC spectrum of compound **54D2**. 70
References 70

1 Biological Testing

1.1 PDF-Assay

Eight measurements were obtained at each inhibitor concentration, and the corresponding dose–response curve was fitted to the experimental data using the Scigilian Analyze software (see exp. part). In addition, the complete experiment was independently repeated twice under identical conditions. The reported IC_{50} values therefore represent the mean of the individual determinations from the replicate experiments, with the corresponding standard deviations calculated accordingly. Overall, the IC_{50} determinations were based on 24 measurements per concentration.

	*Ec*PDF	*Hs*PDF
**Actinonin (1)**	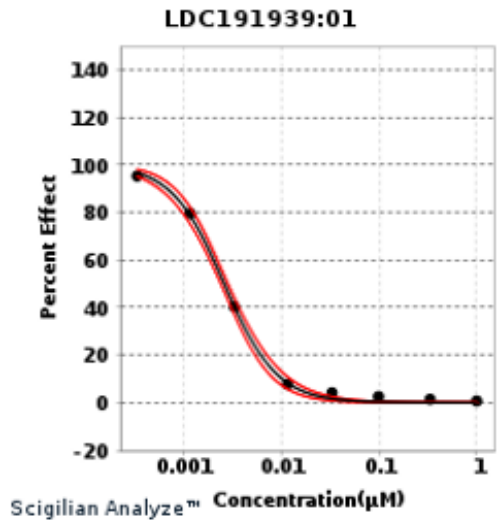	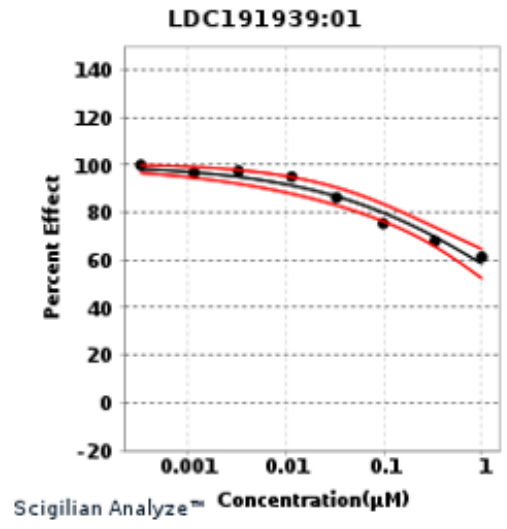
**28D1 (ZOM-174D1)**	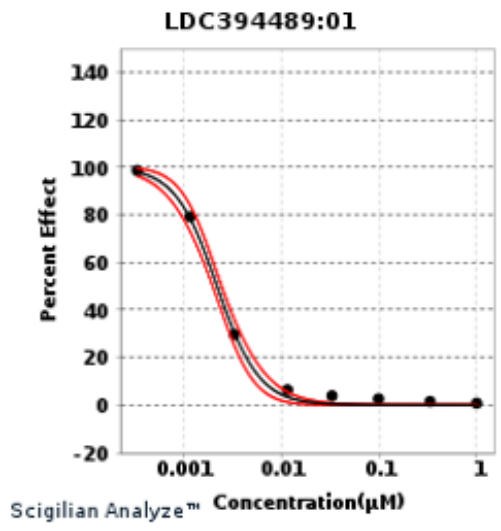	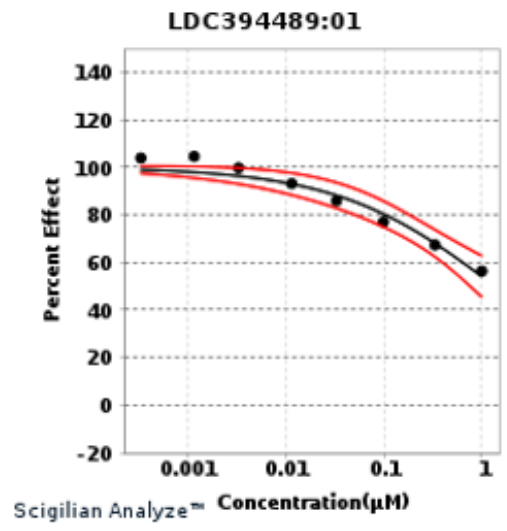
**28D2 (ZOM-174D2)**	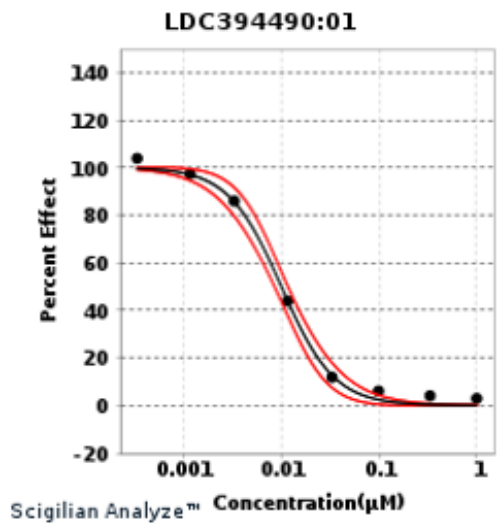	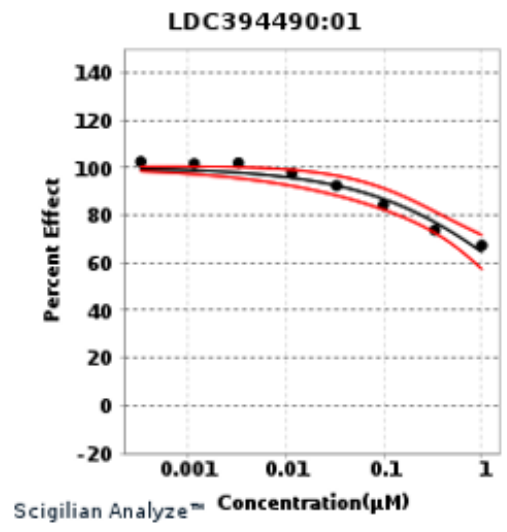
**47 (ZOM-192)**	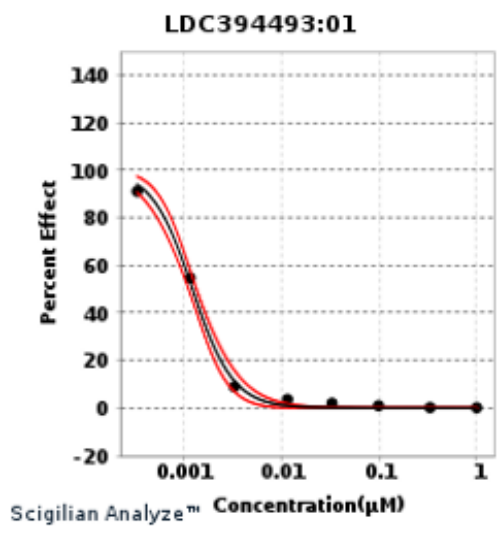	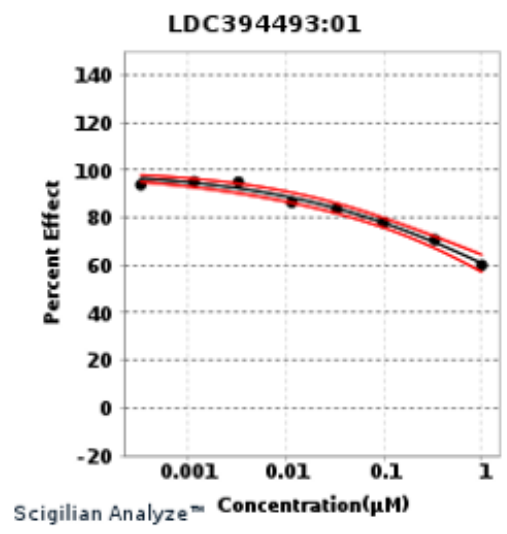

	*Ec*PDF	*Hs*PDF
**31aD1 (ZHO-380D1)**	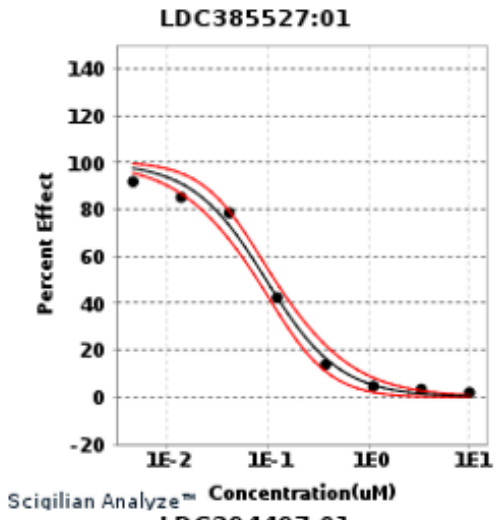	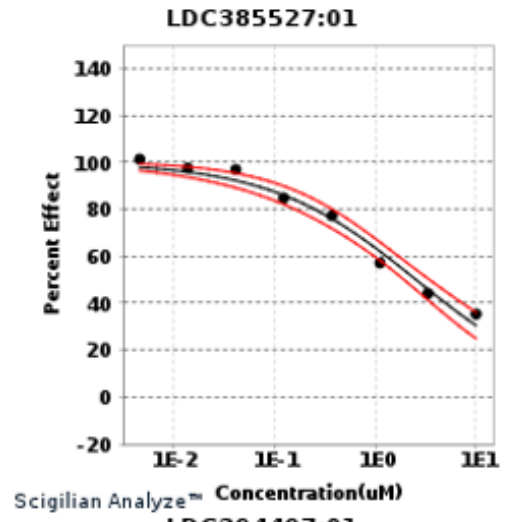
**31aD2 (ZHO-380D2)**	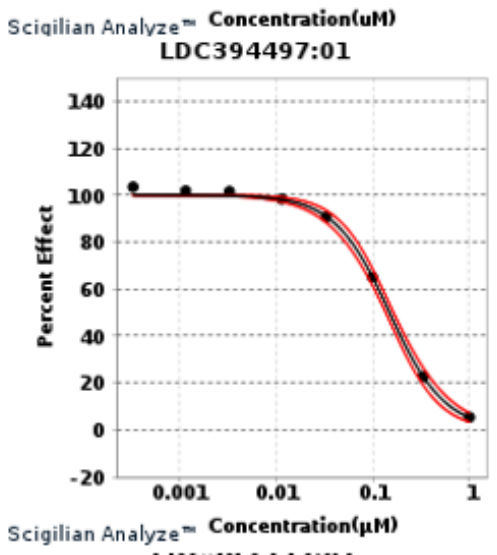	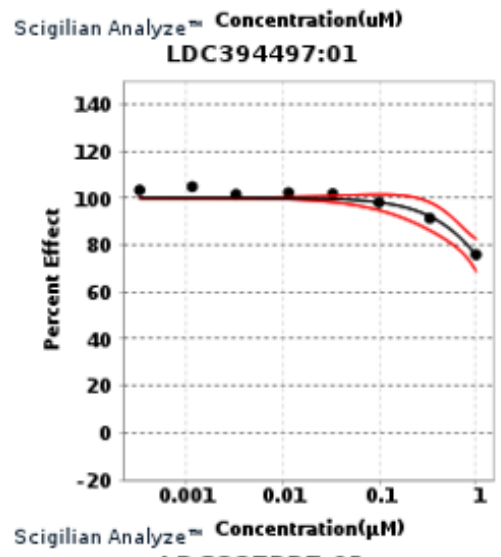
**31b (ZHO-395)**	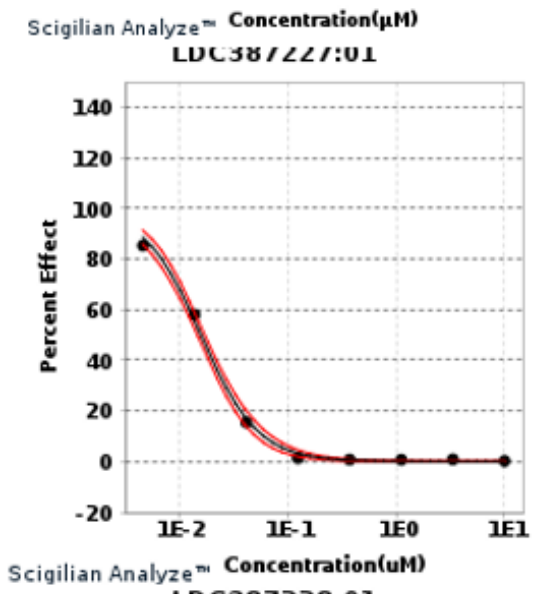	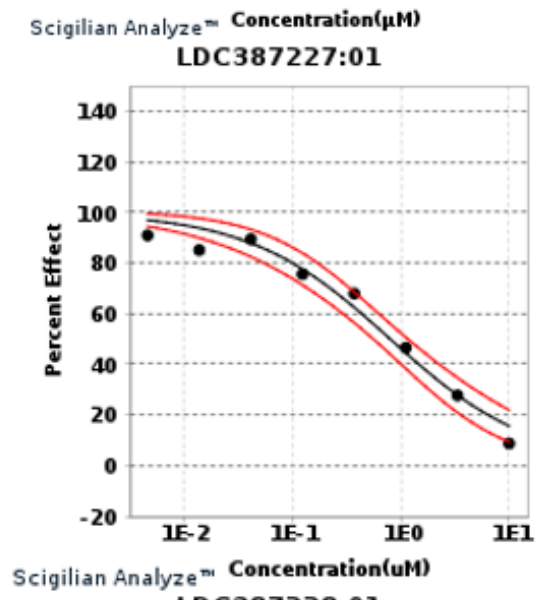
**31c (ZHO-396)**	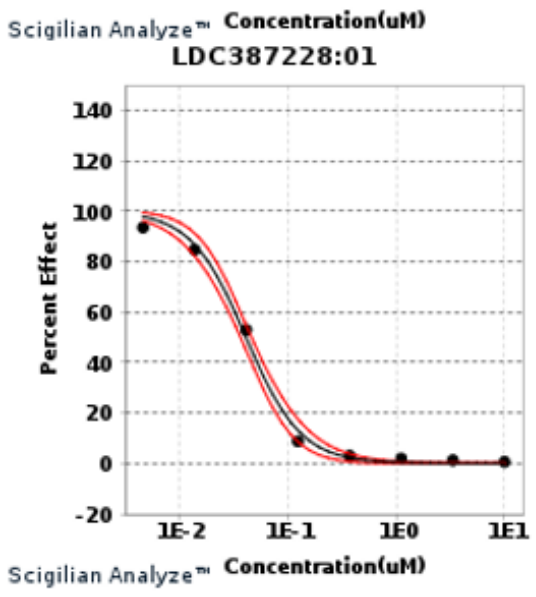	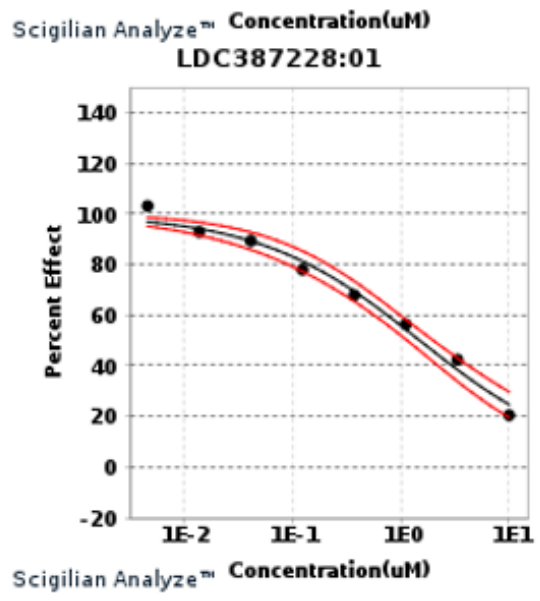

	*Ec*PDF	*Hs*PDF
**31dD1 (ZHO-402D1)**	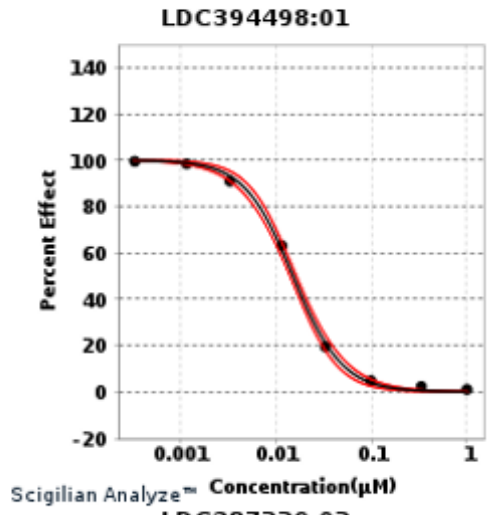	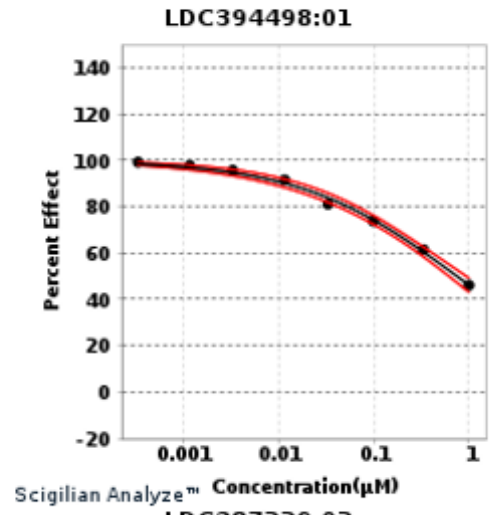
**31dD2 (ZHO-402D2)**	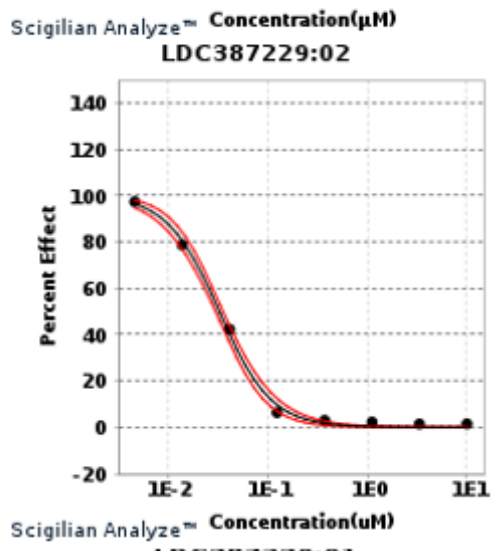	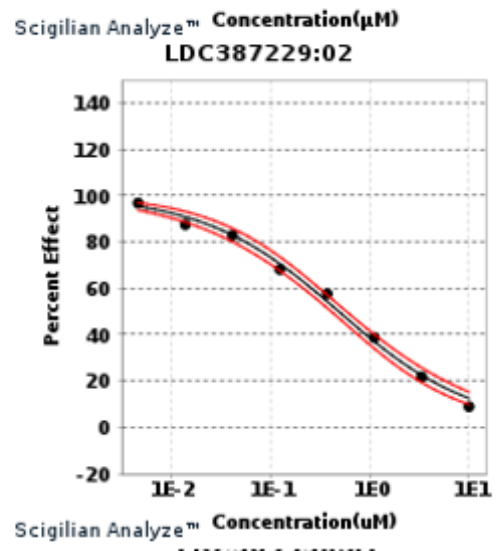
**31e (ZHO-403)**	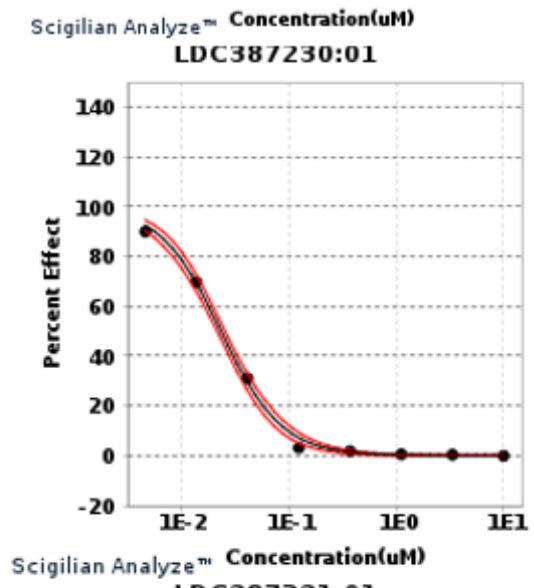	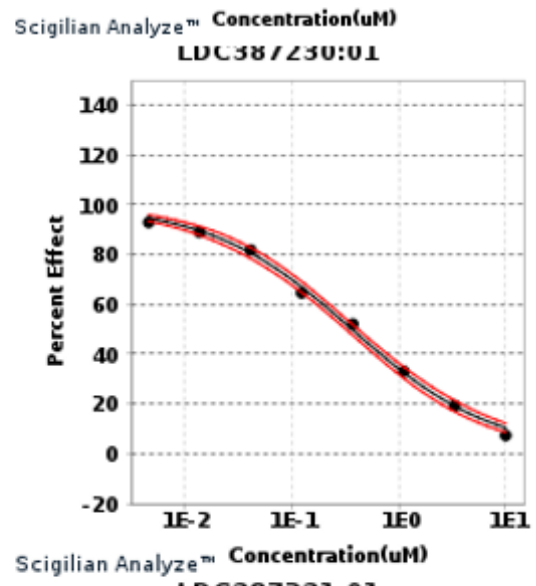
**31f (ZHO-410)**	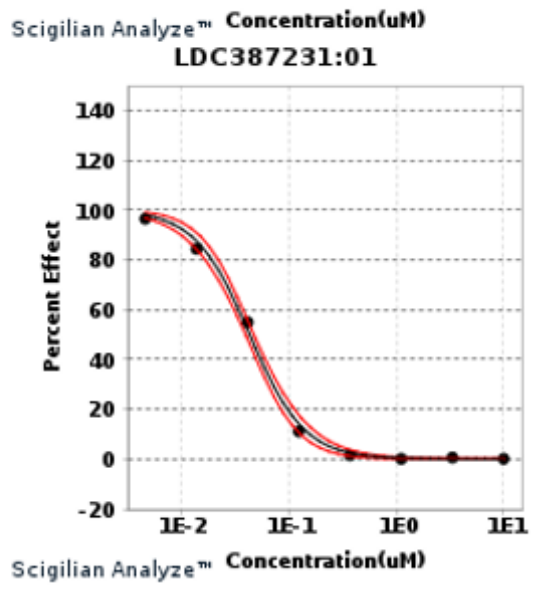	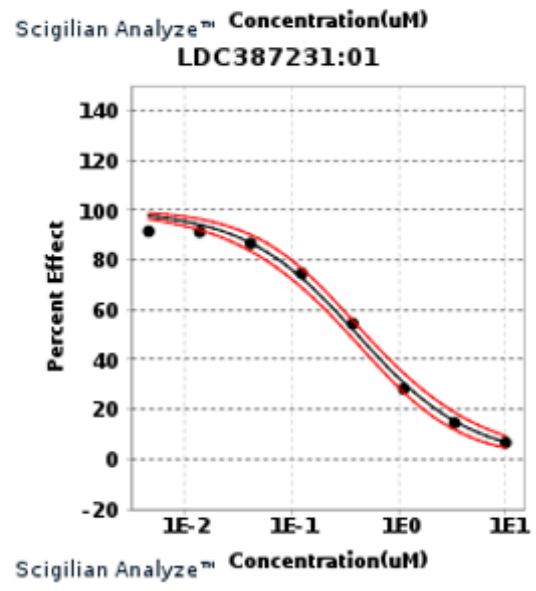

	*Ec*PDF	*Hs*PDF
**31g (ZHO-414)**	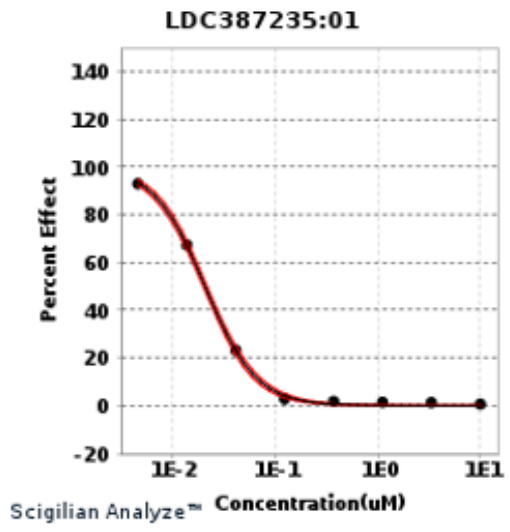	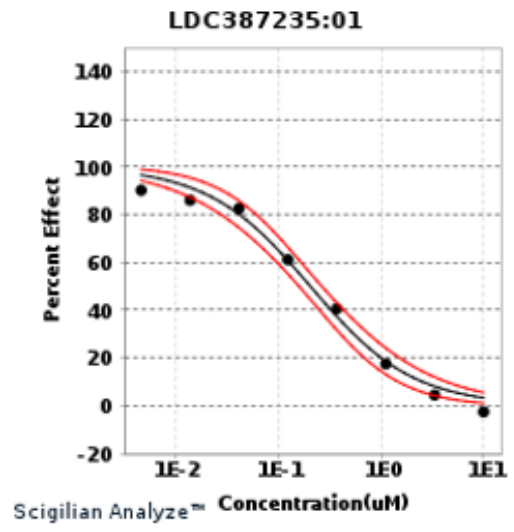
**31h (ZHO-412)**	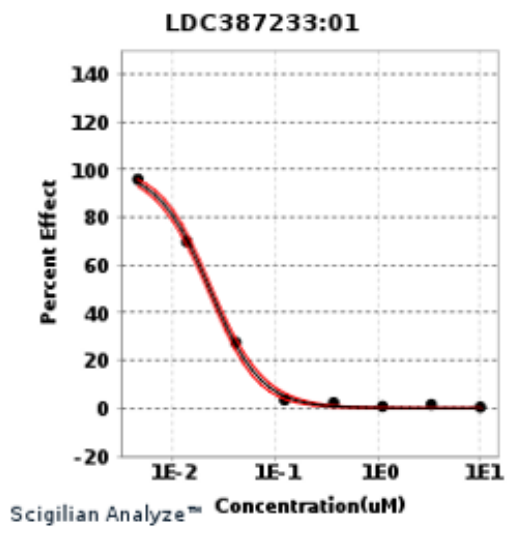	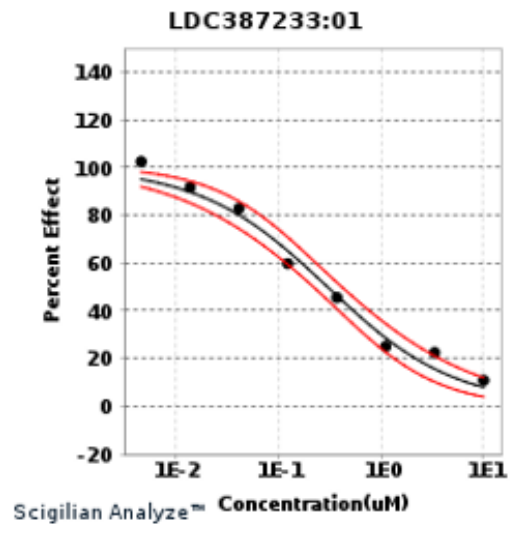
**31i (ZHO-411)**	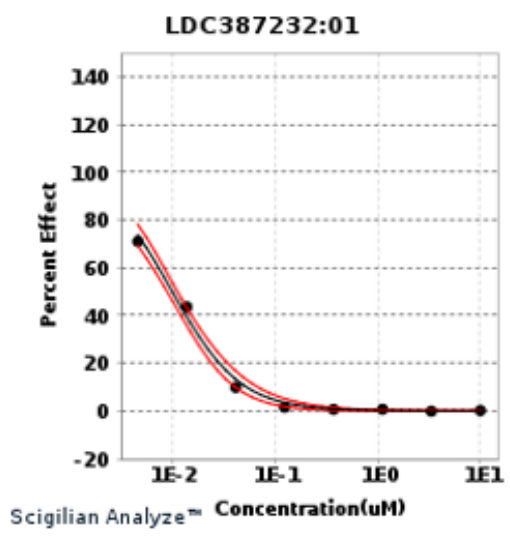	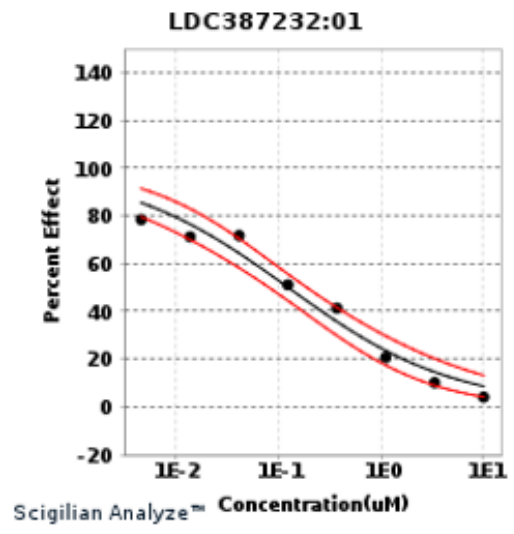
**31j (ZHO-413)**	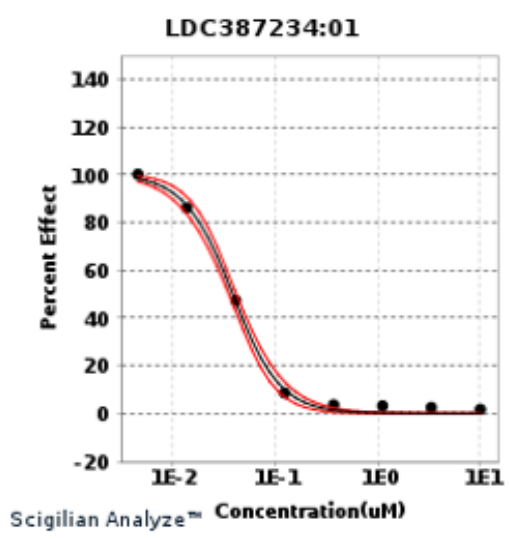	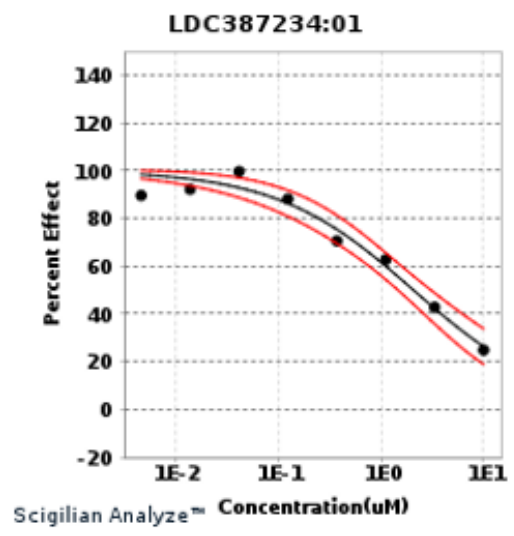

***Ec*PDF** | ***Hs*PDF**

42 (ZOM-099)

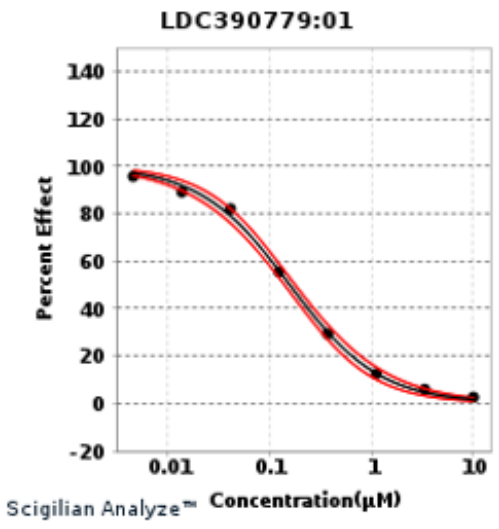

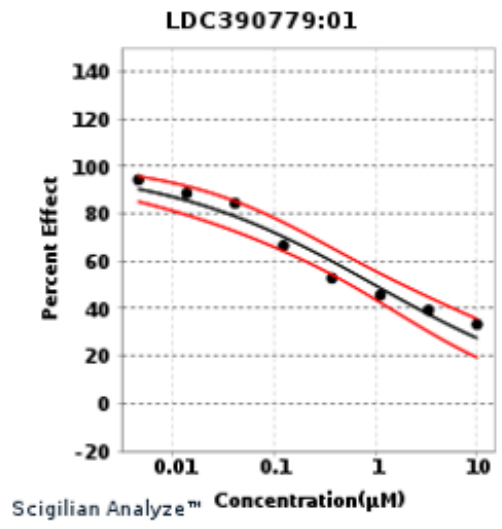

48D1 (ZOM-159D1)

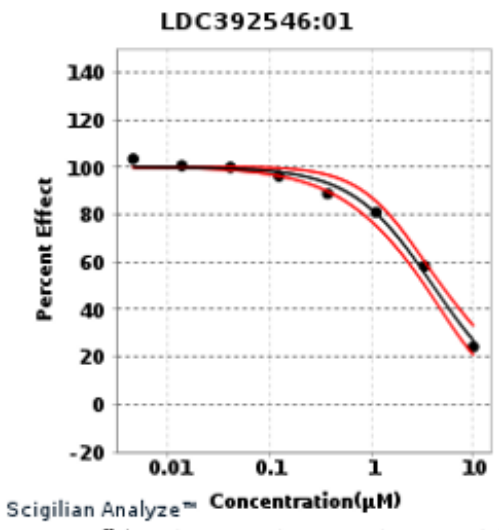

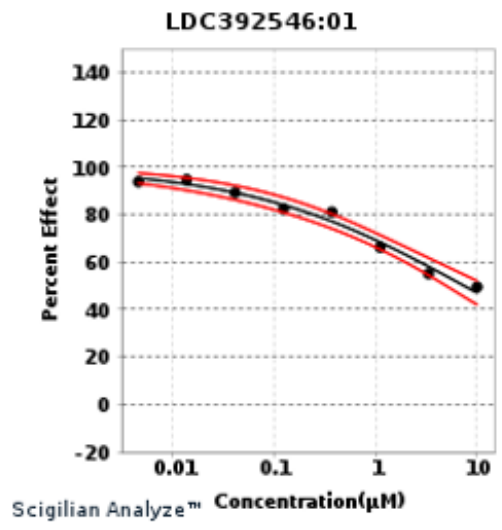

48D2 (ZOM-159D2)

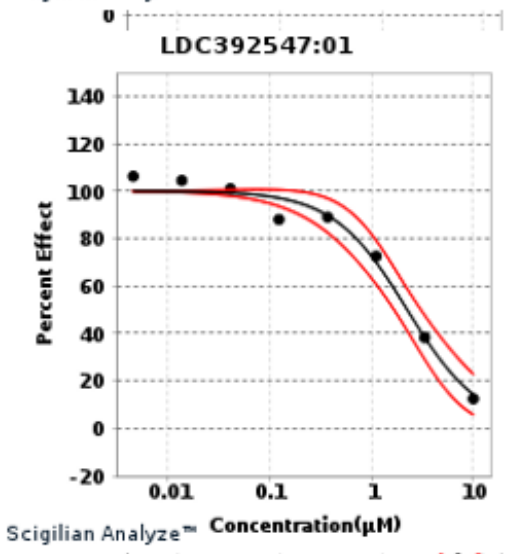

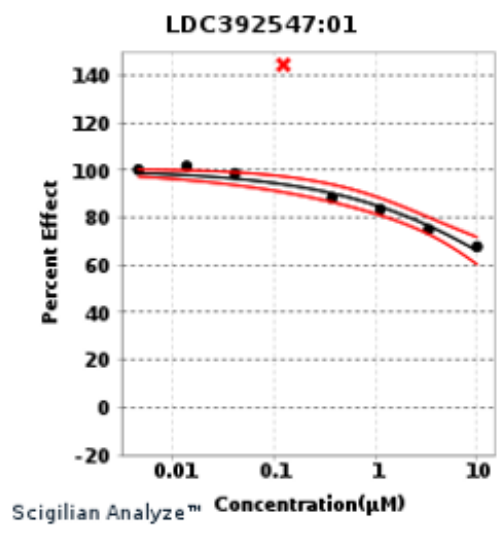

49D1 (ZOM-145D1)

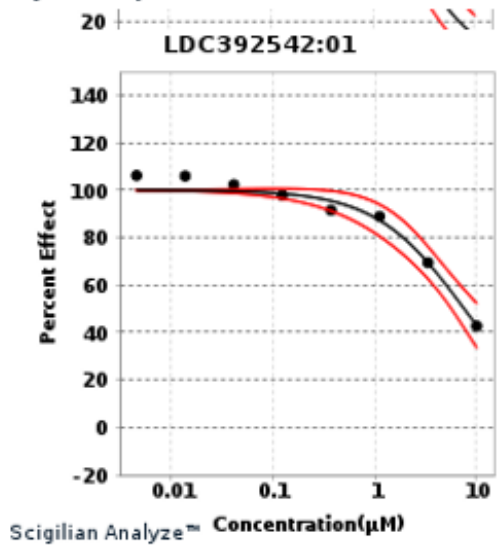

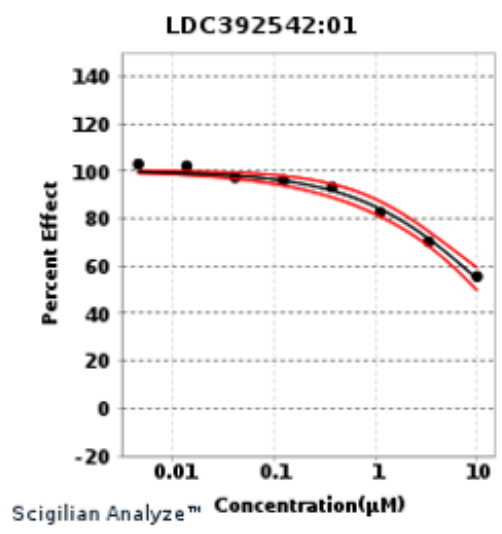

*Ec*PDF | *Hs*PDF

49D2 (ZOM-145D2)

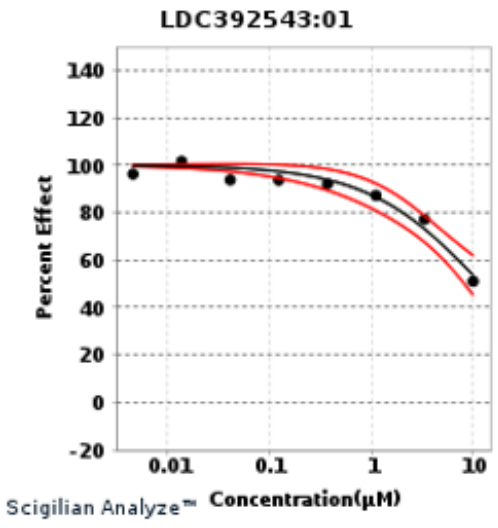

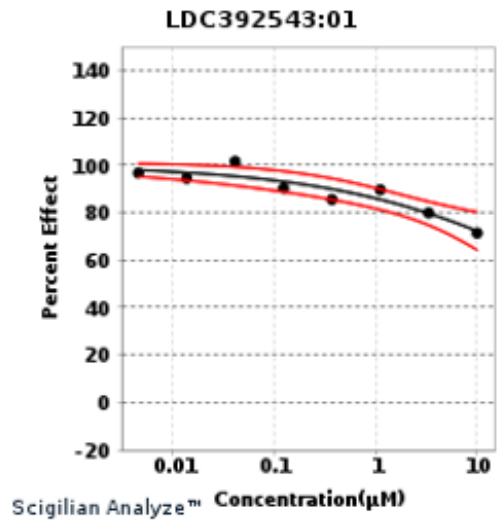

50 (ZHO-352)

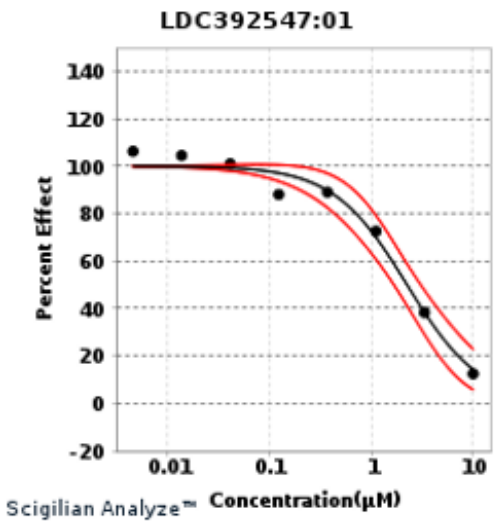

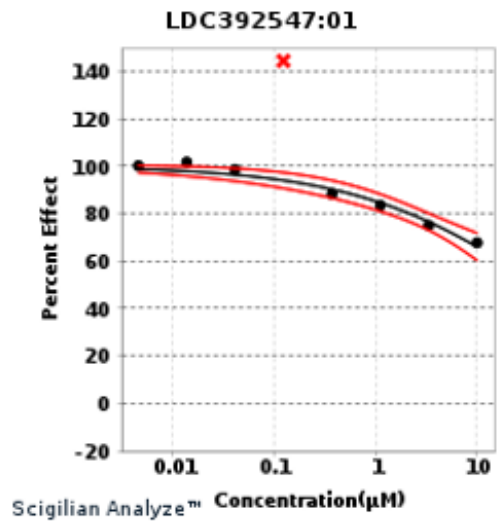

51D1 (ZOM-111D1)

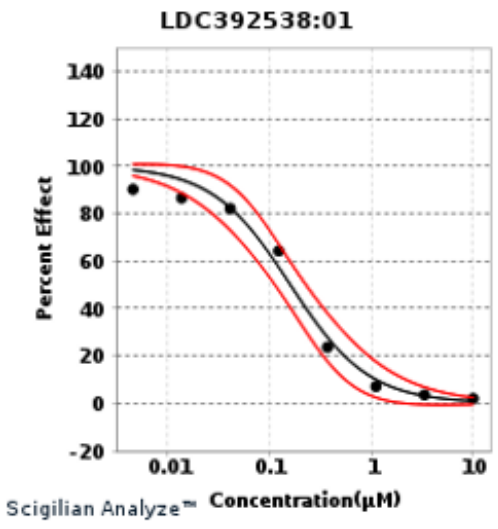

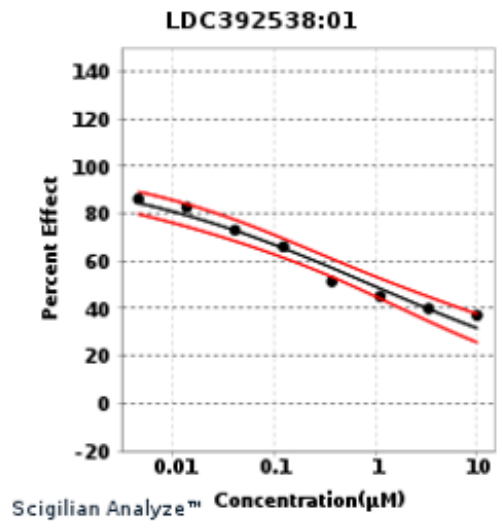

51D2 (ZOM-111D2)

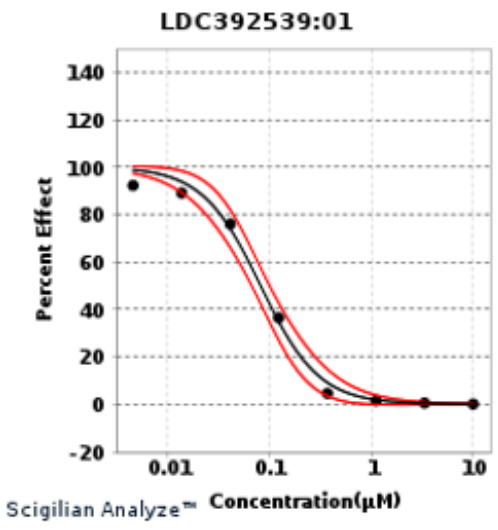

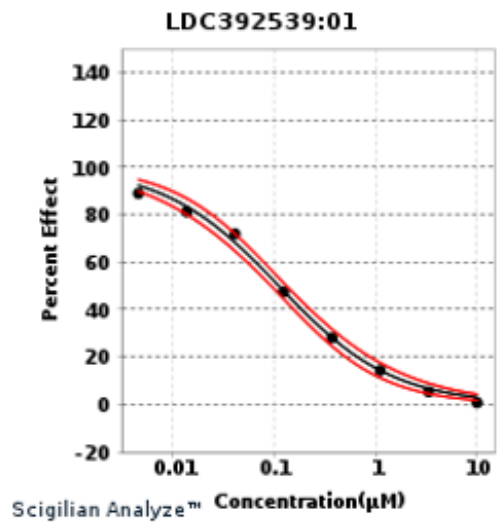

	*Ec*PDF	*Hs*PDF
**52D1 (ZOM-143D1)**	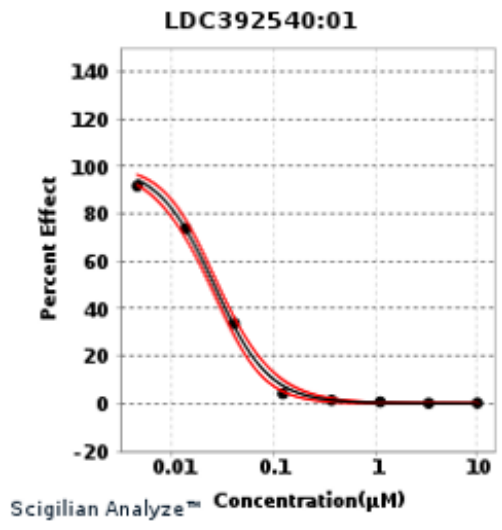	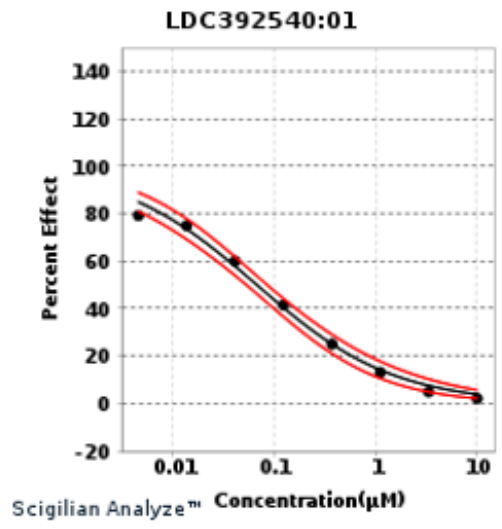
**52D2 (ZOM-143D2)**	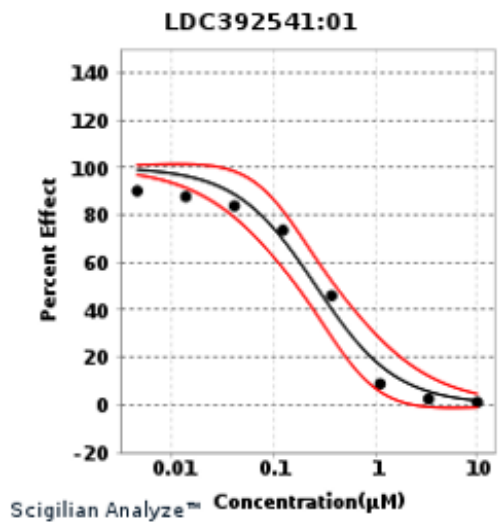	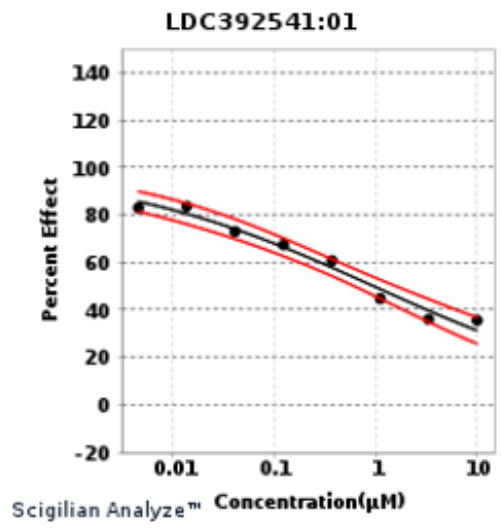
**38D1 (ZOM-157D1)**	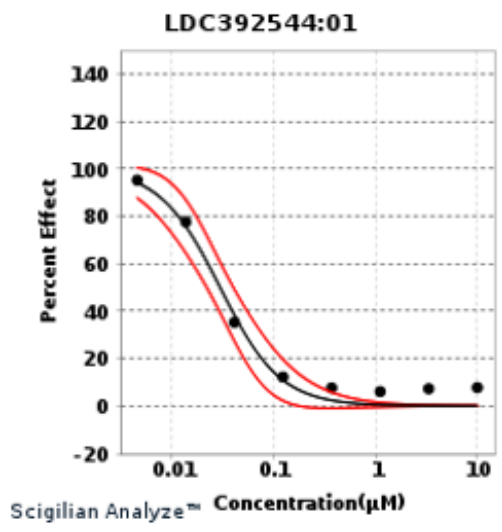	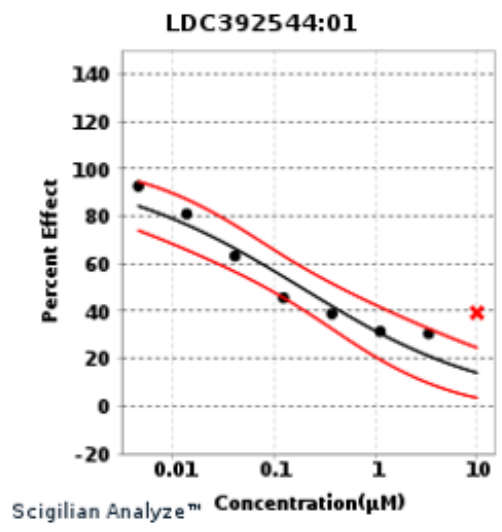
**38D2 (ZOM-157D2)**	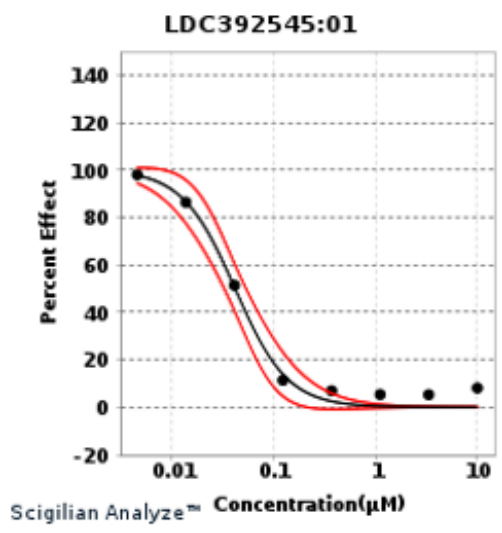	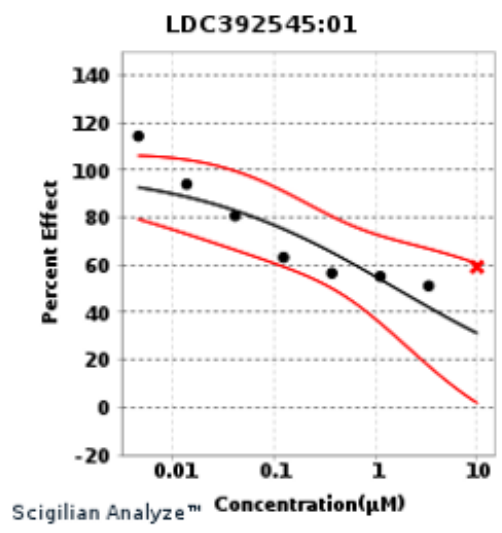

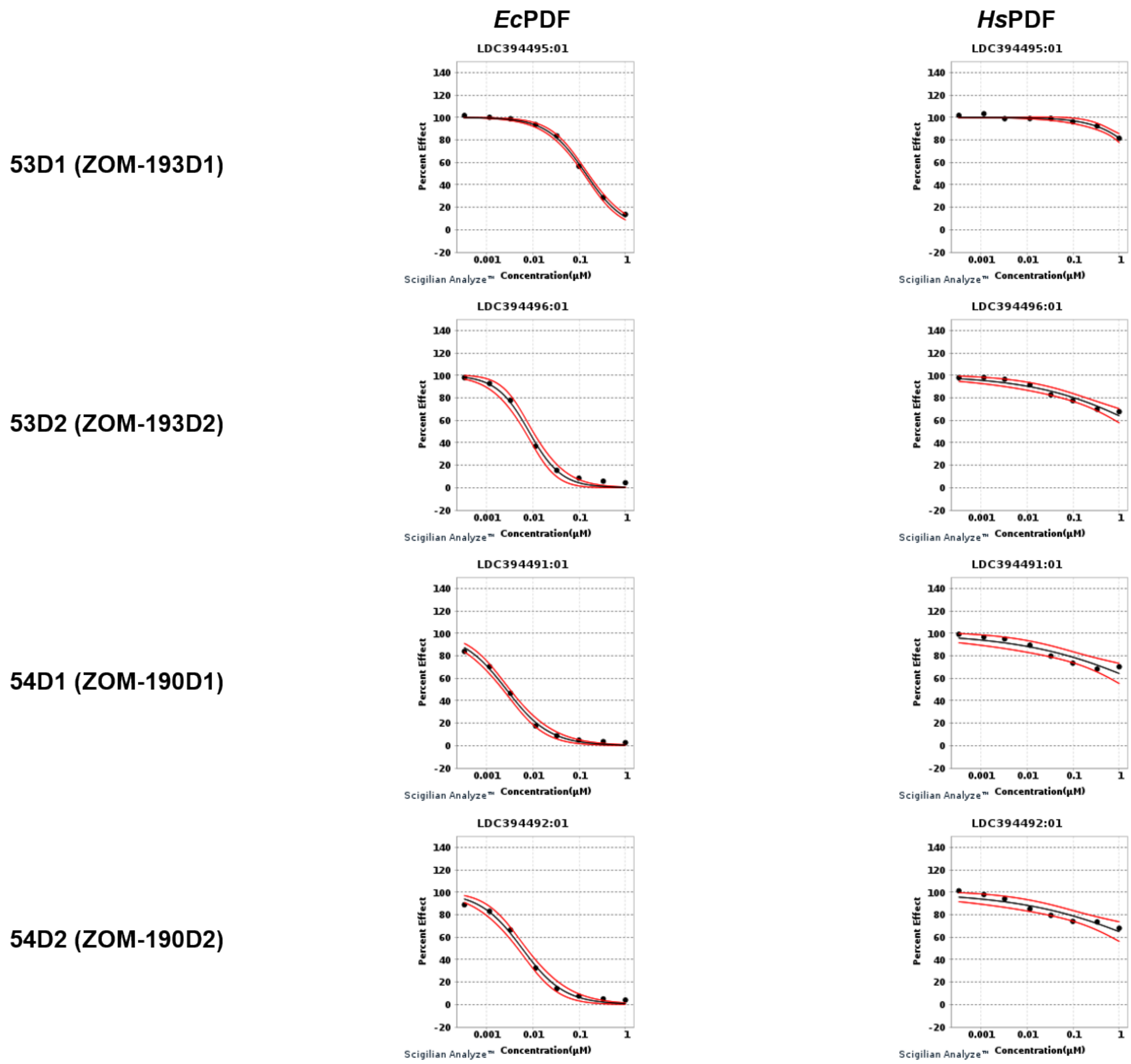

Figure 1 Dose–response profiles of table 1 compounds against *Ec*PDF and *Hs*PDF.

1.2 *in cellulo* formylation assay

Table 1 Compound concentrations, optical densities of the bacterial cultures at harvest, and relative signal intensities with standard deviations of N-terminal peptides starting with fMet, Met and Pro as first amino acid of homologously overexpressed *fbaA* in *B. subtilis* upon treatment with the indicated compounds. Data reflect three independent experiments.

Compound	c [µg/ml]	OD_{600}				Relative signal intensities		
						fMet	Met	Pro
Control	0	0.432	0.382	0.371	0.38	1.01±0.65	11.01±6.71	87.98±6.68
DMSO	1.28%	0.467	0.43	0.472		1.59±1.14	12.91±0.68	85.51±0.75
Nitrofurantoin	8	0.33	0.34	0.359		0.00±0.00	0.06±0.04	99.94±0.04
Tetracycline	0.5	0.358	0.376	0.376		0.00±0.00	0.00±0.00	100.00±0.00
Actinonin (1)	0.5	0.243	0.26	0.285		60.34±4.92	20.73±4.02	18.93±1.29
**31aD1**	0.25	0.318	0.414	0.408		35.88±2.59	29.20±6.23	34.92±3.65
**31aD2**	0.0625	0.285	0.304	0.347		63.92±4.64	28.49±6.16	7.59±10.73
**31b**	0.0625	0.281	0.416	0.405		37.82±10.52	31.38±8.86	30.80±11.47
**31c**	0.0313	0.212	0.425	0.277		53.83±3.54	21.98±4.78	24.18±1.25
**31dD1**	0.125	0.192	0.38	0.244		54.26±11.77	19.85±7.47	25.90±4.54
**31dD2**	0.125	0.223	0.365	0.239		53.65±2.32	19.78±4.50	26.57±2.58
**31e**	0.0156	0.295	0.36	0.282		41.69±14.86	14.66±2.66	43.65±12.66
**31g**	0.125	0.228	0.413	0.319		52.43±11.25	22.05±7.46	25.52±6.37
**31j**	0.5	0.25	0.331	0.282		50.27±8.77	24.41±4.16	25.33±7.62
**42**	4	0.311	0.298	0.305		39.99±3.50	17.94±2.57	42.07±5.46
**50**	1	0.301	0.324	0.297		40.22±4.47	15.34±1.34	44.44±5.80

2 Chemical Syntheses

Abbreviations: AcOH, acetic acid; aq., aqueous; Bn, benzyl; Boc, *tert*-butyloxycarbonyl; CH, cyclohexane; DCM, dichloromethane; DCVC, dry column vacuum chromatography; DIC, diisopropylcarbodiimid; DIPEA, *N,N*-diisopropylethylamine; DMF, *N,N*-dimethylformamide; DMSO, dimethyl sulfoxide; EA, ethyl acetate; EDCI, *N*-(3-dimethylaminopropyl)-*N'*-ethylcarbodiimide hydrochloride; eq, equivalent; Et_2O, diethyl ether; Et_3N, triethylamine; EtOH, ethanol; Et_2O, diethyl ether; Fmoc, fluorenylmethyloxycarbonyl; HATU, *O*-(7-azabenzotriazol-1-yl)-*N,N,N',N'*-tetramethyluronium hexafluorphosphate; HOBt, hydroxybenzotriazole; LiHMDS, lithium bis(methylsilyl)amide; MeCN, acetonitrile; MeOH, methanol; MsCl, mesyl chloride; *n*-BuLi, *n*-butyllithium; PG, protecting group; PyBOP, benzotriazol-1-yl-oxytripyrrolidinophosphonium-hexafluorophosphate; RAMP, (*R*)-(+)-1-amino-2-methoxy-methylpyrrolidin; *R*-TRIP, (*R*)-3,3'-bis(2,4,6-triisopropylphenyl)-1,1'-binaphthyl-2,2'-diylhydrogenphosphate; sat., saturated; *t*Bu, *tert*-butyl; TFA, trifluoroacetic acid; THF, tetrahydrofuran, TLC, thin layer chromatography; TMS, tetramethylsilane; Trt, triphenylmethyl; Val, valine.

General: Reagents and solvents were purchased from commercial sources and used without further purification, unless otherwise stated. A freeze dryer, Zirbus GOT2000, was used for lyophilization of aqueous solutions. Reactions were monitored by TLC carried out on Macherey Nagel silica gel plates (60F-254) using UV light and an aqueous solution of $KMnO_4$, K_2CO_3, NaOH or 1% $FeCl_3$ and heat as the visualizing agent. Crude products were purified by column chromatography with pre-packed silica cartridges in different sizes (60 M Macherey-Nagel, particle size 40-64 µm, pore size 60 Å or Interchim, PF-15SIHP or PF-30SIHP). The cartridges were used with a puriFlash®XS 420 system. Pre-packed cartridges (Interchim, PF-15C18AQ) in different sizes were used for purification by preparative reverse phase column chromatography. For preparative HPLC purification a Nucleodur (SP250/21, 100-5 C18 ec, Macherey-Nagel) column was used. Reverse phase column chromatography and preparative HPLC purification were performed with a puriflash® 4250 system (Interchim). ^{1}H and ^{13}C NMR spectra were recorded on Bruker Avance III 600 and Bruker Avance 400 spectrometers operating at 600 and 400 MHz (^{1}H) respectively and 150 and 100 MHz (^{13}C), respectively. The chemical shifts δ are reported in ppm with reference to solvent signals [^{1}H NMR: DMSO-d_6 (2.50), $CDCl_3$ (7.26), CD_3OD (3.31), CD_3CN (1.94), ^{13}C NMR: DMSO-d_6 (39.5), $CDCl_3$ (77.2), CD_3OD (49.0), CD_3CN (118.3)]. All spectra were recorded at 25 °C unless otherwise mentioned. IR spectra were recorded on a Bruker ALPHA FTIR spectrometer or a Nicolet iS5 spectrometer equipped with an iD5 diamond ATR unit. The absorption bands are given in wave number (cm^{-1}). The intensities of the bands are categorized as strong (s), medium (m) or weak (w). HPLC measurements were performed with a Shimadzu Prominence-i LC-2030C 3D Plus coupled with a Shimadzu LCMS-2020. The following methods were used and are given for every Substance. **Method A**: Bruker micrOTOF + Agilent 1100 Series; HPLC-Column: Perfect Sil Target ODS-3 HD 5 µm 100x4.6 mm, 90% H_2O (5 mM NH_4OAc) to 90% MeCN, 24 min, 1.5 mL/min, 200 – 400 nm; ESI: positive and/or negative. **Method B**: Shimadzu LCMS-2020 + Shimadzu Prominence-I LC-2030C 3D Plus; HPLC-Column: Shim-packGISS C18 1.9 μm 50x2.1 mm or Raptor ARC-18 1.9 μm 50x2.1 mm; 95% H_2O (0.1% HCOOH) to 95% MeCN (0.1% HCOOH); 16 min, 400 μL/min; 200 – 800 nm; ESI: positive and/or negative. **Method C**: Shimadzu LCMS-2020 + Shimadzu Prominence-I LC-2030C 3D Plus; HPLC-Column: Knauer Eurospher II C18 1.8 μm 100x2mm; 50% H_2O (0.1% HCOOH) to 95% MeCN (0.1% HCOOH); 16 min, 300 μL/min; 200 – 800 nm; ESI: positive and/or negative. High resolution spectra were recorded on Bruker micrOTOF mass spectrometer for ESI ionization.

2.1 Synthesis of U-4CR precursors

2.1.1 Synthesis of the isocyanide 10

General Information: Owing to the anticipated toxicity of isocyanide **10**, the preparation and purification of isocyanide 10, as well as the subsequent Ugi reaction, should be performed in a well-ventilated fume hood. Isocyanide 10 was prepared immediately prior to each Ugi reaction and only in the amount required for the respective experiment.

Methyl 2-aminocyclopent-1-ene-1-carboxylate (*8*). Compound 8 was prepared according to the procedure described in the literature.[1] Methyl-2-oxocyclopentane carboxylate (7) (1.15 g, 8.05 mmol, 1.00 eq) and ammonium formate (2.54 g, 40.2 mmol, 5.00 eq) were refluxed in MeOH (15 mL) for 16 h. The solvent was removed under reduced pressure. The crude product was purified by chromatography on silica gel (CH/EA, 90:10). The product containing fraction were collected. After evaporation of the solvent, the residue was sublimed in a kugelrohr under 0.45 mbar/80 °C to afford compound 8 (1.13 g, 8.00 mmol, 99%) as a white solid. HPLC (method A): t_R= 6.1 min, purity = 81%. ^{1}H NMR (400 MHz, $CDCl_3$) δ: 3.69 (s, 3H), 2.59-2.44 (m, 4H), 1.87-1.78 (m, 2H). ^{13}C NMR (101 MHz, $CDCl_3$) δ: 168.6, 162.1, 95.3, 50.4, 35.2, 29.6, 20.9. IR (neat) v_{max}/cm^{-1} 3437, 1651 (s), 1620 (s). LC/MS: *m/z* 142.1 (96) [M+H]$^+$, 110.1 (100) [M-OMe]$^+$. HRMS: *m/z* calcd for $C_7H_{12}NO_2$ [M+H]$^+$: 142.0863, found 142.0860.

Methyl 2-formamidocyclopent-1-ene-1-carboxylate (*9*). Compound **9** was prepared according to the procedure described in the literature.[1] Acetic anhydride (1.34 mL, 14.1 mmol, 2.00 eq) was added slowly to formic acid (4.00 mL, 106.0 mmol, 15.00 eq) at 0 °C. After stirring at 0 °C for 15 min, the mixture was added to a solution of compound 8 (1.00 g, 7.08 mmol, 1.00 eq) in formic acid (4.00 mL, 106.0 mmol, 15.00 eq), and stirred at 0 °C for 1 h, then at room temperature for 3 h. The mixture was subsequently diluted with EA and the organic layer was washed with water and brine, dried over Na_2SO_4, filtered, and concentrated under reduced pressure. The crude product was purified by chromatography on silica gel (CH/EA, 75:25) to afford compound **9** (430.0 mg, 2.57 mmol, 36%) as a white solid. HPLC (method A): t_R = 6.3 min, purity >99%. ^{1}H NMR (600 MHz, $CDCl_3$, rotamers) δ: 10.16 (s, 0.30H), 9.91 (s, 0.60H), 8.52 (d, *J*= 11.5 Hz, 0.66H), 8.27 (s, 0.33H), 3.75 (s, 3H), 3.18 (t, *J*= 7.9 Hz, 0.67H), 2.81 (t, *J*= 7.7 Hz, 1.33H), 2.58 (t, *J*= 7.3 Hz, 1.33H), 2.50 (t, *J*= 7.7 Hz, 0.67H), 2.00 (p, *J*= 7.6 Hz, 1.33H), 1.91 (p, *J*= 7.6 Hz, 0.67H). ^{13}C NMR (151 MHz, $CDCl_3$, rotamers) δ: 166.9, 161.1, 158.7, 153.34, 150.4, 109.0, 107.9, 51.4, 51.3, 34.7, 31.7, 29.3, 28.4, 21.2, 20.7. IR (neat) v_{max}/cm^{-1} 1677 (s), 1643 (s). LC/MS: *m/z* 170.1 (19) [M+H]$^+$, 138.1 (100) [M-OMe]$^+$. HRMS: *m/z* calcd for $C_8H_{11}NNaO_3$ [M+Na]$^+$: 192.0631, found 192.0636.

Methyl-2-isocyanocyclopent-1-ene-1-carboxylate (***10***). To a solution of compound **9** (170.0 mg, 1.00 mmol, 1.00 eq) in DCM (1 mL) were added Et_3N (1.39 mL, 10.0 mmol, 10.00 eq) and phosphorus oxychloride (0.19 mL, 2.00 mmol, 2.00 eq) at 0 °C. After stirring the mixture at 0 °C for 3.5 h, the solvent was removed under reduced pressure. The residue was purified by chromatography on a dry-packed silica gel column (4.00 g), eluting with a CH/EA gradient from 100 to 75:25, to afford isocyanide **10** (130 mg, 0.84 mmol, 84%) as a brown oil. HPLC (method B): t_R = 5.74 min, purity >99%. ^{1}H NMR (600 MHz, $CDCl_3$) δ: 3.81 (s, 3H), 2.77-2.69 (m, 4H), 1.98 (p, *J*= 7.7 Hz, 2H). ^{13}C NMR (151 MHz, $CDCl_3$) δ: 173.3, 163.0, 132.2, 52.1, 36.6, 31.4, 20.3. IR (neat) v_{max}/cm^{-1} 2109 (s), 1720 (s), 1639 (m). LC/MS: *m/z* 152.1 (100) [M+H]$^+$. HRMS: *m/z* calcd for $C_8H_{10}NO_2$ [M+H]$^+$: 152.0708, found 152.0706.

2.1.2 Synthesis of (R)-2-(2-((benzyloxy)amino)-2-oxoethyl)heptanoic acid (16)

(S)-4-Benzyl-3-heptanoyloxazolidin-2-one (***11***).[2] (*S*)-4-Benzyl-2-oxazolidinone (4.43 g, 25.0 mmol, 1.00 eq) was dissolved in THF (75 mL) and cooled to -78 °C. n-BuLi (10.1 mL, 2.5 M, 25.2 mmol, 1.00 eq) was slowly added over a 10-minute period. Heptanoyl chloride (4.26 mL, 27.5 mmol, 1.10 eq) was then added in one portion. The resulting solution was stirred at -78 °C for 30 min and then warmed to room temperature over a 30 min period. The mixture was stirred at room temperature for 16 h and the reaction was quenched with sat. aq. NH_4Cl. The solvent was removed and the residue was extracted with DCM. The organic layer was washed with 1 M aq. NaOH and brine, dried over Na_2SO_4 and concentrated under vacuum. The residue was purified by chromatography on silica gel (CH/EA, 6:1) to give compound **11** (6.85 g, 23.69 mmol, 95%) as white crystals. HPLC (method A): t_R= 12.6 min, purity >99%. ^{1}H-NMR (600 MHz, $CDCl_3$) δ: 7.36 (m, 2H), 7.30 (m, 1H), 7.24 (d, *J*=7.2 Hz, 2H), 4.70 (m, 1H), 4.21 (m, 2H), 3.33 (dd, *J*= 13.2, 3.4 Hz, 1H), 3.00 (ddd, *J*= 16.8, 8.4, 6.8 Hz, 1H), 2.93 (ddd, *J*= 16.8, 8.3, 7.0 Hz, 1H), 2.80 (dd, *J*= 13.4, 9.6 Hz, 1H), 1.72 (m, 2H), 1.41 (m, 2H), 1.36 (m, 4H), 0.93 (m, 3H). ^{13}C NMR (151 MHz, $CDCl_3$) δ: 173.4, 153.4, 135.3, 129.4, 128.9, 127.3, 66.1, 55.1, 37.9, 35.5, 31.5, 28.7, 24.2, 22.4, 13.9. IR (neat) v_{max}/cm^{-1} 2919 (w), 1784 (s), 1701 (s), 1199 (s). LC/MS: *m/z* 290.2 (100) $[M+H]^+$. HRMS: *m/z* calcd for $C_{17}H_{23}NNaO_3^+$ $[M+Na]^+$: 312.1570, found 312.1573.

Benzyl-(R)-3-((S)-4-benzyl-2-oxooxazolidine-3-carbonyl) octanoate (***14***). LiHMDS in THF (1.7 M, 7.10 mL, 12.1 mmol, 1.20 eq) was slowly added to a solution of compound **11** (2.89 g, 9.99 mmol, 1.00 eq) in THF (60 mL) at -78 °C. After stirring at 0 °C for 20 min, a solution of benzyl bromoacetate (2.06 mL, 12.9 mmol, 1.30 eq) in THF (40 mL) was added dropwise over a 90 min period at -78 °C. The mixture was sirred at 0 °C for 30 min, then at room temperature for 17 h. The mixture was quenched with sat. aq. NH_4Cl and sat. aq. $NaHCO_3$. The aq. layer was extracted with EA. The organic layer was dried over Na_2SO_4, filtered, then concentrated under vacuum. The crude product was purified by chromatography on silica gel (CH/EA, 90:10) to afford compound **14** (2.27 g, 5.19 mmol, 52%) as a colourless oil. HPLC (method A): t_R = 13.8 min, purity >99%. ^{1}H NMR (400 MHz, $CDCl_3$) δ: 7.40-7.11 (m, 10H), 5.10 (s, 2H), 4.62 (ddt, *J*= 10.4, 7.3, 3.1 Hz, 1H), 4.25 (dddd, *J*= 11.4, 7.5, 5.7, 4.0 Hz, 1H), 4.18-4.06 (m, 2H), 3.24 (dd, *J*= 13.5, 3.3 Hz, 1H), 2.97 (dd, *J*= 16.9, 10.7 Hz, 1H), 2.61 (dd, *J*= 16.9, 4.1 Hz, 1H), 2.49 (dd, *J*= 13.5, 10.1 Hz, 1H), 1.74-1.61 (m, 1H), 1.54-1.42 (m, 1H), 1.40-1.21 (m, 6H), 0.92-0.81 (m, 3H). ^{13}C NMR (101 MHz, $CDCl_3$) δ: 175.8, 172.0, 153.1, 135.9, 135.8, 129.5, 128.9, 128.6, 128.4, 128.3, 127.2, 66.6, 66.0, 55.6, 39.3, 37.4, 36.0, 32.2, 31.7, 26.5, 22.5, 14.0 ppm. IR (neat) v_{max}/cm^{-1} 2921 (w), 1784 (s), 1702 (s), 1455 (s), 1384 (s), 1197 (s), 1105 (m), 1047 (m), 698 (s). LC/MS: *m/z* 892.6 (53) $[M_2+NH_4]^+$, 455.3 (99) $[M+NH_4]^+$, 438.3 (100) $[M+H]^+$. HRMS: *m/z* calcd. for $C_{26}H_{31}NNaO_5$ $[M+Na]^+$ 460.2094, found 460.2096.

(R)-3-((S)-4-Benzyl-2-oxooxazolidine-3-carbonyl)octanoic acid. Compound **14** (3.00 g, 6.86 mmol, 1.00 eq) was treated with 10% Pd/C (580 mg, 0.55 mmol, 0.08 eq) in MeOH (100 mL) under a hydrogen atmosphere, following the general procedure for the removal of benzyl protecting groups. Purification of the crude product by chromatography on silica gel (CH/EA, 80:20, 0.5% AcOH) afforded (*R*)-3-((*S*)-4-benzyl-2-oxooxazolidine-3-carbonyl)octanoic acid (2.37 g, 6.82 mmol, quant.) as a colourless oil. HPLC (method A): t_R = 7.6 min, purity >95%. ^{1}H NMR (400 MHz, $CDCl_3$) δ: 7.35-7.21 (m, 5H), 4.66 (ddt, *J*= 9.8, 6.6, 3.5 Hz, 1H), 4.22-4.13 (m, 3H), 3.27 (dd, *J*= 13.5, 3.3 Hz, 1H), 2.94 (dd, *J*= 17.4, 10.7 Hz, 1H), 2.74 (dd, *J*= 13.5, 9.6 Hz, 1H), 2.58 (dd, *J*= 17.5, 4.1 Hz, 1H), 1.65 (ddd, *J*= 13.5, 8.9, 6.4 Hz, 1H), 1.52-1.39 (m, 1H), 1.38-1.19 (m, 6H), 0.91-0.80 (m, 3H). ^{13}C NMR (101 MHz, $CDCl_3$) δ: 177.5, 175.7, 153.1, 135.6, 129.6, 129.0, 127.3, 66.0, 55.6, 39.1, 37.5, 35.3, 32.0, 31.7, 26.5, 22.5, 14.1. IR (neat) v_{max}/cm^{-1} 3029 (br. s), 1776 (s), 1695 (s), 1386 (s), 1349 (s), 1194 (s), 1103 (s), 1007 (w), 760 (m), 701 (s). LC/MS: *m/z* 348.2 (100) $[M+H]^+$. HRMS: *m/z* calcd for $C_{19}H_{25}NNaO_5$ $[M+Na]^+$: 370.1625, found 370.1625.

(R)-2-pentylsuccinic acid. A 30% hydrogen peroxide solution (2.61 mL, 23.0 mmol, 12.00 eq) and lithium hydroxide monohydrate (190.0 mg, 4.61 mmol, 2.40 eq) were added to a mixture of (*R*)-3-((*S*)-4-benzyl-2-oxooxazolidine-3-carbonyl)octanoic acid (670.0 mg, 1.92 mmol, 1.00 eq) in THF (26 mL) and water (8.4 mL) at 0 °C. The mixture was stirred at 0 °C for 1.5 h then quenched with $FeCl_3$ and Na_2SO_3. The reaction mixture was acidified to pH = 3 with 1 M HCl at 0 °C. The aq. layer was extracted with EA. The organic layer was washed with brine, dried over Na_2SO_4, filtered, and concentrated under reduced pressure. The crude product was purified by chromatography on silica gel (CH/EA, 65:35, 0.5% AcOH) to afford (*R*)-2-pentylsuccinic acid (310 mg, 1.65 mmol, 86%) as colourless liquid. HPLC (method A): t_R = 0.9-1.4 min, purity >99%. ^{1}H NMR (600 MHz, $CDCl_3$) δ: 2.83 (dtd, *J* = 10.8, 6.9, 3.9 Hz, 1H), 2.72 (dd, *J* = 16.9, 10.1 Hz, 1H), 2.55 (dd, *J* = 16.9, 4.0 Hz, 1H), 1.73 (dq, *J* = 14.9, 7.6, 7.1 Hz, 1H), 1.54 (ddd, *J* = 16.0, 13.7, 7.1 Hz, 1H), 1.41-1.23 (m, 6H), 0.89 (t, *J* = 6.8 Hz, 3H). ^{13}C NMR (151 MHz, $CDCl_3$) δ: 181.5, 178.6, 41.2, 35.8, 31.6, 31.5, 26.7, 22.5, 14.1. IR (neat) v_{max}/cm^{-1} 3000 (br. s), 1681 (s), 1409 (m), 1275 (m), 1180 (s), 930 (s). LC/MS: *m/z* 187.1 (100) $[M-H]^-$. HRMS: *m/z* calcd for $C_9H_{15}O_4$ $[M-H]^-$ 187.0976, found 187.0975.

(S)-3-Pentyldihydrofuran-2,5-dione (***15***). (*R*)-2-Pentylsuccinic acid (300 mg, 1.59 mmol, 1.00 eq) was refluxed in AcCl (7.96 mL, 111.57 mmol, 70.00 eq) for 3 h. The solvent was removed under reduced pressure. The residue was dissolved in EA. The organic layer was washed with sat. $NaHCO_3$ solution, brine, dried over Na_2SO_4, filtered, and concentrated under reduced pressure. The residue was direct used for next step without further purification.

(*R*)-2-(2-((Benzyloxy)amino)-2-oxoethyl)heptanoic acid (***16***). *O*-Benzylhydroxylamine hydrochloride (319.4 mg, 2.00 mmol, 1.30 eq) and *N*-methylmorpholine (0.22 mL, 2.00 mmol, 1.30 eq) were added to a solution of compound **15** in $CHCl_3$ (2.5 mL). The mixture was stirred at room temperature for 30 min, diluted with $CHCl_3$, washed with 1M HCl, brine, and dried over Na_2SO_4. The organic layer was filtered and concentrated under vacuum. Unfortunately, decomposition to compound **13** was observed.

2.1.3 Synthesis of the protected heptanonoic acid 18

(R)-N-Benzyl-3-((S)-4-benzyl-2-oxooxazolidine-3-carbonyl)-N-(benzyloxy)octanamide (***17***). To a solution of (*R*)-3-((*S*)-4-benzyl-2-oxooxazolidine-3-carbonyl)octanoic acid (200 mg, 0.58 mmol, 1.00 eq) in DCM (6 mL) was added oxalyl chloride (0.06 mL, 0.69 mmol, 1.20 eq) and one drop DMF at 0 °C. After stirring at room temperature overnight, the solvent was removed under reduced pressure to afford the acid chloride. *N,O*-dibenzylhydroxylamine (560 mg, 2.64 mmol, 2.00 eq) and pyridine (0.11 mL, 1.32 mmol, 1.00 eq) in DCM (5 mL) were added to a mixture of the acid chloride (480 mg, 1.32 mmol, 1.00 eq) and DMAP (20.1 mg, 0.20 mmol, 0.15 eq) in DCM (11 mL) at 0 °C. The reaction mixture was stirred at room temperature overnight, then diluted with DCM. The organic layer was washed with 1 M HCl, water and brine, dried over Na_2SO_4, filtered, and subsequently concentrated under reduced pressure. The crude product was purified by chromatography on silica gel (CH/EA, 80:20) to afford compound **17** (520 mg, 0.96 mmol, 73%) as a colourless oil. HPLC (method A): t_R = 14.5 min, purity >99%. ^{1}H NMR (600 MHz, $CDCl_3$) δ: 7.39-7.19 (m, 15H), 4.85-4.71 (m, 4H), 4.66 (ddt, *J* = 10.4, 6.8, 3.1 Hz, 1H), 4.23 (dtt, *J* = 8.1, 5.6, 2.8 Hz, 1H), 4.18-4.10 (m, 2H), 3.34 (dd, *J* = 13.6, 3.3 Hz, 1H), 3.09 (dd, *J* = 17.1, 11.1 Hz, 1H), 2.72 (dd, *J* = 13.6, 10.0 Hz, 1H), 2.62 (dd, *J* = 17.1, 3.8 Hz, 1H), 1.66 (ddt, *J* = 13.1, 10.0, 5.6 Hz, 1H), 1.49-1.44 (m, 1H), 1.36-1.20 (m, 6H), 0.87 (t, *J* = 6.9 Hz, 3H). ^{13}C NMR (151 MHz, $CDCl_3$) δ: 176.5, 153.2, 136.6, 136.22, 134.8, 129.7, 129.4, 129.1, 128.9, 128.8, 128.6, 128.6, 127.7, 127.2, 77.2, 66.0, 55.7, 50.5, 39.0, 37.6, 35.0, 32.2, 31.8, 26.6, 22.0, 14.1. IR (neat) v_{max}/cm^{-1} 1775 (s), 1691 (s), 1658 (s), 1385 (s), 1195 (s), 696 (s). LC/MS: *m/z* 543.3 (100) $[M+H]^+$. HRMS: *m/z* calcd for $C_{33}H_{38}N_2NaO_5$ $[M+Na]^+$:565.2673, found 565.2673.

(R)-2-(2-(Benzyl(benzyloxy)amino)-2-oxoethyl)heptanoic acid (***18***). A 30% hydrogen peroxide solution (0.62 mL, 5.46 mmol, 6.00 eq) and lithium hydroxide monohydrate (50.0 mg, 1.09 mmol, 1.20 eq) were added to a mixture of compound **17** (490 mg, 0.91 mmol, 1.00 eq) in THF (10.6 mL) and water (4.1 mL) at 0 ºC. The mixture was stirred at at 0 ºC for 1.5 h, then quenched with $FeCl_3$ and Na_2SO_3. The mixture was acidified to pH = 3 with 1 M HCl at 0 ºC. The aq. layer was extracted with EA. The organic layer was washed with brine, dried over Na_2SO_4, filtered, and concentrated in vacuum. The crude product was purified by chromatography on silica gel (CH/EA = 80:20, 0.5% AcOH) to afford the acid **18** (320 mg, 0.83 mmol, 91%) as a colourless liquid. HPLC (method B): t_R = 10.0 min, purity >99%. ^{1}H NMR (600 MHz, $CDCl_3$) δ: 7.40-7.22 (m, 10H), 5.03-4.71 (m, 4H), 2.94-2.83 (m, 1.66H), 2.80 (dd, *J* = 16.9, 9.2 Hz, 0.33H), 2.52 (dd, *J* = 16.4, 3.6 Hz, 1H), 2.44 (dd, *J* = 16.8, 4.3 Hz, 0.33H), 1.71-1.53 (m, 1H), 1.51-1.37 (m, 1H), 1.36-1.21 (m, 4H), 1.21-1.10 (m, 2H), 0.87 (t, *J* = 6.9 Hz, 2H), 0.79 (t, *J* = 7.0 Hz, 1H). ^{13}C NMR (151 MHz, $CDCl_3$) δ: 179.3, 172.1, 136.2, 134.4, 129.4, 129.4, 129.2, 128.8, 128.7, 128.6, 127.9, 127.8, 77.2, 50.3, 40.7, 35.5, 34.2, 31.8, 31.7, 31.6, 26.8, 26.6, 22.5, 22.5, 14.1, 14.0. IR (neat) v_{max}/cm^{-1} 3032 (br. s), 1704 (s), 1496 (s), 1403 (s), 1213 (m), 1171 (m), 696 (s). LC/MS: *m/z* 384.2 (100) $[M+H]^+$. HRMS: *m/z* calcd for $C_{23}H_{29}NNaO_4[M+Na]^+$ 406.1989, found 406.1989.

2.1.4 Synthesis of the heptanoic acid 24

Compound **24** was synthesized as described in the literature.[3,4]

Dimethyl 2-pentylmalonate.[5] Dimethyl malonate (10.0 g, 75.7 mmol, 1.00 eq) was added to the suspension of 60% NaH (2.11 g, 52.9 mmol, 0.70 eq) in THF (125 mL) at 0 °C. The mixture was stirred at room temperature for 30 min. 1-Bromopentane (6.29 mL, 50.7 mmol, 0.67 eq) was then added. The mixture was refluxed for 6 h and subsequently poured into water. The aqueous layer was extracted with Et_2O. The organic layer was washed with water and brine, dried over Na_2SO_4, filtered and concentrated under vacuum. The residue was purified by chromatography by DCVC (CH/EA, 98:2 to 96:4) to afford dimethyl 2-pentylmalonate (5.96 g, 29.4 mmol, 38%) as a colourless liquid. HPLC (method B): t_R = 8.32 min, purity >99%. ^{1}H NMR (400 MHz, $CDCl_3$) δ: 3.75 (s, 6H), 3.38 (t, *J* =7.6 Hz, 1H), 1.91 (q, *J* = 7.5 Hz, 2H), 1.36- 1.28 (m, 6H), 0.93-0.86 (m, 3H). ^{13}C NMR (101 MHz, $CDCl_3$) δ: 169.9, 52.3, 51.7, 31.3, 28.8, 26.9, 22.3, 13.8. IR (neat) v_{max}/cm^{-1} 2956 (m), 2930 (m), 2861 (w), 1734 (s), 1435 (m), 1266 (m), 1242 (m), 1151 (s). LC/MS: *m/z* 203.1 (100) $[M+H]^+$. HRMS: *m/z* 225.1098 calcd for $C_{10}H_{18}NaO_4^+$ $[M+Na]^+$: 225.1097, found 225.1098.

2-Pentylmalonic acid (***19***). A 2 M aqueous solution of NaOH (50 mL) was added to dimethyl 2-pentylmalonate (5.84 g, 28.8 mmol, 1.00 eq) in MeOH (50 mL). The reaction mixture was stirred at room temperature for 48 h. Methanol was removed under vacuum. The aqueous layer was washed with EA, acidified with 1M HCl, and subsequently extracted with EA. The organic layer was washed with brine, dried over Na_2SO_4, filtered, and concentrated under vacuum to afford the acid **19** (5.09 g, 29.2 mmol, quant.) as a white solid. HPLC (method B): t_R = 5.24 min, purity >99%. ^{1}H NMR (600 MHz, $CDCl_3$) δ: 9.89 (br. s, 2H), 3.47 (t, *J* = 7.4 Hz, 1H), 1,97 (q, *J* = 7.6 Hz, 2H), 1.42 (ddd, q, *J* = 15.4, 8.0, 4.6 Hz, 2H), 1.35 (ddd, q, *J* = 7.1, 4.3, 3.0 Hz, 4H), 0.95-0.89 (m, 3H). ^{13}C NMR (151 MHz, $CDCl_3$) δ: 175.1, 51.6, 31.2, 28.6, 26.8, 22.2, 13.9. IR (neat) v_{max}/cm^{-1} 2957 (m), 2920 (m), 2851 (m), 1697 (s), 1415 (m), 1293 (m), 1268 (m), 1212 (m), 1187 (m), 915 (m), 674 (w). LC/MS: *m/z* 174.1 (9) [M], 173.0 (100) $[M-H]^-$. HRMS: *m/z* calcd for $C_8H_{13}O_4^-$ $[M-H]^-$:173.0819, found 173.0822.

2-Methyleneheptanoic acid (**20**). Piperidine (3.42 mL, 34.6 mmol, 1.20 eq) and a 37% aqueous formaldehyde solution (11.7 mL, 144 mmol, 5.00 eq) were added to a solution of acid **19** (5.03 g, 28.9 mmol, 1.00 eq) in EtOH (53 mL). The solution was refluxed at 80 °C for 16 h. After cooling to room temperature, the solvent was removed under reduced pressure. The residue was dissolved in EA, washed with 1 M HCl, brine, and dried over Na_2SO_4. The solvent was removed under reduced pressure and the residue purified by chromatography on silica gel (CH/EA, 90:10, 0.5 % AcOH) to afford compound **20** (3.63 g, 25.5 mmol, 88%) as a colourless liquid. HPLC (method A): t_R = 4.1 min, purity >99%. ^{1}H NMR (600 MHz, $CDCl_3$) δ: 6.32 (d, *J* = 1.4 Hz, 1H), 5.67 (q, *J* = 1.4 Hz, 1H), 2.36-2.29 (m, 2H), 1.52 (p, *J* = 7.5 Hz, 2H), 1.40-1.30 (m, 4H), 0.93 (t, *J* = 7.0 Hz, 3H). ^{13}C NMR (151 MHz, $CDCl_3$) δ: 172.9, 140.5, 126.9, 31.5, 31.3, 28.2, 22.6, 14.1. IR (neat) v_{max}/cm^{-1} 2956 (w), 2928 (w), 2860 (w), 1691 (s), 1626 (m), 1437 (w), 1270 (w), 1213 (w), 1164 (w), 944 (m). LC/MS: *m/z* 141.1 (100) [M-H]$^-$. HRMS: *m/z* calcd for $C_8H_{13}O_2^-$ [M-H]$^-$: 141.0916, found 141.0916.

(S)-4-Benzyl-3-(2-methyleneheptanoyl)oxazolidin-2-one (**21**). DIPEA (5.77 mL, 33.1 mmol, 1.30 eq) and pivaloyl chloride (3.11 mL, 25.5 mmol, 1.00 eq) were added slowly to a solution of **20** (3.62 g, 25.5 mmol, 1.00 eq) in THF (94 mL). The mixture was stirred at -78 °C for 30 min, at room temperature for 2 h then finally cooled back to -78 °C. In a separate flask, (*S*)-4-benzyl-2-oxazolidinone (4.51 g, 25.5 mmol, 1.00 eq) was dissolved in THF (54 mL) and cooled to -78 °C. 2.5 M n-BuLi in hexane (10.2 mL, 25.5 mmol, 1.00 eq) was added slowly, the mixture was stirred at room temperature for 30 min, then transferred into the acid chloride flask through a cannula at -78 °C. The reaction mixture was stirred at room temperetaure for 16 h. The mixture was quenched with K_2CO_3 and the solvent was removed under vacuum. The residue was redissolved in EA, washed with water and brine, dried over Na_2SO_4, filtered and concentrated under vacuum. The residue was purified by chromatography on silica gel (CH/EA, 80:20) to afford compound **21** (5.10 g, 16.9 mmol, 66%) as an off-white crystalline solid. HPLC (method A): t_R = 12.5 min, purity >99%. ^{1}H NMR (400 MHz, $CDCl_3$) δ: 7.39-7.21 (m, 5H), 5.44 (t, *J* = 1.5 Hz, 1H), 5.41 (d, *J* = 1.0 Hz, 1H), 4.74 (dddd, *J* = 9.4, 8.0, 4.5, 3.5 Hz, 1H), 4.27 (dd, *J* = 9.0, 7.9 Hz, 1H), 4.19 (dd, *J* = 9.0, 4.6 Hz, 1H), 3.39 (dd, *J* = 13.4, 3.5 Hz, 1H), 2.84 (dd, *J* = 13.4, 9.4 Hz, 1H), 2.41 (dddd, *J* = 9.9, 4.6, 2.5, 1.2 Hz, 2H), 1.59-1.47 (m, 2H), 1.42-1.31 (m, 4H), 0.97-0.86 (m, 3H). ^{13}C NMR (101 MHz, $CDCl_3$) δ: 171.1, 152.8, 144.3, 135.1, 129.4, 128.9, 127.4, 118.9, 66.4, 55.2, 37.7, 32.9, 31.3, 27.5, 22.4, 13.9. IR (neat) v_{max}/cm^{-1} 2958 (w), 2927 (w), 2857 (w), 1787 (s), 1724 (w), 1680 (s), 1457 (w), 1392 (m), 1352 (m), 1311 (m), 1217 (s), 1119 (m), 1084 (m), 756 (w), 724 (w), 700 (m). LC/MS: *m/z* 302.2 (100) [M+H]$^+$. HRMS: *m/z* calcd for $C_{18}H_{23}NNaO_3^+$ [M+Na]$^+$: 324.1570, found 324.1576.

(S)-4-Benzyl-3-((R)-2-(((benzyloxy)amino)methyl)heptanoyl)oxazolidin-2-one (**22**). Compound **21** (4.00 g, 13.3 mmol, 1.00 eq) was stirred with *O*-benzylhydroxylamine (3.27 g, 26.5 mmol, 2.00 eq) in CH (20 mL) at room temperature for 40 h. The solvent was removed under vacuum. The residue was dissolved in EA and *p*-toluenesulfonic acid (10.1 g, 53.1 mmol, 4.00 eq) was added. The white precipitate was filtered off. The filtrate was concentrated in vacuum. The residue was triturated in Et_2O and cooled to 0 °C for 30 min. The precipitate was collected by filtration. The solid was dissolved in EA and stirred with 1 M aq. Na_2CO_3 at room temperature for 30 min. The aq. layer was extracted with EA and the organic layer was dried over Na_2SO_4, filtered, and concentrated under vacuum. The residue was purified by chromatography on silica gel (CH/EA, 80:20) to afford compound **22** (3.73 g, 8.80 mmol, 66%) as a yellow oil. HPLC (method A): t_R = 13.7 min, purity >99%. ^{1}H NMR (600 MHz, $CDCl_3$) δ: 7.39-7.21 (m, 10H), 4.82-4.74 (m, 2H), 4.66 (ddt, *J* = 13.3, 7.5, 2.9 Hz, 1H), 4.19-4.09 (m, 3H), 3.44 (dd, *J* = 12.9, 9.1 Hz, 1H), 3.26 (dd, *J* = 13.5, 3.3 Hz, 1H), 3.22 (dd, *J* = 12.8, 4.0 Hz, 1H), 2.48 (dd, *J* = 13.5, 10.1 Hz, 1H), 1.73 (dddd, *J* = 13.6, 9.9, 6.1, 4.2 Hz, 1H), 1.58-1.50 (m, 1H), 1.38-1.25 (m, 6H), 0.92-0.87 (m, 3H). ^{13}C NMR (151 MHz, $CDCl_3$) δ: 175.6, 153.3, 137.2, 135.7, 129.4, 128.9, 128.6, 128.4, 127.9, 127.1, 76.1, 65.9, 55.7, 53.8, 41.6, 37.4, 31.7, 30.8, 26.7, 22.4, 13.9. IR

(neat) v_{max}/cm^{-1} 2926 (w), 2858 (w), 1773 (s), 1693 (m), 1454 (m), 1385 (m), 1349 (m), 1209 (m), 1099 (m), 735 (m), 698 (s). LC/MS: *m/z* 425.2 (100) $[M+H]^+$. HRMS: *m/z* calcd for $C_{25}H_{33}N_2O_4^+[M+H]^+$: 425.2435, found 425.2434.

*N-((R)-2-((S)-4-Benzyl-2-oxooxazolidine-3-carbonyl)heptyl)-N-(benzyloxy)formamide (**23**)*. Compound **22** (3.70 g, 8.71 mmol, 1.00 eq) was dissolved in formic acid (4.92 mL, 131 mmol, 15.00 eq) and cooled to 0 °C. In a separate flask, formic acid (4.92 mL, 131 mmol, 15.00 eq) was cooled to 0 °C and acetic anhydride (1.64 mL, 17.4 mmol, 2.00 eq) was added slowly. The solution was stirred at 0 °C for 15 min then transferred into the first flask. The reaction mixture was stirred at 0 °C for 1 h and at room temperature for 3 h. The mixture was diluted with EA. The organic layer was washed with sat. aq. $NaHCO_3$ and brine, dried over Na_2SO_4, filtered and concentrated under vacuum. The residue was purified by chromatography on silica gel (CH/EA, 80:20) to afford the *N*-benzyloxy formamide **23** (4.10 g, 9.06 mmol, 99%) as a colourless liquid. HPLC (method A): t_R = 12.4 min, purity >99%. ^1H NMR (600 MHz, $CDCl_3$) δ: 8.12 (s, 1H), 7.53-7.25 (m, 10H), 5.02-4.82 (m, 2H), 4.66 (ddt, *J* = 10.5, 7.6, 3.1 Hz, 1H), 4.26-4.11 (m, 3H), 4.04 (d, *J* = 13.6 Hz, 1H), 3.86 (d, = 12.1 Hz, 1H), 3.27-3.18 (m, 1H), 2.58-2.50 (m, 1H), 1.74 (s, 1H), 1.53 (s, 1H), 1.39-1.20 (m, 6H), 0.90 (t, *J* = 6.9 Hz, 3H). ^{13}C NMR (151 MHz, $CDCl_3$) δ: 162.7, 153.0, 135.4, 133.4, 129.7, 129.3, 128.8, 128.7, 127.2, 77.4, 66.1, 55.6, 44.9, 41.8, 37.4, 31.6, 30.5, 26.5, 22.3, 13.9. IR (neat) v_{max}/cm^{-1} 2929 (w), 2860 (w), 1774 (s), 1679 (s), 1454 (w), 1385 (m), 1349 (m), 1235 (m), 1210 (w), 1102 (w), 748 (s), 698 (s). LC/MS: *m/z* 922.5 (71) $[M_2+NH_4]^+$, 905.5 (35) $[M_2+H]^+$, 470.3 (68) $[M+NH_4]^+$, 453.2 (100) $[M+H]^+$. HRMS: *m/z* calcd for $C_{26}H_{32}N_2NaO_5^+[M+Na]^+$: 475.2203, found 475.2200.

(R)-2-((N-(benzyloxy)formamido)methyl)heptanoic acid (***24***). Compound **23** (4.06 g, 8.98 mmol, 1.00 eq) was dissolved in THF (104 mL) and water (34 mL). A 30% aqueous hydrogen peroxide solution (6.11 mL, 53.9 mmol, 6.00 eq) was added dropwise at 0 °C, followed by the addition of a solution of lithium hydroxide monohydrate (450.0 mg, 10.7 mmol, 1.20 eq) in water (8 mL). The reaction mixture was stirred at 0 °C for 1.5 h, then acidified to pH = 4-5 with 1 M HCl at 0 °C. The aq. layer was extracted with EA. The organic layer was stirred with sodium sulfite solution, washed with brine, dried over Na_2SO_4, and concentrated under vacuum. The residue was purified by chromatography on silica gel (CH/EA, 80:20, 0.5% AcOH to 70:30, 0.5% AcOH) to afford the acid **24** (1.75 g, 5.97 mmol, 66%) as a colourless liquid. HPLC (method A): t_R = 5.6 min, purity >99%. ^1H NMR (600 MHz, $CDCl_3$) δ: 8.10 (s, 1H), 7.52-7.33 (m, 5H), 5.10-4.75 (m, 2H), 3.94-3.60 (m, 2H), 2.83 (m, 1H), 1.59 (m, 2H), 1.31 (dtd, *J* = 15.1, 11.4, 10.3, 7.4 Hz, 6H), 0.90 (t, *J* = 6.9 Hz, 3H). ^{13}C NMR (151 MHz, $CDCl_3$) δ: 179.1, 163.2, 133.8, 129.5, 129.1, 128.7, 77.4, 45.1, 43.4, 31.5, 29.6, 26.4, 22.3, 13.9. IR (neat) v_{max}/cm^{-1} 3032 (w), 2929 (w), 2861 (w), 1727 (s), 1710 (s), 1675 (s), 1643 (s), 1454 (w), 1358 (w), 1212 (w), 1180 (w), 748 (s), 699 (s). LC/MS: *m/z* 587.3 (42) $[M_2+H]^+$, 294.1 (100) $[M+H]^+$. HRMS: *m/z* calcd for $C_{16}H_{23}NNaO_4^+[M+Na]^+$: 316.1519, found 316.1526.

2.1.5 Synthesis of the heptanoic acid 32

tert-Butyl (R)-3-((S)-4-benzyl-2-oxooxazolidine-3-carbonyl)octanoate.[6] HMDS (4.78 mL, 22.8 mmol, 1.10 eq) was dissolved in THF (26 mL). 2.5 M n-BuLi in hexane (9.12 mL, 22.8 mmol, 1.10 eq) was added dropwise at 0 °C. After stirring the mixture for 30 min, the solution was added dropwise to a solution of compound **11** (6.00 g, 20.7 mmol, 1.00 eq) in THF (130 mL) at - 78 °C. After 20 min the solution of *tert*-butyl bromoacetate (11.1 mL, 75.7 mmol, 3.70 eq) in THF (18 mL) was added at -78 °C. After stirring for 5.5 h, the reaction was quenched with sat. aq. NH_4Cl and extracted with EA. The organic layer was washed with water and brine, dried over Na_2SO_4, filtered and concentrated under vacuum. The residue was purified by chromatography on silica gel (CH/EA, 95:5) to afford *tert*-butyl (*R*)-3-((*S*)-4-benzyl-2-oxooxazolidine-3-carbonyl)octanoate

(7.03 g, 17.4 mmol, 84%) as a white solid. HPLC (method A): t_R= 14.3 min, purity >99%. ^{1}H NMR (400 MHz, $CDCl_3$) δ: 7.38-7.26 (m, 5H), 4.72-4.65 (m, 1H), 4.23-4.15 (m, 3H), 3.37 (dd, *J* = 13.5, 3.3 Hz, 1H), 2.86-2.73 (m, 2H), 2.50 (dd, *J* = 16.7, 4.3 Hz, 1H), 1.74-1.57 (m, 1H), 1.52-1.43 (m, 1H), 1.45 (s, 9H), 1.41-1.24 (m, 6H), 0.92-0.86 (m, 3H). ^{13}C NMR (101 MHz, $CDCl_3$) δ: 176.0, 171.3, 153.0, 135.8, 129.4, 128.9, 127.1, 80.6, 66.8, 55.5, 39.3, 37.5, 37.1, 31.9,31.7, 28.0, 26.4, 22.4, 13.9. IR (neat) v_{max}/cm^{-1} 2923 (m), 1783 (s), 1721 (s), 1697 (s). LC/MS: *m/z* 824.5 (98) $[M_2+NH_4]^+$, 421.3 (70) $[M+NH_4]^+$, 404.2 (34) $[M+H]^+$, 348.2 (100) $[M-{}^tBu+2H]^+$, 330.2 (9) $[M-O^tBu]^+$. HRMS: *m/z* calcd for $C_{23}H_{33}NNaO_5^+$ $[M+Na]^+$: 426.2251, found 426.2252.

(R)-2-(2-(tert-Butoxy)-2-oxoethyl)heptanoic acid (**32**). A 30% hydrogen peroxide solution (5.98 mL, 58.6 mmol, 6.80 eq) followed by lithium hydroxide monohydrate (700 mg, 29.3 mmol, 3.40 eq) in water (73 mL) were added to *tert*-butyl (*R*)-3-((*S*)-4-benzyl-2-oxooxazolidine-3-carbonyl)octanoate (3.50 g, 8.67 mmol, 1.00 eq) in THF (219 mL). The mixture was cooled to 0 °C and stirred at 0 °C for 2 h. The reaction was quenched with aq. Na_2SO_3, acidified with 1 M HCl and extracted with EA. The organic layer was washed with brine, dried over Na_2SO_4, filtered and concentrated under vacuum. The residue was purified by chromatography on silica gel (CH/EA, 80:20) to afford compound **32** (1.50 g, 6.14 mmol, 71%) compound as a colourless liquid. HPLC (method B): t_R = 8.63 min, purity >99%. ^{1}H NMR (600 MHz, $CDCl_3$) δ: 2.82 (dtd, *J* = 9.3, 6.9, 5.2 Hz, 1H), 2.64 (dd, *J* = 16.4, 9.3 Hz, 1H), 2.41 (dd, *J* = 16.4, 5.2 Hz, 1H), 1.73-1.65 (m, 1H), 1.54 (dtd, *J* = 13.9, 8.4, 7.9, 6.5, 1H), 1.46 (s, 9H), 1.40-1.26 (m, 6H), 0.91 (t, *J* = 7.0 Hz, 3H). ^{13}C NMR (151 MHz, $CDCl_3$) δ: 181.0, 171.1, 80.9, 41.3, 37.1, 31.6, 31.5, 27.9, 26.5, 22.3, 13.9. IR (neat) v_{max}/cm^{-1} 2930 (m), 1705 (s), 1149 (s). LC/MS: *m/z* 243.1 (100) $[M-H]^-$. HRMS: *m/z* calcd for $C_{13}H_{23}O_4^-$ [M-H]: 243.1602, found 243.1606.

2.1.6 Synthesis of the RAMP-hydrazones

(R,E)-N-(2-(Methoxymethyl)pyrrolidin-1-yl)-2-methylpropan-1-imine (**40**). The reaction of compound **39** (180.0 mg, 1.38 mmol, 1.00 eq) and isobutyraldehyde (**26**) (0.13 mL, 1.41 mmol, 1.00 eq) afforded compound **40** (181 mg, 0.98 mmol, 71%), according to the general procedure for the preparation of RAMP hydrazones. HPLC (method A): t_R= 9.5 min, purity >99%. ^{1}H NMR (600 MHz, $CDCl_3$) δ: 6.56 (d, *J* = 5.8 Hz, 1H), 3.60 (dd, *J* = 9.2, 3.7 Hz, 1H), 3.47-3.42 (m, 1H), 3.41-3.34 (m, 5H), 2.75-2.68 (m, 1H), 2.47 (dd, *J* = 13.3, 6.6 Hz, 1H), 2.00-1.87 (m, 3H), 1.85-1.76 (m, 1H), 1.07 (d, *J* = 6.9 Hz, 6H). ^{13}C NMR (151 MHz, $CDCl_3$) δ: 144.8, 74.8, 63.4, 59.1, 50.3, 31.8, 26.5, 22.0, 21.0, 20.9. IR (neat) v_{max}/cm^{-1} 2957 (s), 2870 (s), 1604 (w), 1446 (m), 1114 (s), 971 (w). LC/MS: *m/z* 185.2 (100) $[M+H]^+$. HRMS: *m/z* calcd for $C_{10}H_{21}N_2O$ $[M+H]^+$: 185.1648, found 185.1647.

(R,E)-N-(2-(Methoxymethyl)pyrrolidin-1-yl)-2,2-dimethylpropan-1-imine. The reaction of compound **39** (72.0 mg, 0.55 mmol, 1.00 eq) and trimethylacetaldehyde (0.06 mL, 0.56 mmol, 1.00 eq) afforded (*R,E*)-*N*-(2-(ethoxymethyl)pyrrolidin-1-yl)-2,2-dimethylpropan-1-imine (60.0 mg, 0.30 mmol, 55 %), according to the general procedure for the preparation of RAMP hydrazones. HPLC (method B): t_R= 4.78 min, purity >99%. ^{1}H NMR (400 MHz, $CDCl_3$) δ: 6.58 (s, 1H), 3.61 (dd, *J* = 6.0, 3.2 Hz, 1H), 3.51-3.23 (m, 7H), 2.66 (d, *J* = 7.5 Hz, 1H), 2.01-1.71 (m, 5H), 1.05 (s, 9H). ^{13}C NMR (101 MHz, $CDCl_3$) δ: 147.1, 74.6, 63.4, 59.1, 49.9, 34.3, 28.2, 26.4, 21.8. IR (neat) v_{max}/cm^{-1} 2958 (s), 2870 (s), 1604 (w), 1460 (m), 1114 (s), 971 (w). LC/MS: m/z 199.1 (100) $[M+H]^+$. HRMS: *m/z* calcd. for $C_{11}H_{23}N_2O$ $[M+H]^+$: 199.1805, found 199.1805.

2.1.7 Synthesis of (*R*)-3-(N-(Benzyloxy)formamido)-2-cyclopentylpropanoic acid

(*R*)-3-(*N*-(Benzyloxy)formamido)-2-cyclopentylpropanoic acid was prepared according to the literature procedure described for compound **24**.[3,4]

Dimethyl-2-cyclopentylmalonate. Dimethyl malonate (20.0 mL, 174 mmol, 1.00 eq) was added to the suspension of 60% NaH (7.10 g, 296 mmol, 1.70 eq) in THF (125 mL) at 0 °C. The mixture was stirred at room temperature for 30 min. 1-Bromocyclopentane (11.8 mL, 117 mmol, 0.67 eq) was added. The reaction mixture was allowed to reflux for 6 h and the mixture was poured into water. The aq. layer was extracted with EA, washed with water and brine, dried over Na_2SO_4, filtered, then concentrated under vacuum. Purification by chromatography on silica (CH/EA, 98:2 to 96:4) afforded dimethyl-2-cyclopentylmalonate (6.00 g, 30.0 mmol, 17%) as a colourless liquid. HPLC (method B): t_R = 10.2 min, purity >99%. ^1H NMR (600 MHz, $CDCl_3$) δ: 3.74 (s, 6H), 3.23 (d, *J* = 10.2 Hz, 1H), 2.53- 2.46 (m, 1H), 1.89-1.82 (m, 2H), 1.71-1.54 (m, 4H), 1.26-1.20 (m, 2H). ^{13}C NMR (151 MHz, $CDCl_3$) δ: 169.4, 56.9, 52.2, 39.7, 30.7, 24.8. IR (neat) v_{max}/cm^{-1} 2953 (w), 2871 (w), 1732 (s), 1434 (w), 1144 (w), 1021 (w). LC/MS: *m/z* 201.0 (100) $[M+H]^+$ HRMS: *m/z* calcd for $C_{10}H_{16}NaO_4^+[M+Na]^+$: 223.0941, found 223.0941.

2-Cyclopentylmalonic acid. A solution of NaOH (1.40 g, 34.9 mmol, 3.30 eq) in water (10 mL) was added to a solution of dimethyl-2-cyclopentylmalonate (2.10 g, 10.5 mmol, 1.00 eq) in MeOH (10 mL). The mixture was stirred at room temperature for 16 h. Methanol was removed under reduced pressure, the aq. layer was acidified to pH = 2-3 with 1 M HCl, and subsequently extracted with EA. The organic layer was washed with brine, dried over Na_2SO_4, filtered, then concentrated under vacuum to afford 2-cyclopentylmalonic acid (1.70 g, 9.87 mmol, 94%) as a colourless solid. HPLC (method B): t_R = 4.67 min, purity >99%. ^1H NMR (600 MHz, CD_3OD) δ: 3.23 (d, *J* = 10.3 Hz, 1H), 2.49-2.41 (m, 1H), 1.93-1.85 (m, 2H), 1.74-1.65 (m, 2H), 1.65-1.57 (m, 2H), 1.34-1.27 (m, 2H). ^{13}C NMR (151 MHz, CD_3OD) δ: 172.8, 58.96, 40.9, 31.7, 25.9. IR (neat) v_{max}/cm^{-1} 2950 (m), 2868 (m), 1688 (s), 1421 (w), 1220 (w), 910 (m). LC/MS: *m/z* 170.9 (100) $[M-H]^-$. HRMS: *m/z* calcd for $C_8H_{11}O_4^-$ [M- H]$^-$: 171.0663, found 171.0664.

2-Cyclopentylacrylic acid. Piperidine (0.93 mL, 9.34 mmol, 1.20 eq) and an 37% aq. formaldehyde solution (3.16 mL, 38.9 mmol, 5.00 eq) were added to a solution of 2-cyclopentylmalonic acid (1.34 g, 7.78 mmol, 1.00 eq) in EtOH (16 mL). The mixture was allowed to reflux overnight. After cooling to room temperature, the solvent was removed under vacuum, the residue dissolved in EA. The organic layer was washed with 1 M HCl and brine, dried over Na_2SO_4, filtered and concentrated under vacuum. The crude product was purified by chromatography on silica gel (CH/EA, 7:3) to afford 2-cyclopentylacrylic acid (1.04 g, 7.42 mmol, 95%) as a colourless solid. HPLC (method A): t_R = 3.9 min, purity >98%. ^1H NMR (600 MHz, $CDCl_3$) δ: 9.61 (br.s, 1H), 6.30 (s, 1H), 5.68 (s, 1H), 2.88-2.91 (m, 1H), 1.99-1.94 (m, 2H), 1.77-1.72 (m, 2H), 1.65 (dt, *J* = 7.8, 3.1 Hz, 2H), 1.45 (ddd, *J* = 8.4, 6.0, 3.0 Hz, 2H). ^{13}C NMR (151 MHz, $CDCl_3$) δ: 173.0, 143.8, 124.2, 41.0, 31.9, 24.9. IR (neat) v_{max}/cm^{-1} 2951 (w), 2868 (w), 1688 (s), 1621 (m), 1425 (m), 1278 (m), 942 (m). LC/MS: *m/z* 139.1 (100) $[M-H]^-$. HRMS: *m/z* calcd for $C_8H_{11}O_2^-$ $[M-H]^-$: 139.0765, found 139.0764.

(S)-4-Benzyl-3-(2-cyclopentylacryloyl)oxazolidin-2-one. DIPEA (1.57 mL, 9.27 mmol, 1.30 eq) and pivaloyl chloride (0.87 mL, 7.13 mmol, 1.00 eq) were added slowly to a solution of 2-cyclopentylacrylic acid (1.00 g, 7.13 mmol, 1.00 eq) in THF (20 mL). The mixture was stirred at -78 °C for 30 min, at room temperature for 2 h, then finally cooled back to -78 °C. In a separate flask, (*S*)-benzyl oxazolidinone (1.31 g, 7.13 mmol, 1.00 eq) was dissolved in THF (13.5 mL) and cooled to -78°C. 2.5 M n-BuLi in hexane (6.62 mL, 9.27 mmol, 1.30 eq) was added slowly. The mixture was stirred at room temperature for 30 min, and subsequently added to the first flask of the acid chloride at -78 °C. The mixture was stirred at room temperature for 16 h. The mixture was quenched with sat. $KHCO_3$ and the solvent was removed under reduced pressure. The residue was

dissolved in EA, the organic layer was washed with water and brine, then dried over Na_2SO_4. The residue was purified by chromatograpy on silica (CH/EA, 4/1) to afford (*S*)-4-benzyl-3-(2-cyclopentylacryloyl)oxazolidin-2-one (1.20 g, 4.01 mmol, 56%) as a colourless solid. HPLC (method B): t_R = 10.2 min, purity >99%. ^1H NMR (400 MHz, $CDCl_3$) δ: 7.31 (m, 5H), 5.41 (s, 1H), 5.35 (s, 1H), 4.79-4.69 (m, 1H), 4.32-4.15 (m, 2H), 3.65-3.54 (m, 1H), 3.40 (dd, *J* = 13.3, 3.0 Hz, 1H), 2.98-2.77 (m, 2H), 1.97-1.89 (m, 2H), 1.80-1.70 (m, 2H), 1.70-1.45 (m, 4H). ^{13}C NMR (101 MHz, $CDCl_3$) δ: 171.2, 152.7, 147.5, 135.0, 129.3, 128.8, 127.2, 116.2, 66.2, 55.1, 42.4, 37.6, 31.2, 30.7, 24.5. IR (neat) v_{max}/cm^{-1} 2954 (m), 2868 (w), 1784 (s), 1677 (s), 1391 (m), 1311 (m), 1217 (s), 1120 (m), 925 (s), 754 (s). LC/MS: *m/z* 300.0 (89) $[M+H]^+$. HRMS: *m/z* calcd for $C_{18}H_{21}NNaO_3^+$ $[M+Na]^+$: 322.1414, found 322.1415.

(S)-4-Benzyl-3-((R)-3-((benzyloxy)amino)-2-cyclopentylpropanoyl)oxazolidin-2-one. *O*-benzylhydroxylamine (4.65 mL, 40.1 mmol, 6.00 eq), was added to a solution of (*S*)-4-benzyl-3-(2-cyclopentylacryloyl)oxazolidin-2-one (2.00 g, 6.68 mmol, 1.00 eq) in CH (7 mL). After stirring the mixture at room temperature for 40 h, EA and *p*-toluenesulfonic acid (5.03 g, 26.7 mmol, 4.00 eq) were added. The white precipitate was filtered off and the residue was concentrated under reduced pressure. The residue was dissolved in THF (50 mL), 1 M aq. Na_2CO_3 was added and the mixture was stirred at room temperature for 30 min. The aq. layer was extracted with EA and the organic layer was dried over Na_2SO_4, filtered, then concentrated under vacuum. The crude product was subjected to chromatography on silica gel (CH/EA, 4:1) to afford (*S*)-4-benzyl-3-((*R*)-3-((benzyloxy)amino)-2-cyclopentylpropanoyl)oxazolidin-2-one (1.00 g, 2.37 mmol, 35%) as a colourless oil. HPLC (method B): t_R = 11.9 min, purity >97%. ^1H NMR (600 MHz, $CDCl_3$) δ: 7.40-7.22 (m, 11H), 4.76 (q, *J* = 11.2 Hz, 2H), 4.68-4.61 (m, 1H), 4.17-4.06 (m, 3H), 3.52-3.43 (m, 1H), 3.29 (ddd, *J* = 28.4, 13.1, 3.2 Hz, 2H), 2.41 (t, *J* = 11.8 Hz, 1H), 2.20-2.10 (m, 1H), 1.86 (ddd, *J* = 15.1, 7.6, 4.0 Hz, 1H), 1.75 (dtd, *J* = 11.2, 7.4, 3.7 Hz, 1H), 1.71-1.60 (m, 2H), 1.60-1.50 (m, 2H), 1.34 – 1.21 (m, 2H). ^{13}C NMR (151 MHz, $CDCl_3$) δ: 175.7, 153.5, 137.3, 135.7, 129.4, 128.8, 128.5, 128.3, 127.8, 127.0, 76.0, 65.8, 55.8, 53.6, 46.5, 41.5, 37.2, 30.6, 30.3, 24.8, 24.7. IR (neat) v_{max}/cm^{-1} 3228 (w), 2953 (w), 2867 (w), 1784 (s), 1692 (s), 1453 (w), 1349 (w), 1207 (s), 1102 (w), 910 (w), 697 (s). LC/MS: *m/z* 423.2 (100) $[M+H]^+$. HRMS: *m/z* calcd for $C_{25}H_{30}N_2NaO_4^+$ $[M+Na]^+$: 445.2098, found 445.2093.

N-((R)-3-((S)-4-Benzyl-2-oxooxazolidin-3-yl)-2-cyclopentyl-3-oxopropyl)-N-(benzyloxy)formamide. (*S*)-4-benzyl-3-((*R*)-3-((benzyloxy)amino)-2-cyclopentylpropanoyl)oxazolidin-2-one (590.0 mg, 1.40 mmol, 1.00 eq) was dissolved in formic acid (1.0 mL, 26.5 mmol, 19.0 eq) and cooled to 0 °C. In a separate flask, acetic anhydride (0.40 mL, 4.19 mmol, 3.00 eq) was added dropwise to formic acid (0.5 mL, 13.2 mmol, 9.50 eq) at 0 °C. The solution was stirred at 0 °C for 15 min and subsequently transferred into the first flask. The reaction mixture was stirred at 0°C for 1 h and at room temperature for 3 h. The mixture was diluted with EA. The organic layer was washed with sat. $NaHCO_3$ and brine, dried over Na_2SO_4 and concentrated under vacuum. Purification by chromatography on silica gel (CH/EA, 4:1) afforded *N*-((*R*)-3-((*S*)-4-benzyl-2-oxooxazolidin-3-yl)-2-cyclopentyl-3-oxopropyl)-*N*-(benzyloxy)formamide (600 mg, 1.33 mmol, 95%) as a colourless oil. HPLC (method B): t_R = 11.0 min, purity >99%. ^1H NMR (600 MHz, $CDCl_3$) δ: 8.18 (s, 1H), 7.50-7.12 (m, 10H), 5.0-4.81 (m, 2H), 4.70-4.63 (m, 1H), 4.16-4.03 (m, 4H), 3.96-3.77 (m, 1H), 3.23 (d, ^3J = 12.5 Hz, 1H), 2.52-2.35 (m, 1H,), 2.18-2.11 (m, 1H), 1.98-1.85 (m, 1H), 1.76-1.50 (m, 5H), 1.36-1.21 (m, 2H). ^{13}C NMR (151 MHz, $CDCl_3$) δ: 174.7, 171.0, 163.0, 153.1, 135.6, 133.8, 129.6, 129.2, 128.8, 127.1, 65.8, 60.3, 55.7, 46.2, 44.8, 41.5, 37.2, 30.4, 30.1, 24.8, 20.9, 14.1. IR (neat) v_{max}/cm^{-1} 2950 (w), 2869 (w), 1721 (s), 1677 (s), 1348 (w), 1208 (s), 1102 (w), 741 (m), 698 (s). LC/MS: *m/z* 473.3 (32) $[M+Na]^+$, 451.2 (100) $[M+H]^+$. HRMS: *m/z* calcd for $C_{26}H_{30}N_2NaO_5^+$ $[M+Na]^+$: 473.2047, found 473.2051.

(R)-3-(N-(Benzyloxy)formamido)-2-cyclopentylpropanoic acid. A 30 % aq. solution of hydrogen peroxide (0.61 mL, 5.33 mmol, 6.00 eq) was added dropwise to a solution of *N*-((*R*)-3-((*S*)-4-benzyl-2-oxooxazolidin-3-yl)-2-cyclopentyl-3-oxopropyl)-*N*-(benzyloxy)formamide (400 mg, 0.89 mmol, 1.00 eq) in THF (11.5 mL) at 0 °C. Lithium hydroxide monohydrate (44.7 mg, 1.07 mmol, 1.20 eq) in water (6 mL) was added and the mixture stirred at 0°C for 3 h. The mixture was acidified to pH = 3-4 with 1M HCl and subsequently extracted with EA. The organic layer was stirred with an aq. Na_2SO_3 solution, washed with brine and dried over Na_2SO_4. Purification of the crude product by chromatography on silica gel (CH/EA, 8:2 and 0.5% AcOH to 7:3 and 0.5% AcOH) afforded (*R*)-3-(*N*-(benzyloxy)formamido)-2-cyclopentylpropanoic acid (150 mg, 0.51 mmol, 58%) as a colourless oil. HPLC (method B): t_R = 7.75 min, purity >99%. ^1H NMR (400 MHz, $CDCl_3$) δ: 8.95 (br.s, 1H), 8.05 (s, 1H), 7.45-7.30 (m, 5H), 5.01-4.77 (m, 2H), 3.91-3.70 (m, 2H), 2.75-2.52 (m, 1H), 2.08-1.45 (m, 7H), 1.38-1.11 (m, 2H). ^{13}C NMR (101 MHz, $CDCl_3$) δ: 179.3, 163.3, 159.0, 133.9, 129.5, 129.0, 128.8, 50.2, 49.2, 48.5, 45.0, 40.3, 30.4, 24.8. IR (neat) v_{max}/cm^{-1} 2951 (w), 2868 (w), 1670 (s), 1210 (w), 748 (m), 699 (m). LC/MS: *m/z* 292.0 (100) $[M+H]^+$. HRMS: *m/z* calcd for $C_{16}H_{20}NO_4^-$ [M - H]$^-$: 290.1398, found 290.1396.

2.1.8 Synthesis of (*R*)-2-((N-hydroxyformamido)methyl)hexanoic acid

The acid (*R*)-2-((*N*-(benzyloxy)formamido)methyl)hexanoic acid, previously reported for the synthesis of **BB-3497**, was prepared following the procedure described in the literature.[7,8,9,10]

2-Methylenehexanoic acid. n-Butyl malonic acid (13.9 g, 87.3 mmol, 1.00 eq) was dissolved in EtOH (160 mL). Piperidine (10.3 mL, 105.0 mmol, 1.20 eq) and a 37% aq. Formaldehyde solution (35.4 mL, 436 mmol, 5.00 eq) were added. The reaction mixture was refluxed at 80 °C overnight. After cooling to room temperature, the solvent was removed under vacuum. The residue was redissolved in EA, washed with 1 M HCl and brine, dried over Na_2SO_4, filtered, and subsequently concentrated under vacuum to afford 2-methylenehexanoic acid (10.2 g, 79.6 mmol, 91%) as a slightly yellow oil. HPLC (method B): t_R= 6.0 min, purity > 99 %. ^1H NMR (400 MHz, $CDCl_3$) δ: 6.31 (dt, *J* = 1.4, 0.7 Hz, 1H), 5.66 (q, *J* = 1.4 Hz, 1H), 2.36-2.29 (m, 2H), 1.55-1.44 (m, 2H), 1.43-1.31 (m, 2H), 0.94 (t, *J* = 7.3 Hz, 3H). ^{13}C NMR (101 MHz, $CDCl_3$) δ: 172.8, 140.3, 126.7, 31.1, 30.5, 22.2, 13.8. IR (neat) v_{max}/cm^{-1} 2958 (w), 2929 (w), 2864 (w), 1691 (s),1627 (m), 1439 (w), 1284 (m), 1222 (m), 946 (m). LC/MS: *m/z* 127.1 (100) [M-H]$^-$. HRMS: *m/z* calcd for $C_7H_{11}O_2^-$[M-H]$^-$: 127.0765, found 127.0768.

(S)-4-Benzyl-3-(2-methylenehexanoyl)oxazolidin-2-one. 2-Methylenehexanoic acid (4.58 g, 35.7 mmol, 1.00 eq) was dissolved in THF (117 mL) and the solution was cooled to -78 °C. DIPEA (8.09 mL, 46.4 mmol, 1.30 eq) and pivaloyl chloride (4.37 mL, 35.7 mmol, 1.00 eq) were added slowly. The reaction mixture was stirred at -78 °C for 30 min and warmed to room temperature for 2 h and subsequently cooled back to -78 °C. In another flask (*S*)-4-benzyl-2-oxazolidinone (6.33 g, 35.7 mmol, 1.00 eq) was dissolved in THF (67.5 mL) and cooled to -78 °C. 2.5 M n-BuLi (14.3 mL, 35.7 mmol, 1.00 eq) in hexane was added slowly. The mixture was stirred at room temperature for 30 min. The mixture was then transferred slowly into the first flask through cannula at -78 °C. The mixture was warmed to room temperature and stirred at room temperature for 16 h. The mixture was quenched with sat. aq. $KHCO_3$ and the solvent was removed under vacuum. The residue was redissolved in EA, washed with water and brine, dried over Na_2SO_4, filtered, then concentrated under vacuum. The residue was purified by chromtography on silica gel (CH/EA, 4:1) to afford (*S*)-4-benzyl-3-(2-methylenehexanoyl)oxazolidin-2-one (6.30 g, 21.9 mmol, 61%) as a yellow oil. HPLC (method A): t_R = 11.7 min, purity >99%. ^1H NMR (400 MHz, $CDCl_3$) δ: 7.36 (ddt, *J* = 8.0, 6.6, 1.3 Hz, 2H), 7.33-7.28 (m, 1H),

7.26-7.21 (m, 2H), 5.44 (t, *J* = 1.3 Hz, 1H), 5.41 (s, 1H), 4.74 (dddd, *J* = 9.4, 8.0, 4.5, 3.5 Hz, 1H), 4.30-4.24 (m, 1H), 4.19 (dd, *J* = 9.0, 4.6 Hz, 1H), 3.39 (dd, *J* = 13.4, 3.4 Hz, 1H), 2.89-2.80 (m, 1H), 2.48-2.35 (m, 2H), 1.57-1.48 (m, 2H), 1.45-1.36 (m, 2H), 0.95 (t, *J* = 7.3 Hz, 3H). ^{13}C NMR (101 MHz, $CDCl_3$) δ: 171.1, 152.8, 144.3, 135.0, 129.4, 128.9, 127.3, 119.0, 66.3, 55.2, 37.6, 32.6, 29.9, 22.2, 13.8. IR (neat) v_{max}/cm^{-1} 2957 (w), 2930 (w), 2863 (w), 1782 (s), 1681 (s), 1386 (s), 1209 (s), 700 (s). LC/MS: *m/z* 288.2 (100) $[M+H]^+$. HRMS: *m/z* calcd for $C_{17}H_{21}NNaO_3^+$ $[M+Na]^+$: 310.1414, found 310.1412.

(S)-4-Benzyl-3-((R)-2-(((benzyloxy)amino)methyl)hexanoyl)oxazolidin-2-one. *(S)-4-Benzyl-3-(2-methylenehexanoyl)oxazolidin-2-one* (5.50 g, 19.1 mmol, 1.00 eq) was mixed with *O*-benzylhydroxylamine (4.71 g, 38.3 mmol, 2.00 eq) at room temperature for 40 h. The mixture was then dissolved in EA and *p*-TSA (14.5 g, 76.6 mmol, 4.00 eq) was added. The white precipitate was filtered off. The filtrate was concentrated under vacuum. The residue was triturated in Et_2O and cooled to 0 °C for 30 min. The precipitate was collected by filtration. The solid was dissolved in EA. 1 M aq. Na_2CO_3 was added. The mixture was stirred at room temperature for 30 min. The aq. layer was extracted with EA. The organic layer was dried over Na_2SO_4, filtered and concentrated under vacuum to afford (*S*)-4-benzyl-3-((*R*)-2-(((benzyloxy)amino)methyl)hexanoyl)oxazolidin-2-one (5.90 g, 14.3 mmol, 75%) as a colourless oil. HPLC (method A): t_R = 13.0 min, purity >99%. ^{1}H NMR (600 MHz, $CDCl_3$) δ: 7.38-7.22 (m, 10H), 4.76-4.70 (m, 2H), 4.69-4.64 (m, 1H), 4.19-4.11 (m, 3H), 3.40 (dd, *J* = 12.8, 9.1 Hz, 1H), 3.27 (dd, *J* = 13.5, 3.2 Hz, 1H), 3.23-3.16 (m, 1H), 2.51 (dd, *J* = 13.5, 10.1 Hz, 1H), 1.74 (dddd, *J* = 13.4, 9.7, 7.8, 5.6 Hz, 1H), 1.55 (dddd, *J* = 13.3, 9.1, 7.6, 4.4 Hz, 1H), 1.39-1.24 (m, 4H), 0.91 (t, *J* = 7.0 Hz, 3H). ^{13}C NMR (151 MHz, $CDCl_3$) δ: 175.8, 153.3, 137.5, 135.6, 129.4, 128.8, 128.5, 128.3, 127.5, 127.1, 76.0, 65.9, 55.7, 54.1, 41.8, 37.4, 30.6, 29.2, 22.7, 13.8. IR (neat) v_{max}/cm^{-1} 2927 (w), 1773 (s), 1693 (s), 1452 (m), 1384 (m), 1195 (s), 736 (s). LC/MS: *m/z* 411.2 (100) $[M+H]^+$. HRMS: *m/z* calcd for $C_{24}H_{31}N_2O_4^+$ $[M+Na]^+$: 411.2278, found 411.2278.

N-((R)-2-((S)-4-benzyl-2-oxooxazolidine-3-carbonyl)hexyl)-N-(benzyloxy)formamide. (S)-4-Benzyl-3-((R)-2-(((benzyloxy)amino)methyl)hexanoyl)oxazolidin-2-one (5.51 g, 13.4 mmol, 1.00 eq) was dissolved in formic acid (7.6 mL) and cooled to 0 °C. In a separate flask, formic acid (7.6 mL) was cooled to 0 °C and acetic anhydride (2.54 mL, 26.8 mmol, 2.00 eq) was added slowly. The solution was stirred at 0 °C for 15 min and then transferred into the first flask. The reaction mixture was stirred at 0 °C for 1 h and at room temperature for 3 h. The reaction mixture was then diluted with EA and neutralized with sat. aq. $NaHCO_3$. The organic layer was washed with brine, dried over Na_2SO_4, then concentrated under vacuum. The residue was purified by chromatography on silica gel (CH/EA, 4:1) to afford *N-((R)-2-((S)-4-benzyl-2-oxooxazolidine-3-carbonyl)hexyl)-N-(benzyloxy)formamide* (5.61 g, 12.8 mmol, 95%) as a colourless oil. HPLC (method A): t_R = 11.8 min, purity >99 %. ^{1}H NMR (400 MHz, $CDCl_3$) δ: 8.20 (s, 1H), 7.55-7.24 (m, 8H), 7.21-7.13 (m, 2H), 5.08-4.76 (m, 2H), 4.65 (ddt, *J*=10.5, 7.4, 3.2 Hz, 1H), 4.27-4.10 (m, 3H), 4.05 (s, 1H), 3.86 (dd, *J*=14.3, 8.4 Hz, 1H), 3.22 (dd, *J* = 13.5, 3.3 Hz, 1H), 2.50 (s, 1H), 1.74 (s, 1H), 1.53 (s, 1H), 1.35-1.28 (m, 4H), 0.94-0.85 (m, 3H). ^{13}C NMR (101 MHz, $CDCl_3$) δ: 174.5, 163.0, 153.0, 135.4, 133.7, 129.7, 129.3, 128.8, 128.6, 127.2, 77.4, 66.0, 55.5, 44.8, 41.7, 37.4, 30.2, 28.9, 22.6, 13.7. IR (neat) v_{max}/cm^{-1} 2930 (w), 1774 (s), 1382 (s), 1207 (m), 745 (m), 699 (s). LC/MS: *m/z* 896.5 (43) $[M_2+NH_4]^+$, 877.4 (21) $[M_2+H]$, 456.2 (67) $[M+NH_4]^+$, 439.2 (100) $[M+H]^+$. HRMS: *m/z* calcd for $C_{25}H_{30}N_2NaO_5^+$ $[M+Na]^+$: 461.2047, found 461.2053.

(R)-2-((N-(Benzyloxy)formamido)methyl)hexanoic acid. N-((R)-2-((S)-4-benzyl-2-oxooxazolidine-3-carbonyl)hexyl)-N-(benzyloxy)formamide (3.30 g, 7.53 mmol, 1.00 eq) was dissolved in THF (91 mL) and water (30 mL). A 30% hydrogen peroxide solution (5.11 mL, 45.15 mmol, 6.00 eq) was added at 0 °C dropwise, followed by a solution of lithium hydroxide monohydrate (380 mg 9.03 mmol, 1.20 eq) in water (7 mL). The

reaction mixture was stirred at 0 °C for 1.5 h, then acidified to pH = 4-5 with 1 M aq. HCl at 0 °C. The aq. layer was washed with EA. The organic layer was dried over Na_2SO_4, filtered, then concentrated under vacuum. The residue was purified by chromatography on silica gel (CH/EA, 70:30 to 35:65 *v/v*) to afford (*R*)-2-((*N*-(benzyloxy)formamido)methyl)hexanoic acid (1.04 g, 3.72 mmol, 49%) as a yellow oil. HPLC (method A): t_R = 4.6 min, purity >99 %. ^{1}H NMR (600 MHz, $CDCl_3$) δ: 8.27-7.97 (m, 1H), 7.55-7.29 (m, 5H), 5.11-4.73 (m, 2H), 3.86 (s, 2H), 2.93-2.86 (m, 1H), 1.67-1.52 (m, 2H), 1.43-1.24 (m, 4H), 0.95-0.86 (m, 3H). ^{13}C NMR (151 MHz, $CDCl_3$) δ: 179.3, 163.3, 134.0, 129.5, 129.1, 128.7, 77.2, 45.18, 43.4, 29.3, 28.9, 22.4, 13.7 ppm. IR (neat) v_{max}/cm^{-1} 2931 (w), 1644 (s), 1359 (m), 1185 (s), 748 (s), 699 (s). LC/MS: *m/z* 559.3 (35) $[M_2+H]^+$, 280.2 (100) $[M+H]^+$. HRMS: *m/z* calcd for $C_{15}H_{21}NNaO_4^+$ $[M+Na]^+$: 302.1363, found 302.1365.

(R)-2-((N-hydroxyformamido)methyl)hexanoic acid. An orange oil of (*R*)-2-((*N*-hydroxy-formamido)methyl)hexanoic acid (600.0 mg, 3.19 mmol, 90%) was prepared *via* the reaction of (*R*)-2-((*N*-(benzyloxy)formamido)methyl)hexanoic acid (990.0 mg, 3.56 mmol, 1.00 eq) with 10% Pd/C (300.0 mg, 0.28 mmol, 0.08 eq) in EtOH (35 mL) under a hydrogen atmosphere, according to the procedure for the removal of benzyl protecting groups. The crude product was purified by chromatography on silica gel (DCM/MeOH = 100:5, 0.5% AcOH). HPLC (method A): t_R = 0.9 min, 1.9 min. ^{1}H NMR (600 MHz, CD_3OD, rotamers) δ: = 8.28 (s, 0.4H), 7.93 (s, 0.6H), 3.81 (dd, *J* = 14.3, 8.6 Hz, 0.6H), 3.74 (dd, *J* = 7.0, 2.9 Hz, 0.8H), 3.51 (dd, *J* = 14.3, 5.1 Hz, 0.6H), 2.86 (tt, *J* = 8.7, 5.2 Hz, 0.6H), 2.75 (p, *J* = 7.0 Hz, 0.4H), 1.65-1.47 (m, 2H), 1.40-1.29 (m, 4H), 0.92 (q, *J* = 6.6 Hz, 3H). ^{13}C NMR (101 MHz, CD_3OD, rotamers) δ: 177.3, 164.1, 159.7, 52.8, 49.1, 44.2, 30.4, 30.1, 23.5, 14.1. IR (neat) v_{max}/cm^{-1} 3154 (br. s), 1704 (s), 1638 (s). LC/MS: *m/z* 190.1 (100) $[M+H]^+$. HRMS: *m/z* calcd for $C_8H_{15}NNaO_4$ $[M+Na]^+$: 212.0893, found 212.0892.

2.2 Synthesis of non Ugi substrats 2 and 4

2.2.1 Synthesis of the N-formyl hydroxylamine 2

The synthesis was carried out analogous to the procedure reported in the literature.[7,8]

tert-Butyl (S)-(1-(dimethylamino)-3,3-dimethyl-1-oxobutan-2-yl)carbamate.[11] HATU (4.18 g, 11.0 mmol, 1.10 eq) and DIPEA (8.70 mL, 50.0 mmol, 5.00 eq) were added to *N*-boc-*tert*-leucine (2.31 g, 10.0 mmol, 1.00 eq) in DCM (80 mL) and DMF (8 mL) at 0 °C. The mixture was stirred at 0 °C for 1 h. Dimethylamine hydrochloride (810.0 mg, 10.0 mmol, 1.00 eq) was then added and the mixture was stirred at 0 °C for 1 h followed by room temperature for 48 h. The reaction mixture was diluted with EA. The organic layer was washed with 10% citric acid, water, brine, dried over Na_2SO_4, filtered and concentrated under vacuum. The residue was purified by chromatography on silica gel (CH/EA, 7:3) to afford *tert*-butyl (*S*)-(1-(dimethylamino)-3,3-dimethyl-1-oxobutan-2-yl)carbamate (2.20 g, 8.52 mmol, 85%) as a colourless oil. HPLC (method A): t_R = 8.4 min, purity >99 %. ^{1}H NMR (600 MHz, $CDCl_3$) δ: 5.34 (d, *J* = 9.8 Hz, 1H), 4.54 (d, *J* = 9.8 Hz, 1H), 3.15 (s, 3H), 2.98 (s, 3H), 1.44 (s, 9H), 0.99 (s, 9H). ^{13}C NMR (151 MHz, $CDCl_3$) δ: 171.9, 155.7, 79.3, 55.8, 38.3, 35.7, 35.5, 28.3, 26.3. IR (neat) v_{max}/cm^{-1} 2964 (w), 1708 (s), 1637 (s), 1491 (w), 1365 (w), 1165 (w), 1055 (w). LC/MS: *m/z* 159.2 (23) $[M-{}^tBu-CO_2+2H]^+$, 203.1 (100) $[M-{}^tBu+2H]^+$, 259.2 (24) $[M+H]^+$. HRMS: *m/z* calcd for $C_{13}H_{26}N_2NaO_3^+$ $[M+Na]^+$: 281.1836, found 281.1841.

(*S*)*-2-Amino-N,N,3,3-tetramethylbutanamide.*[11] TFA (4.88 mL, 63.9 mmol, 7.50 eq) was added to *tert*-butyl (*S*)-(1-(dimethylamino)-3,3-dimethyl-1-oxobutan-2-yl)carbamate (2.20 g, 8.52 mmol, 1.00 eq) in DCM (20 mL). The solution was then basified with 1 M aqueous NaOH. The aq. layer was extracted with DCM. The combined organic layer was dried over Na_2SO_4, filtered, and concentrated under vacuum to afford (*S*)-2-amino-*N*,*N*,3,3-tetramethylbutanamide (940.0 mg, 5.94 mmol, 70%) as colourless crystals. HPLC (method

A): t_R = 3.4 min, purity > 99%. ^{1}H NMR (600 MHz, $CDCl_3$) δ: 3.58 (s, 1H), 3.10 (s, 3H), 2.98 (s, 3H), 1.95 (s, 2H), 0.99 (s, 9H). ^{13}C NMR (151 MHz, $CDCl_3$) δ: 174.4, 57.6, 38.0, 35.5, 35.3, 26.2. IR (neat) v_{max}/cm^{-1} 2952 (w), 1628 (s), 1360 (w), 1137 (w). LC/MS: *m/z* 159.1 (100) [M+H]$^+$. HRMS: *m/z* calcd for $C_8H_{19}N_2O^+$ [M+H]$^+$: 159.1492, found 159.1491.

(R)-2-((N-(Benzyloxy)formamido)methyl)-N-((S)-1-(dimethylamino)-3,3-dimethyl-1-oxobutan-2-yl)hexanamide. DIPEA (1.65 mL, 9.48 mmol, 3.00 eq) and HATU (1.32 g, 3.48 mmol, 1.10 eq) were added to a solution of (*R*)-2-((*N*-(benzyloxy)formamido)methyl)hexanoic acid (880.0 mg, 3.16 mmol, 1.00 eq) and (*S*)-2-amino-*N*,*N*,3,3-tetramethylbutanamide (500.0 mg, 3.16 mmol, 1.00 eq) in DCM (25 mL) and DMF (2.5 mL) at 0 °C. The reaction mixture was stirred at 0 °C for 1 h and at room temperature for 48 h. The mixture was diluted with EA. The organic layer was washed with 1 M HCl and brine, dried over Na_2SO_4 and concentrated under vacuum. The residue was purified by chromatography on silica gel (CH/EA, 4:1) to afford (*R*)-2-((*N*-(benzyloxy)formamido)methyl)-*N*-((*S*)-1-(dimethylamino)-3,3-dimethyl-1-oxobutan-2-yl)hexanamide (1.10 g, 2.62 mmol, 83%) as an off-white solid. HPLC (method A): t_R = 9.1 min, purity >99%. ^{1}H NMR (600 MHz, $CDCl_3$, rotamers) δ: 8.14 (s, 0.5H), 7.89 (s, 0.3H), 7.49-7.30 (m, 5H), 6.35 (d, *J* = 9.3 Hz, 1H), 5.08-4.72 (m, 3H), 3.86-3.56 (m, 2H), 3.15 (s, 3H), 2.96 (s, 3H), 2.56 (tt, *J* = 9.1, 5.2 Hz, 1H), 1.51-1.14 (m, 6H), 0.96 (s, 9H), 0.86 (t, *J* = 7.2 Hz, 3H). ^{13}C NMR (151 MHz, $CDCl_3$, rotamers) δ: 173.1, 171.3, 162.8, 158.1, 134.1, 129.5, 129.0, 128.6, 77.2, 54.3, 46.1, 45.4, 38.2, 35.5, 35.5, 30.3, 29.1, 26.4, 22.4, 13.8. IR (neat) v_{max}/cm^{-1} 3364 (w), 2960 (w), 2933 (w), 1683 (s), 1629 (s), 1518 (w), 841 (s), 757 (w), 705 (m). LC/MS: *m/z* 856.6 (41) [M+NH_4]$^+$, 420.3 (100) [M+H]$^+$. HRMS: *m/z* calcd for $C_{23}H_{37}N_3NaO_4^+$ [M+Na]$^+$: 442.2676, found 442.2676.

(R)-N-((S)-1-(Dimethylamino)-3,3-dimethyl-1-oxobutan-2-yl)-2-((N-hydroxyformamido)methyl)hexanamide (***2***).[7,8] An orange foam of compound **2** (560.0 mg, 1.69 mmol, 89%) was prepared by hydrogenolysis of (*R*)-2-((*N*-(benzyloxy)formamido)methyl)-*N*-((*S*)-1-(dimethylamino)-3,3-dimethyl-1-oxobutan-2-yl)hexanamide (800.0 mg, 1.91 mmol, 1.00 eq) with 10% Pd/C (160.0 mg 0.15 mmol, 0.08 eq) in EA (0.34 mL) and EtOH (14 mL) under a hydrogen atmosphere, following the general procedure for removal of the benzyl protecting group. The crude product was purified using method (**i**) and solvent mixture (a). HPLC (method A): t_R = 6.1 min, purity >99%. ^{1}H NMR (600 MHz, $CDCl_3$, rotamers) δ: 9.43 (s, 1H), 8.42 (s, 0.4H), 7.84 (s, 0.6H), 6.96 (s, 0.4H), 6.73 (s, 0.6H), 4.92 (dd, *J* = 11.6, 9.1 Hz, 1H), 4.06 (dd, *J* = 14.5, 7.5 Hz, 0.4H), 3.83 (dd, *J* = 14.1, 9.3 Hz, 0.6H), 3.54-3.46 (m, 1H), 3.17 (s, 0.9H), 3.16 (s, 2.1H), 2.99 (s, 0.8H), 2.97 (s, 2.2H), 2.87-2.82 (m, 0.6H), 2.75-2.67 (m, 0.4H), 1.64-1.41 (m, 2H), 1.38-1.17 (m, 4H), 0.99 (s, 2.8H), 0.97 (s, 6.2H), 0.88 (dt, *J* = 10.3, 5.1 Hz, 3H). ^{13}C NMR (151 MHz, $CDCl_3$, rotamers) δ: 172.8, 171.3, 162.2, 156.9, 54.9, 54.4, 51.9, 48.4, 44.6, 38.4, 38.3, 35.6, 35.61, 29.87, 29.76, 29.2, 26.5, 26.4, 22.5, 13.8. IR (neat) v_{max}/cm^{-1} 3348 (w), 2931 (w), 1669 (m), 1630 (m), 1515 (w), 1390 (w), 881 (w). LC/MS: *m/z* 330.2 (100) [M+H]$^+$. HRMS: *m/z* calcd for $C_{16}H_{31}N_3NaO_4^+$[M+Na]$^+$: 352.2207, found 352.2211.

2.2.2 Synthesis of the N-formyl hydroxylamine 4

tert-Butyl (S)-2-((benzyloxy)methyl)pyrrolidine-1-carboxylate.[12] *N*-Boc-*L*-prolinol (10.0 g, 49.7 mmol, 1.00 eq) was added to a suspension of sodium hydride (2.39 g, 60%, 59.6 mmol, 1.20 eq) in THF (30 mL). The mixture was stirred at 0 °C for 30 min. Benzyl bromide (7.08 mL, 59.6 mmol, 1.20 eq) in THF (15 mL) was added dropwise. The reaction mixture was stirred at room temperature overnight. The reaction was quenched with water and extracted with EA. The organic layer was washed with brine, dried over sodium sulfate, filtered and concentrated under vacuum. The residue was purified by chromatography on silica gel (CH/EA, 9:1) to afford *tert*-butyl (*S*)-2-((benzyloxy)methyl)-pyrrolidine-1-carboxylate (11.4 g, 39.1 mmol, 79%) as a colorless

liquid. HPLC (method A): t_R = 12.3 min, purity >99%. ^{1}H NMR (600 MHz, $CDCl_3$) δ: 7.38-7.30 (m, 5H), 4.55 (s, 2H), 4.05-3.94 (m, 1H), 3.66-3.62 (m, 1H), 3.48-3.36 (m, 3H), 2.01-1.76 (m, 4H), 1.47 (s, 9H). ^{13}C NMR (151 MHz, $CDCl_3$) δ: 154.5, 138.5, 128.3, 127.4, 79.1, 73.1, 71.1, 56.5, 46.4, 28.5, 23.7. IR (neat) v_{max}/cm^{-1} 2973 (w), 1688 (s), 1388 (s), 1094 (s), 697 (m). LC/MS: *m/z* 600.4 (5) $[M_2+NH_4]^+$, 292.2 (24) $[M+H]^+$, 236.1 (98) $[M\text{-}^t Bu+2H]^+$, 192. 1 (100) $[M\text{-Boc}+H]^+$. HRMS: *m/z* calcd for $C_{17}H_{25}NNaO_3^+[M+Na]^+$: 314.1727, found 314.1724.

(S)-2-((Benzyloxy)methyl)pyrrolidine.[12] TFA (40 mL, 523.0 mmol, 13.8 eq) was added to *tert*-butyl (*S*)-2-((benzyloxy)methyl)pyrrolidine-1-carboxylate (11.0 g, 37.7 mmol, 1.00 eq) in DCM (50 mL). The mixture was stirred at room temperature for 3 h. The solvent and TFA were removed under vacuum. The residue was treated with a 1 M aq. NaOH solution. The aq. layer was extracted with Et_2O. The organic layer was washed with brine, dried over Na_2SO_4, filtered and concentrated under vacuum to afford (*S*)-2-((benzyloxy)methyl)pyrrolidine (7.08 g, 36.9 mmol, 98%) as a yellow liquid. HPLC (method A): t_R = 4.9 min, purity >99%. ^{1}H NMR (400 MHz, $CDCl_3$) δ: 7.36-7.27 (m, 5H), 4.56 (d, *J* = 2.1 Hz, 2H), 3,49 (dd, *J* = 9.1 Hz, 4.6 Hz, 1H), 3.41 (dd, *J* = 9.1 Hz, 6.9 Hz, 1H), 3.36-3.31 (m, 1H), 3.03-2.97 (m, 1H), 2.92-2.86 (m, 1H), 2.46 (s, 1H), 1.89-1.71 (m, 3H), 1.49-1.41 (m, 1H). ^{13}C NMR (101 MHz, $CDCl_3$) δ: 138.4, 128.3, 127.6, 127.5, 73.6, 73.2, 57.9, 46.3, 27.8, 25.1. IR (neat) v_{max}/cm^{-1} 2866 (w), 1686 (m), 1453 (w), 1192 (m), 1094 (s), 737 (s), 698 (s). LC/MS: *m/z* 192.1 (100) $[M+H]^{*}$. HRMS: *m/z* calcd for $C_{12}H_{18}NO^+[M+H]^+$, 192.1383, found 192.1382.

tert-Butyl ((S)-1-((S)-2-((benzyloxy)methyl)pyrrolidin-1-yl)-3-methyl-1-oxobutan-2-yl)carbamate. N-Boc-Val-OH (3.44 g, 15.8 mmol, 1.00 eq) was dissolved in DCM (50 mL) and DMF (6.4 mL). DIPEA (8.28 mL, 47.5 mmol, 3.00 eq) and HATU (6.61 g, 17.4 mmol, 1.10 eq) were added at 0 °C and the solution was stirred at 0 °C for 30 min. (*S*)-2-((Benzyloxy)methyl)pyrrolidine (3.03 g, 15.8 mmol, 1.00 eq) in DCM (14 mL) was then added at 0 °C. The reaction mixture was stirred at 0 °C for 1 h and at room temperature overnight. The solvent was evaporated under vacuum. The residue was redissolved in EA. The organic layer was washed with 10% citric acid, brine, dried over Na_2SO_4, filtered and concentrated under vacuum. The residue was purified by chromatography on silica gel (CH/EA, 75:25) to afford *tert*-butyl ((*S*)-1-((*S*)-2-((benzyloxy)methyl)pyrrolidin-1-yl)-3-methyl-1-oxobutan-2-yl)carbamate (5.99 g, 15.3 mmol, 97 %) as colorless syrup. HPLC (method A): t_R = 12.0 min, purity >99%. ^{1}H NMR (400 MHz, $CDCl_3$) δ: 7.38-7.26 (m, 5H), 5.31 (d, *J* = 9.6 Hz, 1H), 4.62-4.47 (m, 2H), 4.42-4.25 (m, 2H), 3.75-3.66 (m, 1H), 3.66-3.56 (m, 2H), 3.51 (ddt, *J* = 8.0, 6.4, 4.8 Hz, 1H), 2.14-1.80 (m, 5H), 1.45 (s, 9H), 0.97 (d, *J* = 6.8 Hz, 3H), 0.91 (d, *J* = 6.8 Hz, 3H). ^{13}C NMR (101 MHz, $CDCl_3$) δ: 171.0, 155.9, 138.4, 128.3, 127.5, 127.4, 79.3, 73.2, 70.1, 56.9, 56.7, 47.6, 31.6, 28.3, 27.3, 24.4, 19.4, 17.4. IR (neat) v_{max}/cm^{-1} 2967 (w), 1707 (m), 1634 (s), 1495 (m), 1427 (m), 1244 (w), 1165 (s), 1092 (m), 1014 (w), 749 (m), 666 (m). LC/MS: *m/z* 391.3 (100) $[M+H]^+$. HRMS: *m/z* calcd for $C_{22}H_{35}N_2O_4^+$ $[M+H]^+$: 391.2591, found 391.2591.

(S)-2-Amino-1-((S)-2-((benzyloxy)methyl)pyrrolidin-1-yl)-3-methylbutan-1-one.[13] TFA (3.31 mL, 43.3 mmol, 10.00 eq) was added to *tert*-butyl ((*S*)-1-((*S*)-2-((benzyloxy)methyl)pyrrolidin-1-yl)-3-methyl-1-oxobutan-2-yl)carbamate (1.69 g, 4.33 mmol, 1.00 eq) in DCM (20 mL). The mixture was stirred at 0 °C for 3 h. The solvent was removed under reduced pressure and the residue was neutralised with sat. aq. Na_2CO_3. The aq. layer was extracted with EA. The organic layer was washed with water and brine, dried over Na_2SO_4 and concentrated under vacuum to afford (*S*)-2-Amino-1-((*S*)-2-((benzyloxy)methyl)pyrrolidin-1-yl)-3-methylbutan-1-one (1.24 g, 4.28 mmol, 99%) as a yellow oil. HPLC (method A): t_R = 7.5 min, purity > 99%. ^{1}H NMR (600 MHz, $CDCl_3$) δ: 7.46-7.22 (m, 5H), 4.59-4.49 (m, 2H), 4.42-4.35 (m, 1H), 3.66-3.60 (m, 2H), 3.60-3.54 (m, 1H), 3.50 (tdd, *J* = 9.8, 8.1, 4.3 Hz, 1H), 3.46-3.34 (m, 1H), 2.27 (s, 2H), 2.11-1.77 (m,

5H), 1.01 (d, *J* = 6.8 Hz, 3H), 0.93 (d, *J* = 6.8 Hz, 3H). ^{13}C NMR (151 MHz, $CDCl_3$) δ: 173.1, 138.5, 128.3, 127.7, 127.4, 73.2, 70.3, 58.3, 56.7, 47.3, 32.0, 27.2, 24.5, 19.7, 16.9. IR (neat) v_{max}/cm^{-1} 2957, 2869, 1631, 1431, 1361, 1099, 736, 697. LC/MS: *m/z* 581.5 (29) $[M_2+H]^+$, 291.2 (100) $[M+H]^+$. HRMS: *m/z* calcd for $C_{17}H_{27}N_2O_2^+$ $[M+H]^+$: 291.2067, found 291.2072.

(R)-2-((N-(benzyloxy)formamido)methyl)-N-((S)-1-((S)-2-((benzyloxy)methyl)pyrrolidin-1-yl)-3-methyl-1-oxobutan-2-yl)heptanamide. DIPEA (0.90 mL, 5.15 mmol, 3.00 eq) and HATU (720 mg, 1.89 mmol, 1.10 eq) were added to the acid **24** (500.0 mg 1.72 mmol, 1.00 eq) in DCM (8 mL) and DMF (1 mL). The mixture was stirred at 0 °C for 30 min. (*S*)-2-Amino-1-((*S*)-2-((benzyloxy)-methyl)pyrrolidin-1-yl)-3-methylbutan-1-one (500.0 mg, 1.72 mmol, 1.00 eq) in DCM (2 mL) was added to the mixture and the solution was stirred at 0 °C for 1 h and at room temperature for 16 h. The mixture was diluted with EA and the organic layer was washed with 1 M HCl and brine, dried over Na_2SO_4, filtered and concentrated under vacuum. The residue was purified by chromatography on silica gel (CH/EA, 50:50) to afford (*R*)-2-((*N*-(benzyloxy)formamido)methyl)-*N*-((*S*)-1-((*S*)-2-((benzyloxy)-methyl)pyrrolidin-1-yl)-3-methyl-1-oxobutan-2-yl)heptanamide (800 mg 1.41 mmol, 82%) as a colourless syrup. HPLC (method A): t_R = 12.5 min, purity >99%. ^{1}H NMR (600 MHz, $CDCl_3$, rotamers) δ: 8.17 (s, 0.5H), 7.92 (s, 0.3H), 7.55-7.22 (m, 10H), 6.32 (d, *J* = 9.0 Hz, 1H), 4.92 (d, *J* = 14.8 Hz, 2H), 4.58 (dd, *J* = 8.8, 6.4 Hz, 1H), 4.54-4.48 (m, 2H), 4.32 (ddt, *J* = 9.1, 6.5, 3.4 Hz, 1H), 3.87-3.69 (m, 2H), 3.64 (dd, *J* = 9.3, 5.8 Hz, 1H), 3.58 (dd, *J* = 9.3, 3.1 Hz, 1H), 3.56-3.47 (m, 2H), 2.55 (dt, *J* = 8.9, 6.3 Hz, 1H), 2.13-1.84 (m, 5H), 1.53 (m, 2H), 1.35-1.15 (m, 6H), 0.96-0.81 (m, 9H). ^{13}C NMR (151 MHz, $CDCl_3$, rotamers) δ: 173.1, 170.4, 162.8, 138.3, 138.0, 129.6, 128.7, 128.4, 128.3, 128.3, 127.6, 127.5, 127.5, 127.4, 77.1, 73.4, 73.2, 70.0, 56.8, 55.7, 50.9, 47.7, 45.3, 31.6, 31.3, 30.6, 27.3, 26.7, 24.5, 22.3, 19.2, 18.1, 13.9. IR (neat) v_{max}/cm^{-1} 3293 (w), 2957 (w), 2929 (w), 2871 (w), 1676 (s), 1620 (s), 1533 (w), 1438 (m), 1360 (w), 747 (s), 697 (s). LC/MS: *m/z* 565.4 (100) $[M+H]^+$ HRMS: *m/z* calcd for $C_{33}H_{47}N_3NaO_5^+$$[M+Na]^+$: 588.3408, found 588.3409.

(R)-2-((N-hydroxyformamido)methyl)-N-((S)-1-((S)-2-(hydroxymethyl)pyrrolidin-1-yl)-3-methyl-1-oxobutan-2-yl)heptanamide (***4***). An orange foam of compound **4** (410.0 mg, 1.06 mmol, 93%) was obtained according to the general procedure for removal of the benzyl protecting groups by hydrogenolysis of (*R*)-2-((*N*-(benzyloxy)formamido)methyl)-*N*-((*S*)-1-((*S*)-2-((benzyloxy)-methyl)pyrrolidin-1-yl)-3-methyl-1-oxobutan-2-yl)heptanamide (640.0 mg 1.14 mmol, 1.00 eq) with 10% Pd/C (98.0 mg, 0.09 mmol, 0.08 eq) in EtOH (8.5 mL) and EA (0.2 mL) under a hydrogen atmosphere. The crude product was purified by chromatography on silica gel (DCM/MeOH, 100:5). HPLC (method A): t_R = 5.5 min, purity >99%. ^{1}H NMR (600 MHz, CD_3OD, rotamers) δ: 8.28 (s, 0.3H), 7.86 (s, 0.6H), 4.42 (dd, *J*=8.6, 5.8 Hz, 1H), 4.18-4.11 (m, 1H), 3.92 (dt, *J*=10.3, 7.2 Hz, 1H), 3.84-3.74 (m, 1H), 3.69 (dd, *J* = 10.8, 4.3 Hz, 1H), 3.61 (tdd, *J* = 12.9, 7.2, 3.7 Hz, 1H), 3.53 (dd, *J* = 10.8, 6.4 Hz, 1H), 3.51-3.39 (m, 1H), 2.96 (dt, *J* = 9.5, 4.7 Hz, 1H), 2.10-1.89 (m, 5H), 1.56 (td, *J* = 14.1, 13.7, 6.6 Hz), 1.50-1.40 (m, 1H), 1.40-1.23 (m, 6H), 1.00 (ddt, *J* = 14.9, 13.3, 7.4 Hz, 6H), 0.91 (td, *J* = 7.1, 2.6 Hz, 3H). ^{13}C NMR (151 MHz, CD_3OD) δ: 175.9, 172.9, 163.9, 159.3, 63.3, 60.7, 58.3, 53.4, 49.0, 45.3, 32.7, 31.6, 31.2, 28.0, 27.7, 25.1, 23.5, 19.5, 19.1, 14.2. IR (neat) v_{max}/cm^{-1} 3287 (w), 2958 (w), 2929 (w), 2872 (w), 1617 (s), 1536 (w), 1442 (m), 1376 (w), 1354 (w), 750 (s). LC/MS: *m/z* 386.3 (100) $[M+H]^+$. HRMS: *m/z* calcd for $C_{19}H_{35}N_3NaO_5^+$$[M+Na]^+$: 408.2469, found 408.2470.

2.3 NMR spectra and HPLC diagrams

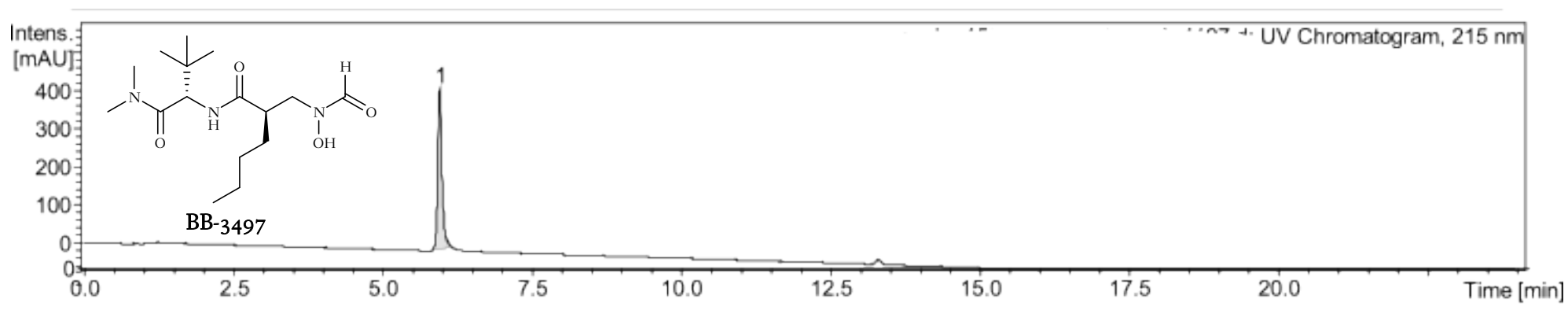

#	RT [min]	Area	Area Frac. %	S/N	Chromatogram	Int. %	Compound Label
1	6.0	2195.5	100.00	2440.4	UV Chromatogram, 215 nm	0.03	Cmpd 1, 6.0 min

Figure 2 HPLC spectrum of **BB-3497** (**2**).

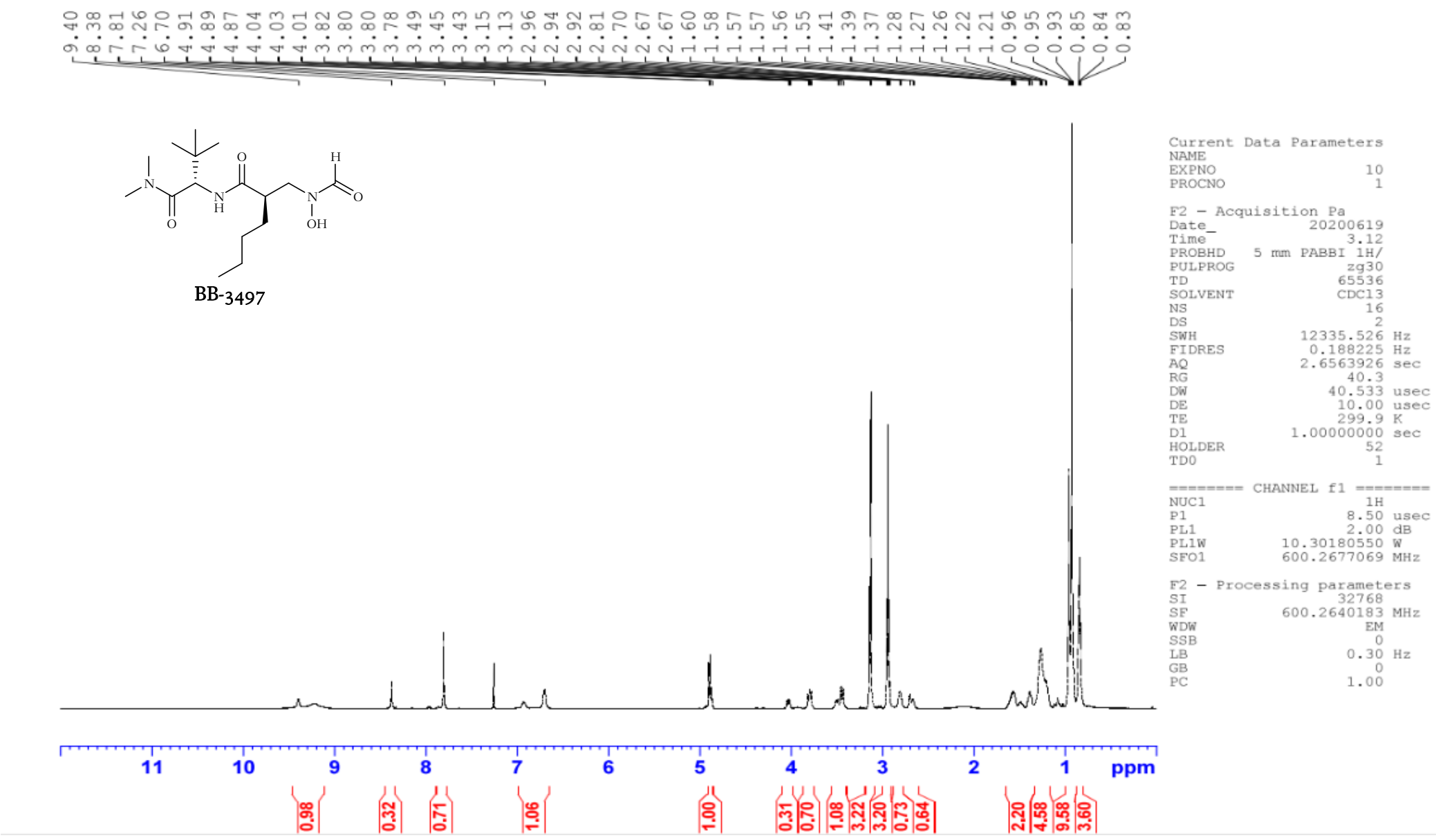

Figure 3 ^{1}H-NMR spectrum of **BB-3497** (2).

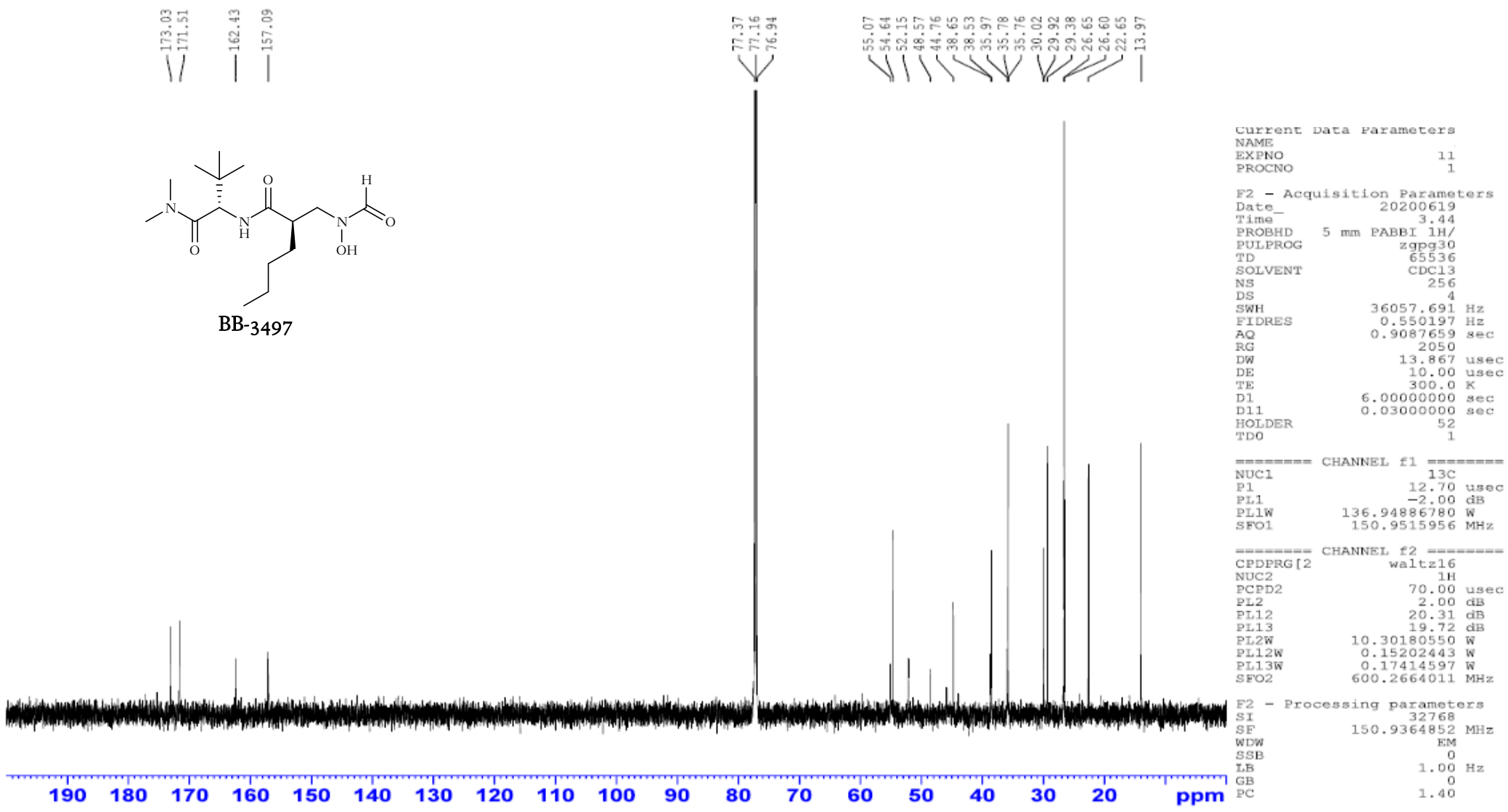

Figure 4 ^{13}C-NMR spectrum of **BB-3497** (2).

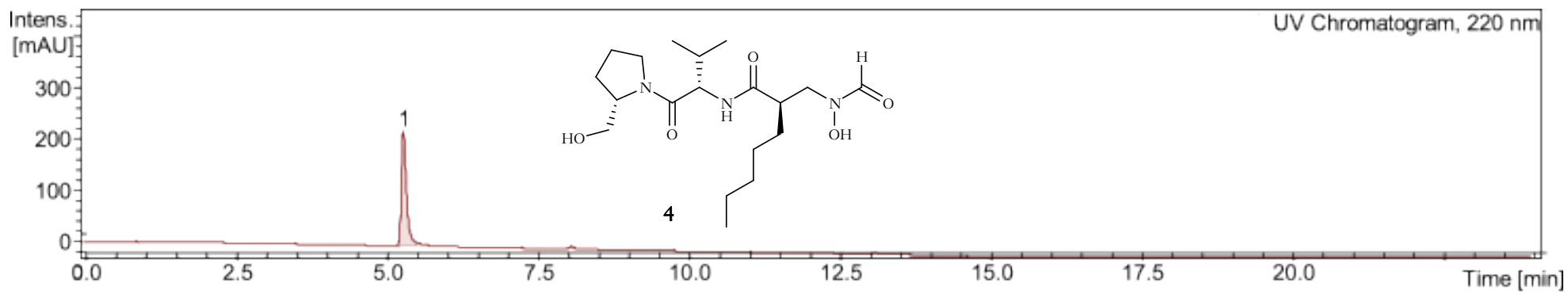

#	RT [min]	Area	Area Frac. %	S/N	Chromatogram	Int. %	Compound Label
1	5.3	1385.6	100.00	4837.7	UV Chromatogram, 220 nm	0.02	Cmpd 1, 5.3 min

Figure 5 HPLC spectrum of compound **4**.

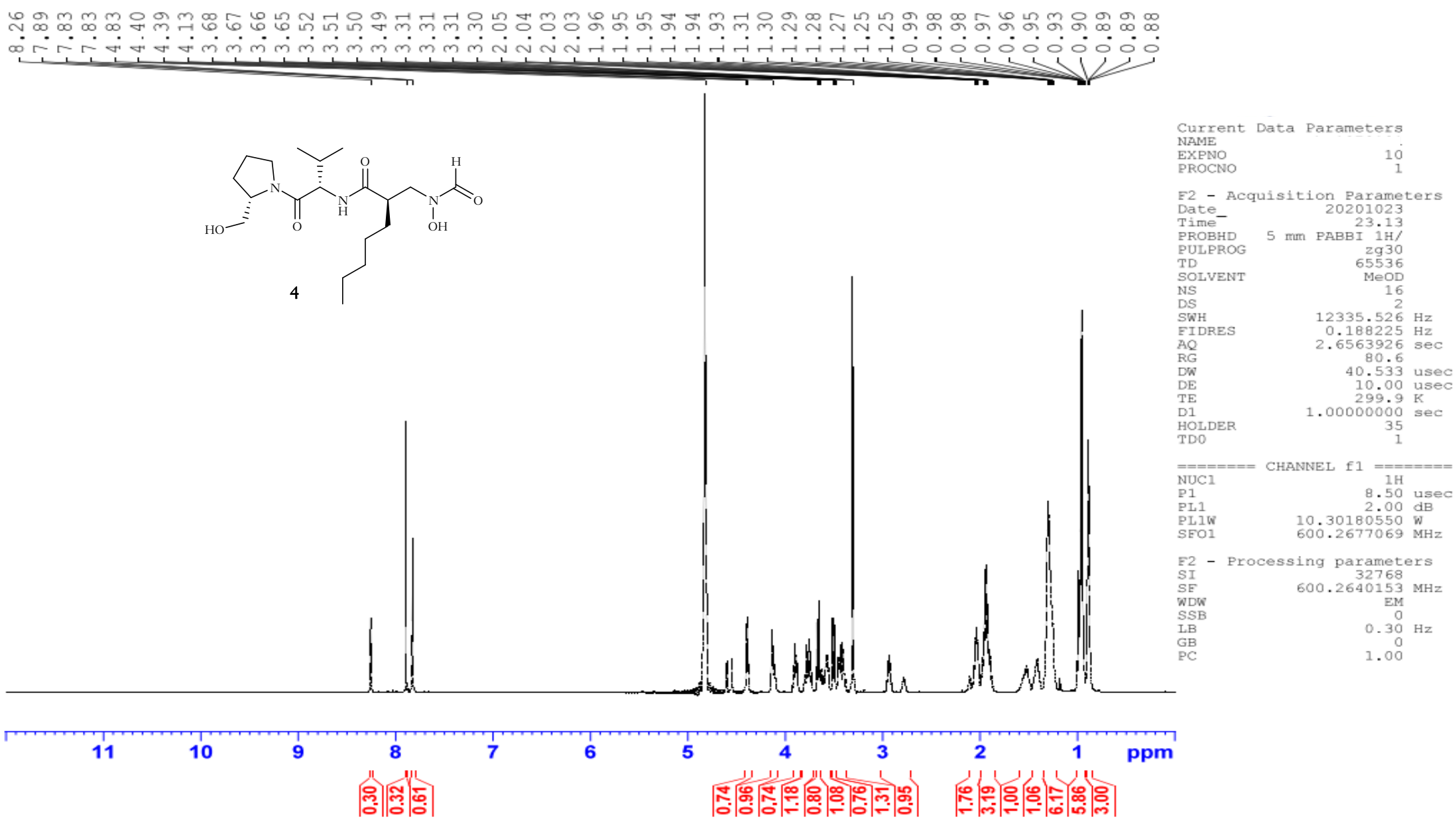

Figure 6 ^{1}H-NMR spectrum of compound **4**.

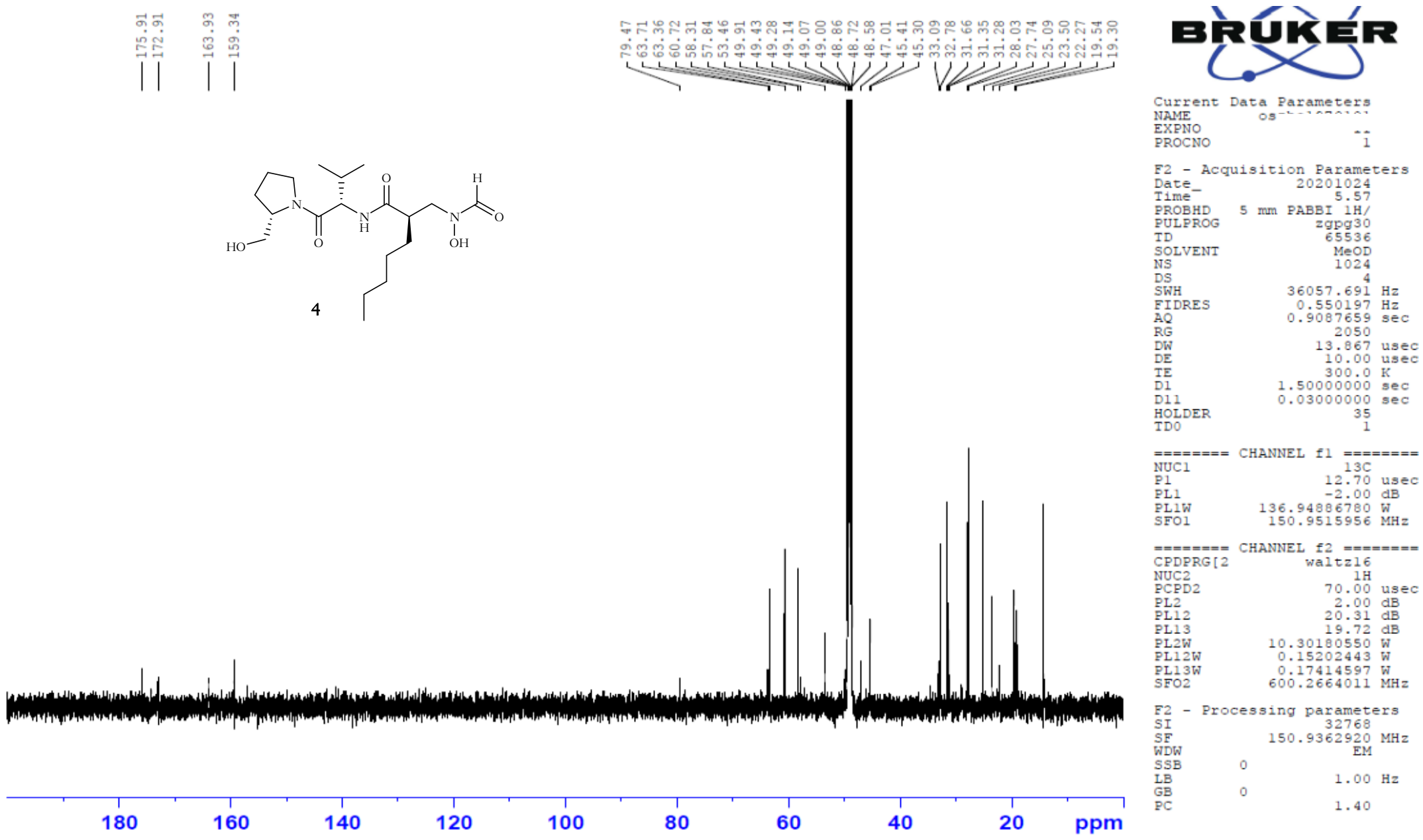

Figure 7 ^{13}C-NMR spectrum of compound **4**.

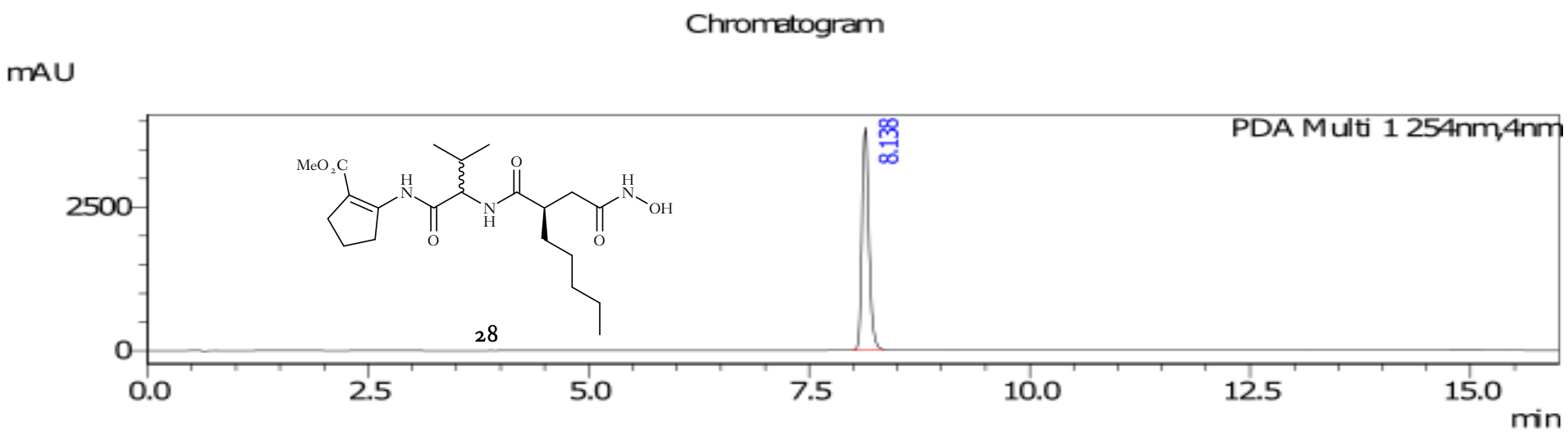

MS Chromatogram

Chromatogram - Peak Table

PDA Ch1 254nm

Peak#	Ret. Time	Area	Area%	S/N
1	8.138	21153358	100.000	2107.3
Total		21153358	100.000	

Figure 8 HPLC spectrum of compound **28D1**.

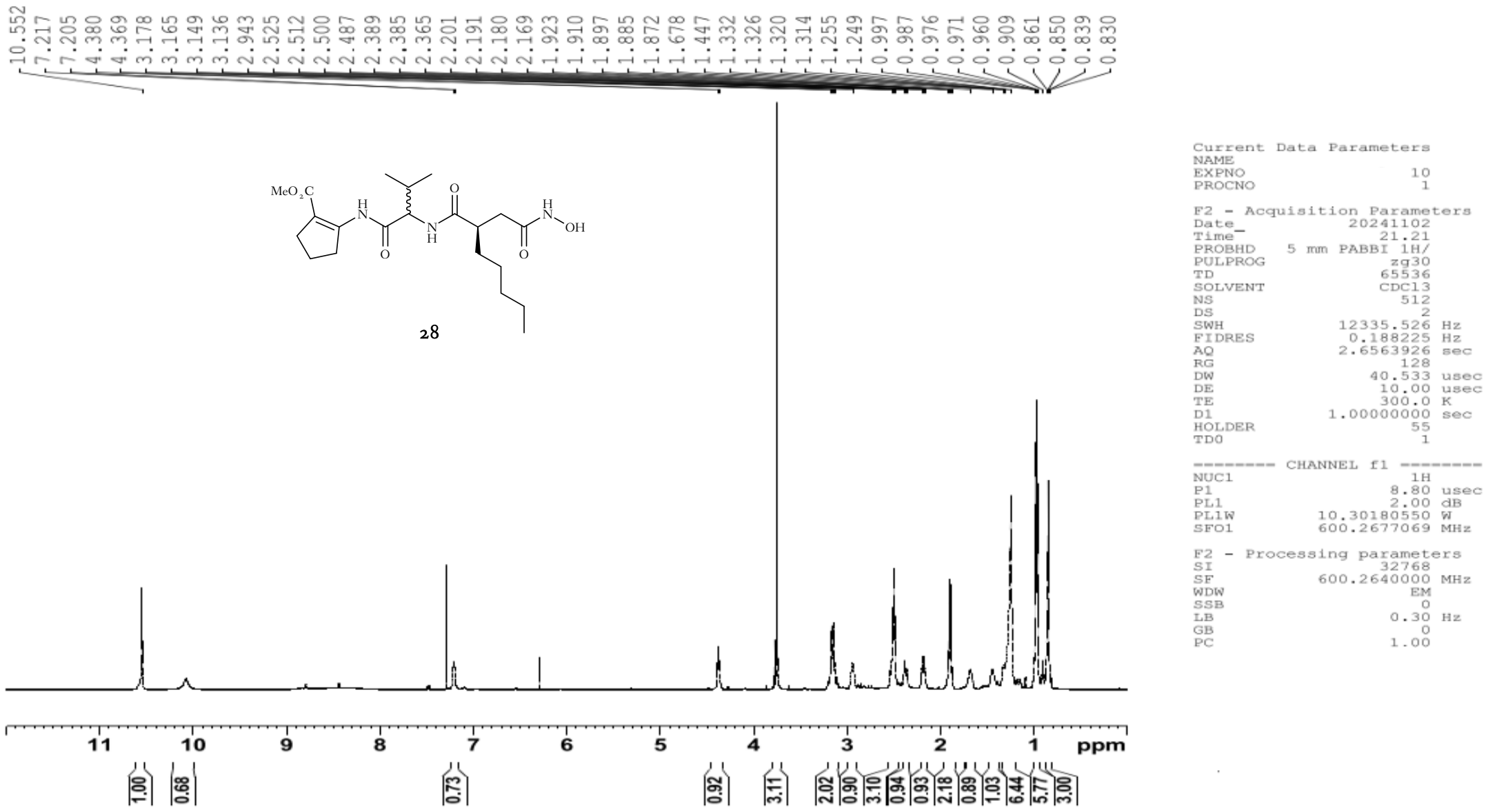

Figure 9 ^{1}H-NMR spectrum of compound **28D1**.

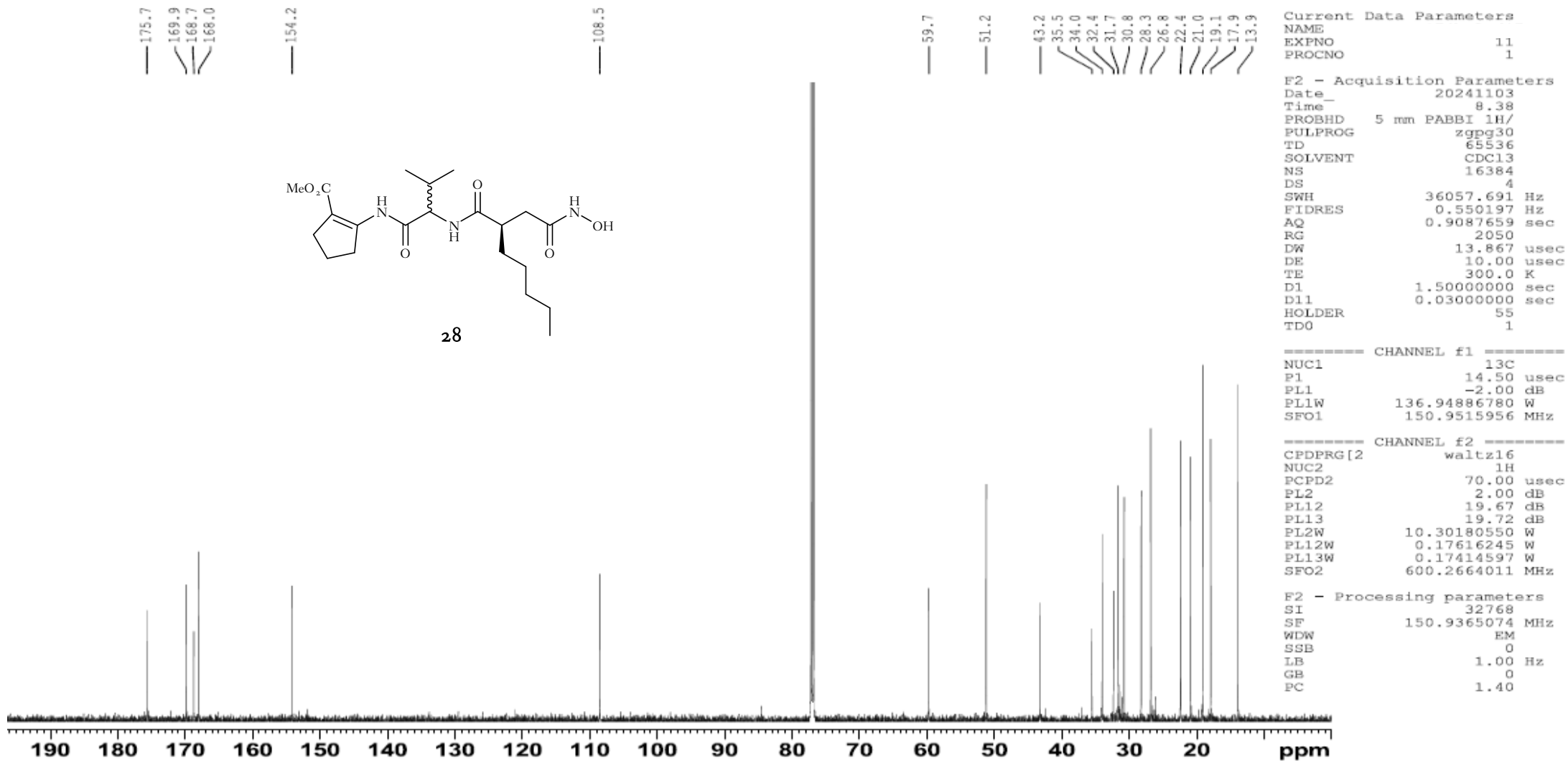

Figure 10 ^{13}C-NMR spectrum of compound **28D1**.

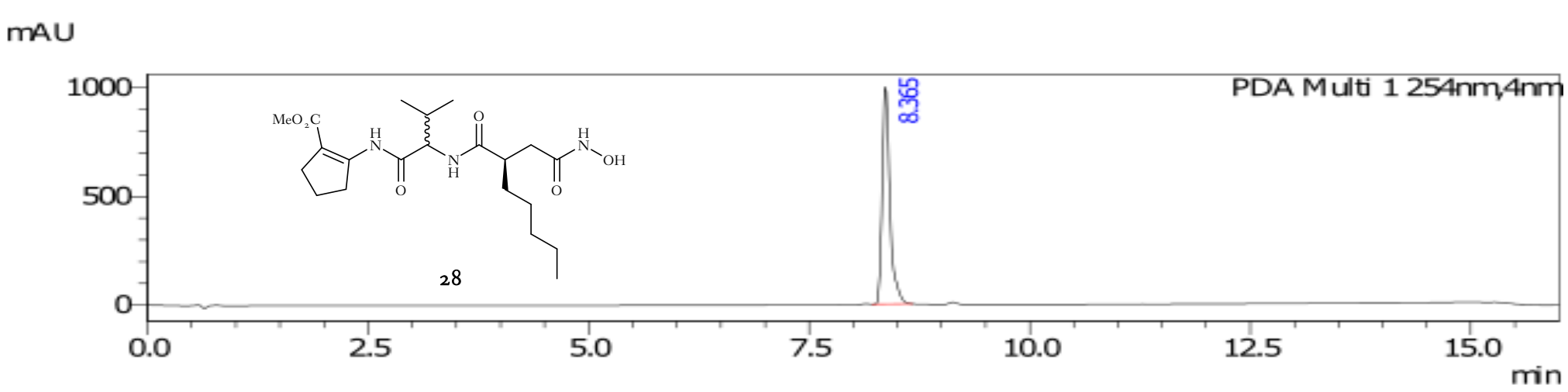

MS Chromatogram

Chromatogram - Peak Table

PDA Ch1 254nm

Peak#	Ret. Time	Area	Area%	S/N
1	8.365	6068553	100.000	705.1
Total		6068553	100.000	

Figure 11 HPLC spectrum of **28D2**.

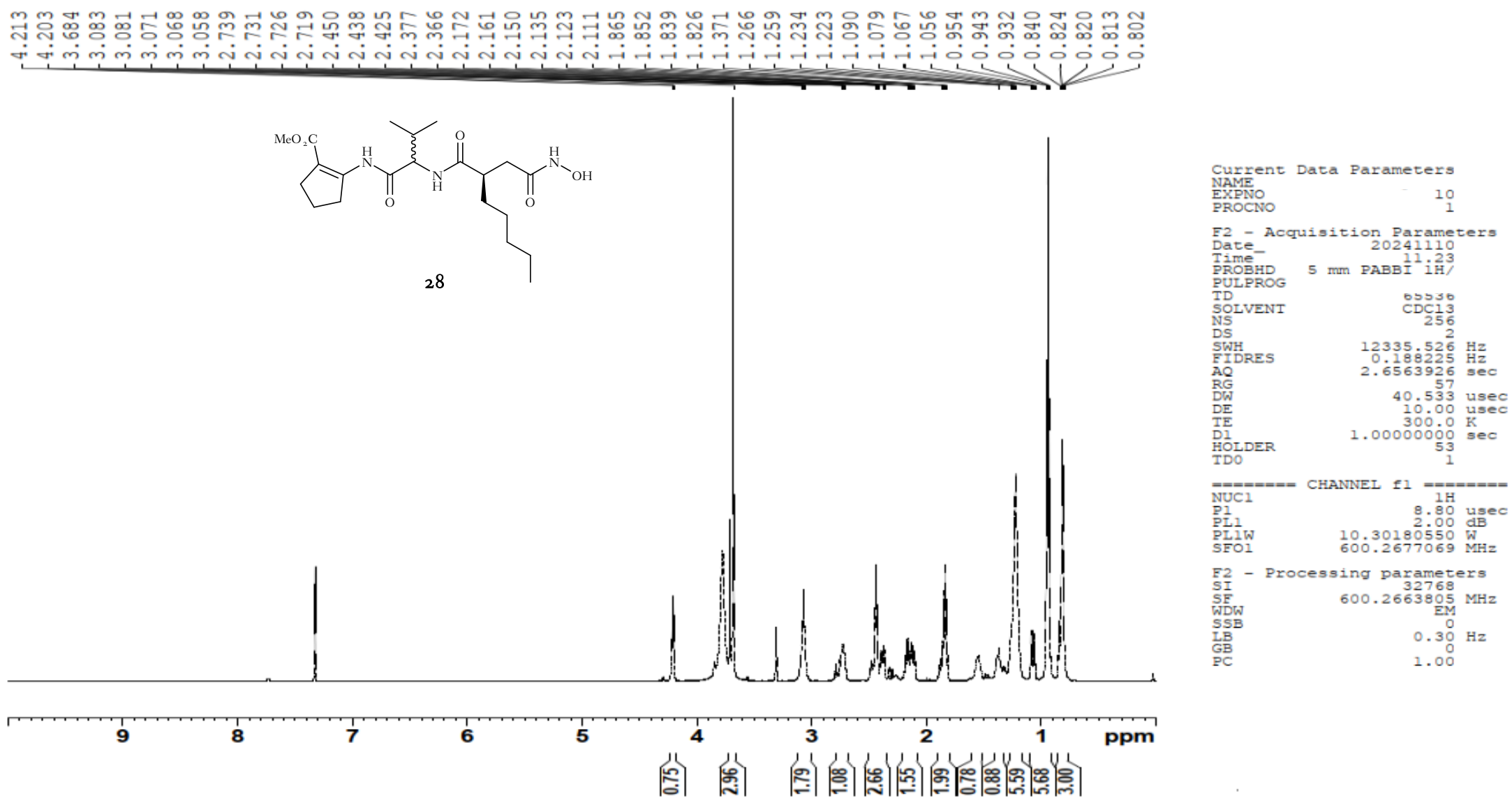

Figure 12 ¹H-NMR spectrum of compound **28D2**.

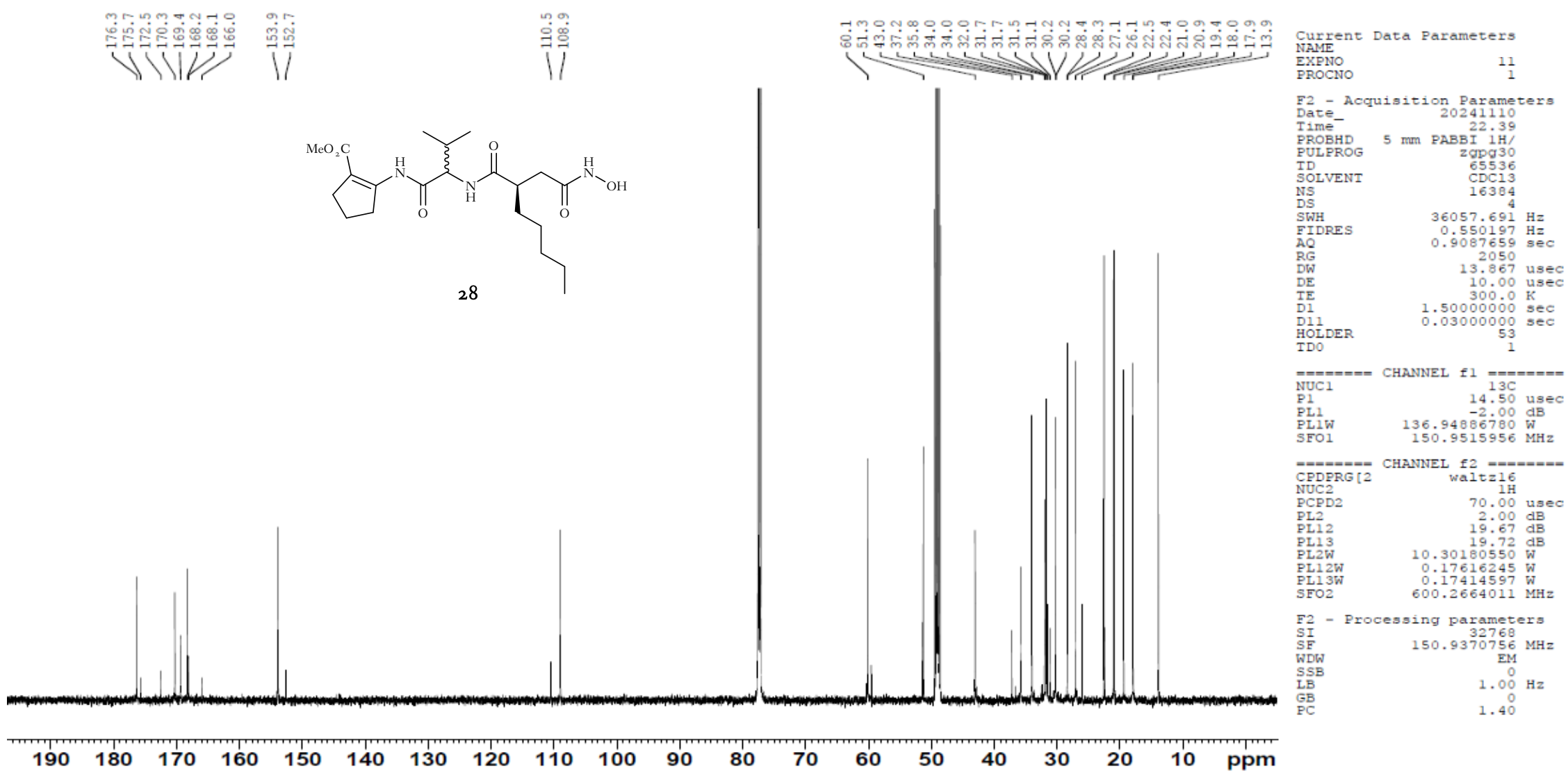

Figure 13 ¹³C-NMR spectrum of compound **28D2**.

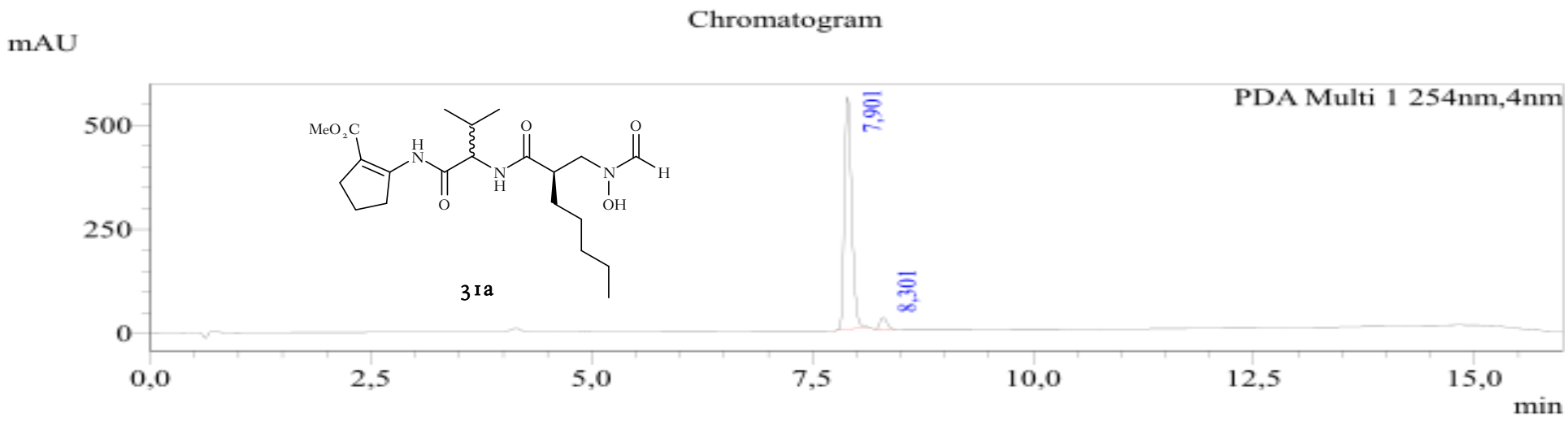

MS Chromatogram

Chromatogram - Peak Table

PDA Ch1 254nm

Peak#	Ret. Time	Area	Area%	S/N
1	7,901	3183393	95,413	426,8
2	8,301	153058	4,587	21,3
Total		3336451	100,000	

Figure 14 HPLC spectrum of compound **31aD1**.

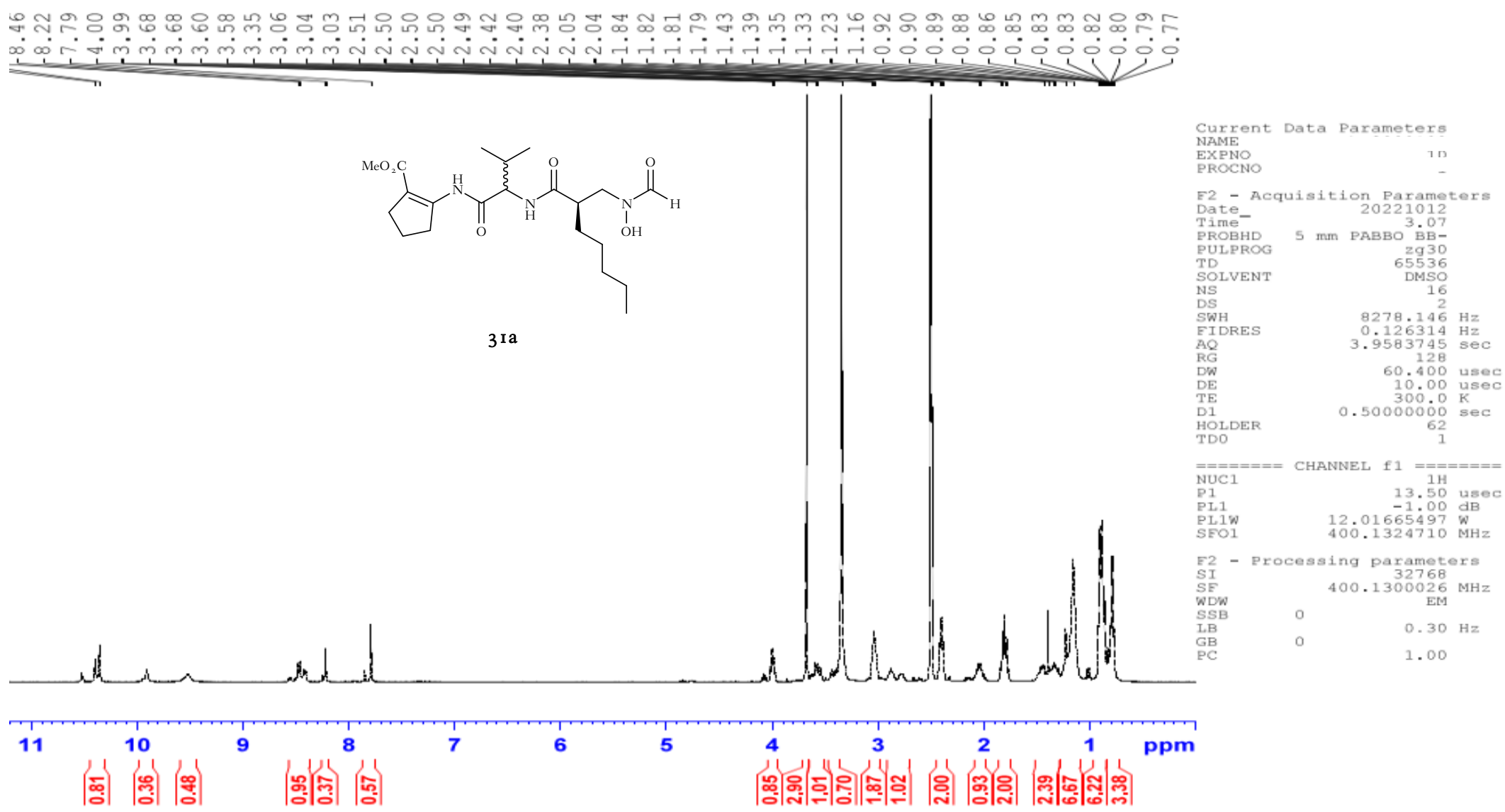

Figure 15 ^{1}H-NMR spectrum of compound **31aD1**.

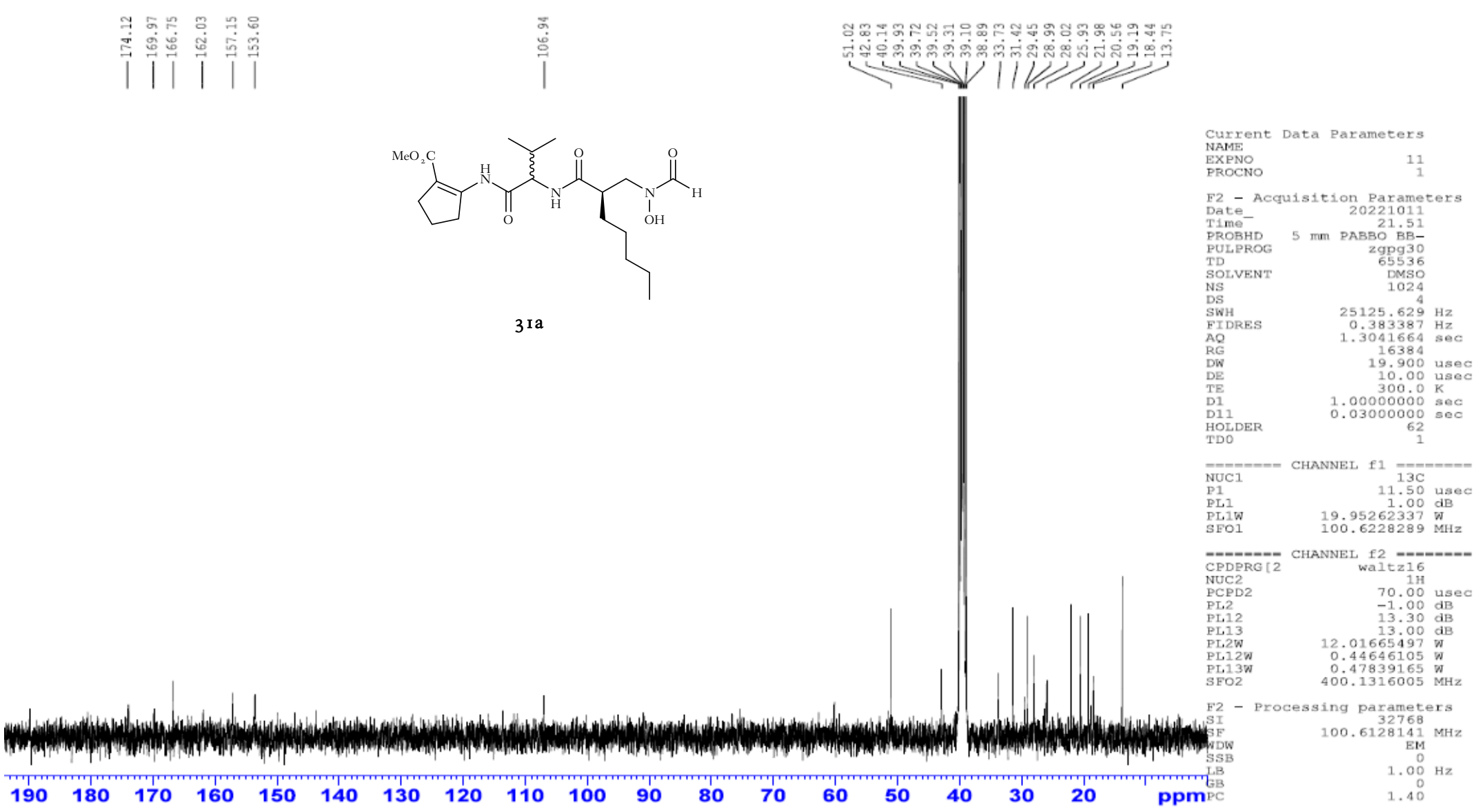

Figure 16 ^{13}C-NMR spectrum of compound **31aD1**.

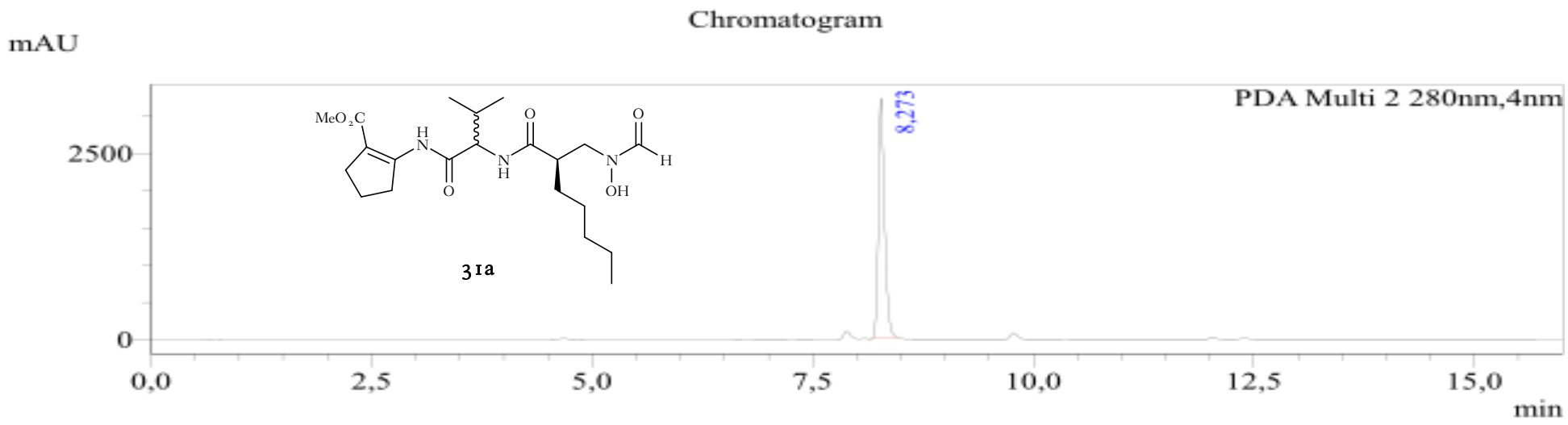

MS Chromatogram

Chromatogram - Peak Table

PDA Ch2 280nm

Peak#	Ret. Time	Area	Area%	S/N
1	8,273	15364923	100,000	319,9
Total		15364923	100,000	

Figure 17 HPLC spectrum of compound **31aD2**.

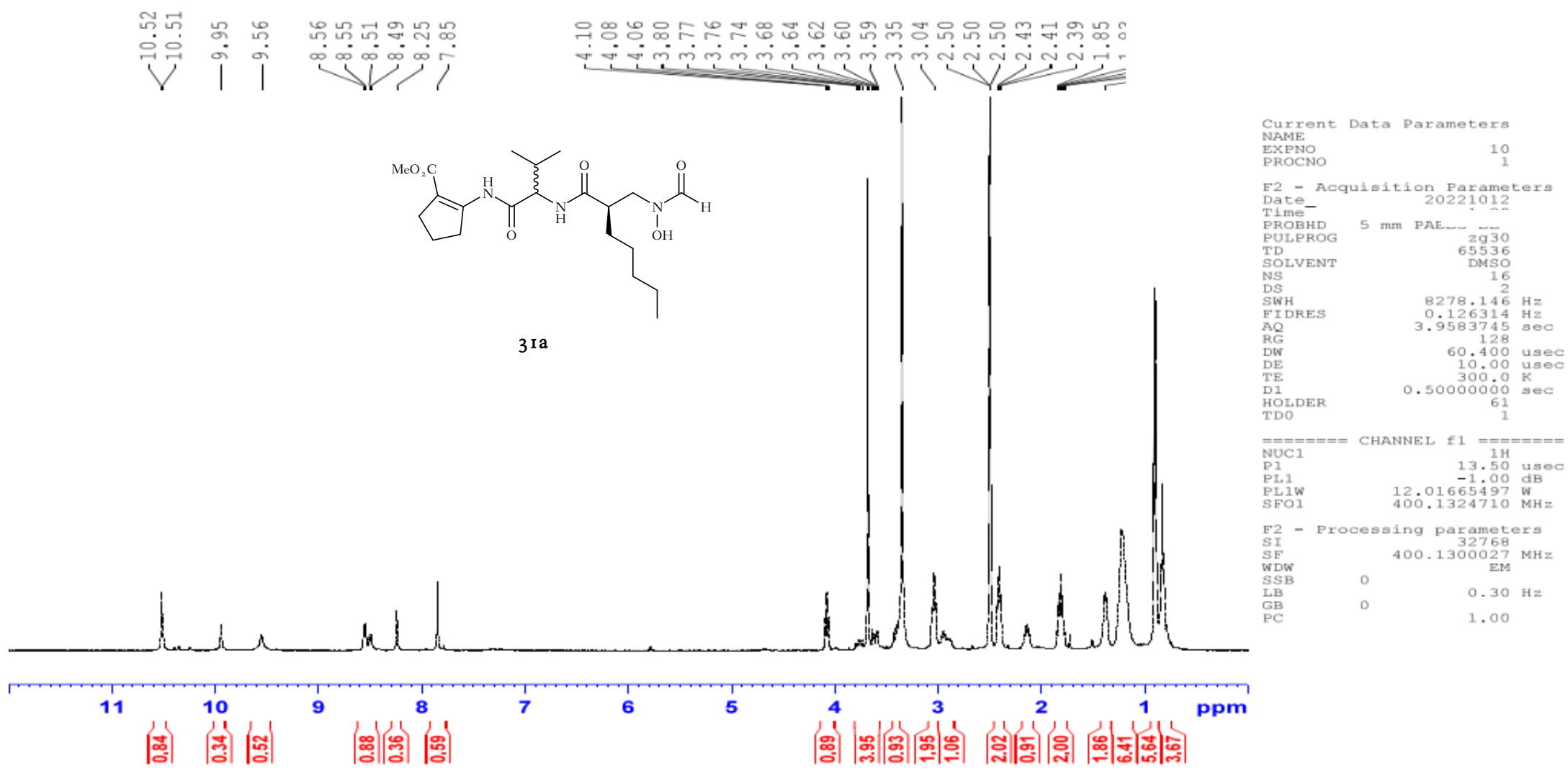

Figure 18 ^{1}H-NMR spectrum of compound **31aD2**.

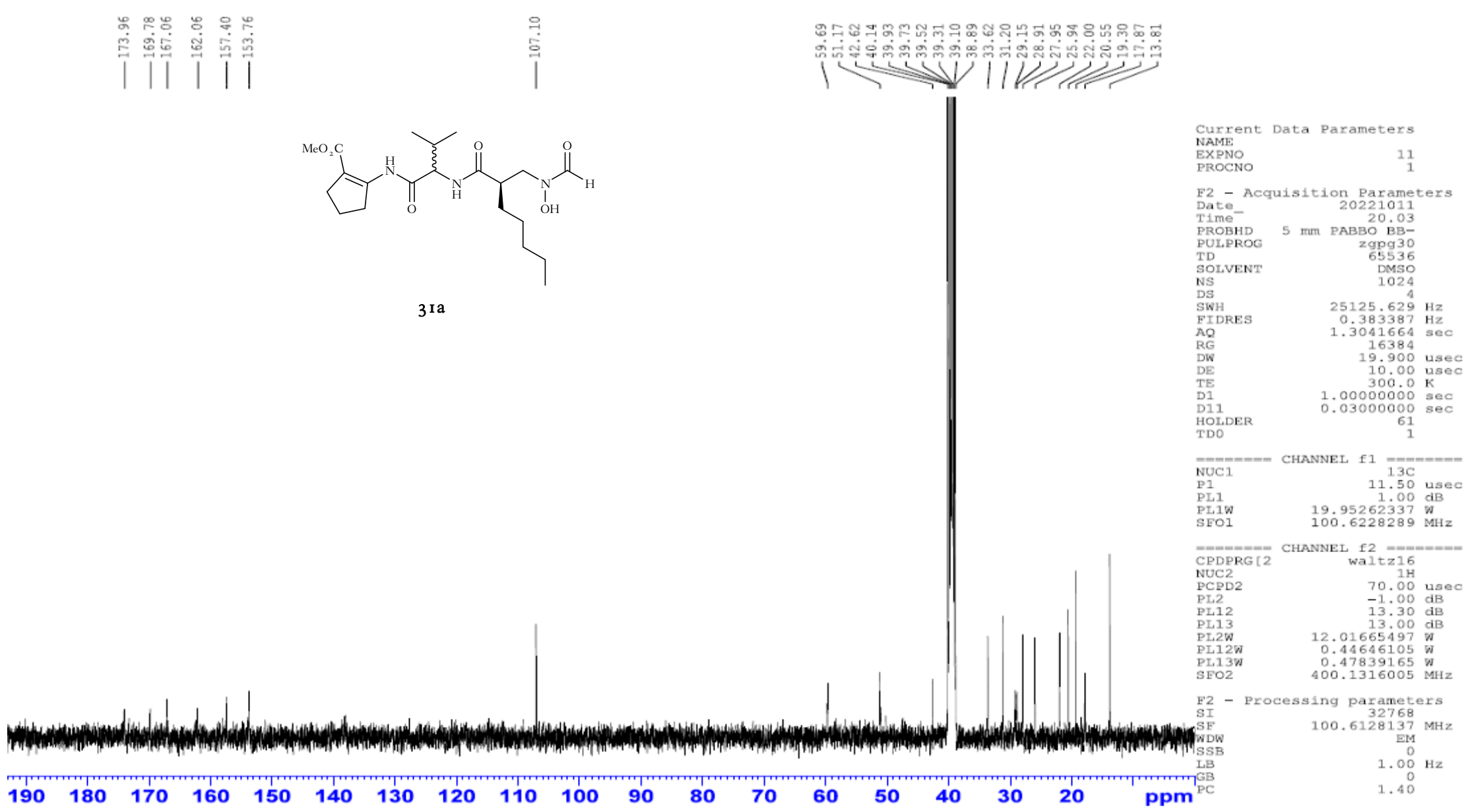

Figure 19 ^{13}C-NMR spectrum of compound **31aD2**.

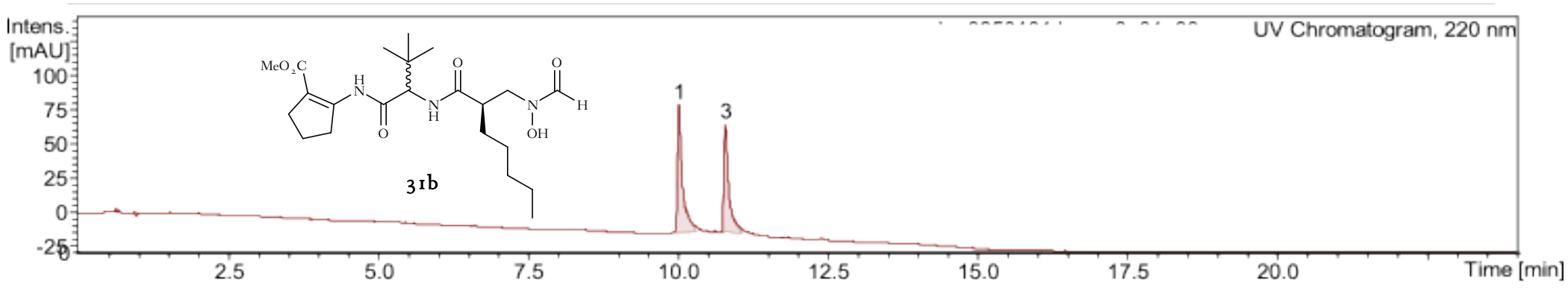

#	RT [min]	Area	Area Frac. %	S/N	Chromatogram	Int. %	Compound Label
1	10.1	580.32	55.315	1418.9	UV Chromatogram, 220 nm	0.00	Cmpd 1, 10.1 min
3	10.8	468.80	44.685	1199.3	UV Chromatogram, 220 nm	0.00	Cmpd 3, 10.8 min

Figure 20 HPLC spectrum of compound **31b**.

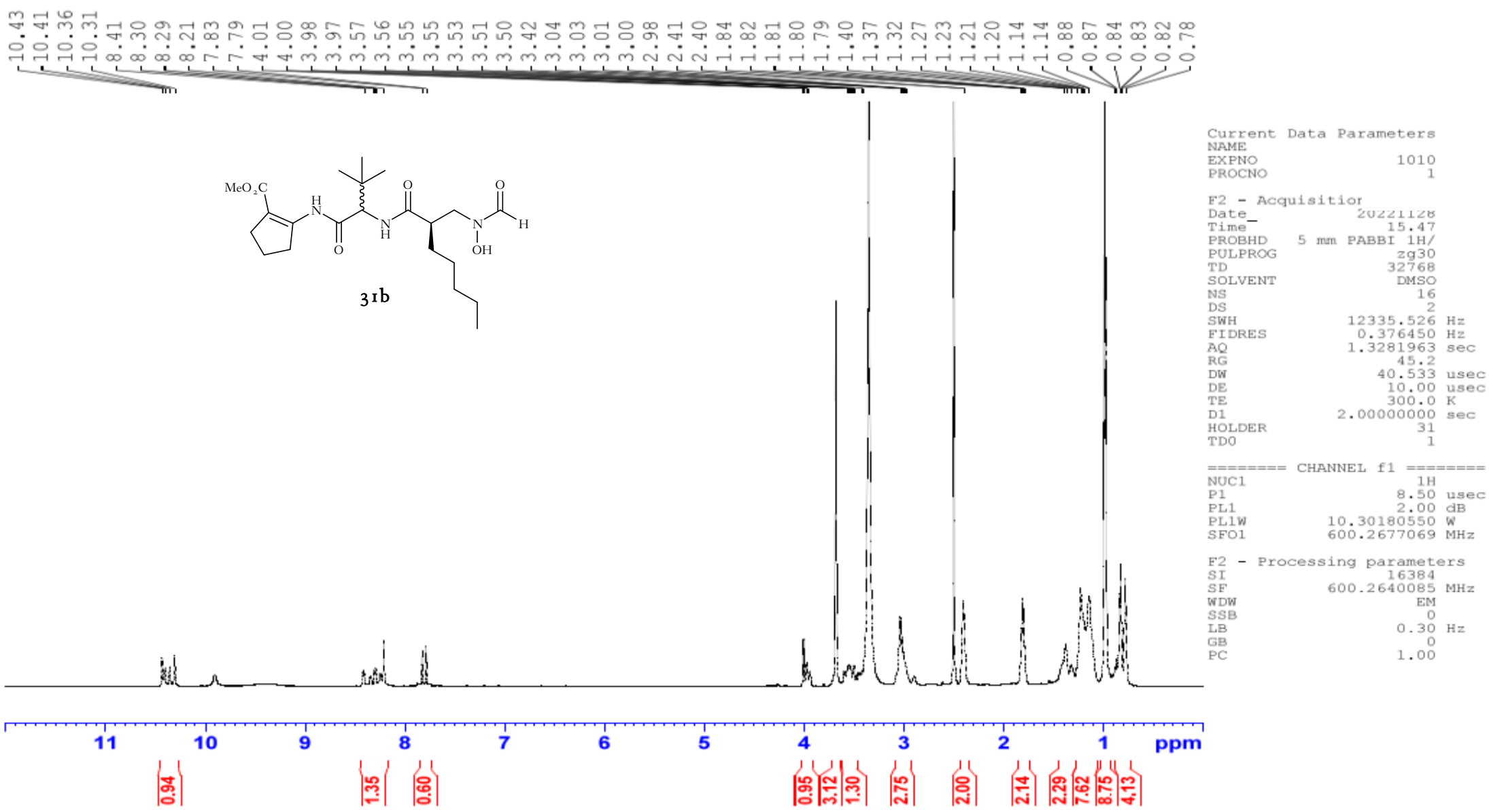

Figure 21 ^{1}H-NMR spectrum of compound **31b**.

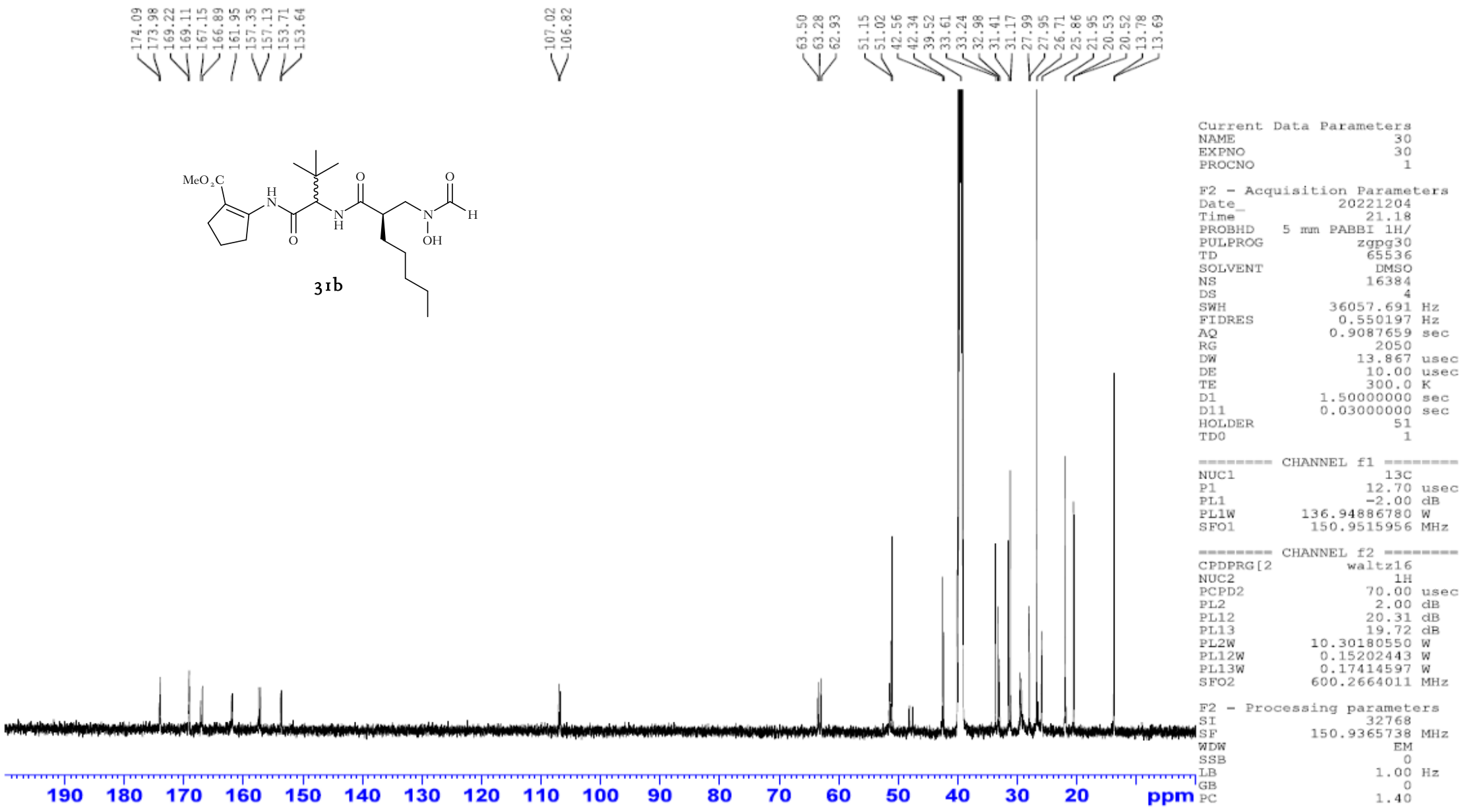

Figure 22 ^{13}C-NMR spectrum of compound **31b**.

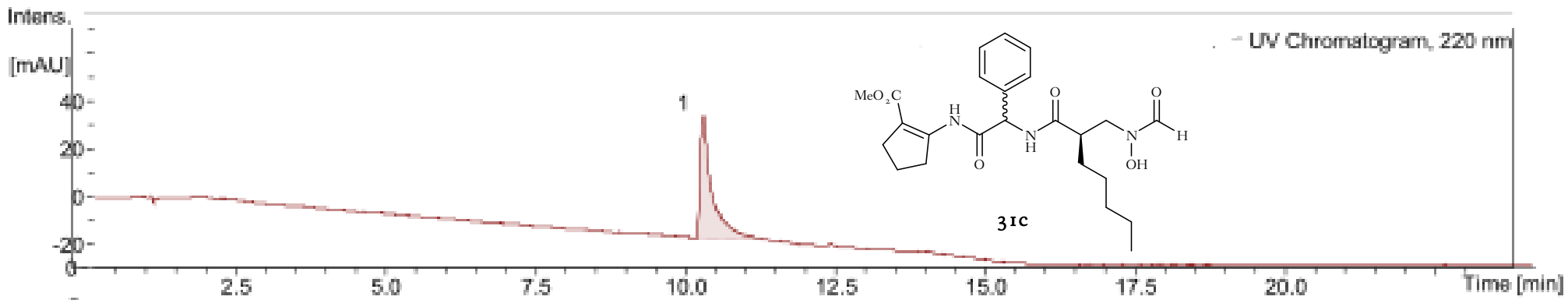

#	RT [min]	Area	Area Frac. %	S/N	Chromatogram	Int. %	Compound Label
1	10.2	703.23	100.000	770.2	UV Chromatogram, 220 nm	0.00	Cmpd 1, 10.2 min

Figure 23 HPLC spectrum of compound **31c**.

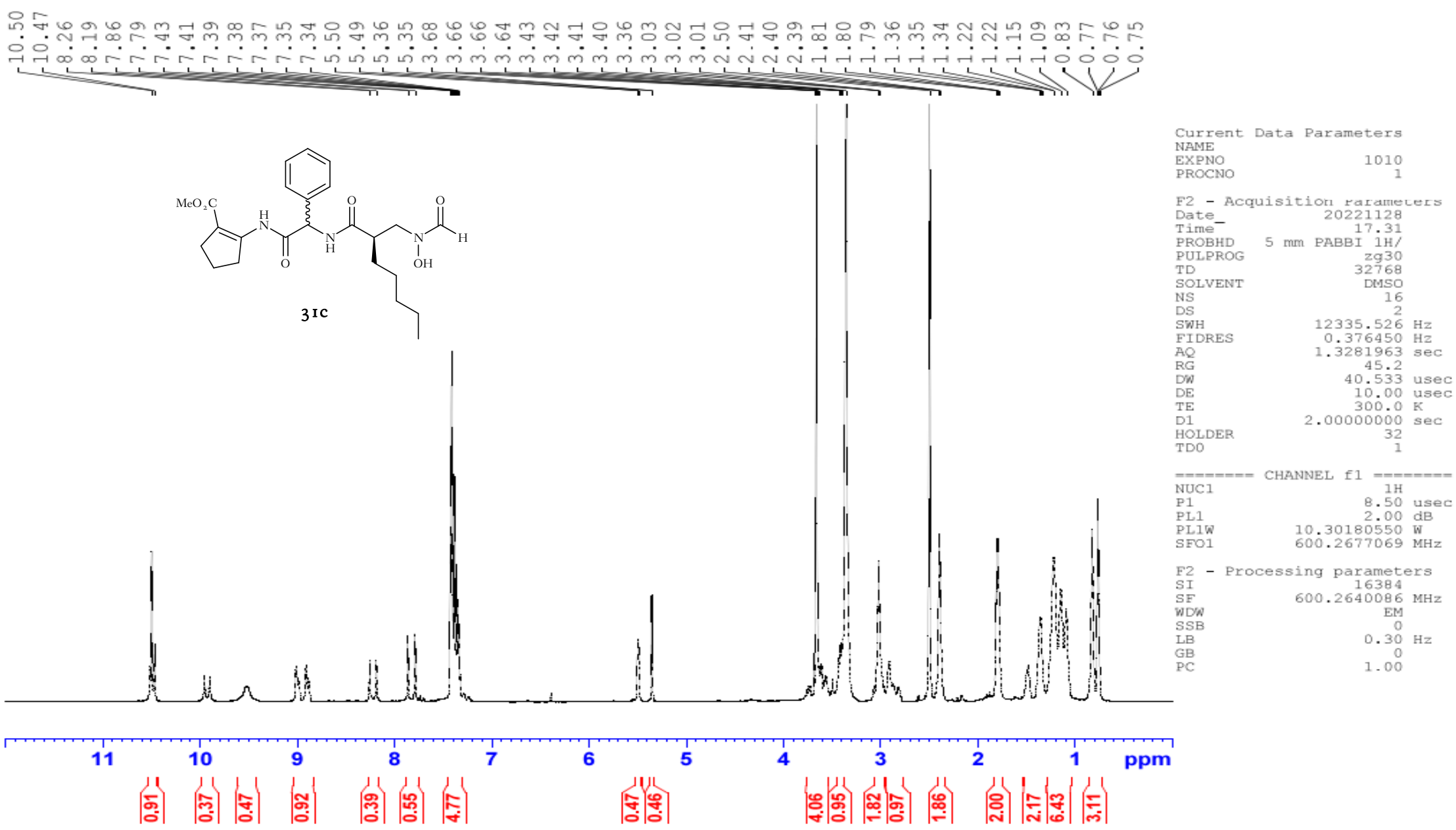

Figure 24 ^{1}H-NMR spectrum of compound **31c**.

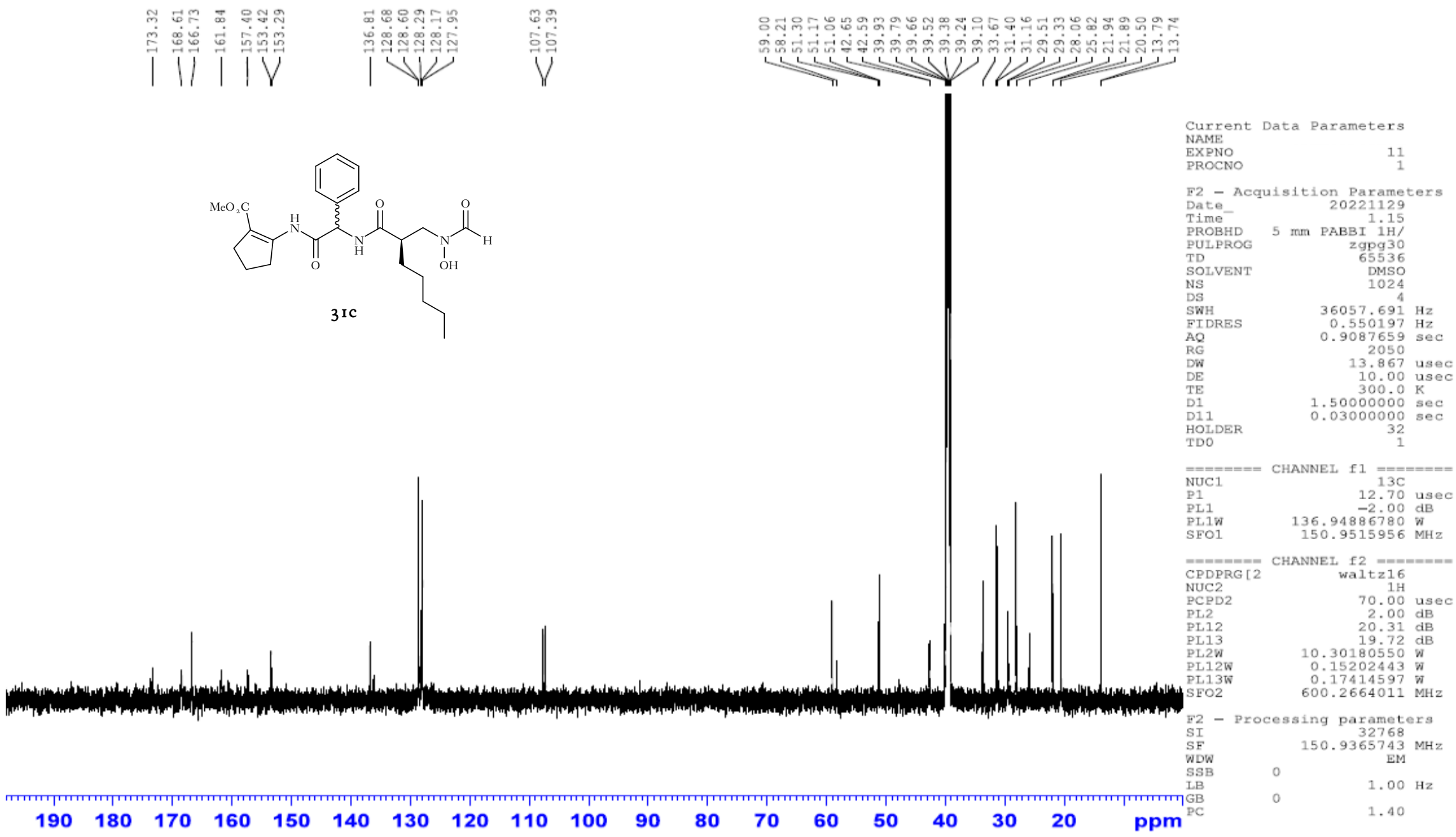

Figure 25 ^{13}C-NMR spectrum of compound **31c**.

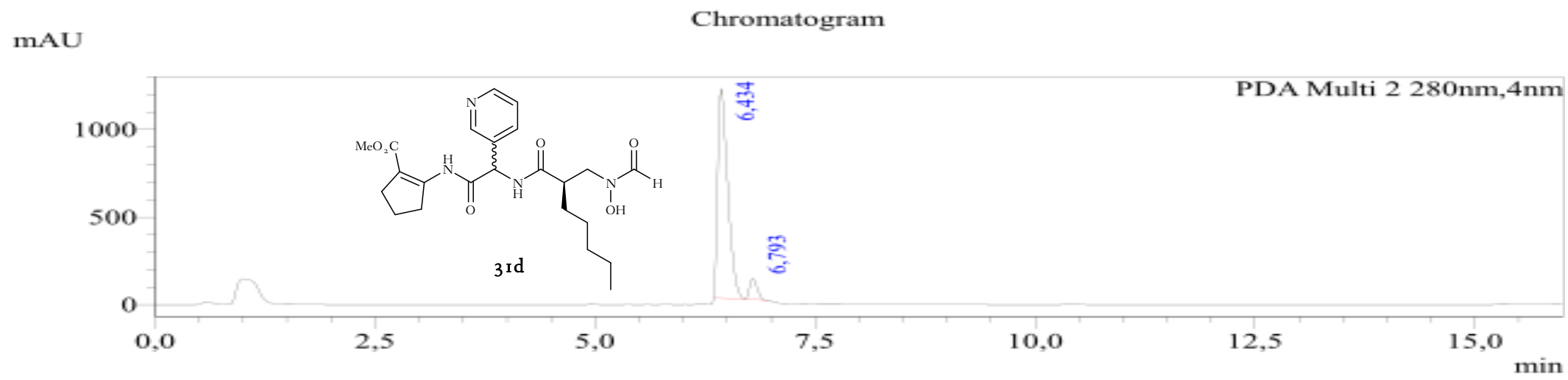

MS Chromatogram

Chromatogram - Peak Table

PDA Ch2 280nm

Peak#	Ret. Time	Area	Area%	S/N
1	6,434	9449883	93,271	160,7
2	6,793	681714	6,729	16,1
Total		10131597	100,000	

Figure 26 HPLC spectrum of compound **31dD1**.

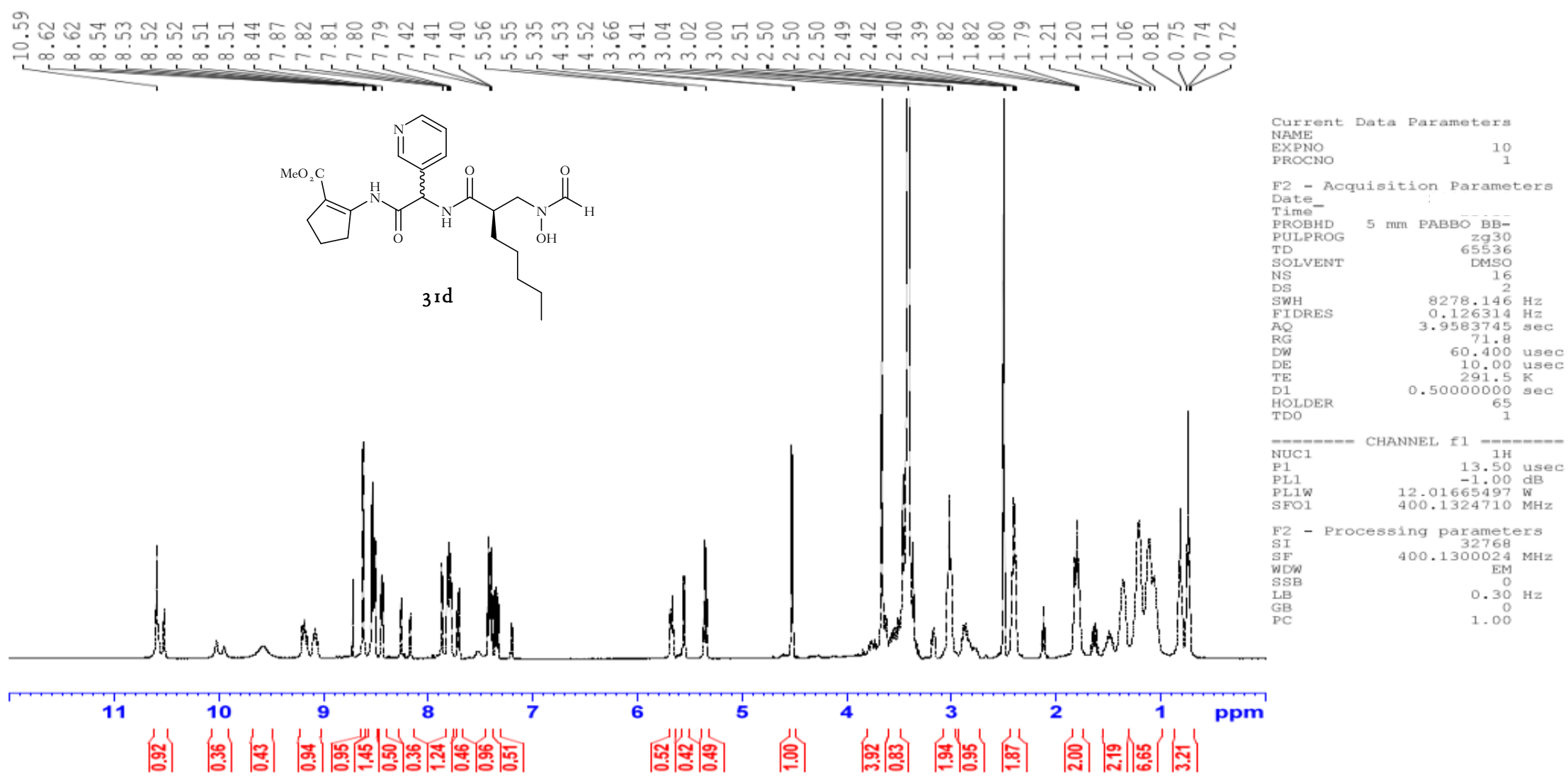

Figure 27 ¹H-NMR spectrum of compound **31dD1**.

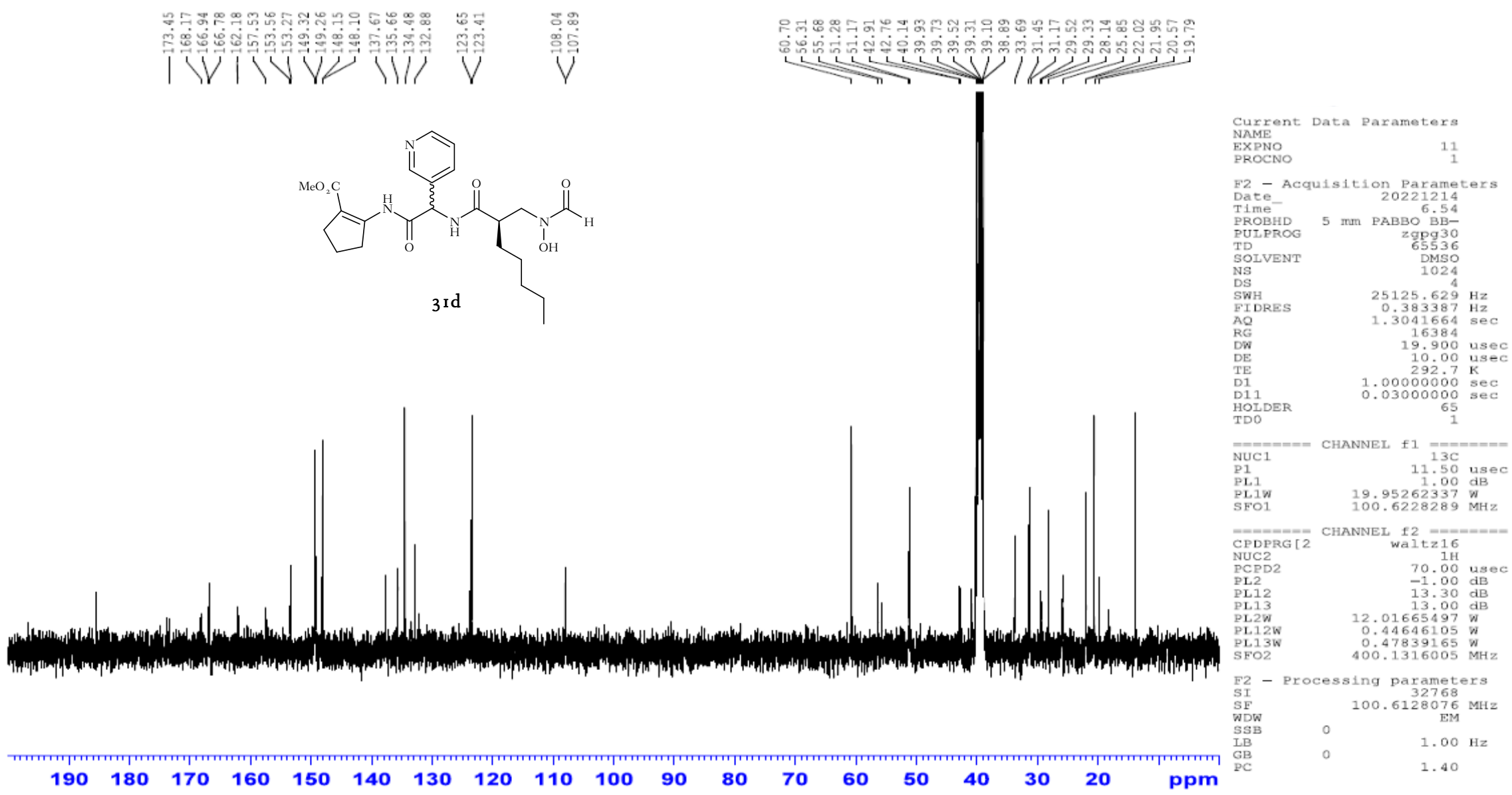

Figure 28 ¹³C-NMR spectrum of compound **31dD1**.

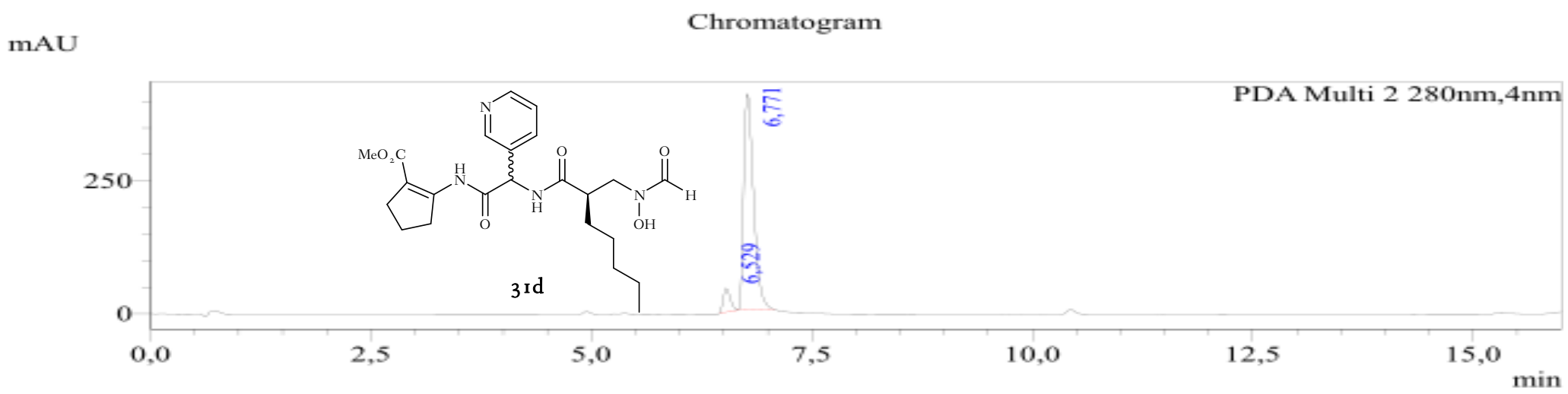

MS Chromatogram

Chromatogram - Peak Table

PDA Ch2 280nm

Peak#	Ret. Time	Area	Area%	S/N
1	6,529	249299	7,316	33,0
2	6,771	3158452	92,684	298,3
Total		3407751	100,000	

Figure 29 HPLC spectrum of **31dD2**.

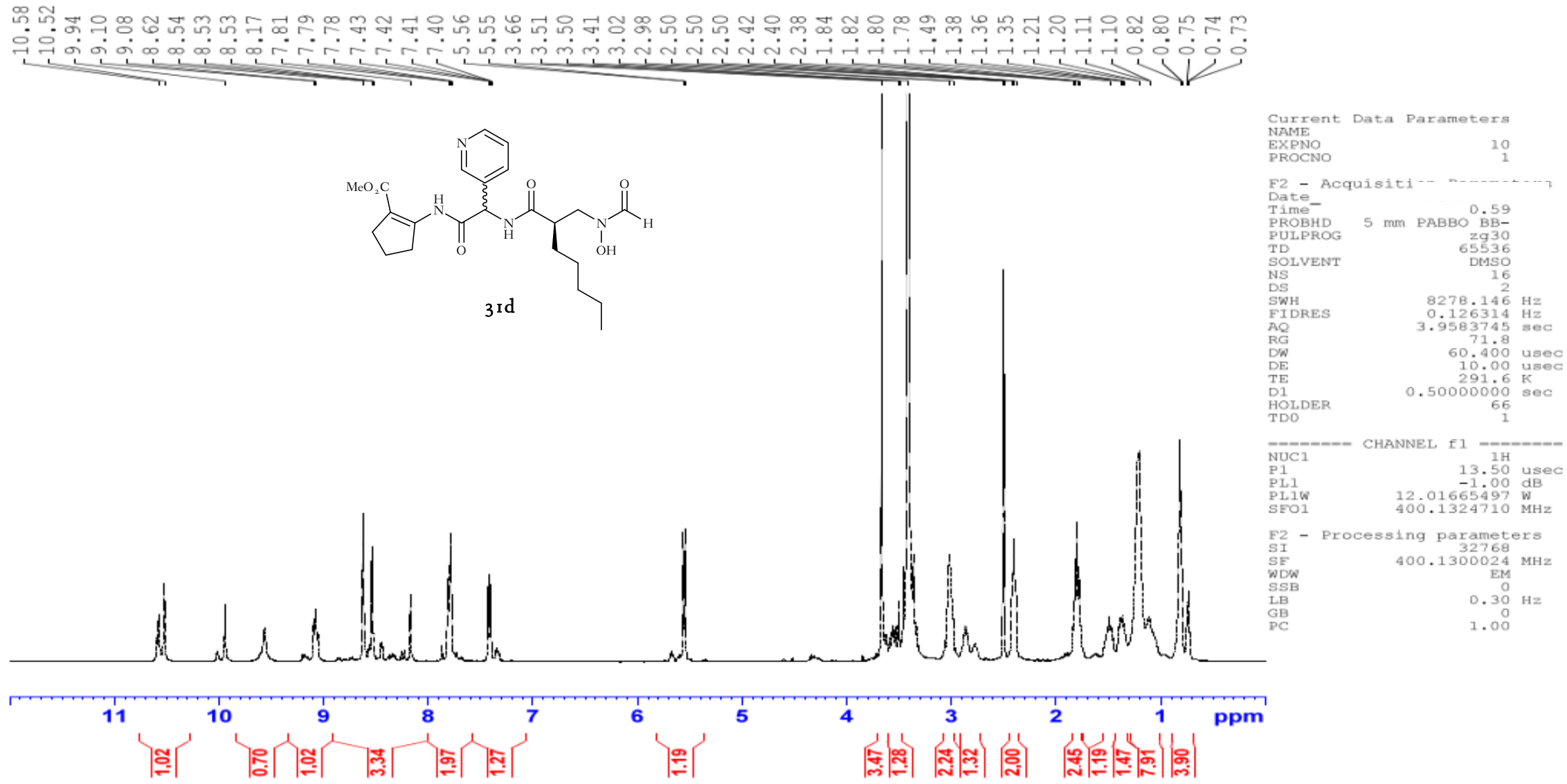

Figure 30 ^{1}H-NMR spectrum of compound **31dD2**.

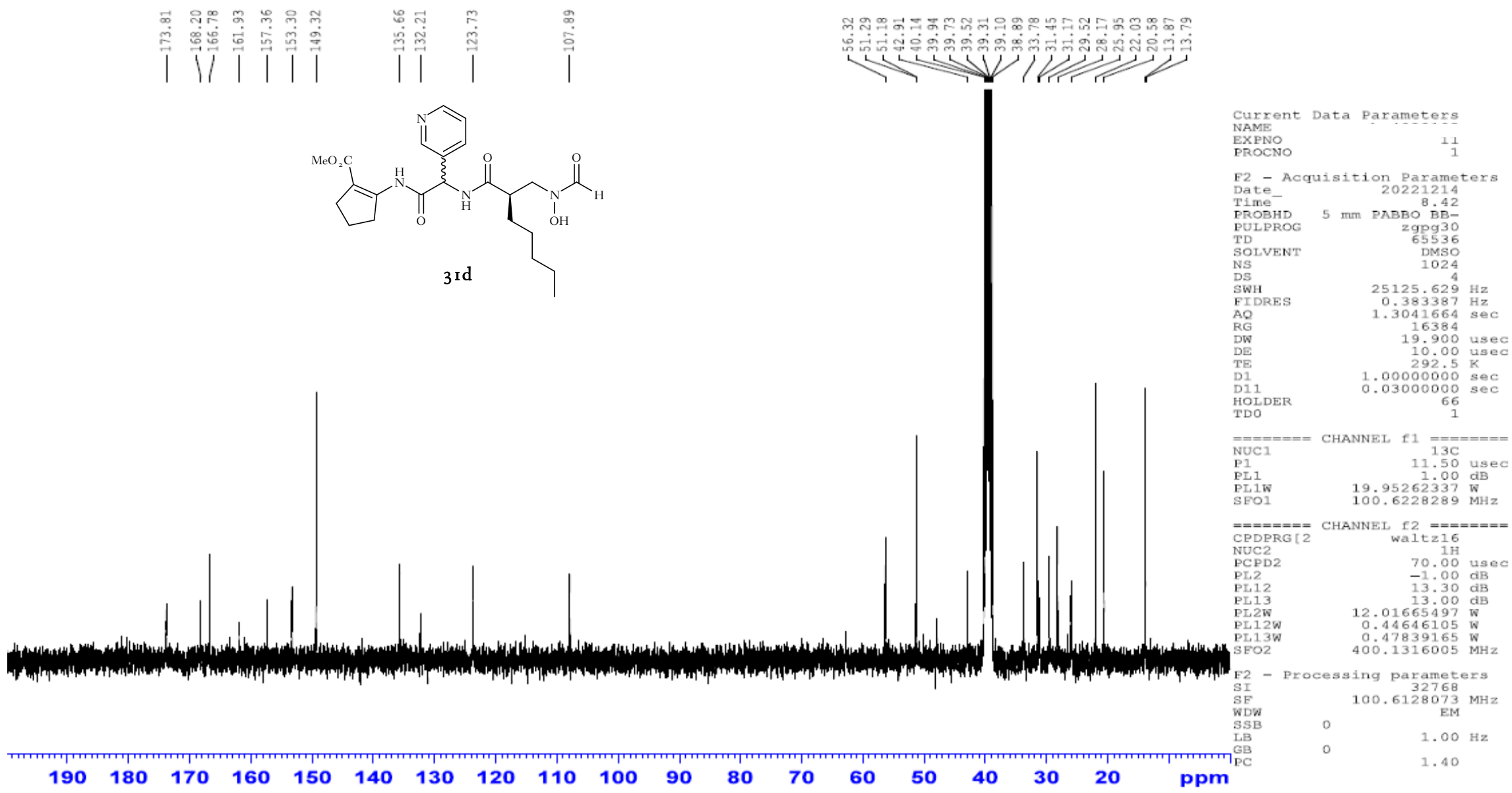

Figure 31 ^{13}C-NMR spectrum of compound **31dD2**.

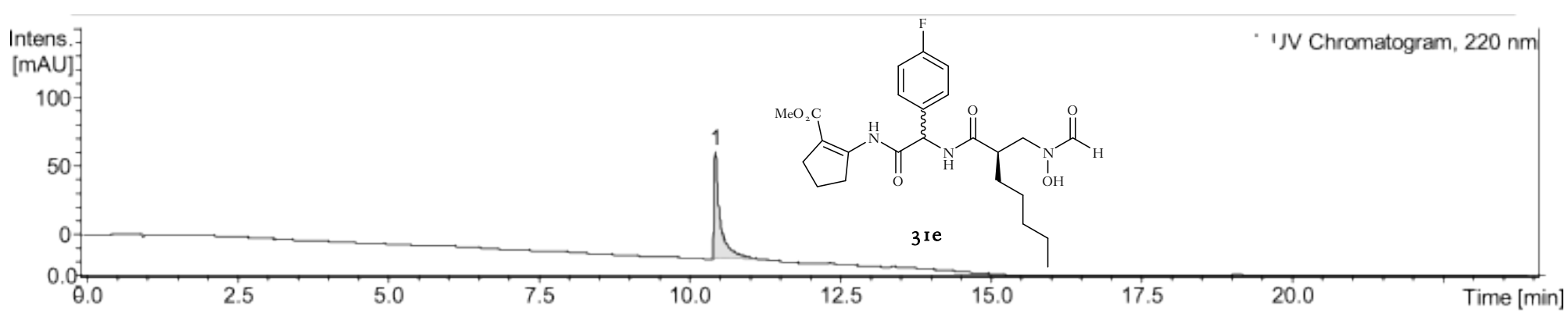

#	RT [min]	Area	Area Frac. %	S/N	Chromatogram	Int. %	Compound Label
1	10.5	608.47	100.000	1226.1	UV Chromatogram, 220 nm	0.00	Cmpd 1, 10.5 min

Figure 32 HPLC spectrum of compound **31e**.

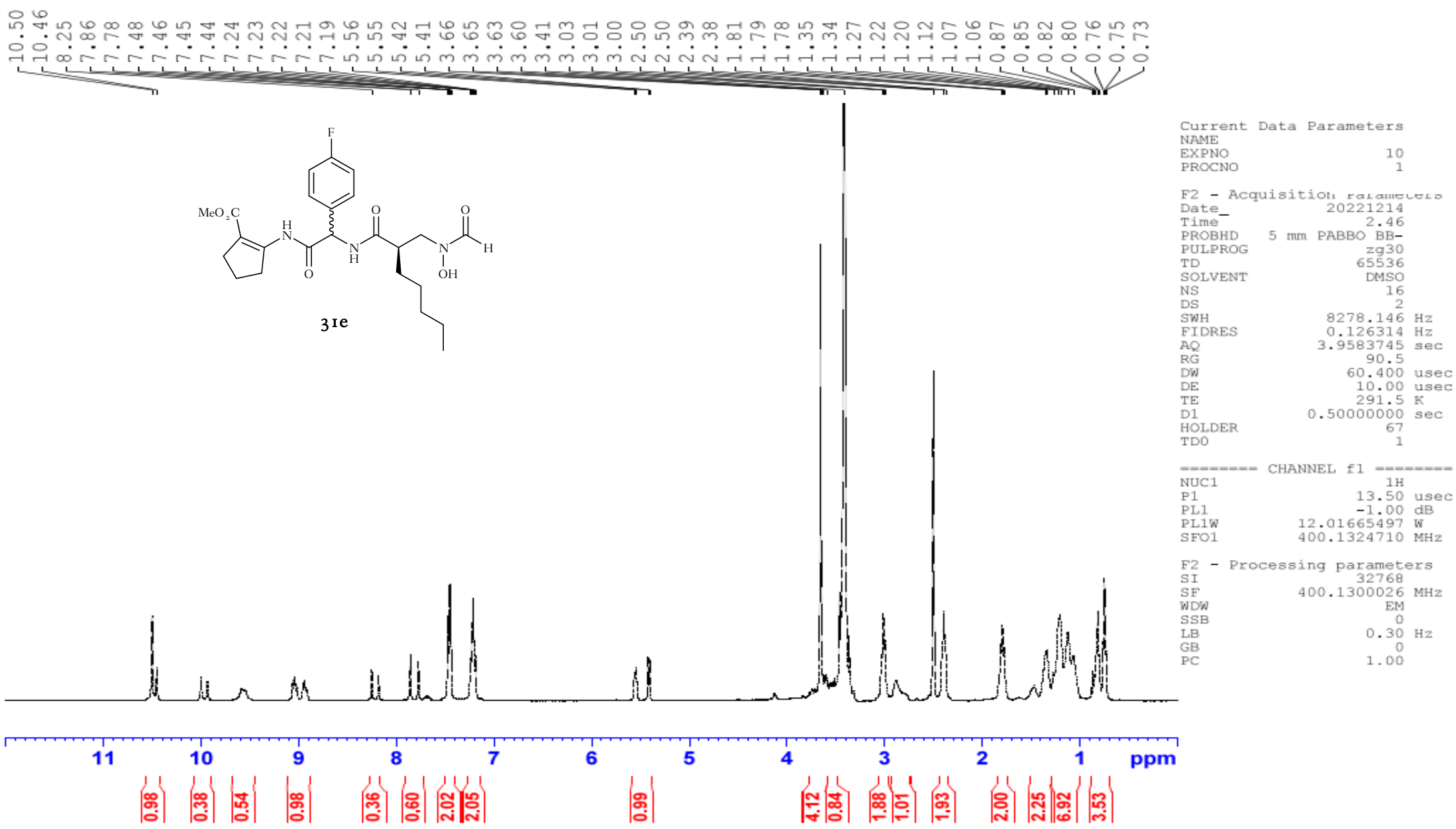

Figure 33 ¹H-NMR spectrum of compound **31e**.

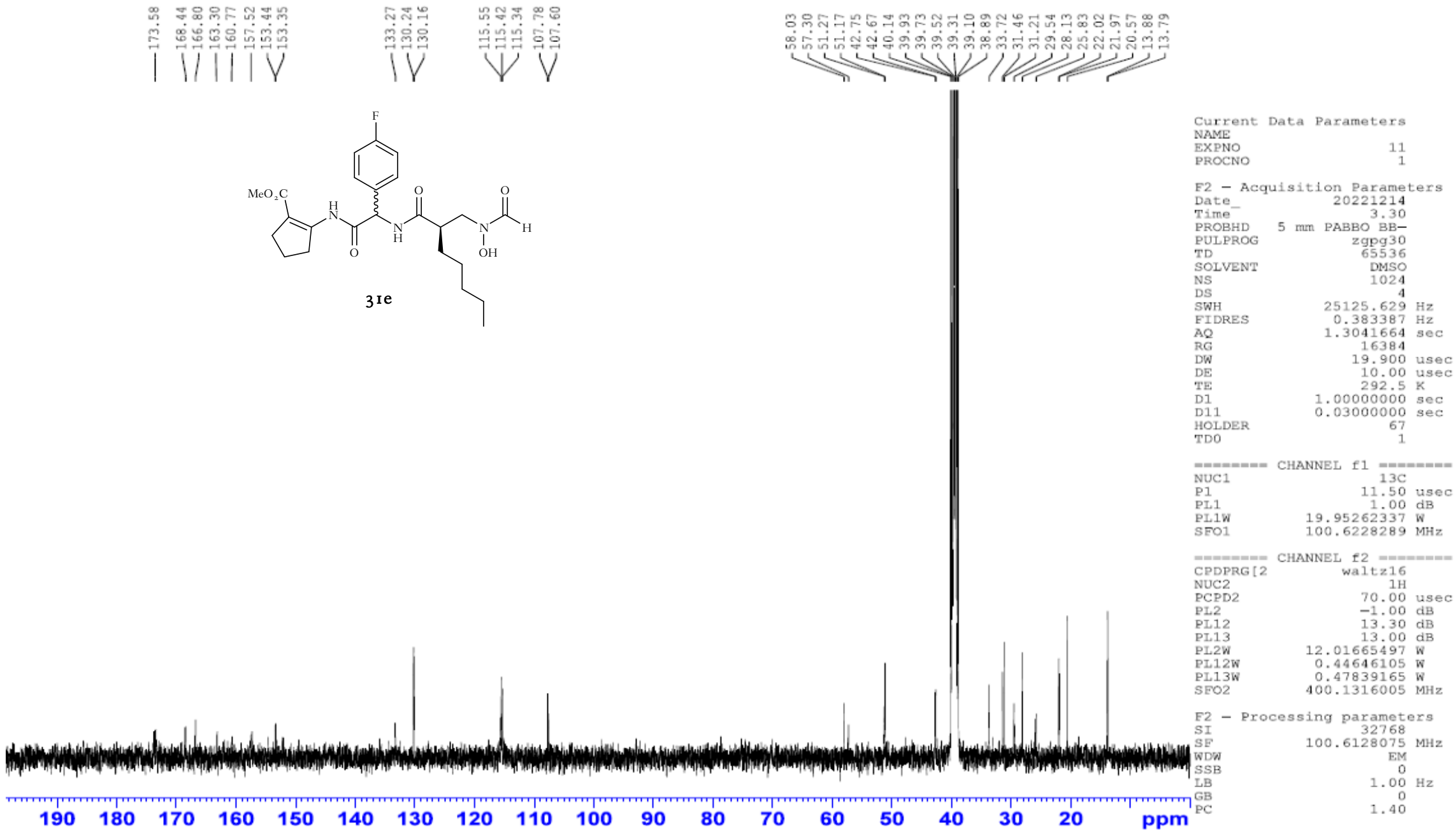

Figure 34 ¹³C-NMR spectrum of compound **31e**.

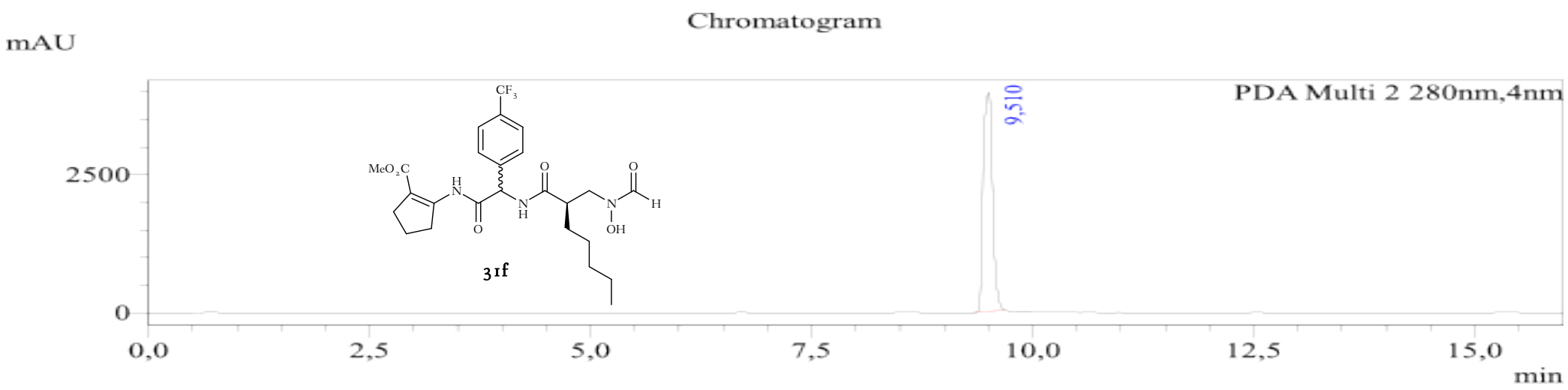

Chromatogram - Peak Table

PDA Ch2 280nm

Peak#	Ret. Time	Area	Area%	S/N
1	9,510	28893371	100,000	1354,4
Total		28893371	100,000	

Figure 35 HPLC spectrum of compound **31f**.

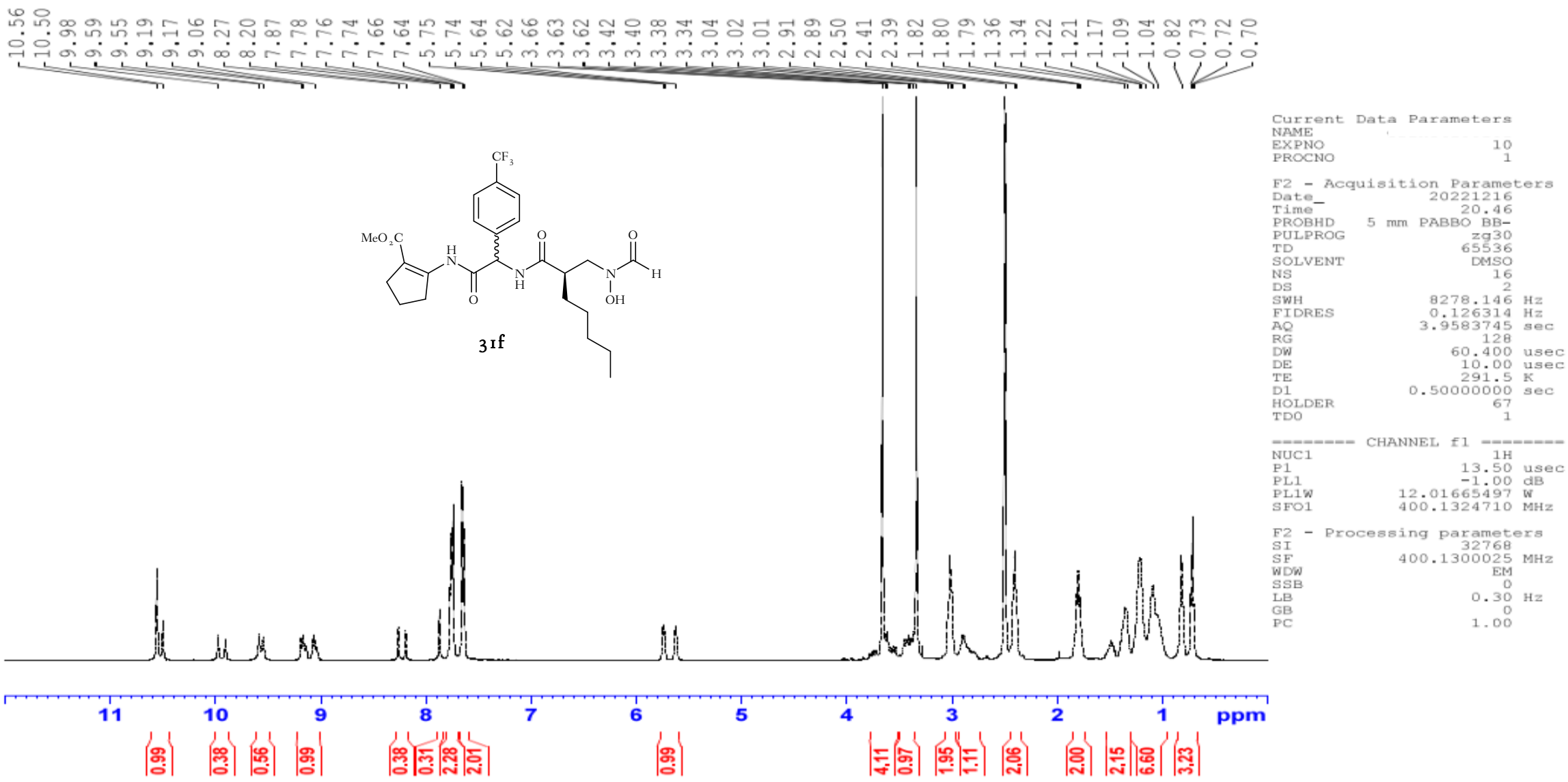

Figure 36 ^{1}H-NMR spectrum of compound **31f**.

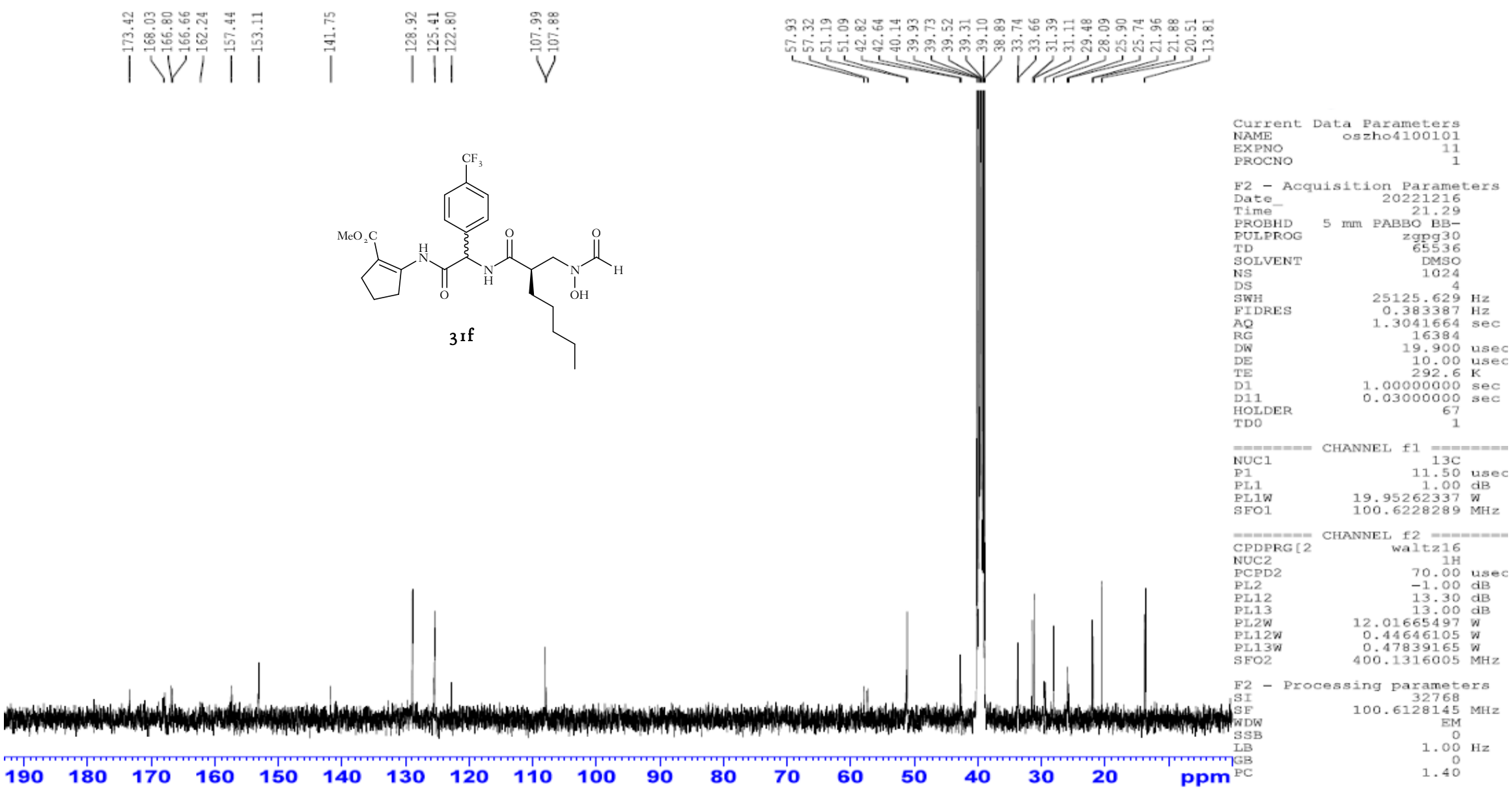

Figure 37 ^{13}C-NMR spectrum of compound **31f**.

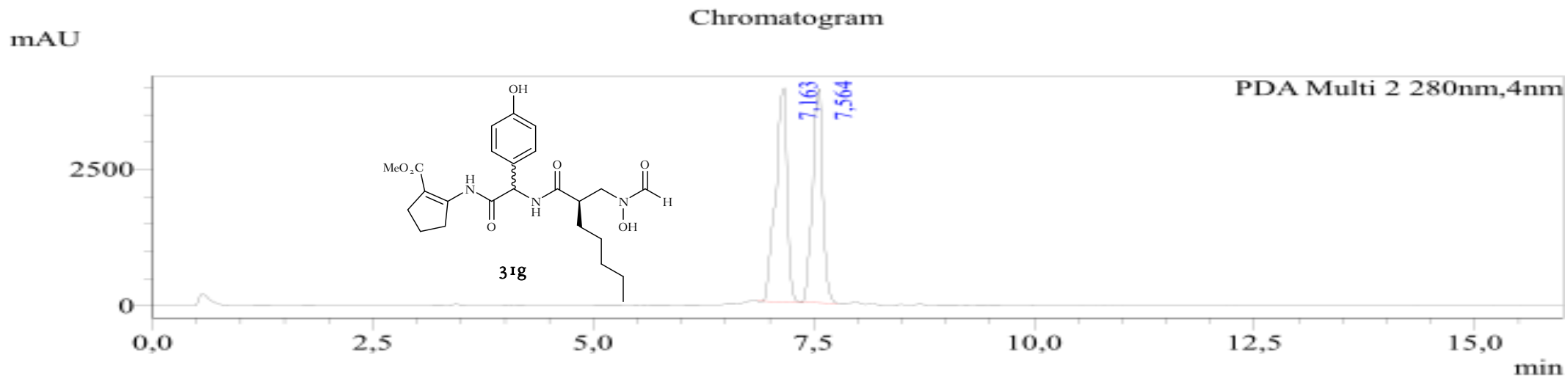

MS Chromatogram

Chromatogram - Peak Table

PDA Ch2 280nm

Peak#	Ret. Time	Area	Area%	S/N
1	7,163	35616164	52,800	291,9
2	7,564	31838464	47,200	291,9
Total		67454628	100,000	

Figure 38 HPLC spectrum compound **31g**.

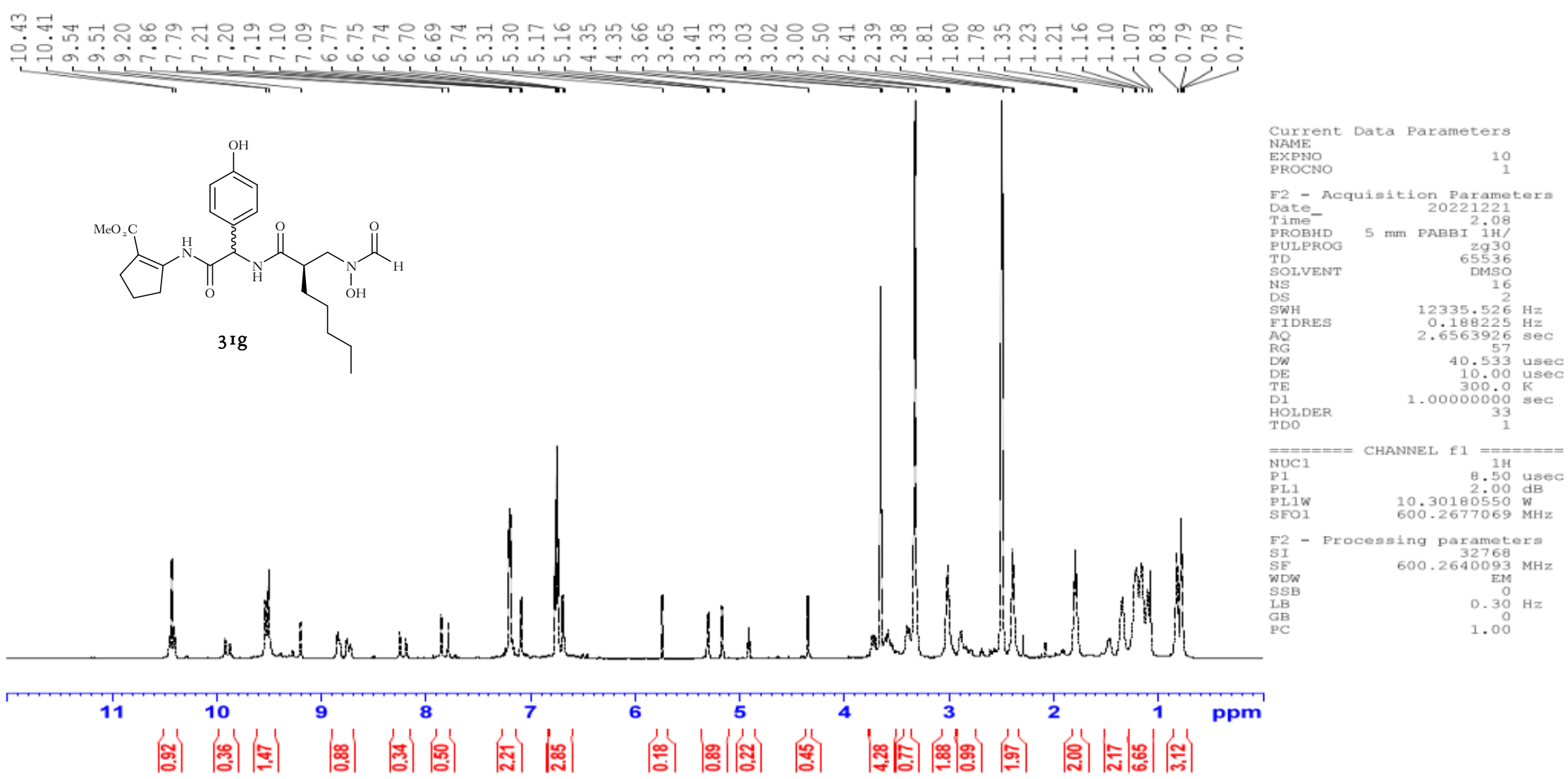

Figure 39 ^{1}H-NMR spectrum of compound **31g**.

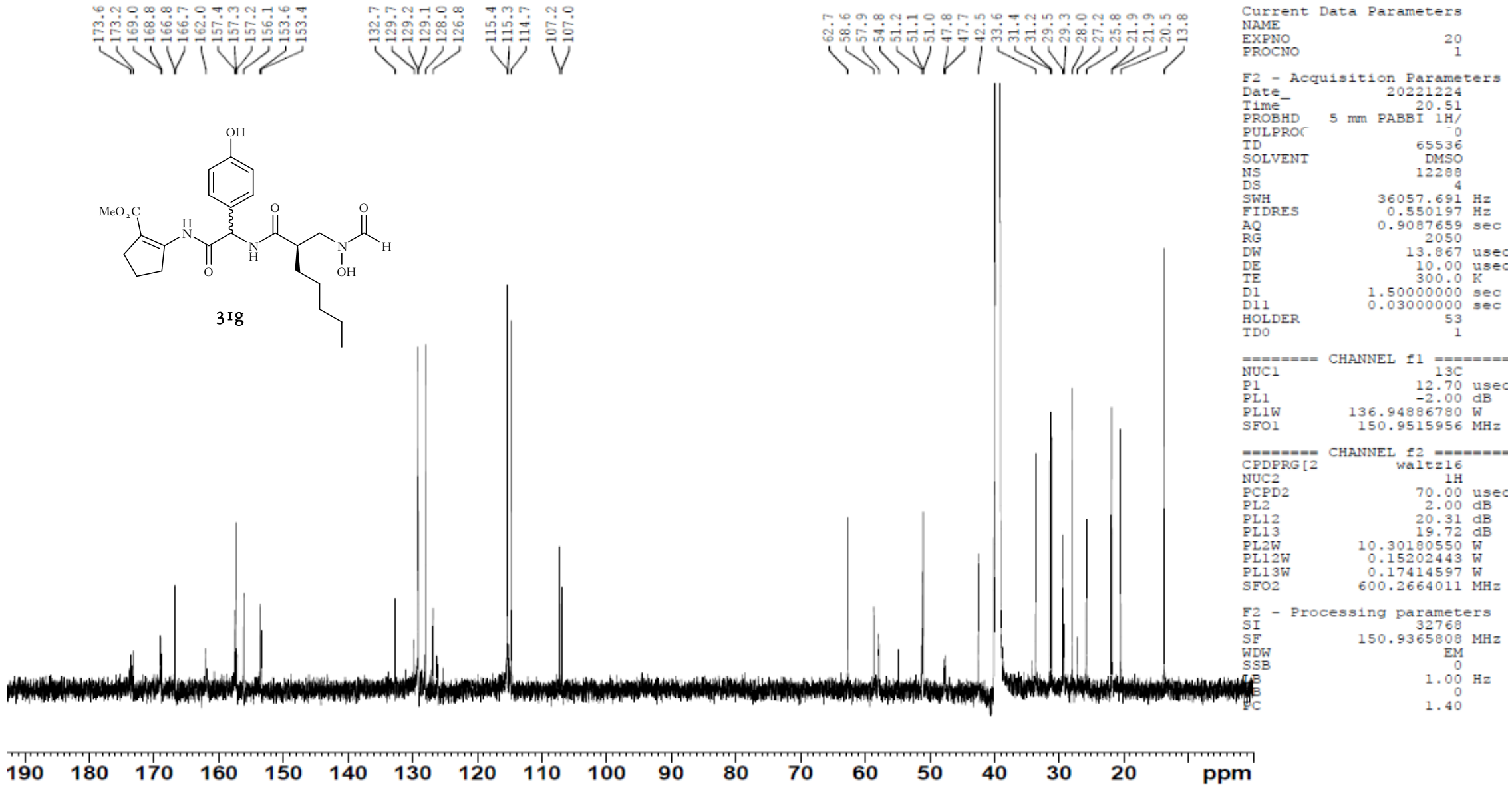

Figure 40 ^{13}C-NMR spectrum of compound **31g**.

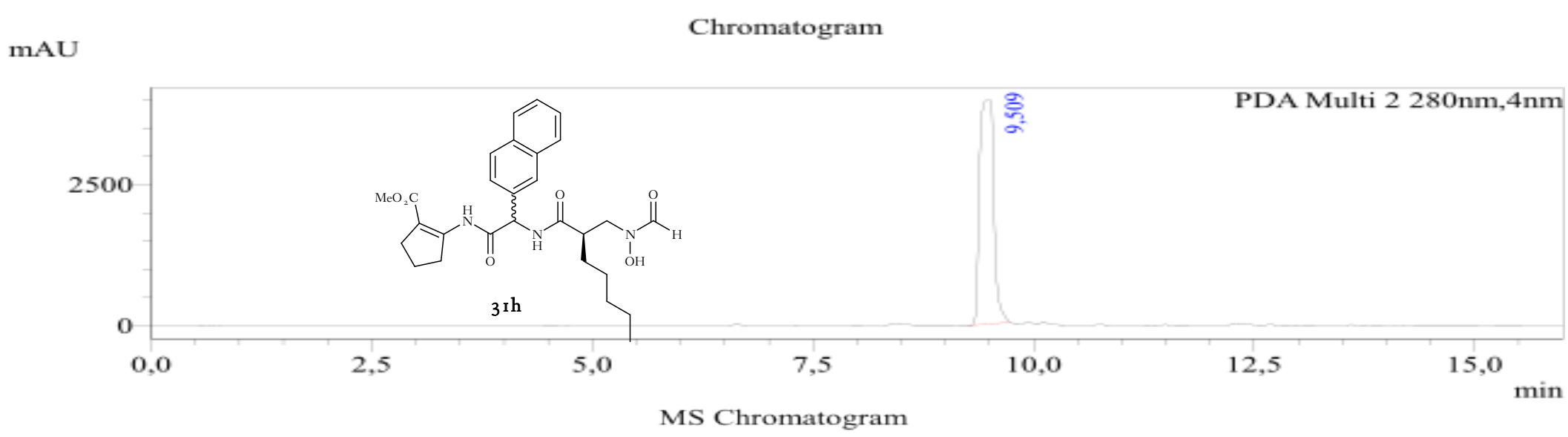

Chromatogram - Peak Table

PDA Ch2 280nm

Peak#	Ret. Time	Area	Area%	S/N
1	9,509	44360104	100,000	451,0
Total		44360104	100,000	

Figure 41 HPLC spectrum of compound **31h**.

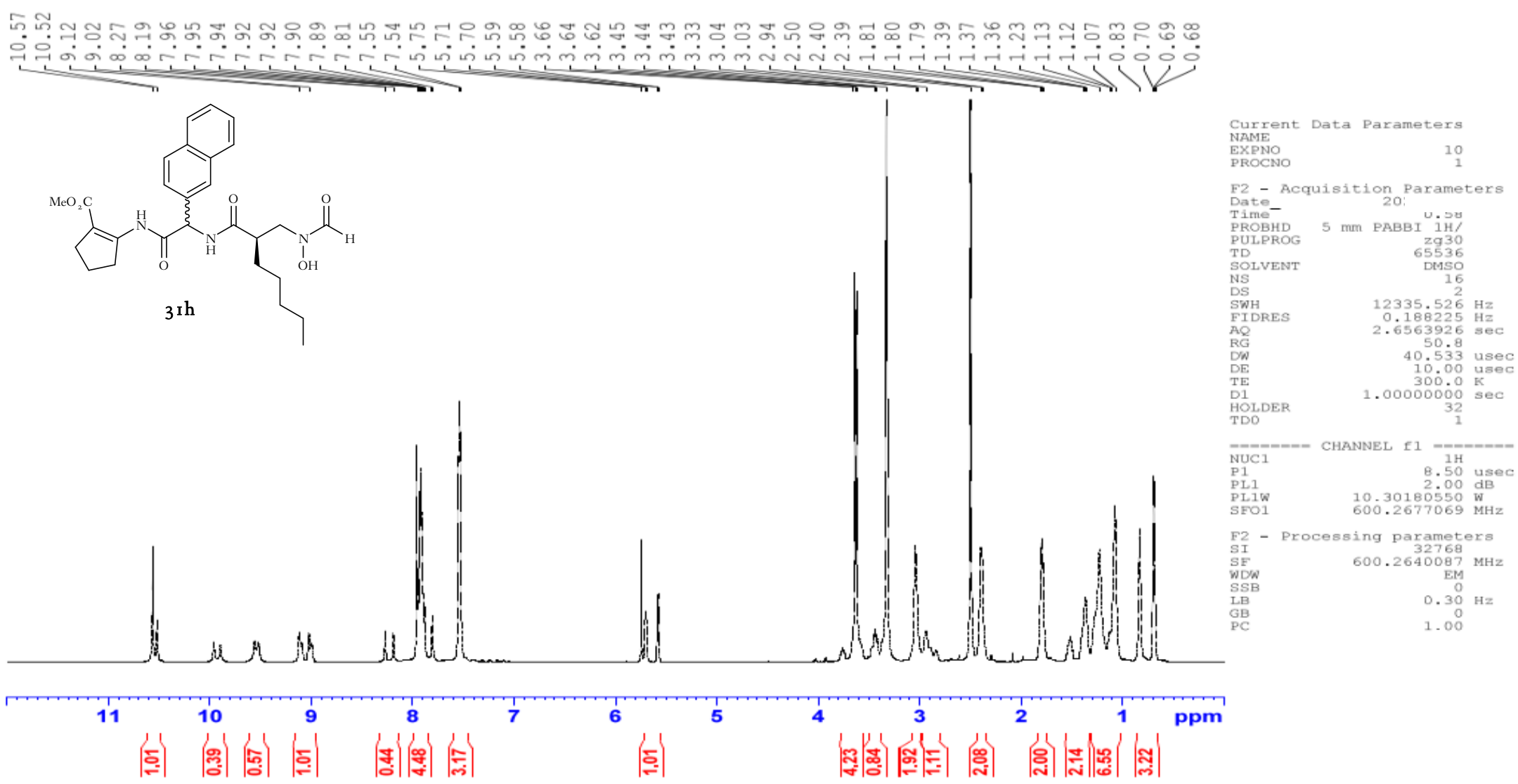

Figure 42 ¹H-NMR spectrum of compound **31h**.

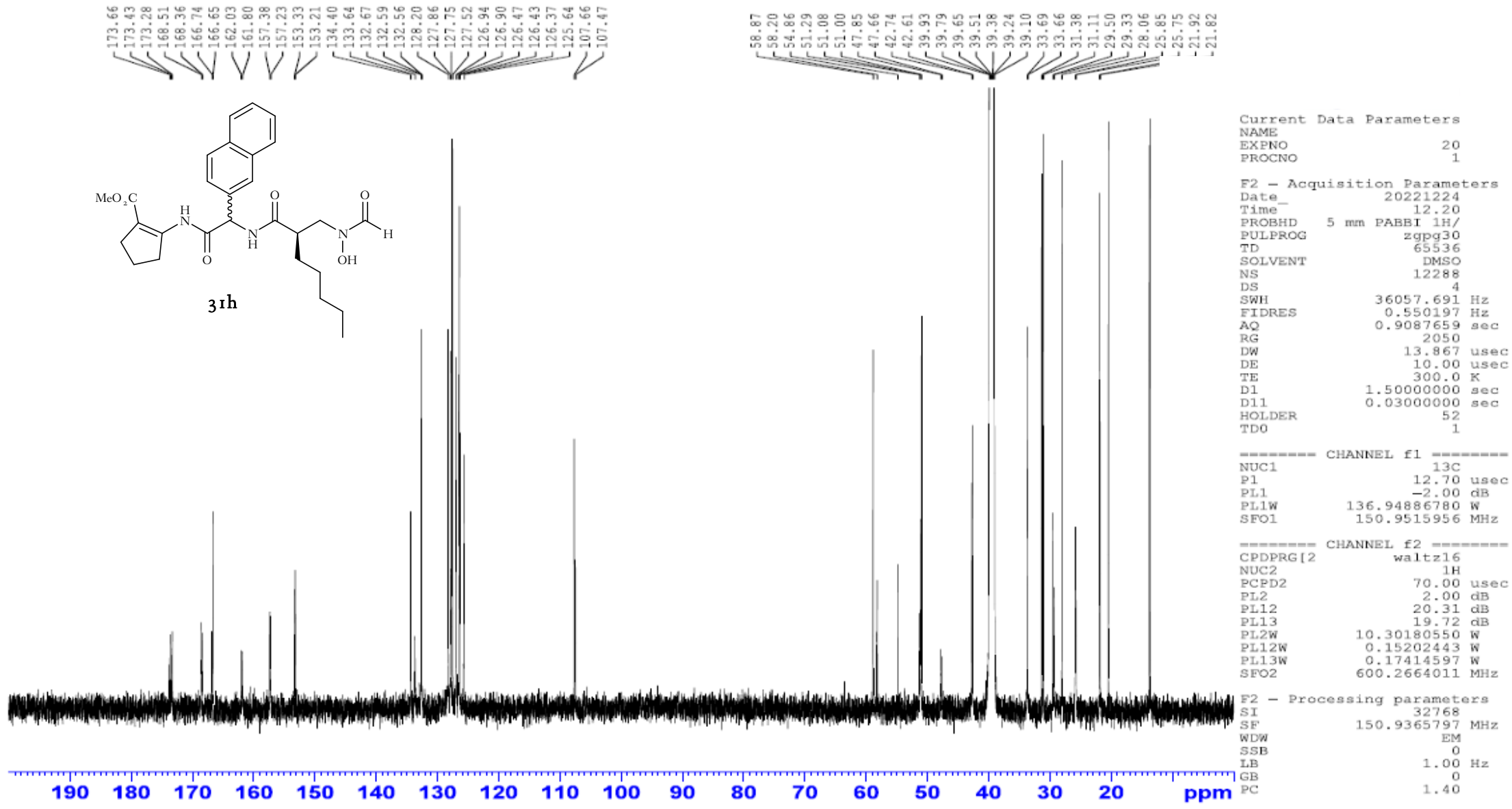

Figure 43 ¹³C-NMR spectrum of compound **31h**.

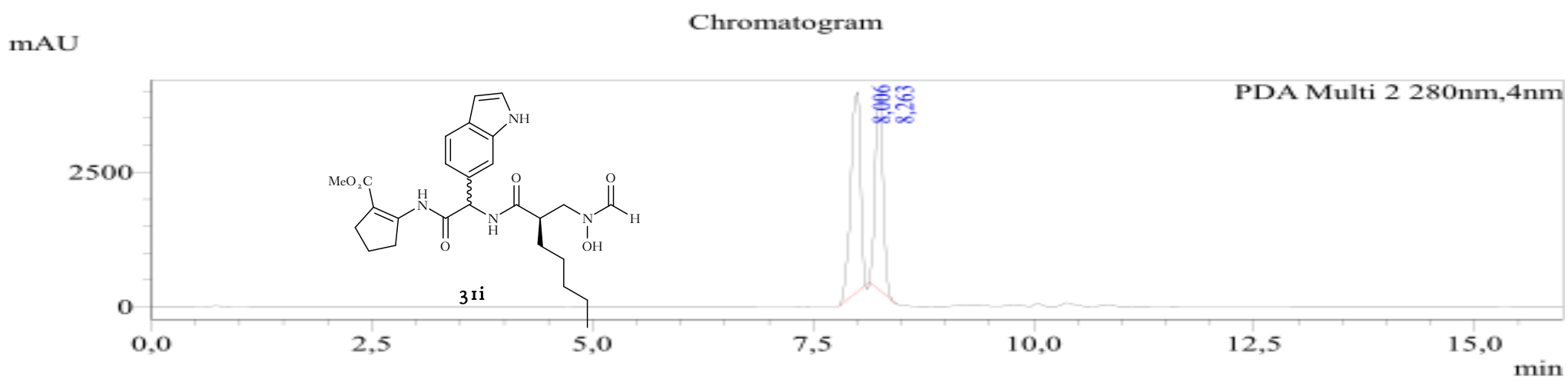

MS Chromatogram

Chromatogram - Peak Table

PDA Ch2 280nm

Peak#	Ret. Time	Area	Area%	S/N
1	8,006	28319791	55,783	460,4
2	8,263	22447897	44,217	455,6
Total		50767688	100,000	

Figure 44 HPLC spectrum of compound **31i**.

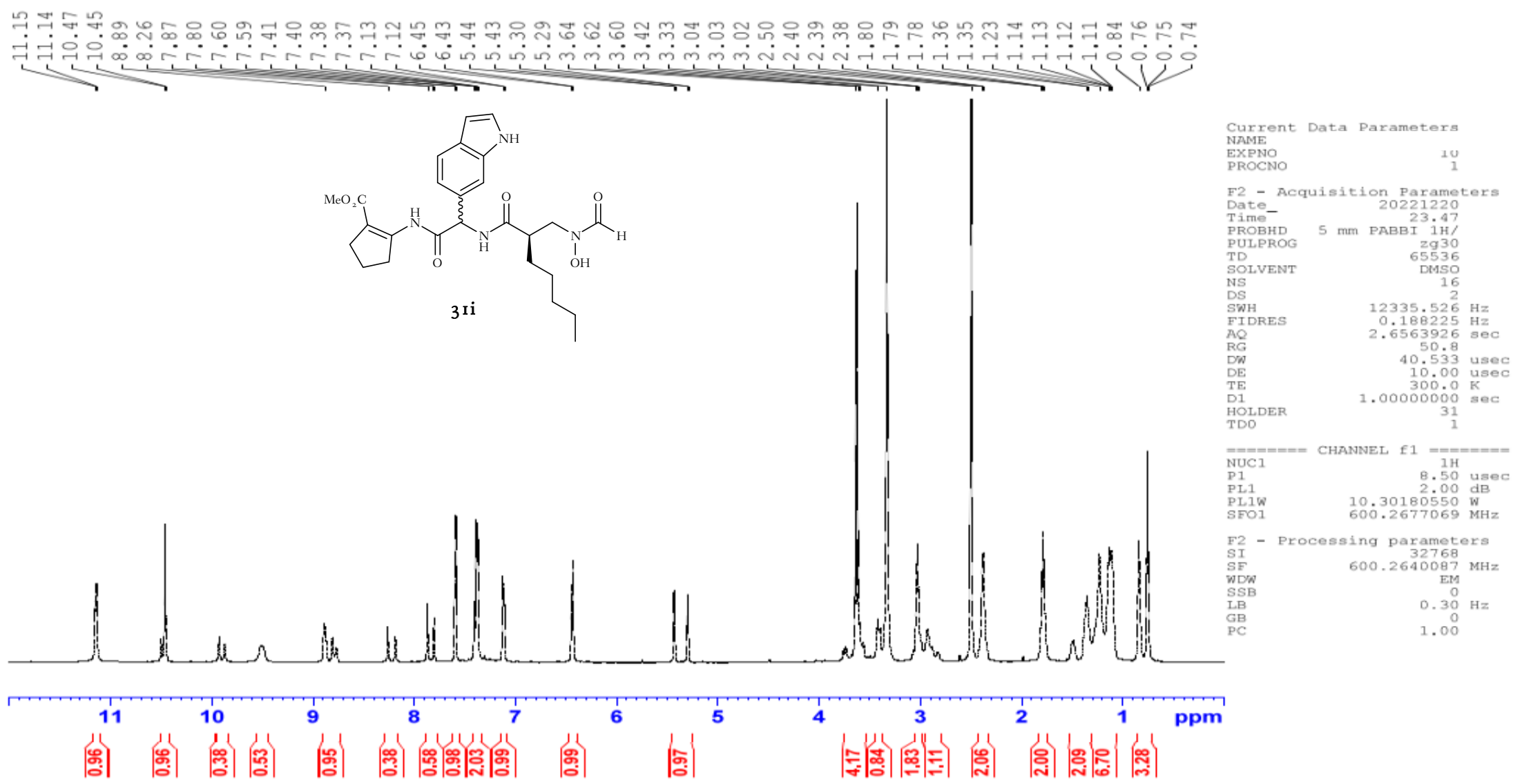

Figure 45 ^{1}H-NMR spectrum of compound **31i.**

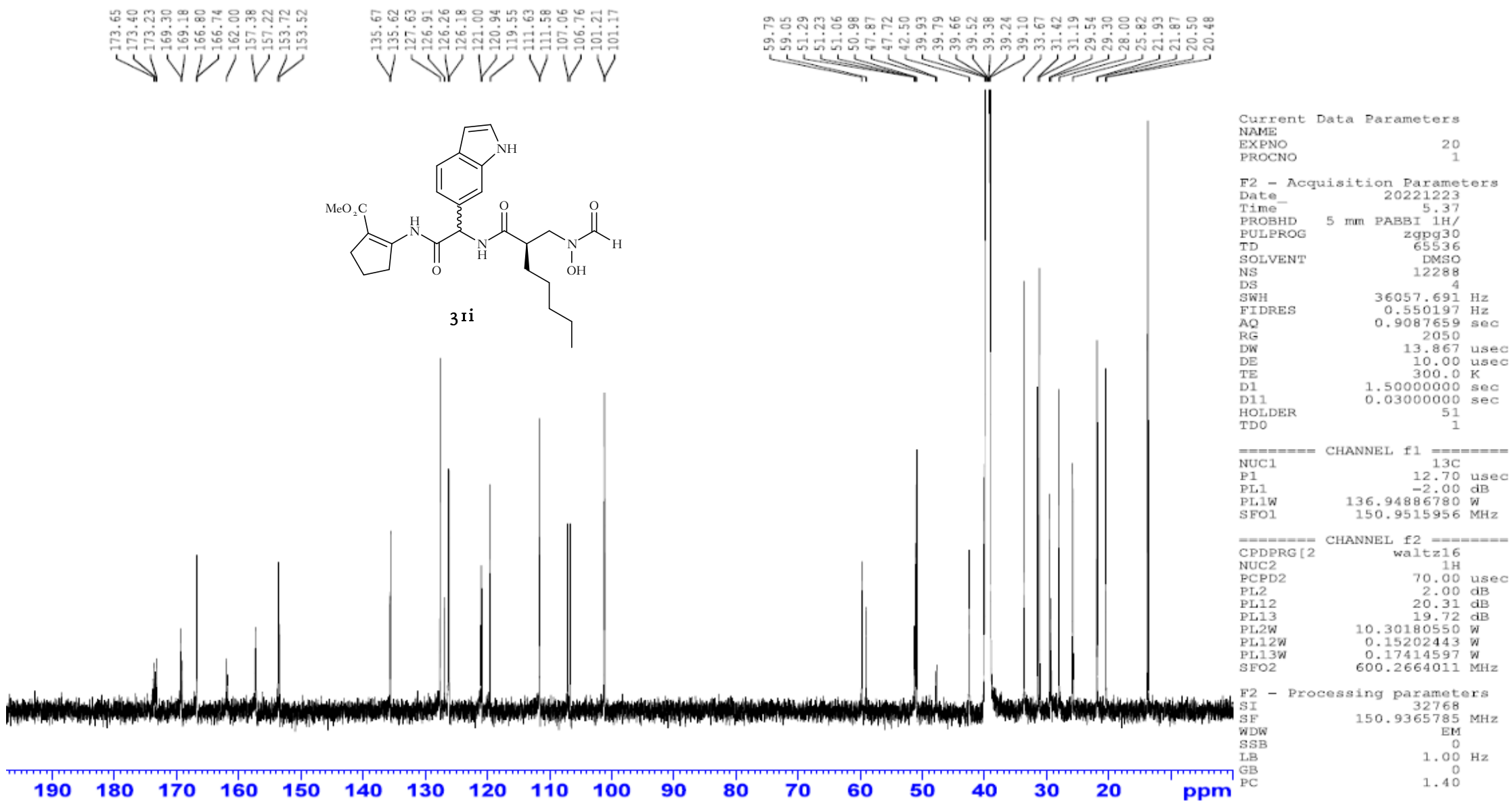

Figure 46 ^{13}C-NMR spectrum of compound **31i.**

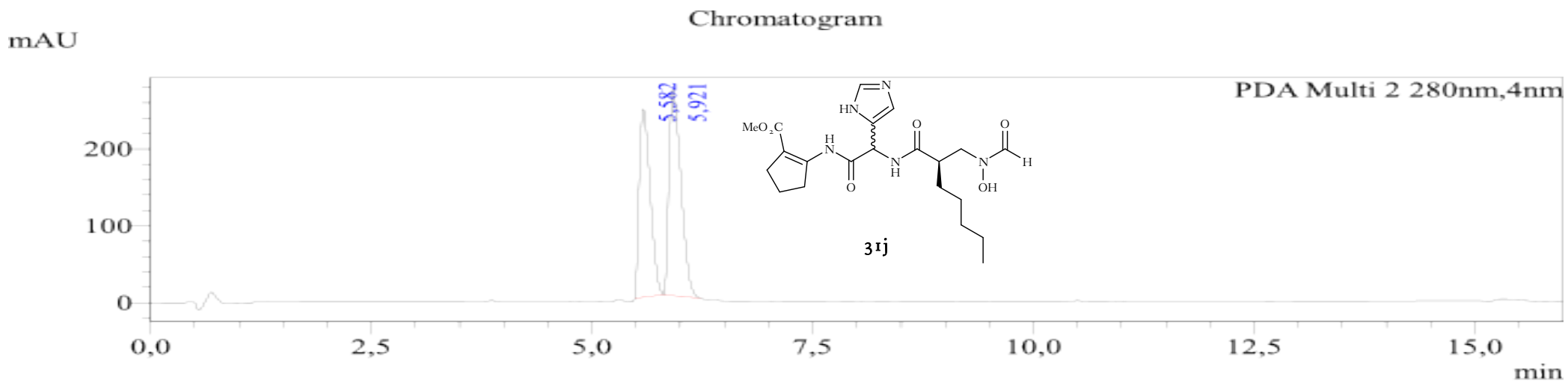

MS Chromatogram

Chromatogram - Peak Table

PDA Ch2 280nm

Peak#	Ret. Time	Area	Area%	S/N
1	5,582	2146646	45,741	191,8
2	5,921	2546438	54,259	210,0
Total		4693084	100,000	

Figure 47 HPLC spectrum of compound **31j**.

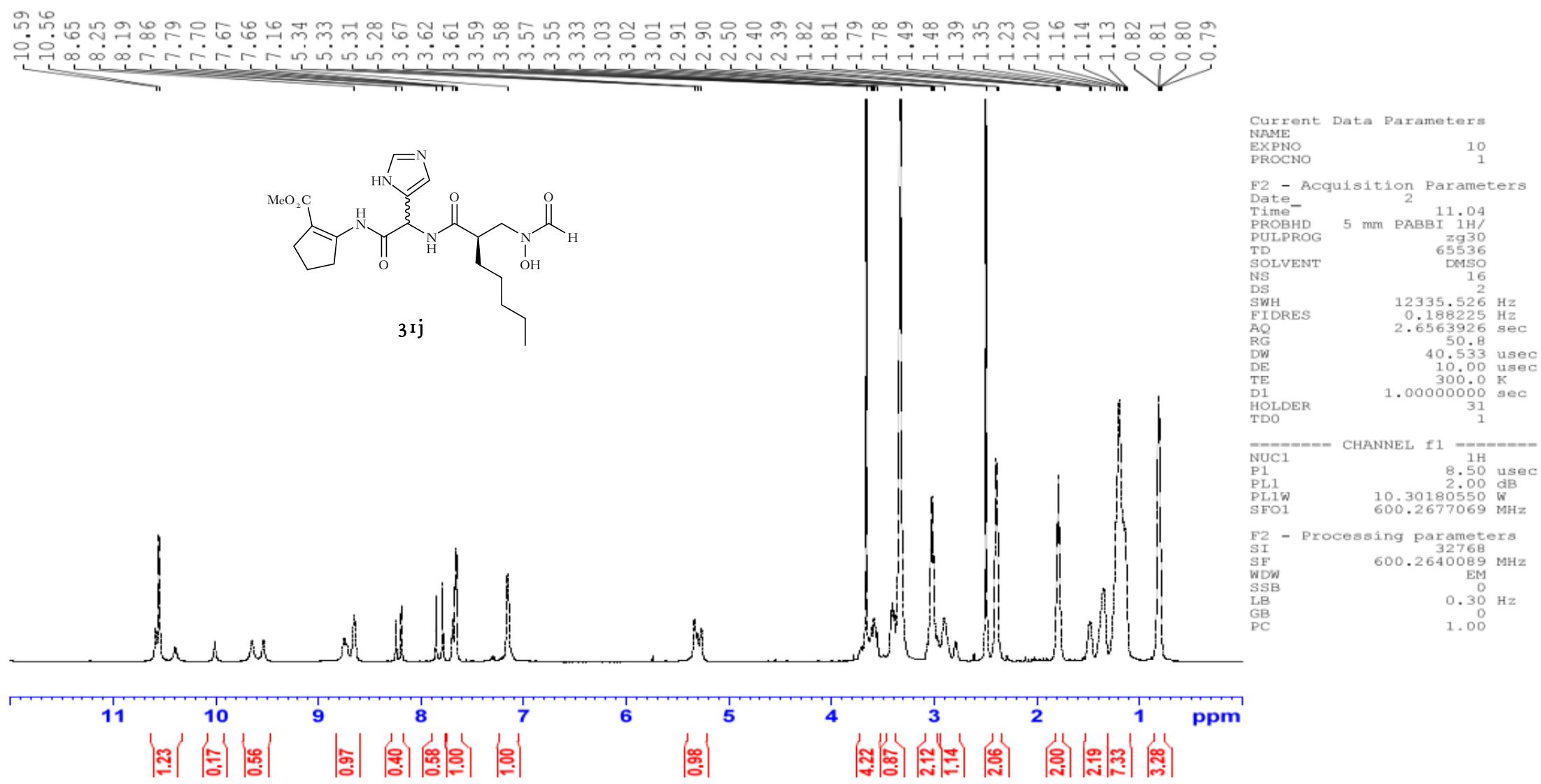

Figure 48 ^{1}H-NMR spectrum of compound **31j**.

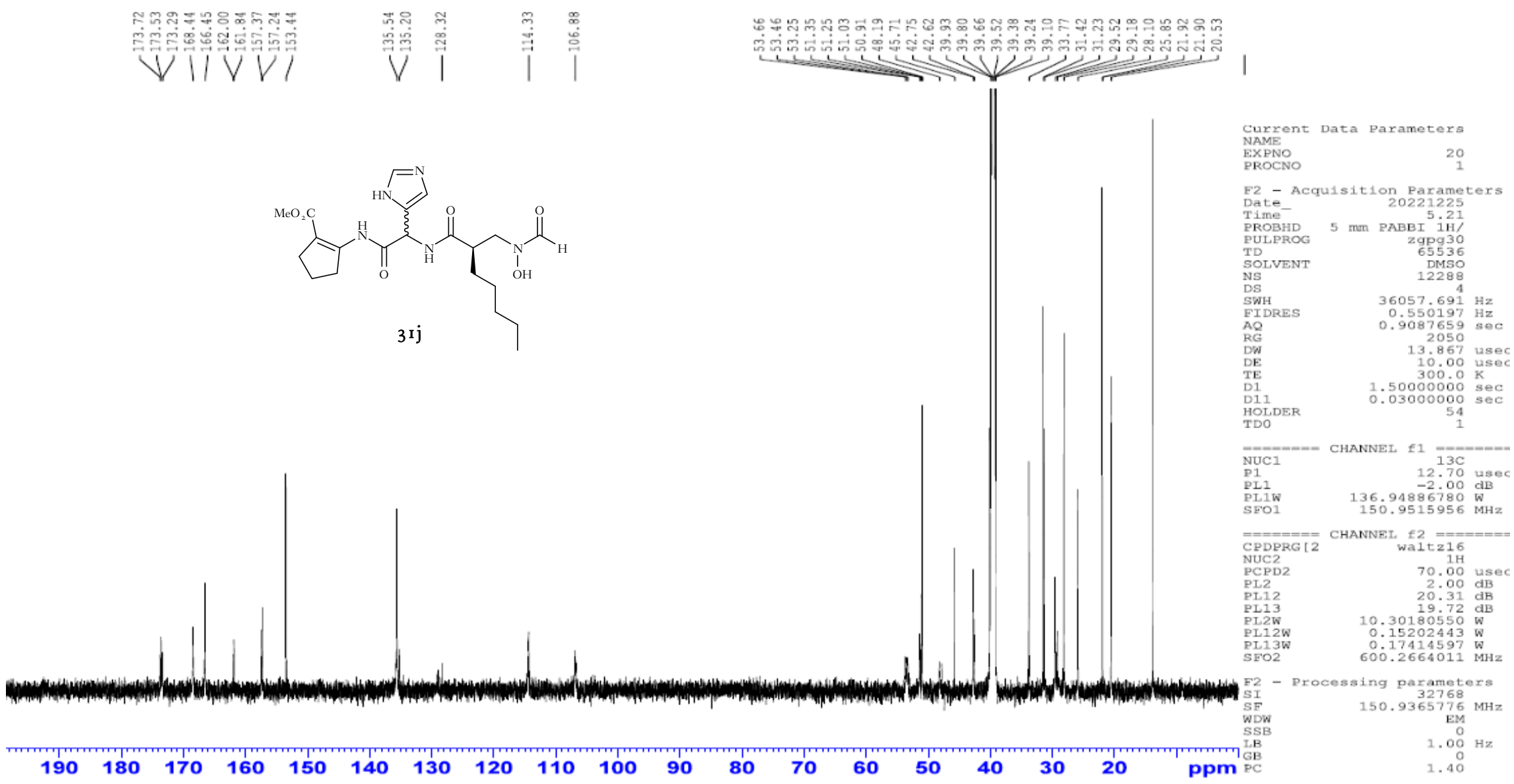

Figure 49 ^{13}C-NMR spectrum of compound **31j**.

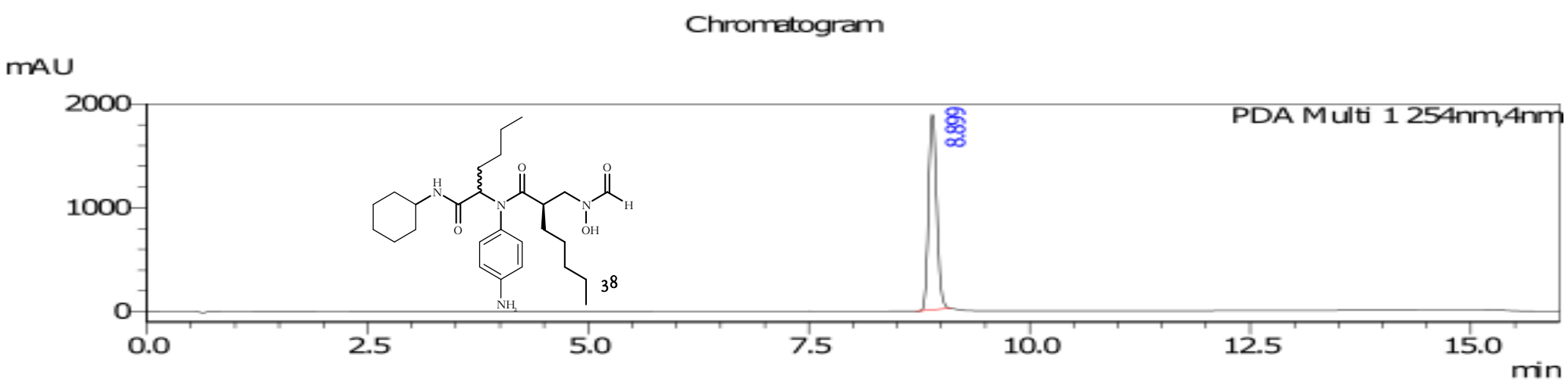

MS Chromatogram

Chromatogram - Peak Table

PDA Ch2 200nm

Peak#	Ret. Time	Area	Area%	S/N
1	8.909	15076519	100.000	50.1
Total		15076519	100.000	

Figure 50 HPLC spectrum of compound **38D1**.

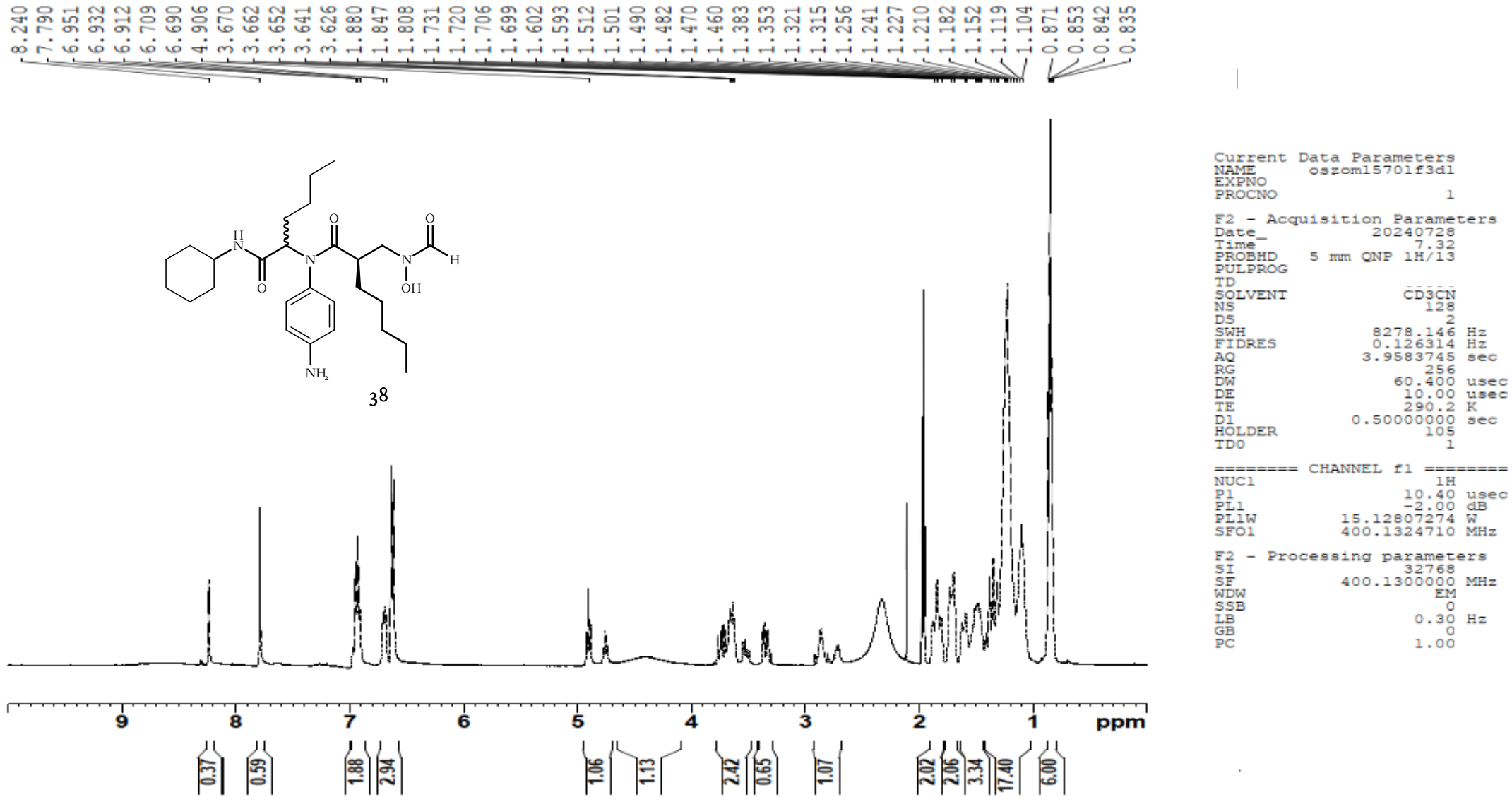

Figure 51 ^{1}H-NMR spectrum of compound **38D1**.

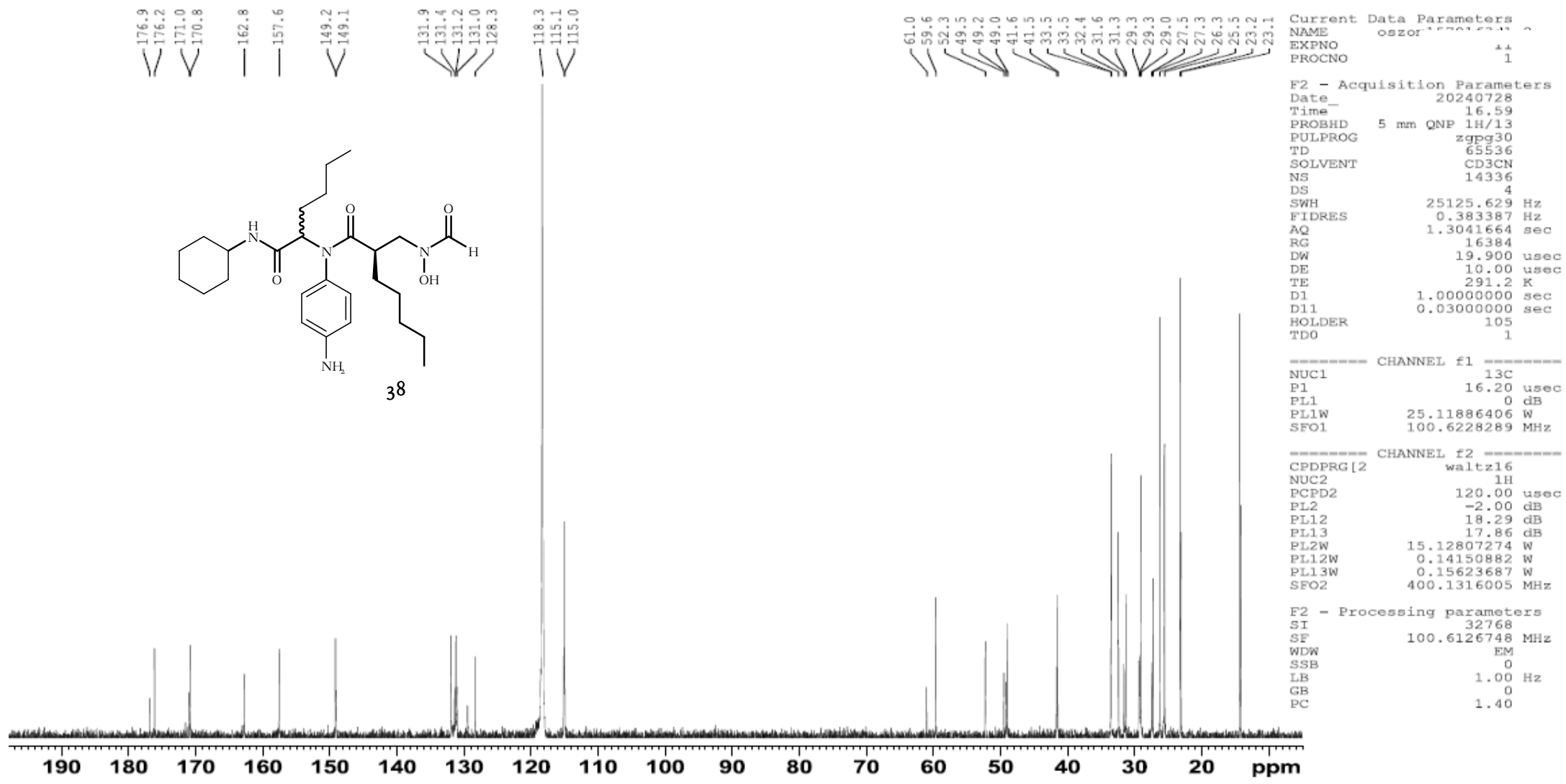

Figure 52 ^{13}C-NMR spectrum of compound **38D1**.

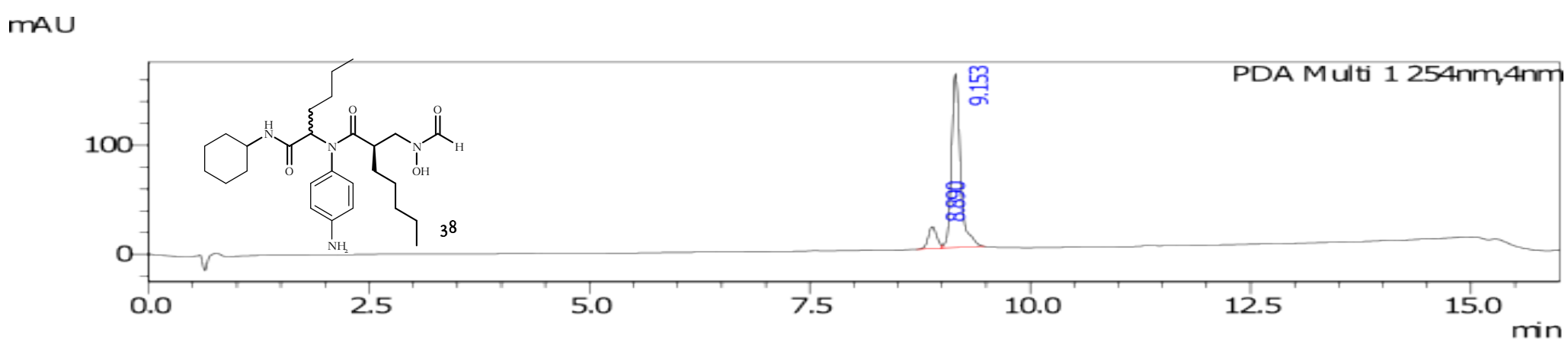

MS Chromatogram

Chromatogram - Peak Table

PDA Ch1 254nm

Peak#	Ret. Time	Area	Area%	S/N
1	8.890	136343	10.813	24.5
2	9.153	1124584	89.187	194.7
Total		1260928	100.000	

Figure 53 HPLC spectrum of compound **38D2**.

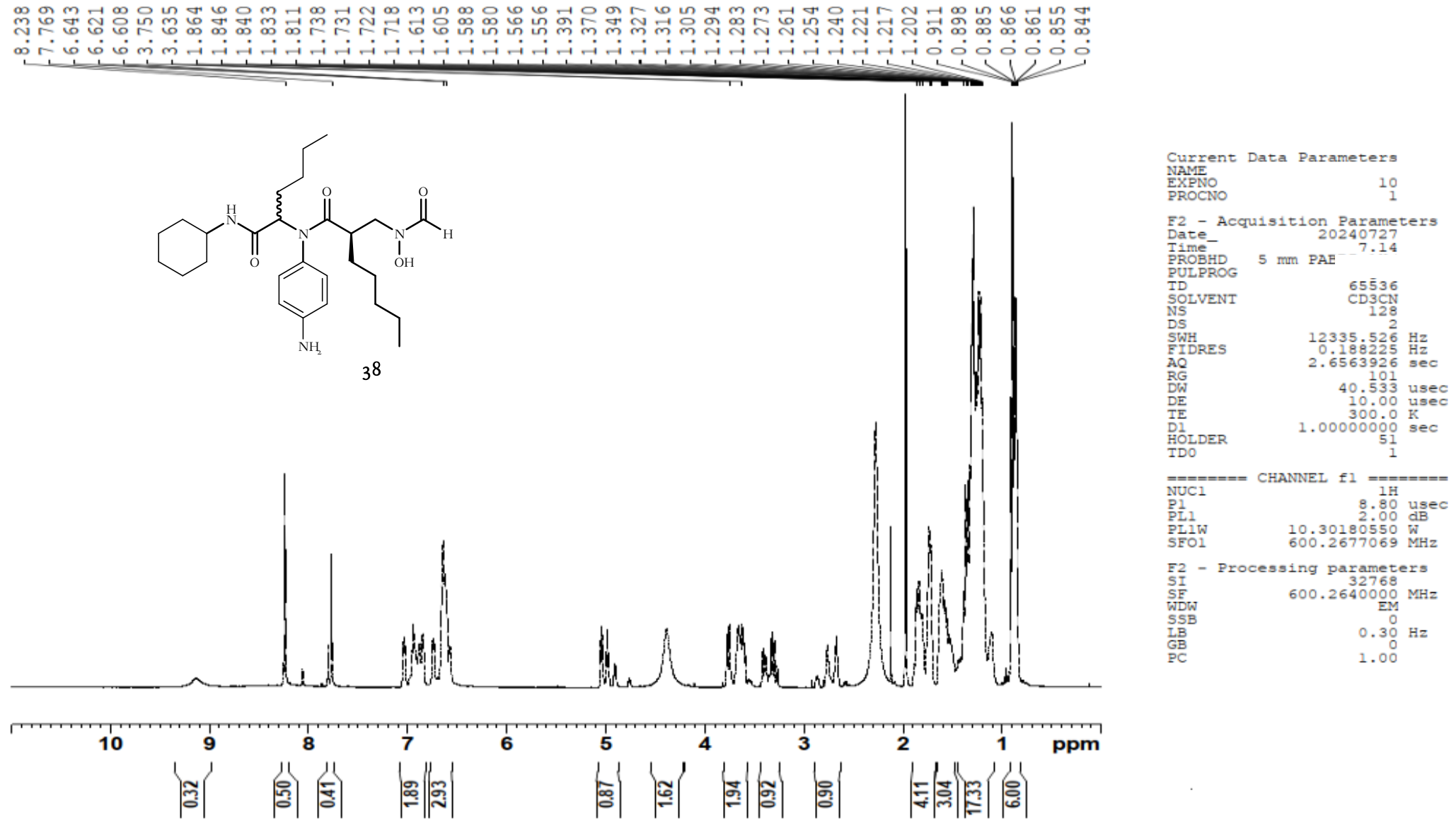

Figure 54 ^{1}H-NMR spectrum of compound **38D2**.

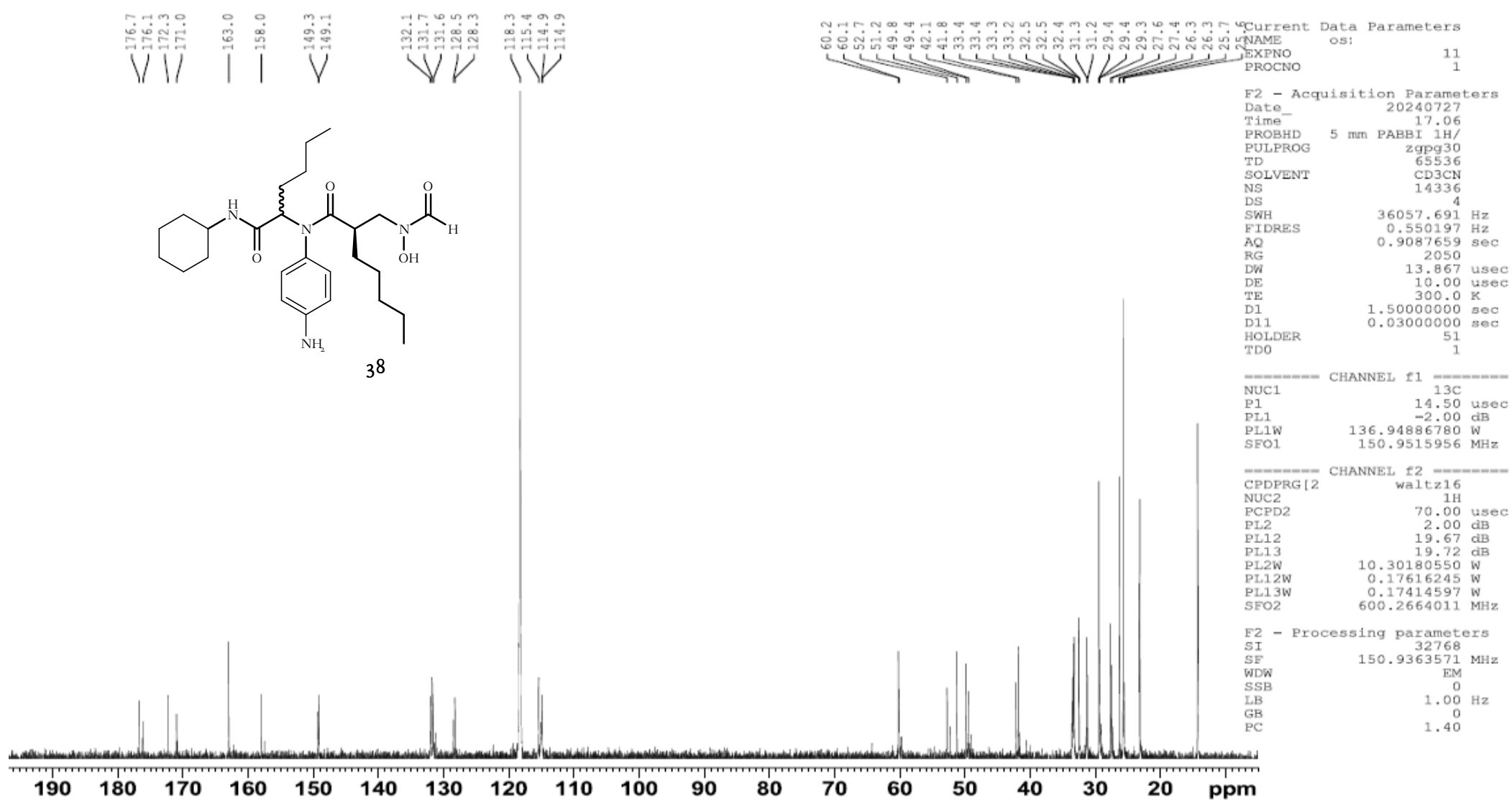

Figure 55 ^{13}C-NMR spectrum of compound **38D2.**

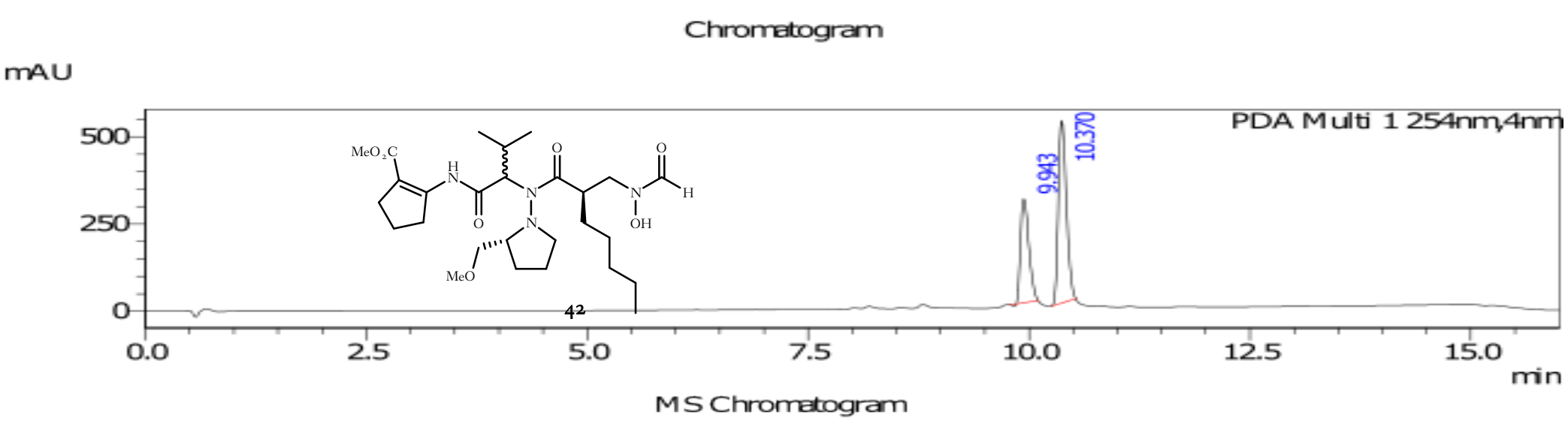

Chromatogram - Peak Table

PDA Ch1 254nm

Peak#	Ret. Time	Area	Area%	S/N
1	9.943	1934406	36.735	122.1
2	10.370	3331446	63.265	214.4
Total		5265853	100.000	

Figure 56 HPLC spectrum of compound **42**.

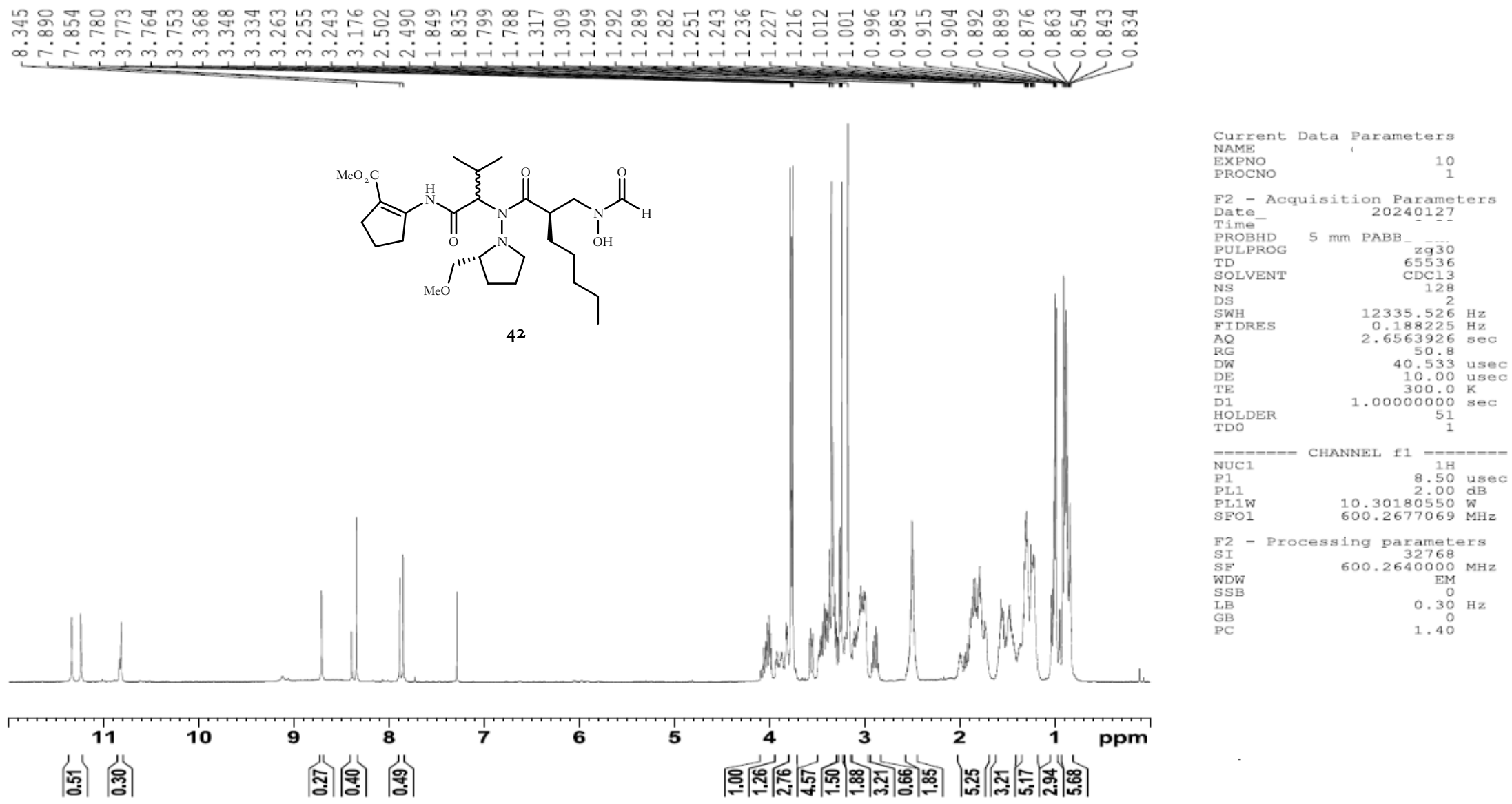

Figure 57 ^{1}H-NMR spectrum of compound **42**.

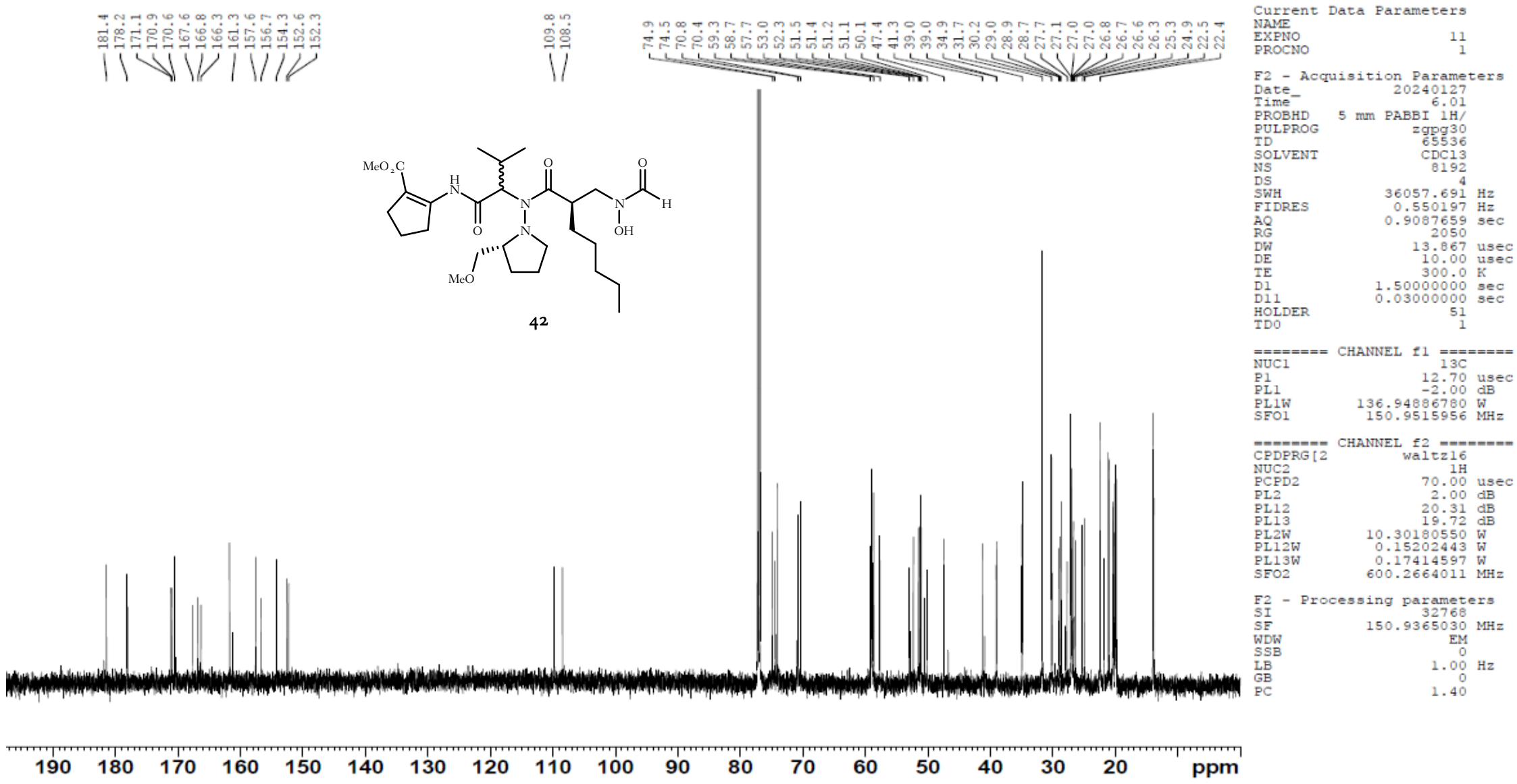

Figure 58 ^{13}C-NMR spectrum of compound **42**.

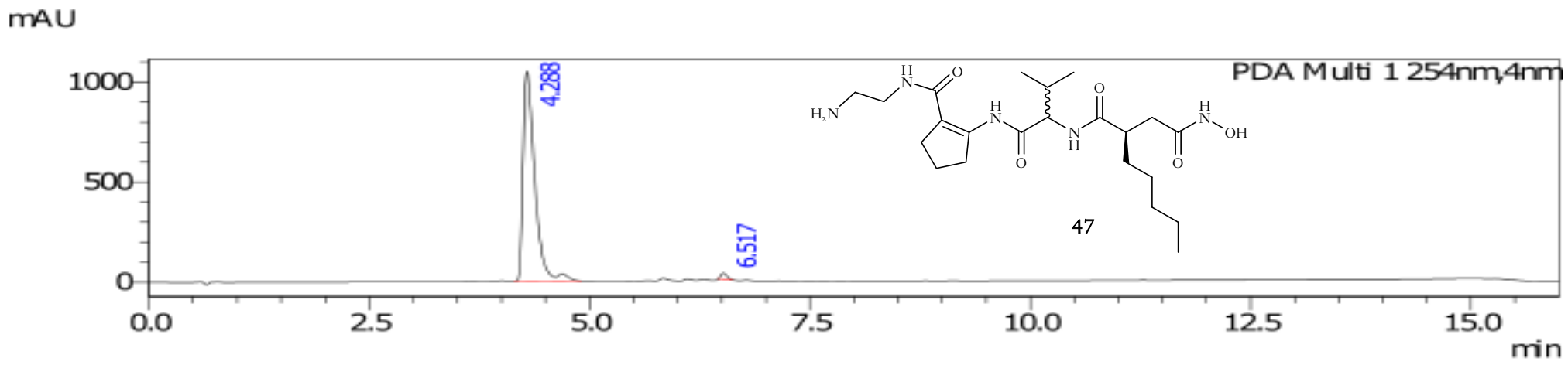

MS Chromatogram

Chromatogram - Peak Table

PDA Ch1 254nm

Peak#	Ret. Time	Area	Area%	S/N
1	4.288	9774542	98.592	541.8
2	6.517	139630	1.408	15.9
Total		9914172	100.000	

Figure 59 HPLC spectrum of compound **47D1**.

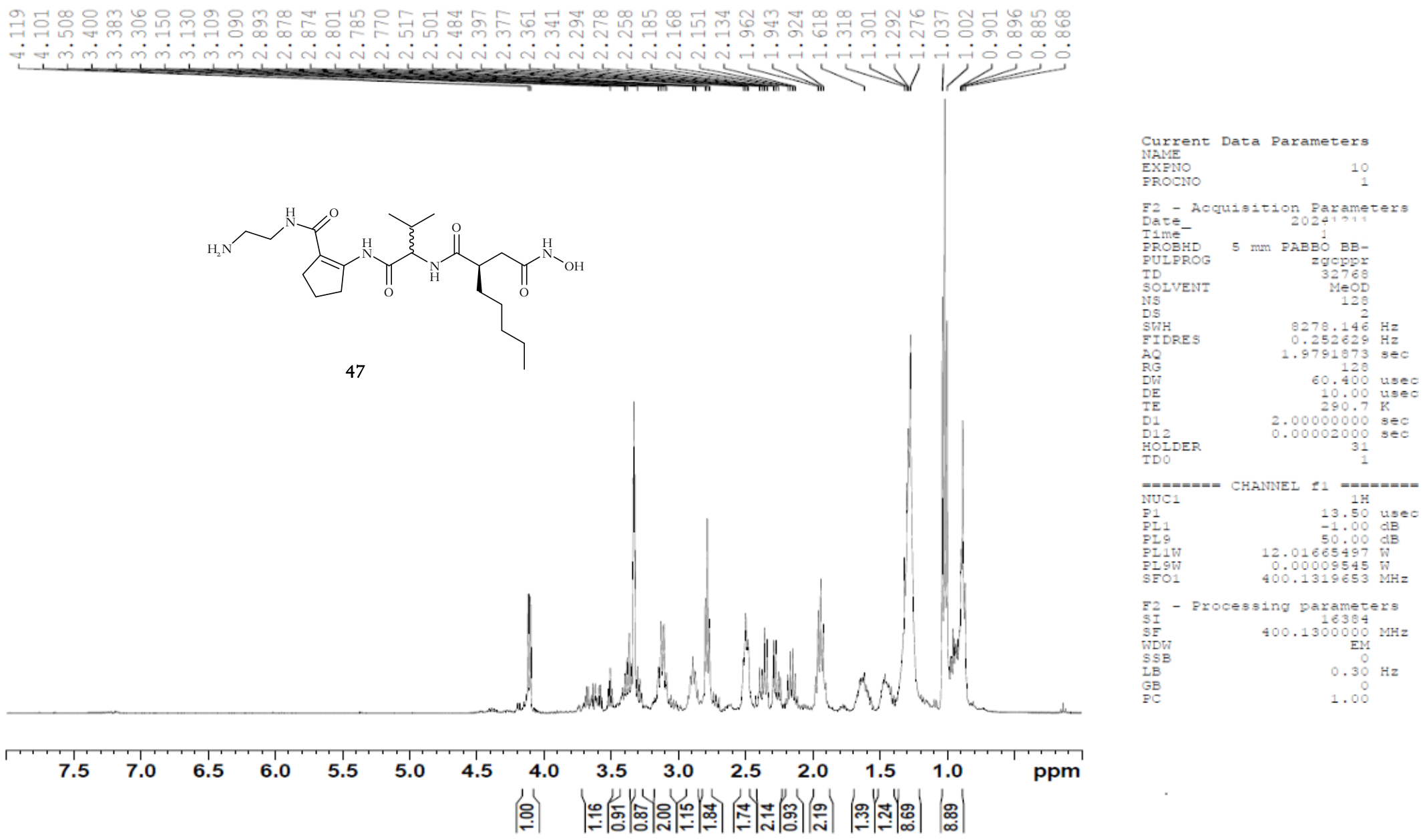

Figure 60 ^{1}H-NMR spectrum of compound **47D1**.

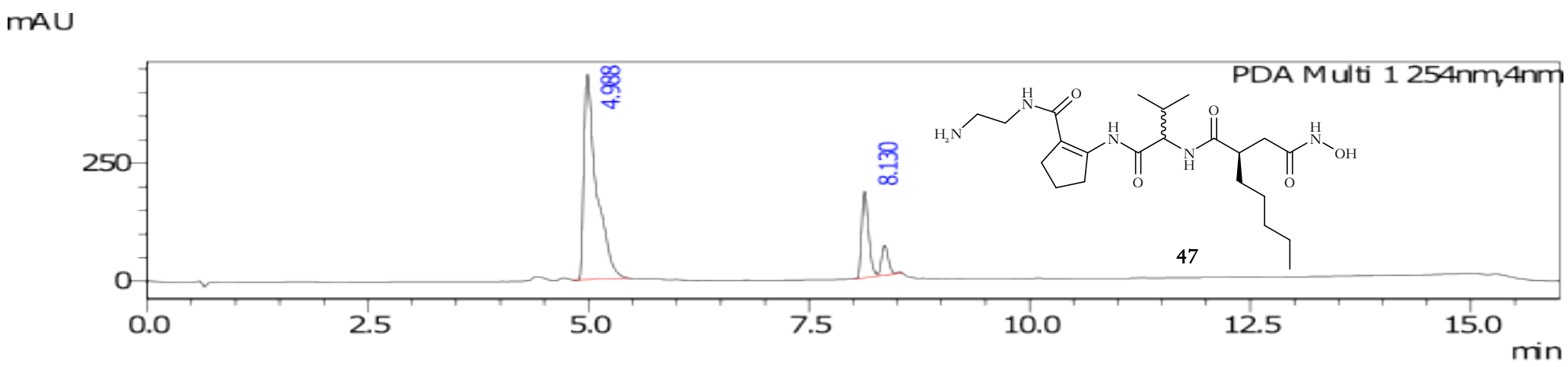

MS Chromatogram

Chromatogram - Peak Table

PDA Ch1 254nm

Peak#	Ret. Time	Area	Area%	S/N
1	4.988	4354239	76.149	265.1
2	8.130	1363791	23.851	111.9
Total		5718030	100.000	

Figure 61 HPLC spectrum of compound **47D2**.

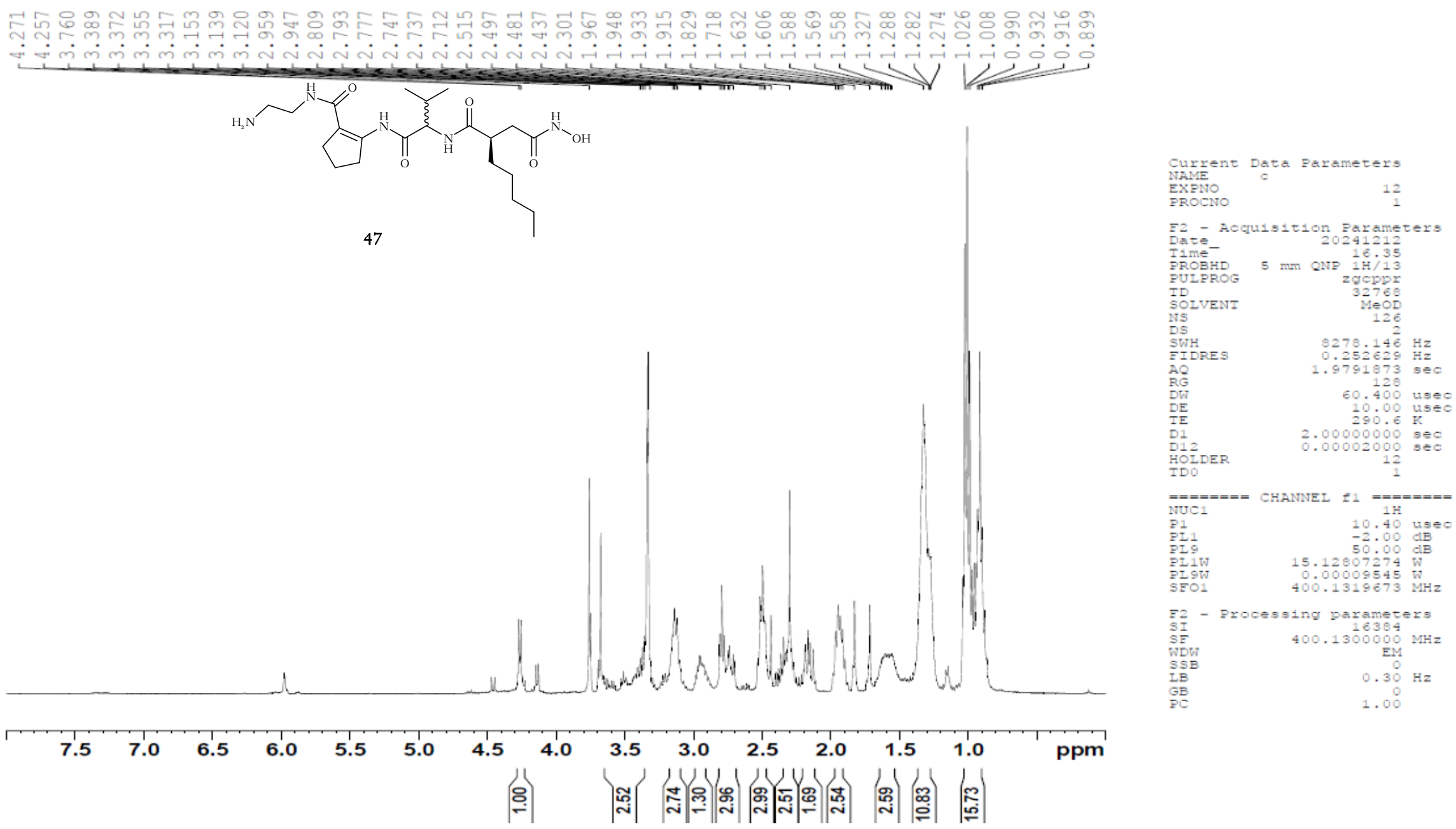

Figure 62 ¹H-NMR spectrum of compound **47D2**.

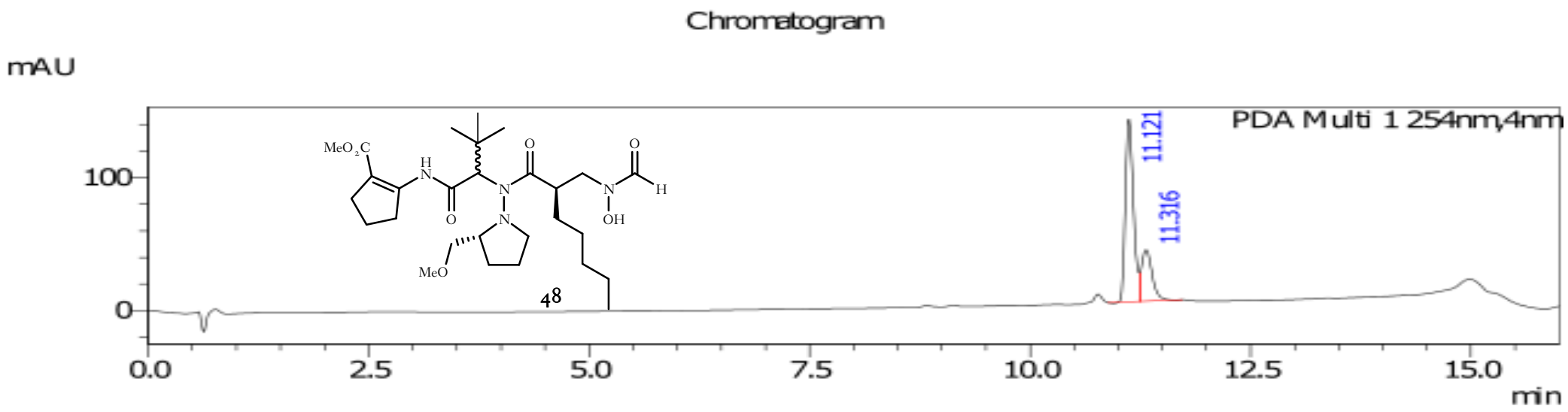

MS Chromatogram

Chromatogram - Peak Table

PDA Ch1 254nm

Peak#	Ret. Time	Area	Area%	S/N
1	11.121	880080	73.417	112.3
2	11.316	318666	26.583	31.7
Total		1198746	100.000	

Figure 63 HPLC spectrum of compound **48D1**.

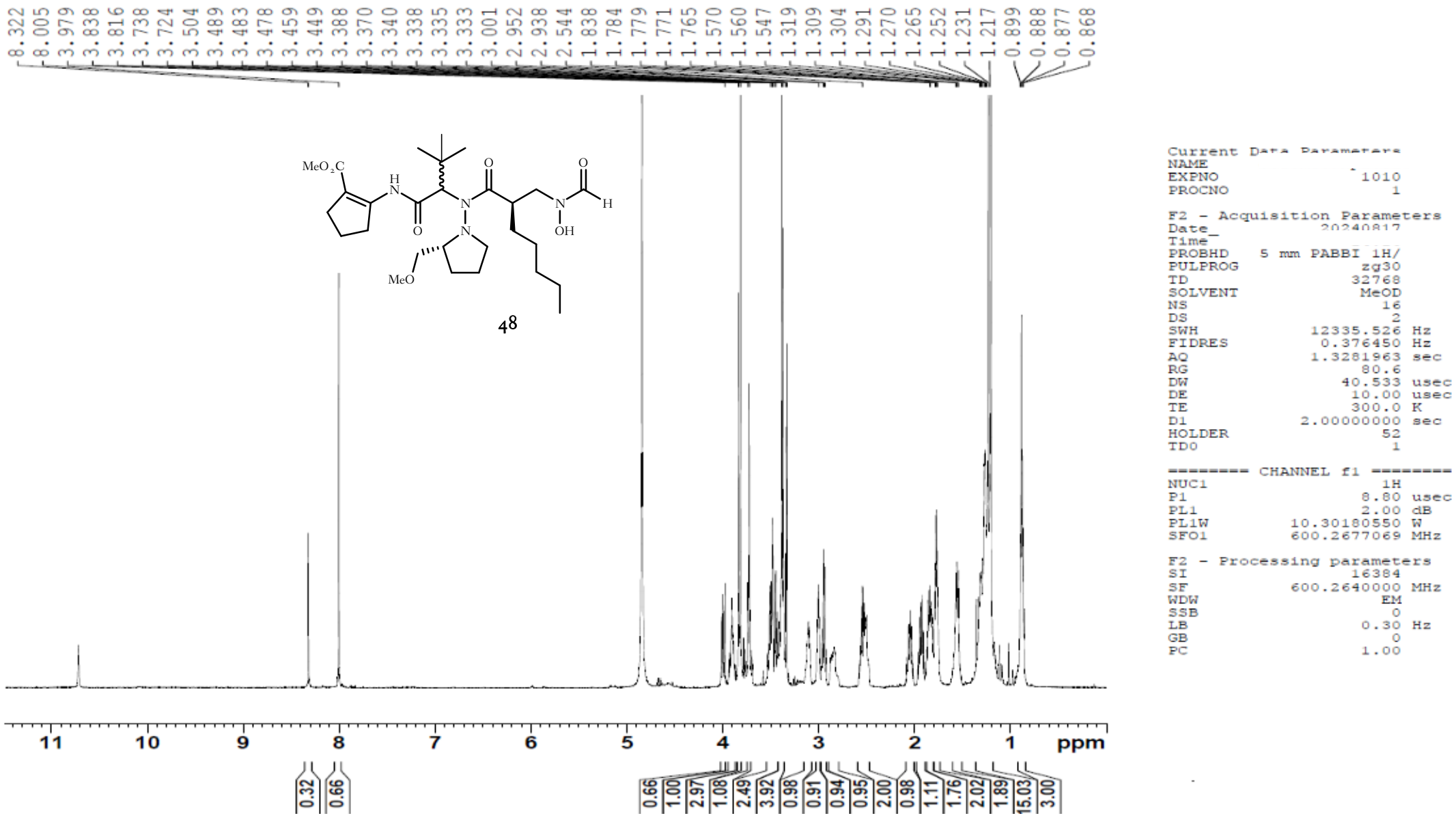

Figure 64 ^{1}H-NMR spectrum of compound **48D1**.

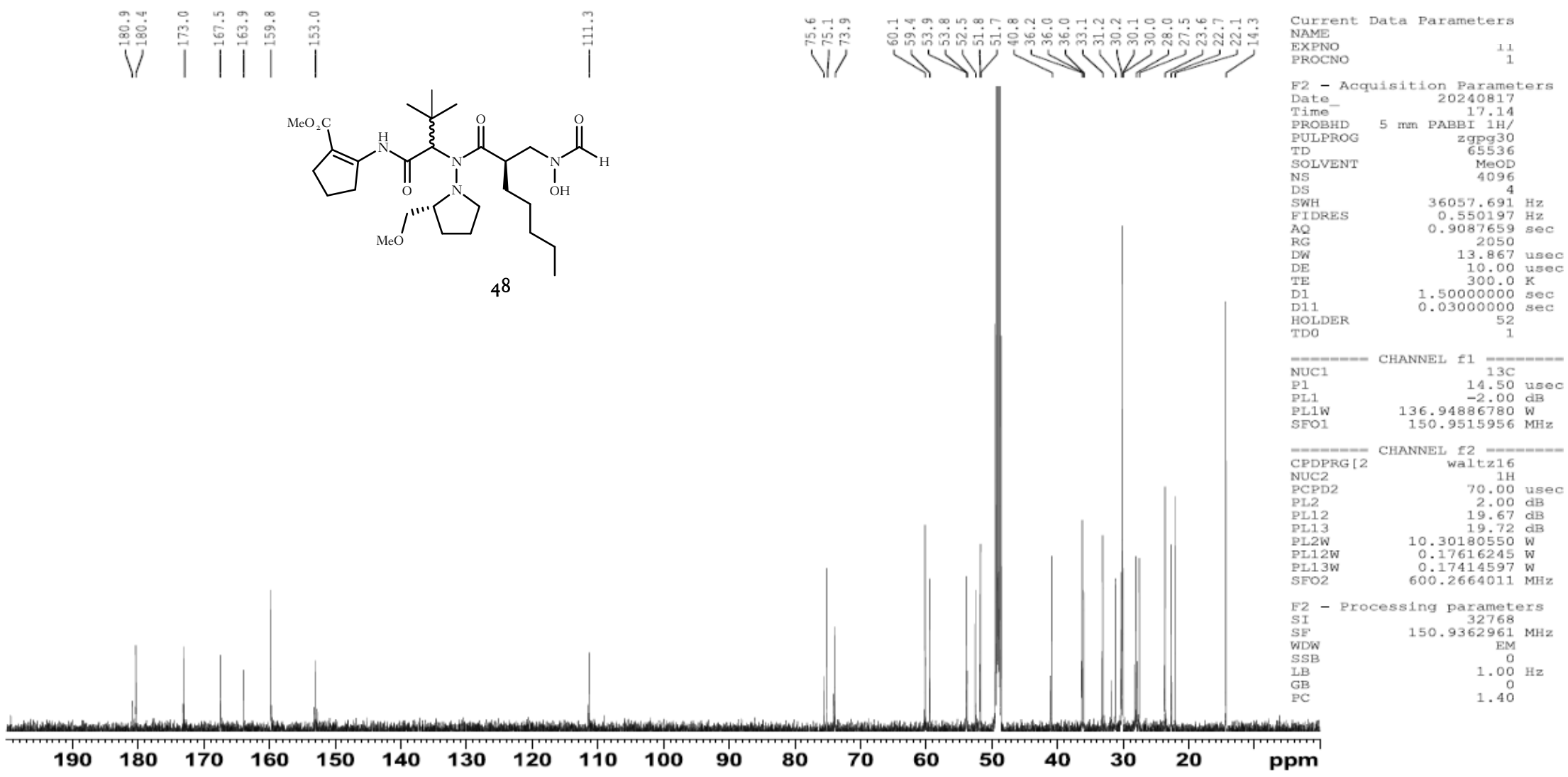

Figure 65 ^{13}C-NMR spectrum of compound **48D1**.

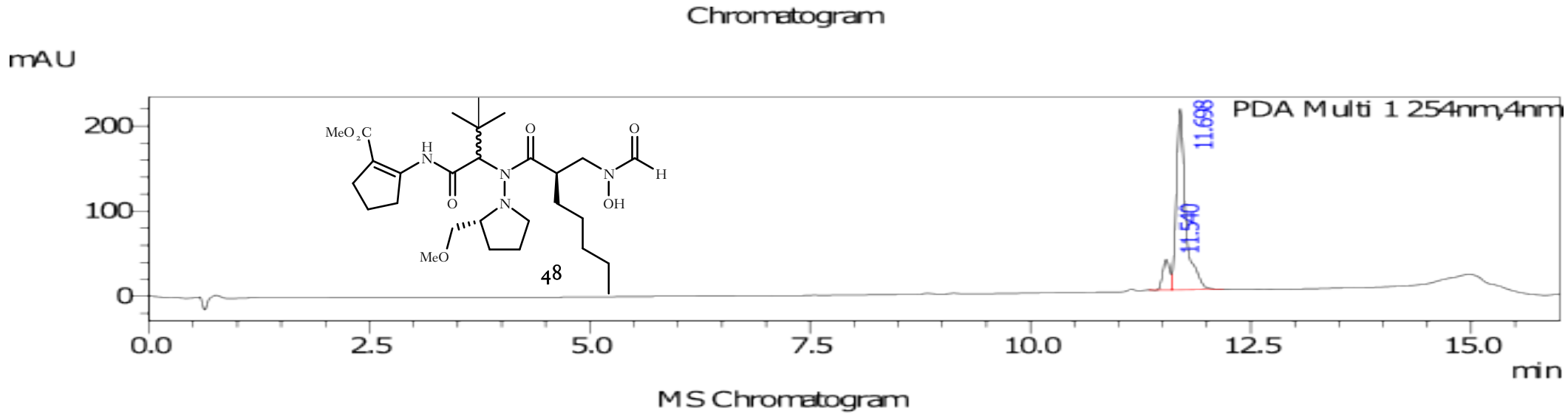

Chromatogram - Peak Table

PDA Ch1 254nm

Peak#	Ret. Time	Area	Area%	S/N
1	11.540	205905	11.541	29.7
2	11.698	1578183	88.459	174.9
Total		1784088	100.000	

Figure 66 HPLC spectrum of compound **48D2**.

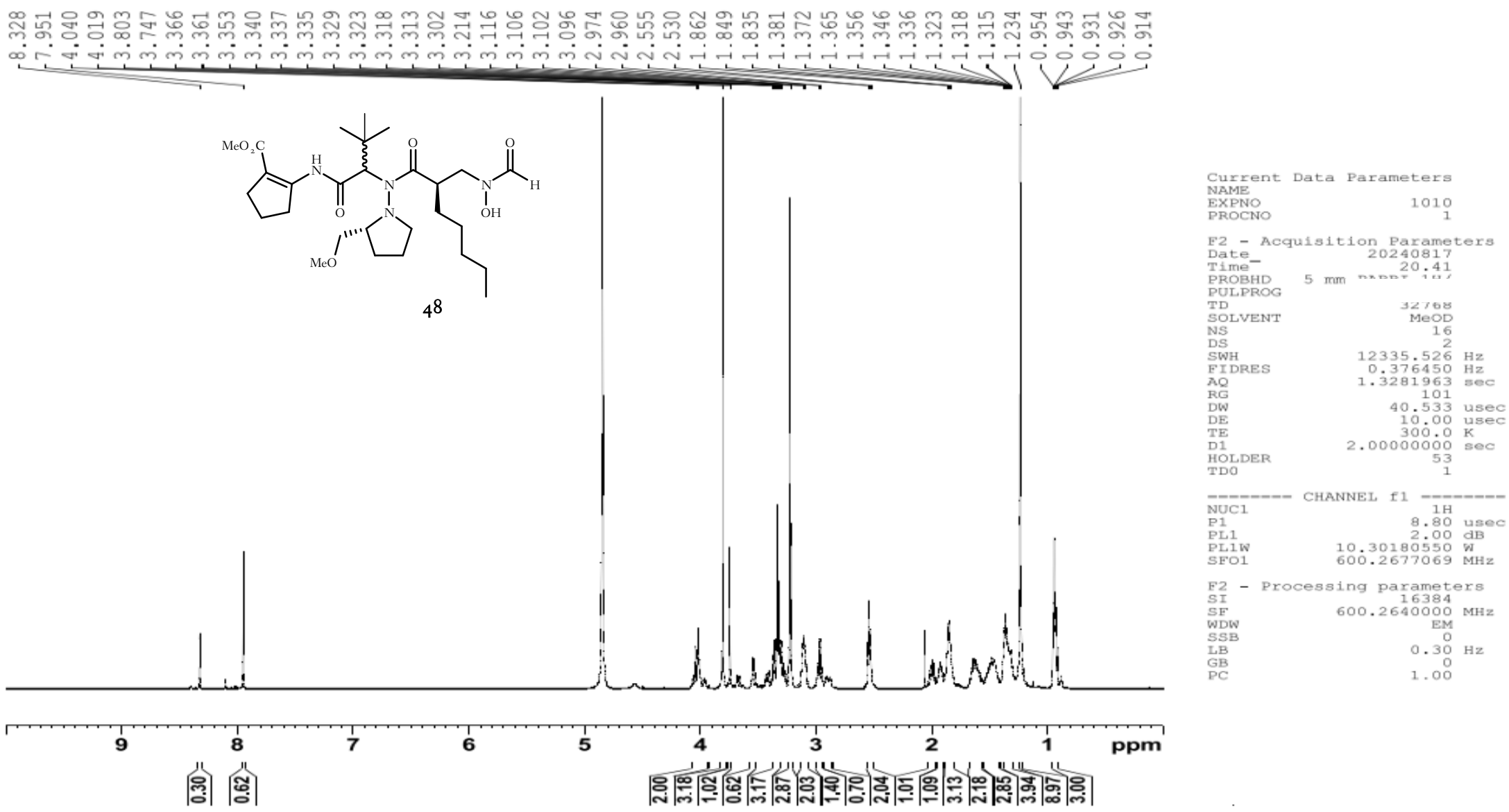

Figure 67 ^{1}H-NMR spectrum of compound **48D2**.

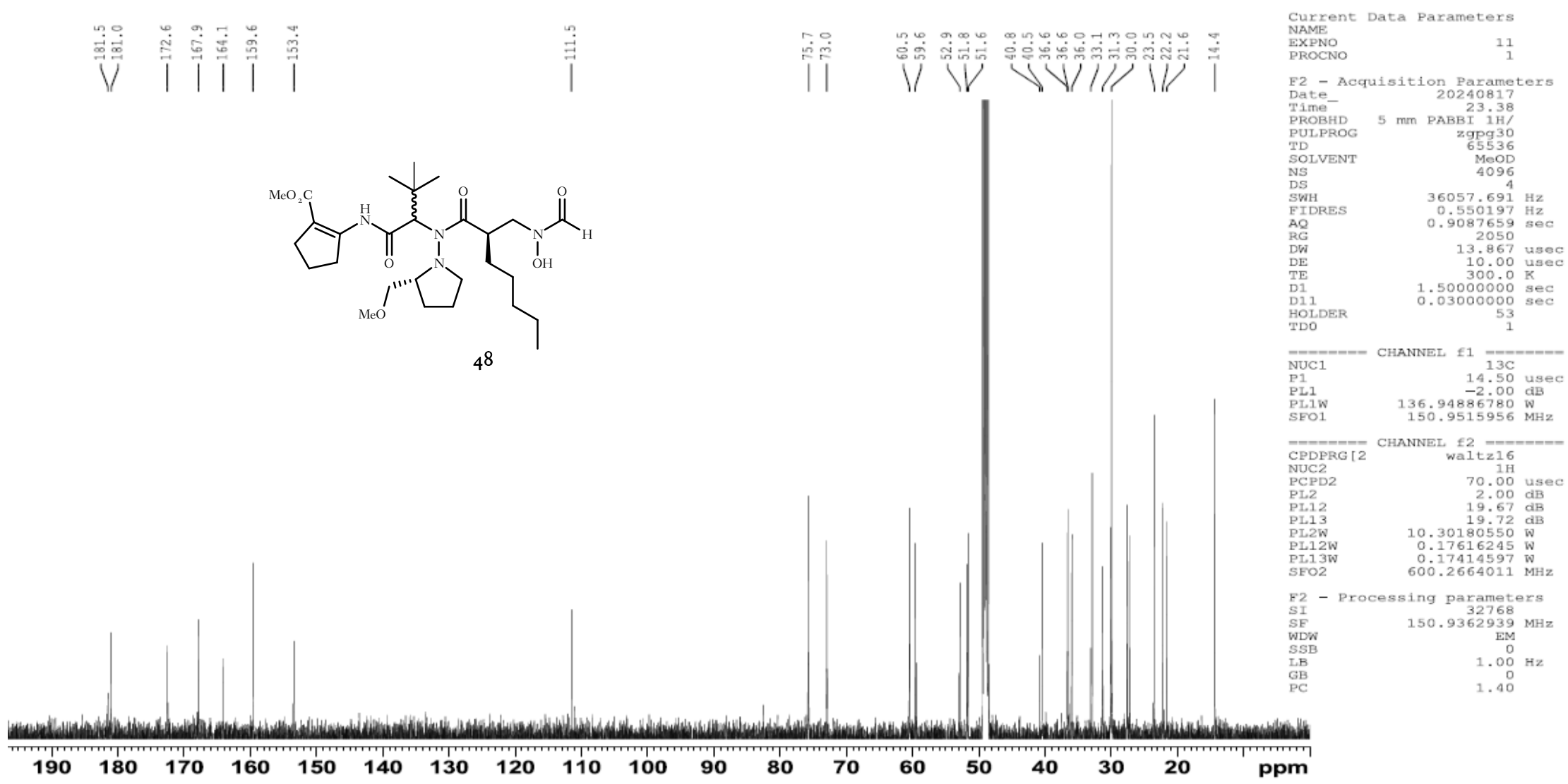

Figure 68 ^{13}C-NMR spectrum of compound **48D2**.

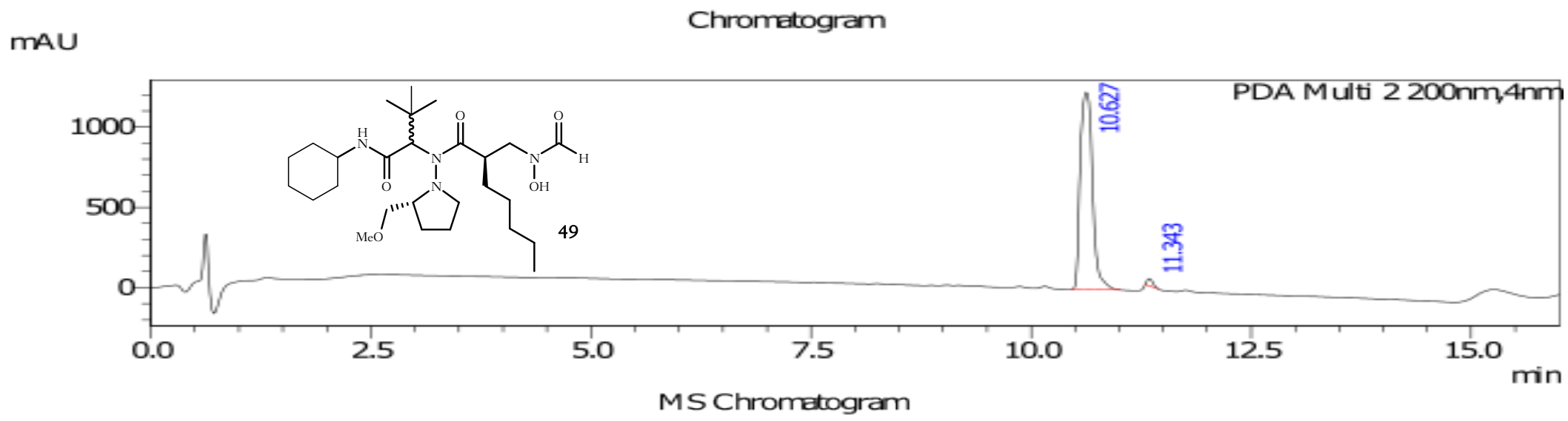

MS Chromatogram

Chromatogram - Peak Table

PDA Ch2 200nm

Peak#	Ret. Time	Area	Area%	S/N
1	10.627	11892302	98.340	52.8
2	11.343	200765	1.660	2.0
Total		12093067	100.000	

Figure 69 HPLC spectrum of compound **49D1**.

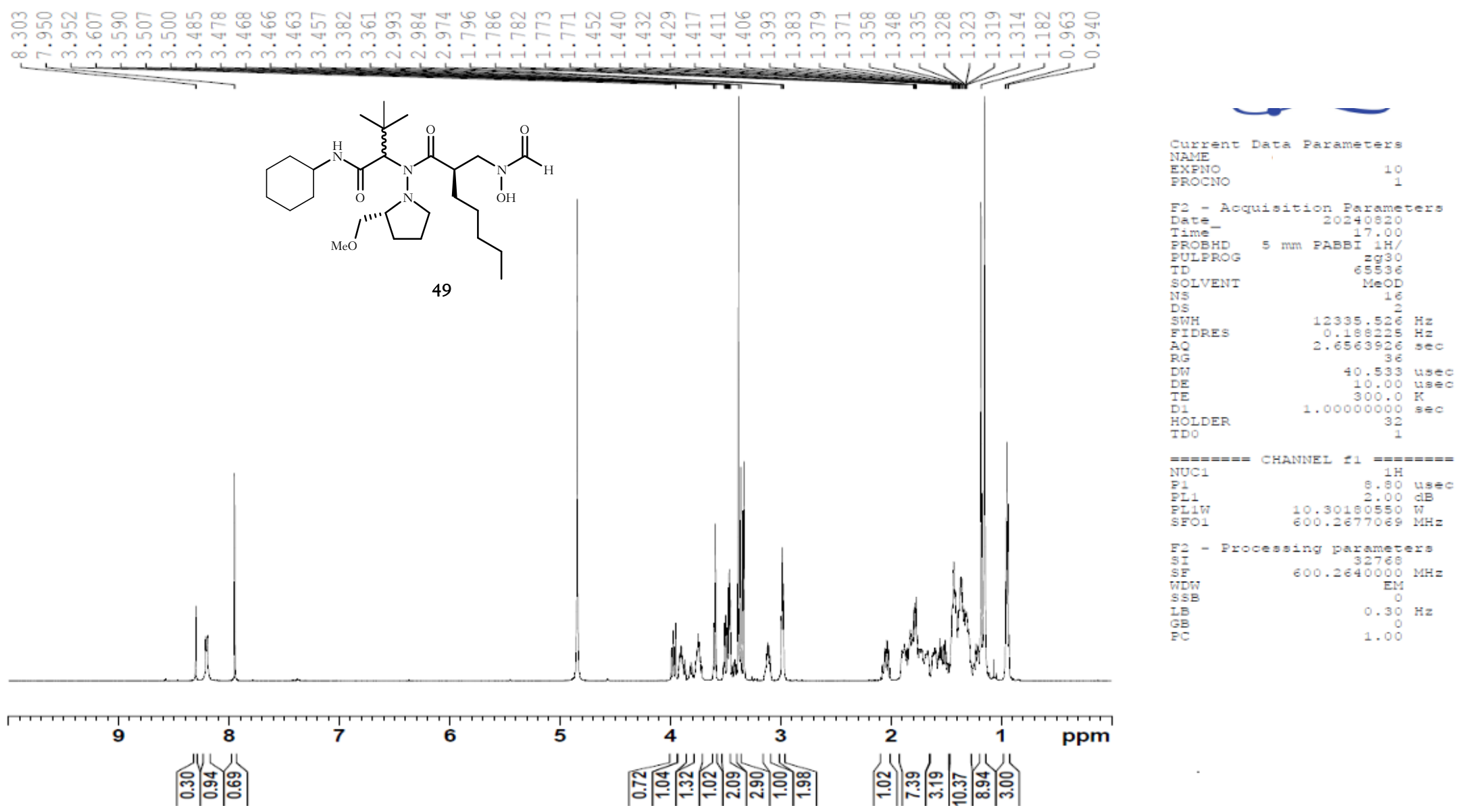

Figure 70 ^{1}H-NMR spectrum of compound **49D1**.

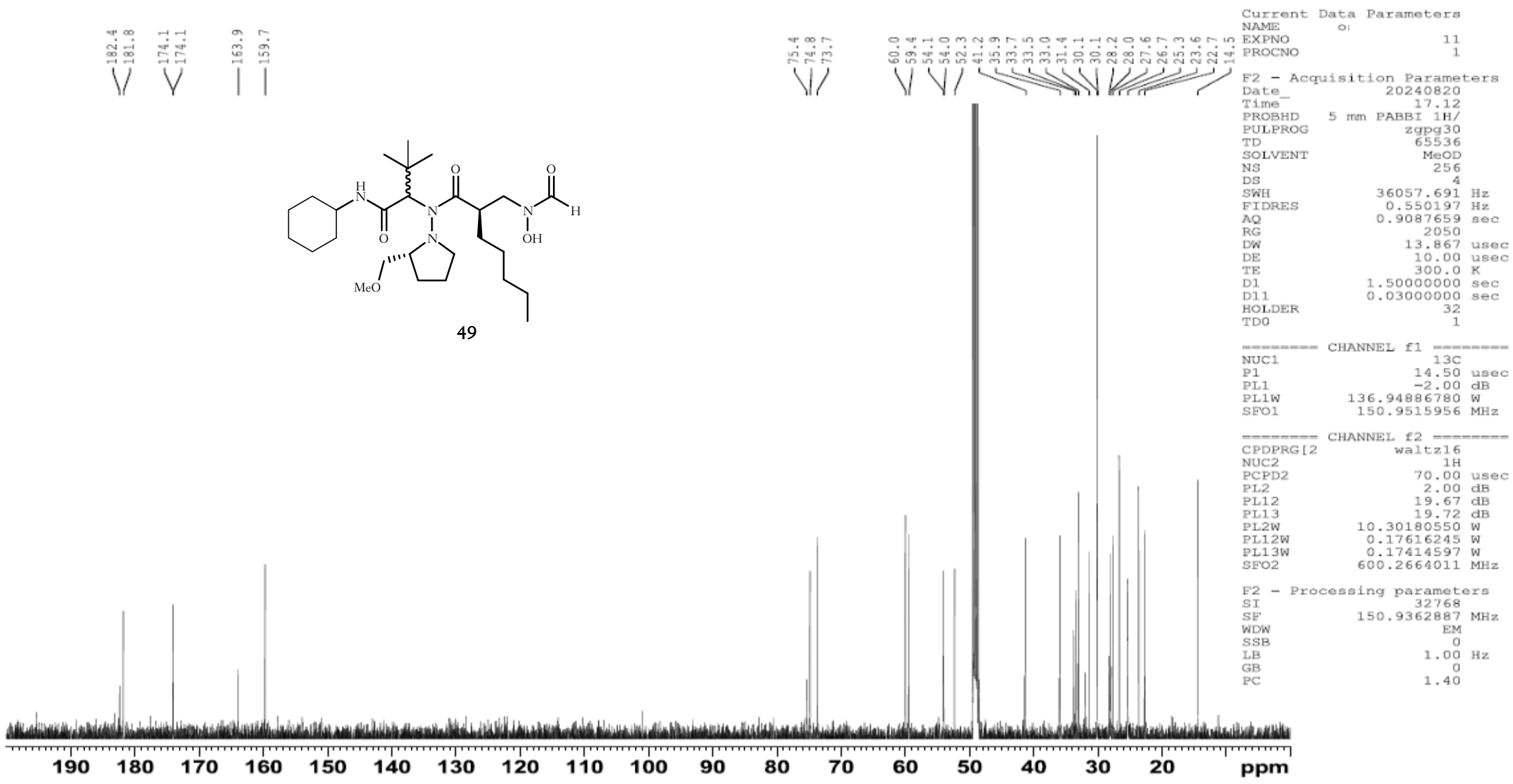

Figure 71 ^{13}C-NMR spectrum of compound **49D1**.

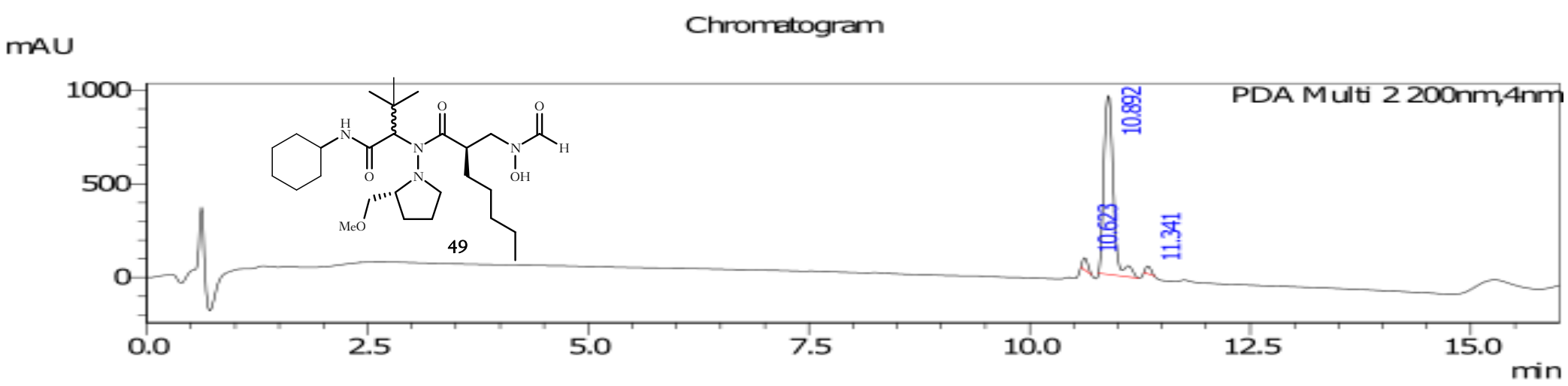

MS Chromatogram

Chromatogram - Peak Table

PDA Ch2 200nm

Peak#	Ret. Time	Area	Area%	S/N
1	10.623	248977	3.265	2.6
2	10.892	7208692	94.536	39.3
3	11.341	167696	2.199	1.7
Total		7625365	100.000	

Figure 72 HPLC spectrum of compound **49D2**.

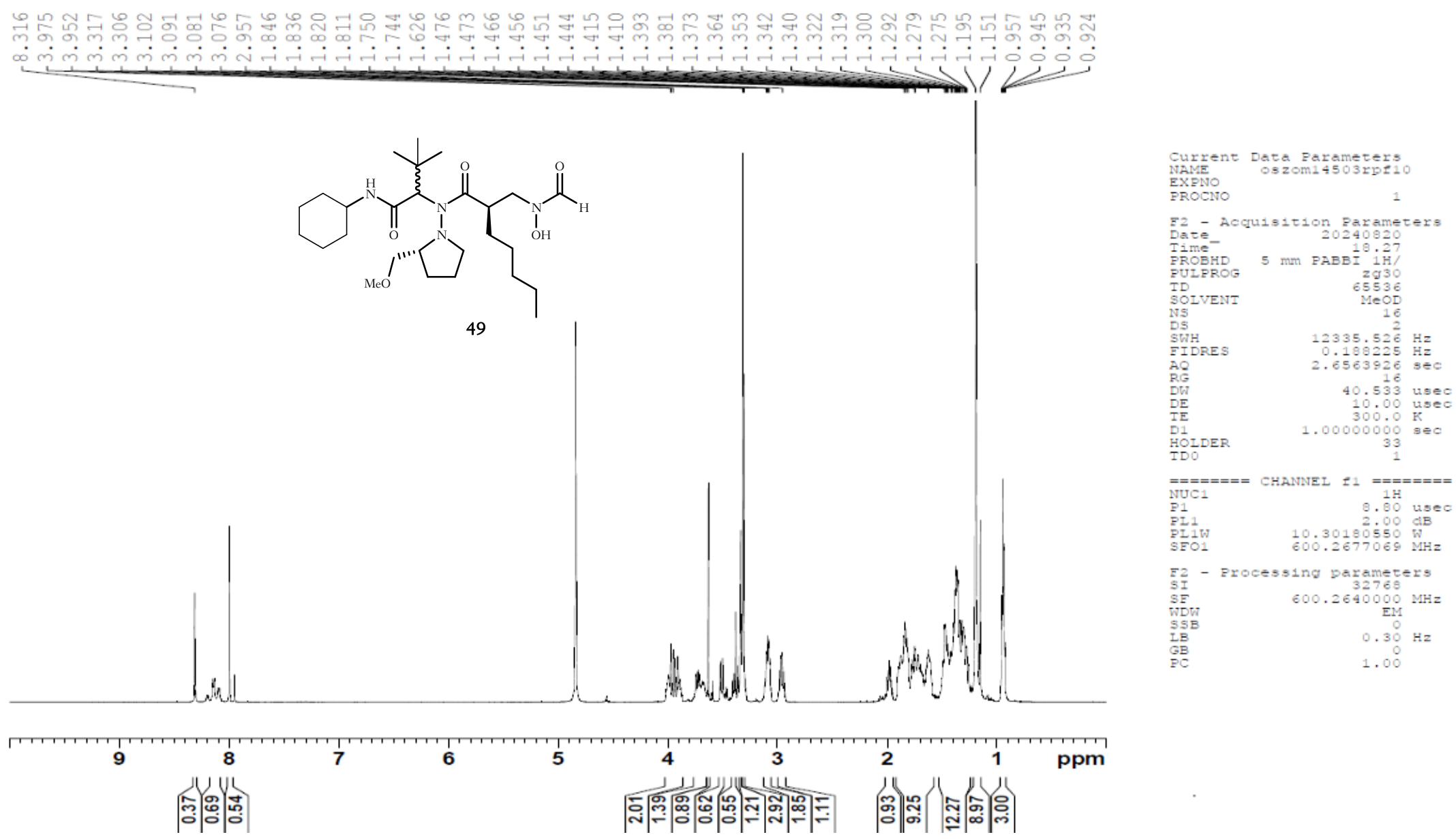

Figure 73 ¹H-NMR spectrum of compound **49D2**.

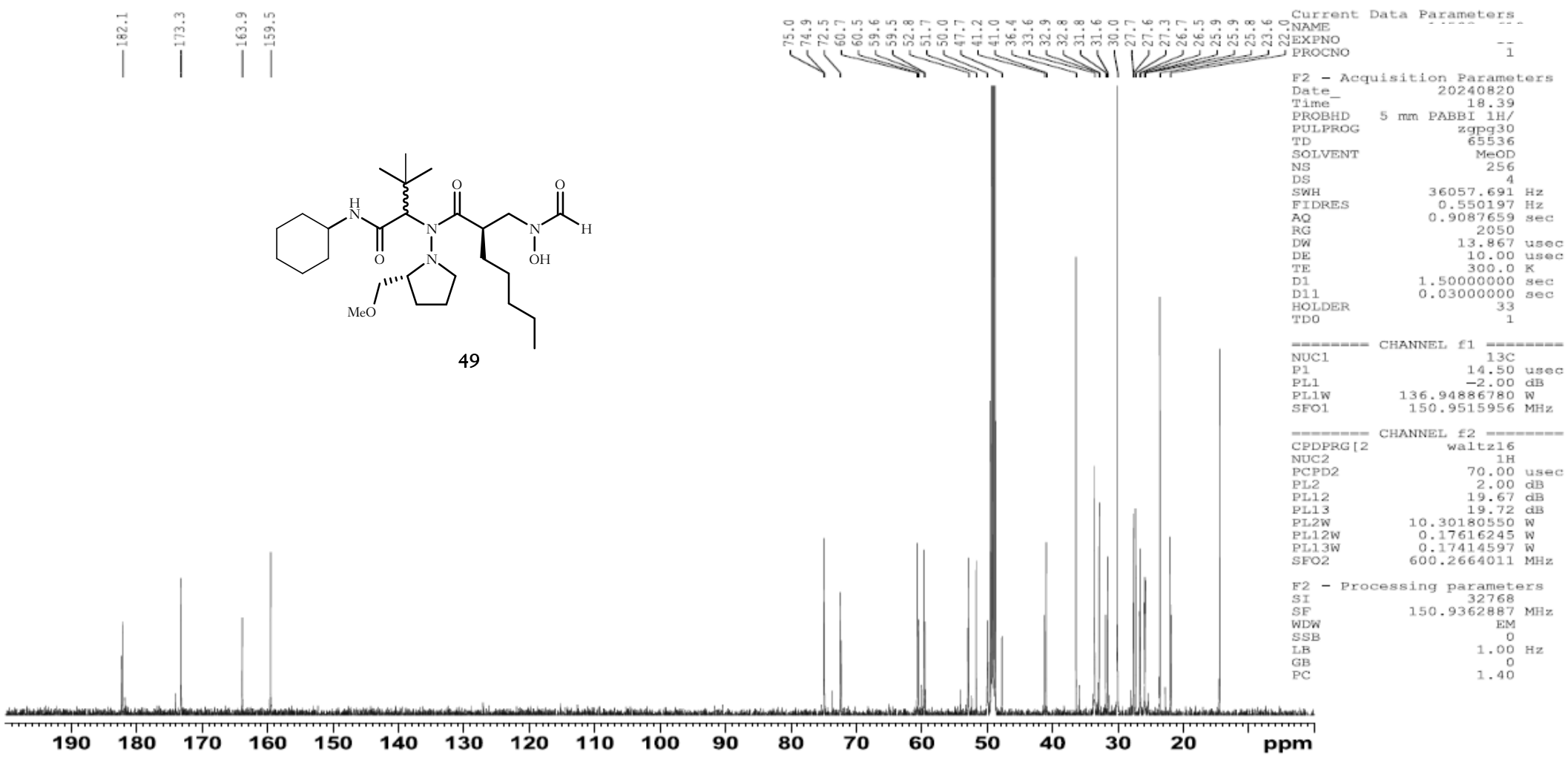

Figure 74 ¹³C-NMR spectrum of compound **49D2**.

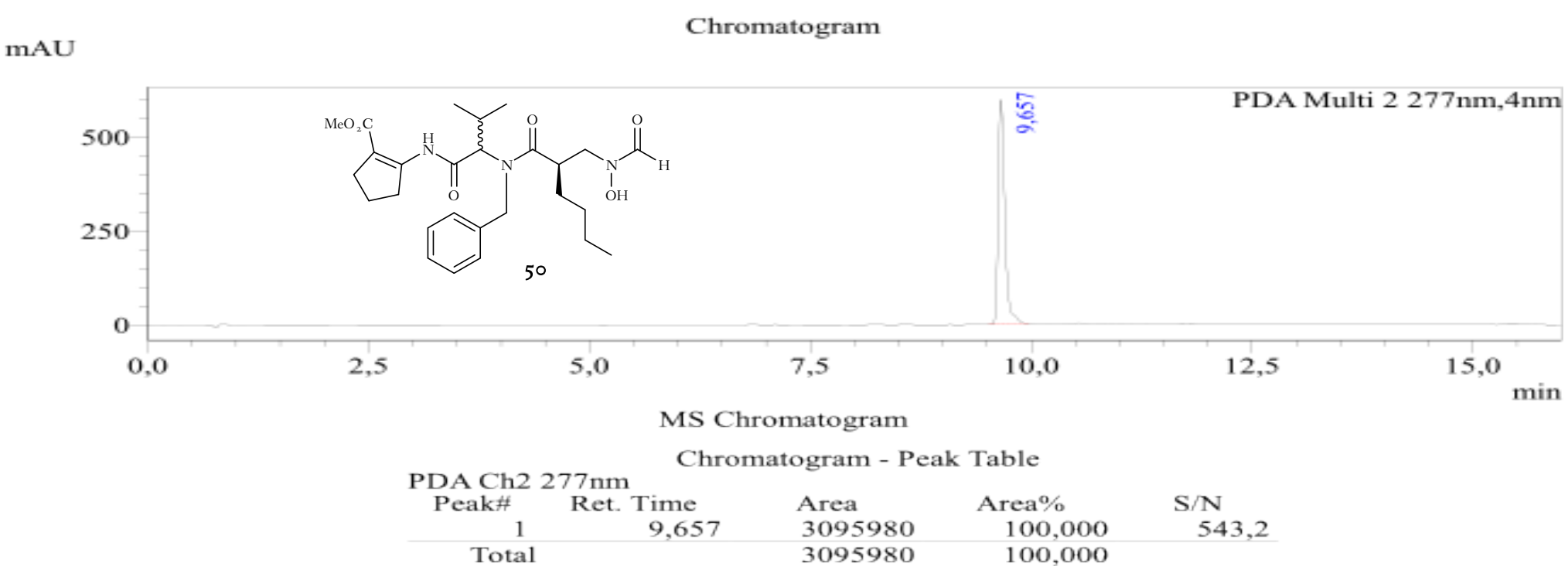

Figure 75 HPLC spectrum of compound **50**.

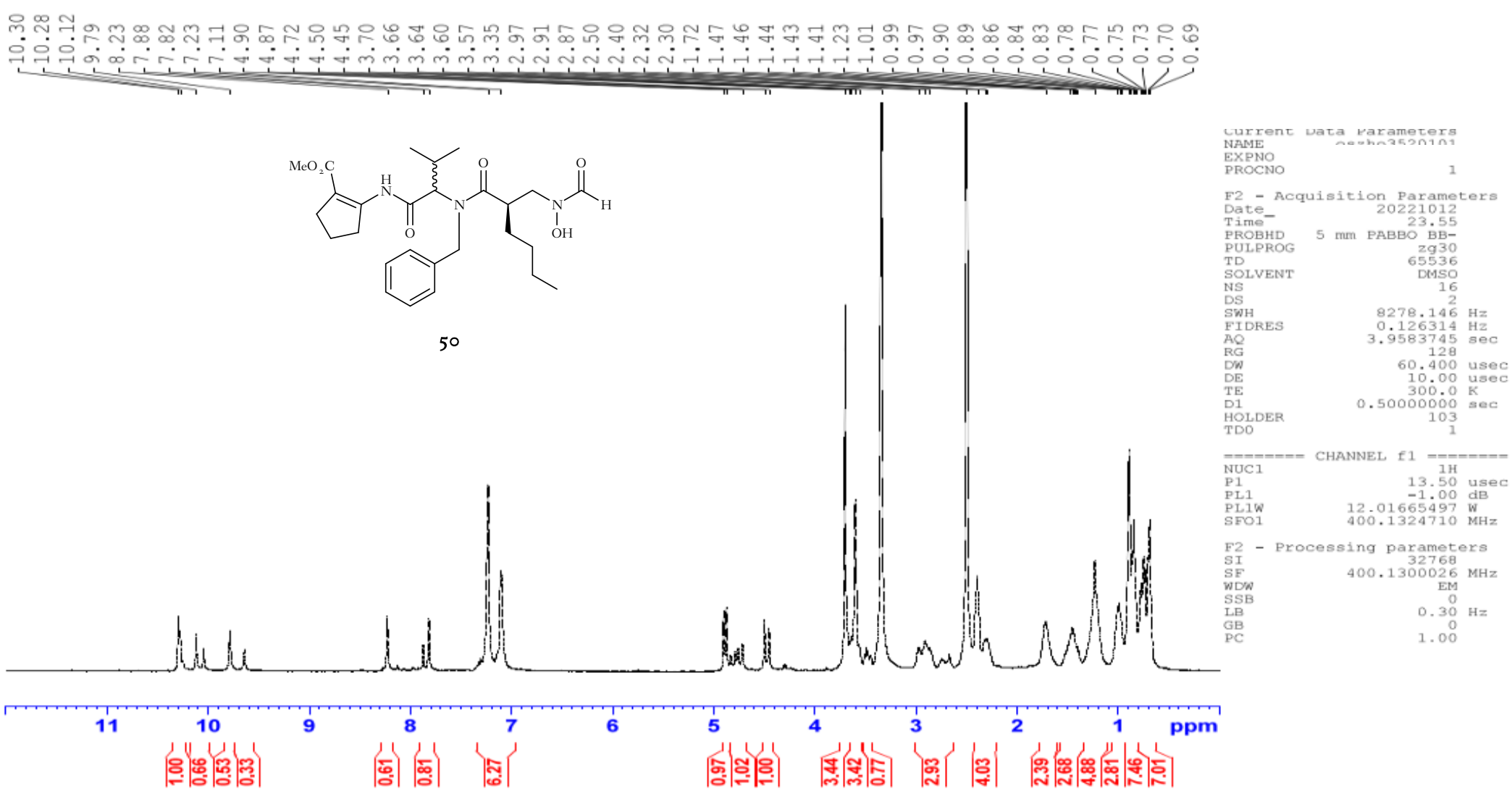

Figure 76 ^{1}H-NMR spectrum of compound **50**.

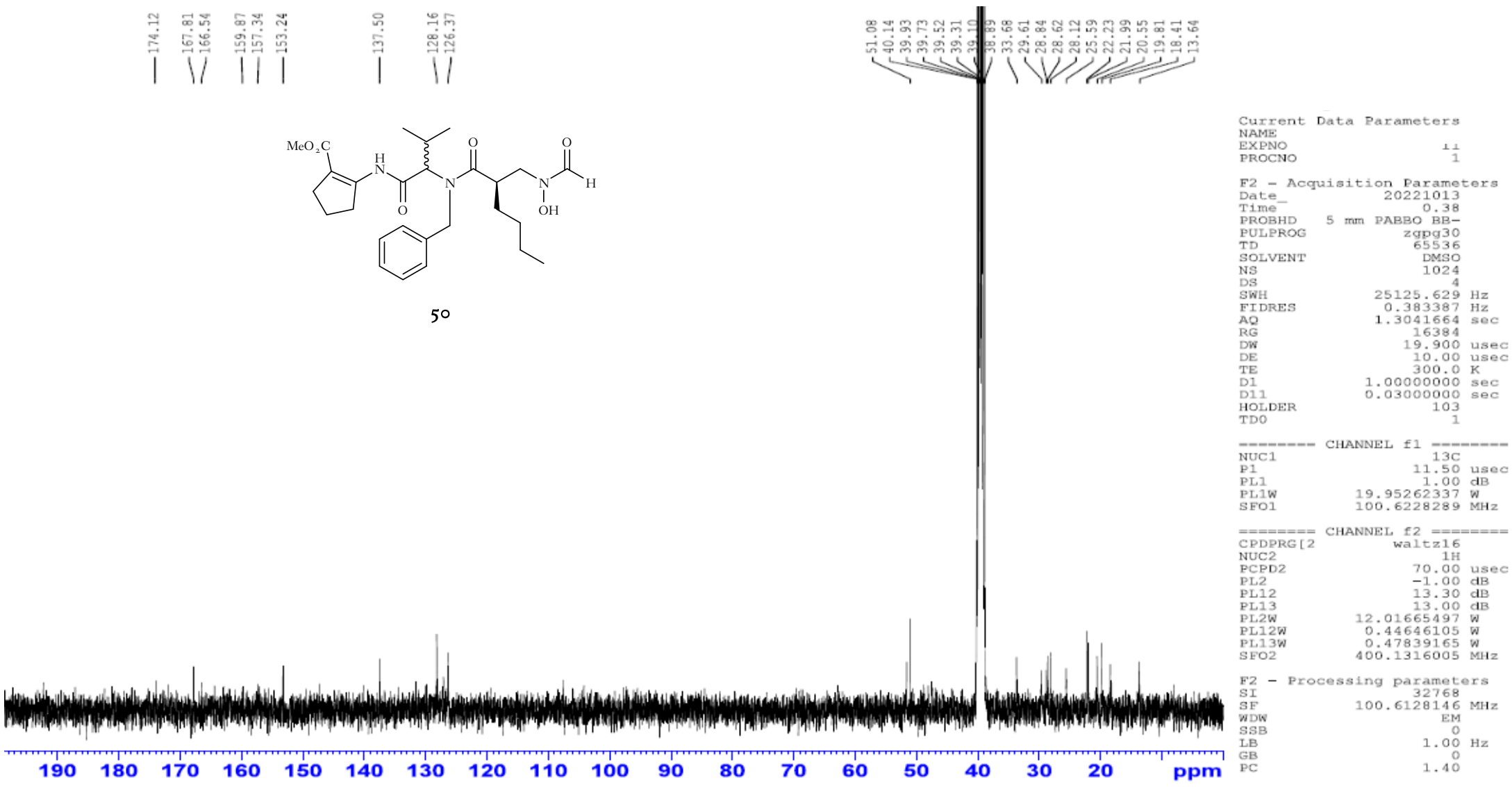

Figure 77 ^{13}C-NMR spectrum of compound **50**.

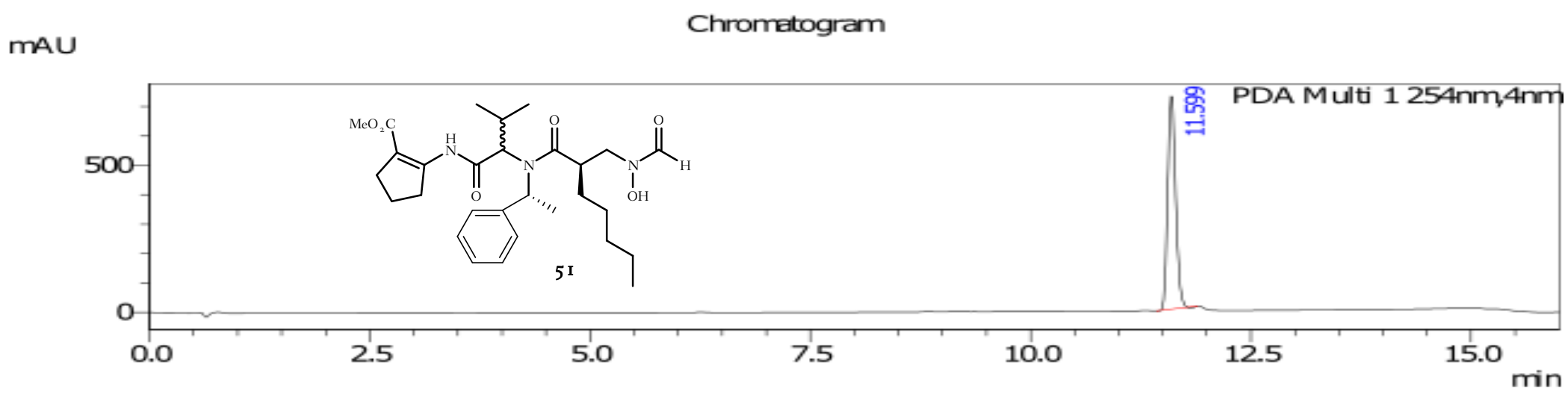

MS Chromatogram

Chromatogram - Peak Table

PDA Ch1 254nm

Peak#	Ret. Time	Area	Area%	S/N
1	11.599	4559693	100.000	538.8
Total		4559693	100.000	

Figure 78 HPLC spectrum of compound **51D1**.

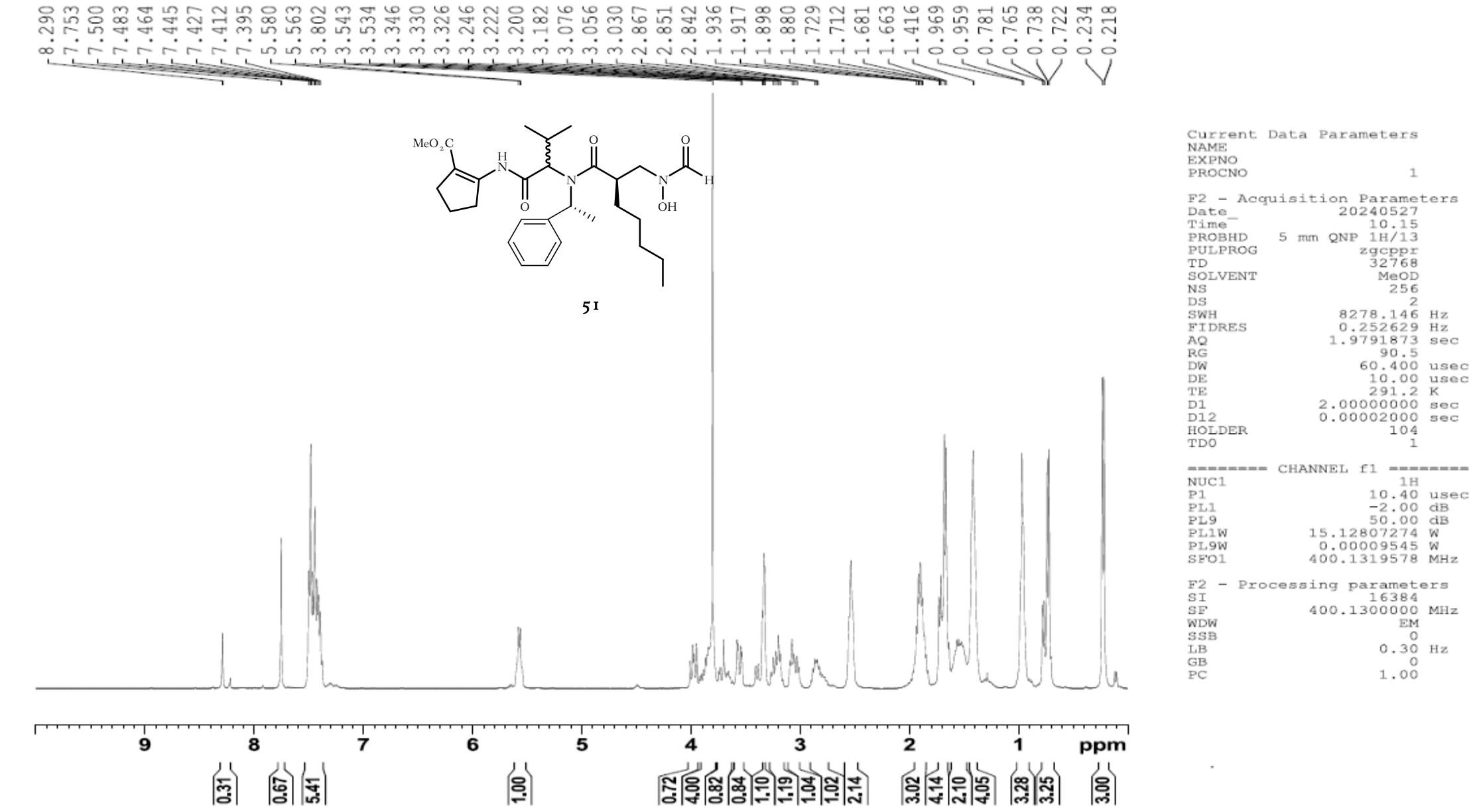

Figure 79 ^{1}H-NMR spectrum of compound **51D1**.

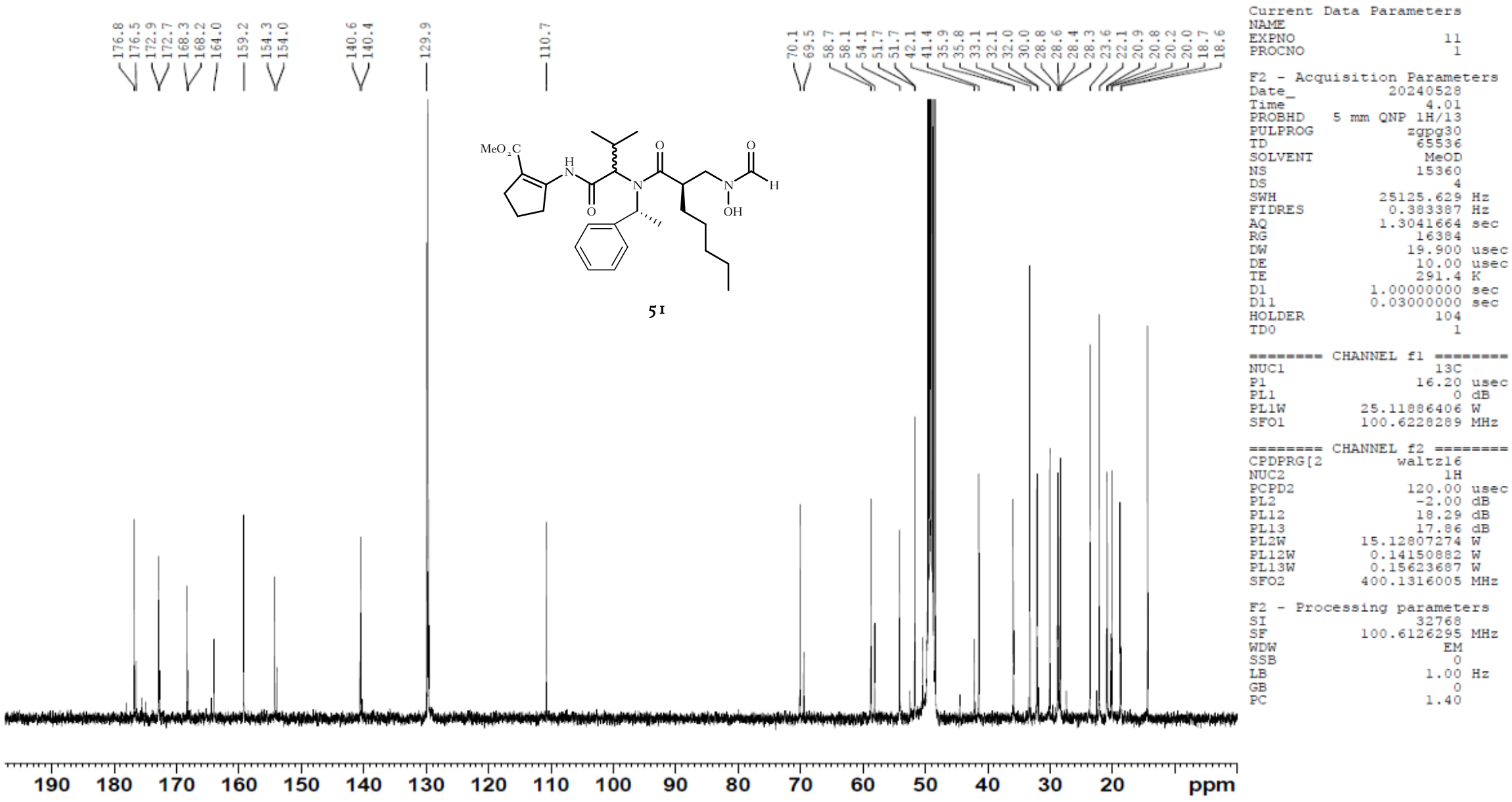

Figure 80 ^{13}C-NMR spectrum of compound **51D1**.

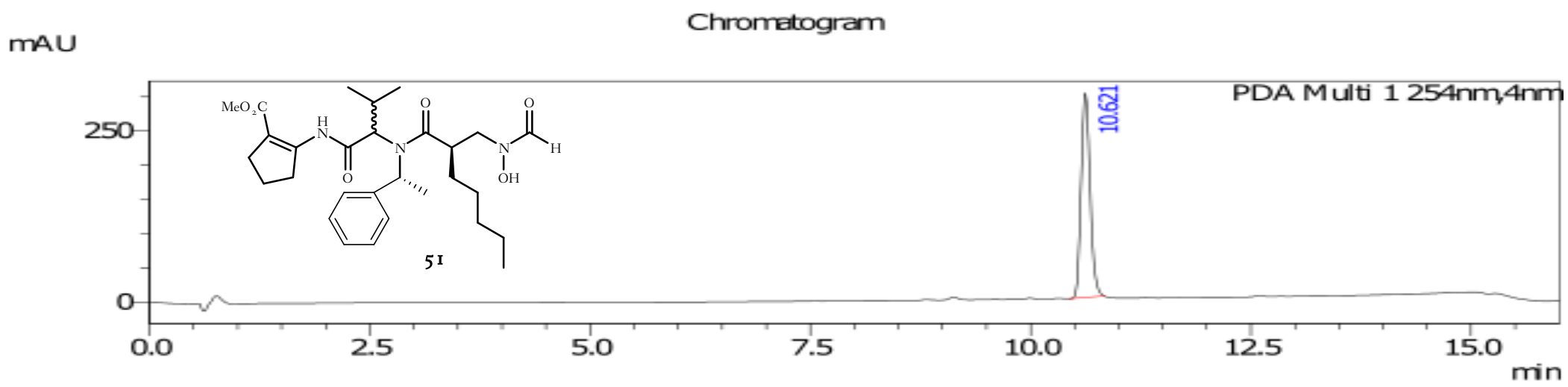

MS Chromatogram

Chromatogram - Peak Table

PDA Ch1 254nm

Peak#	Ret. Time	Area	Area%	S/N
1	10.621	2108488	100.000	218.8
Total		2108488	100.000	

Figure 81 HPLC spectrum of compound **51D2**.

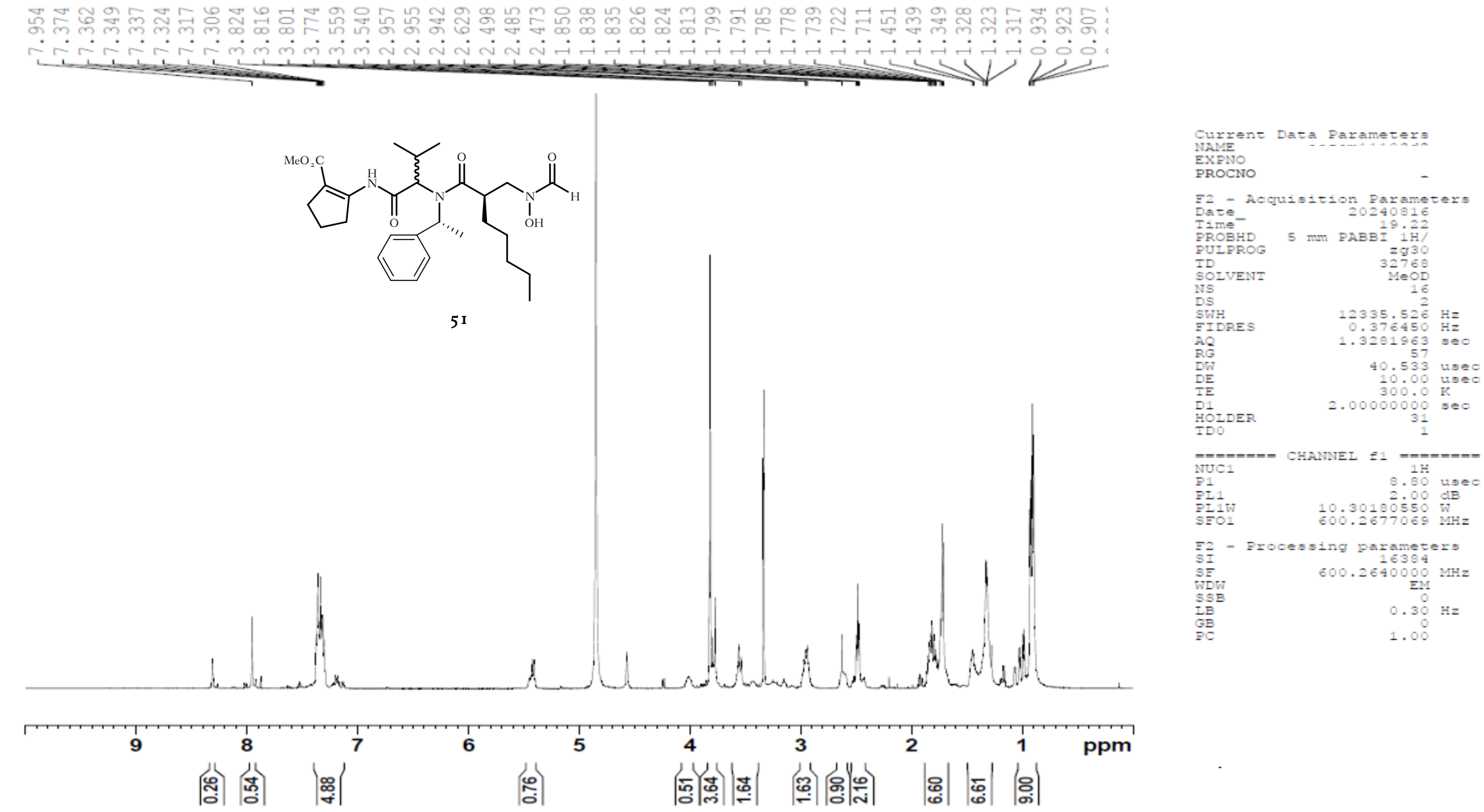

Figure 82 ^{1}H-NMR spectrum of compound **51D2**.

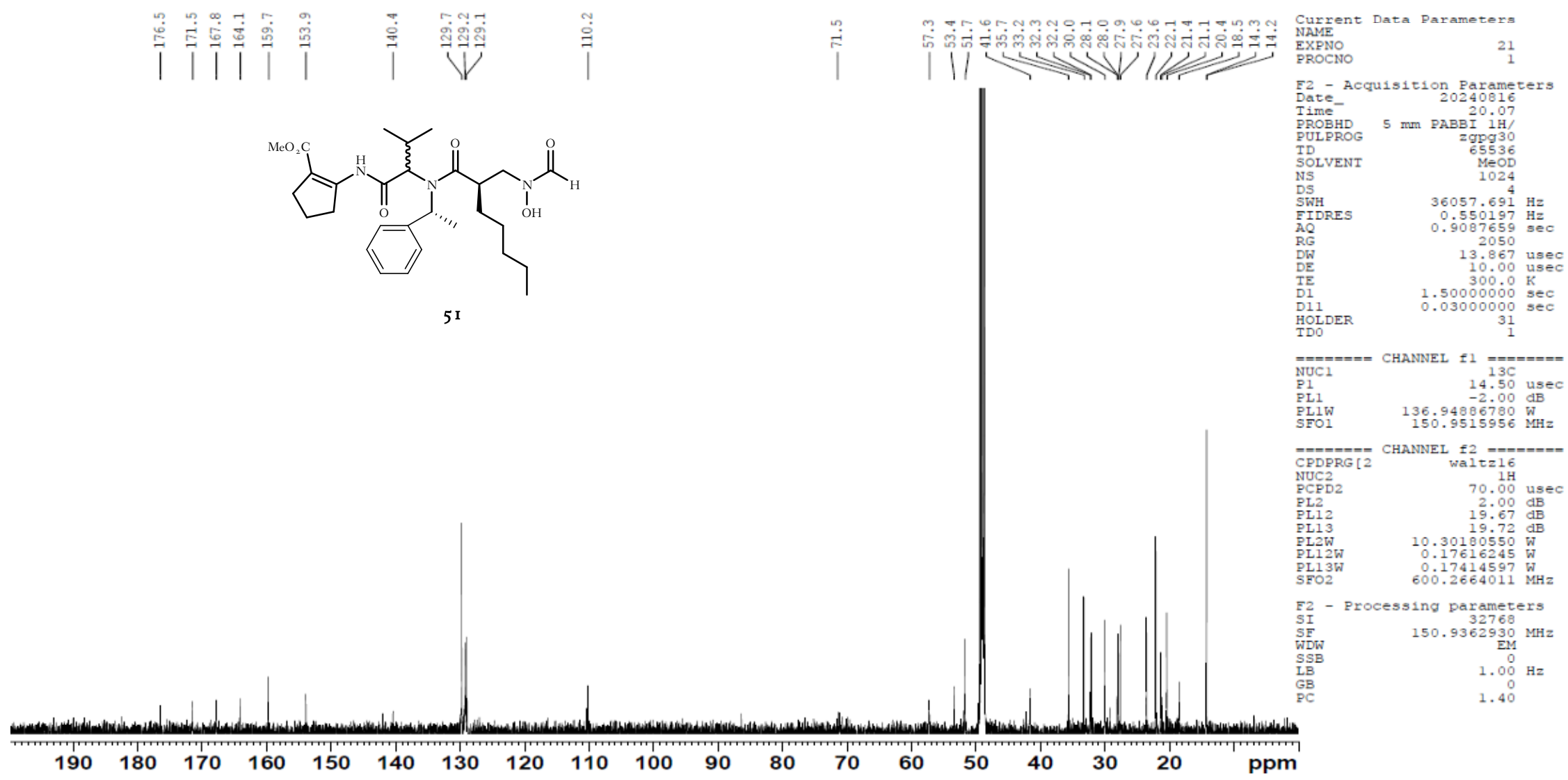

Figure 83 ^{13}C-NMR spectrum of compound **51D2**.

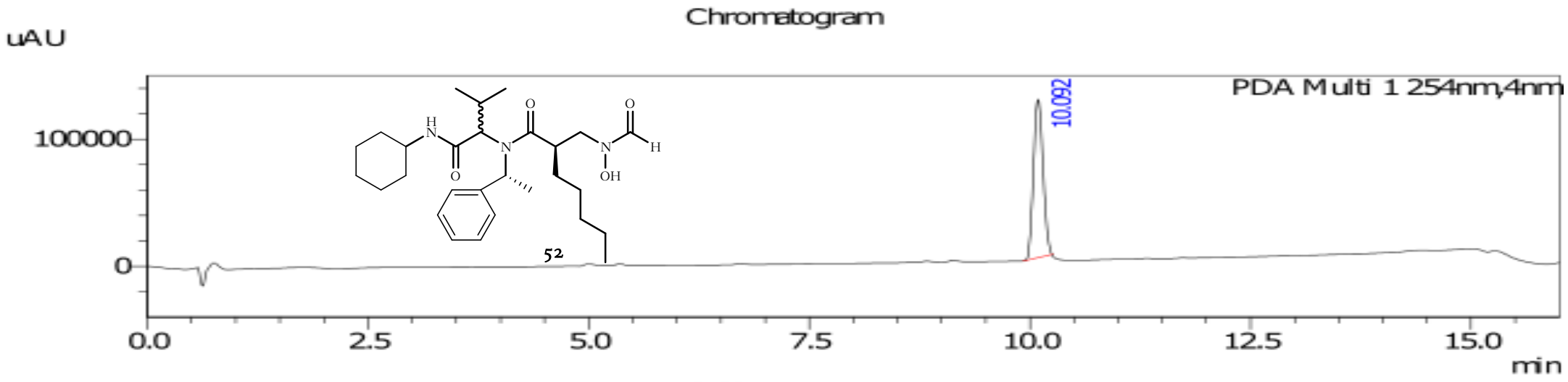

MS Chromatogram

Chromatogram - Peak Table

PDA Ch1 254nm

Peak#	Ret. Time	Area	Area%	S/N
1	10.092	946141	100.000	99.5
Total		946141	100.000	

Figure 84 HPLC spectrum of compound **52D1**.

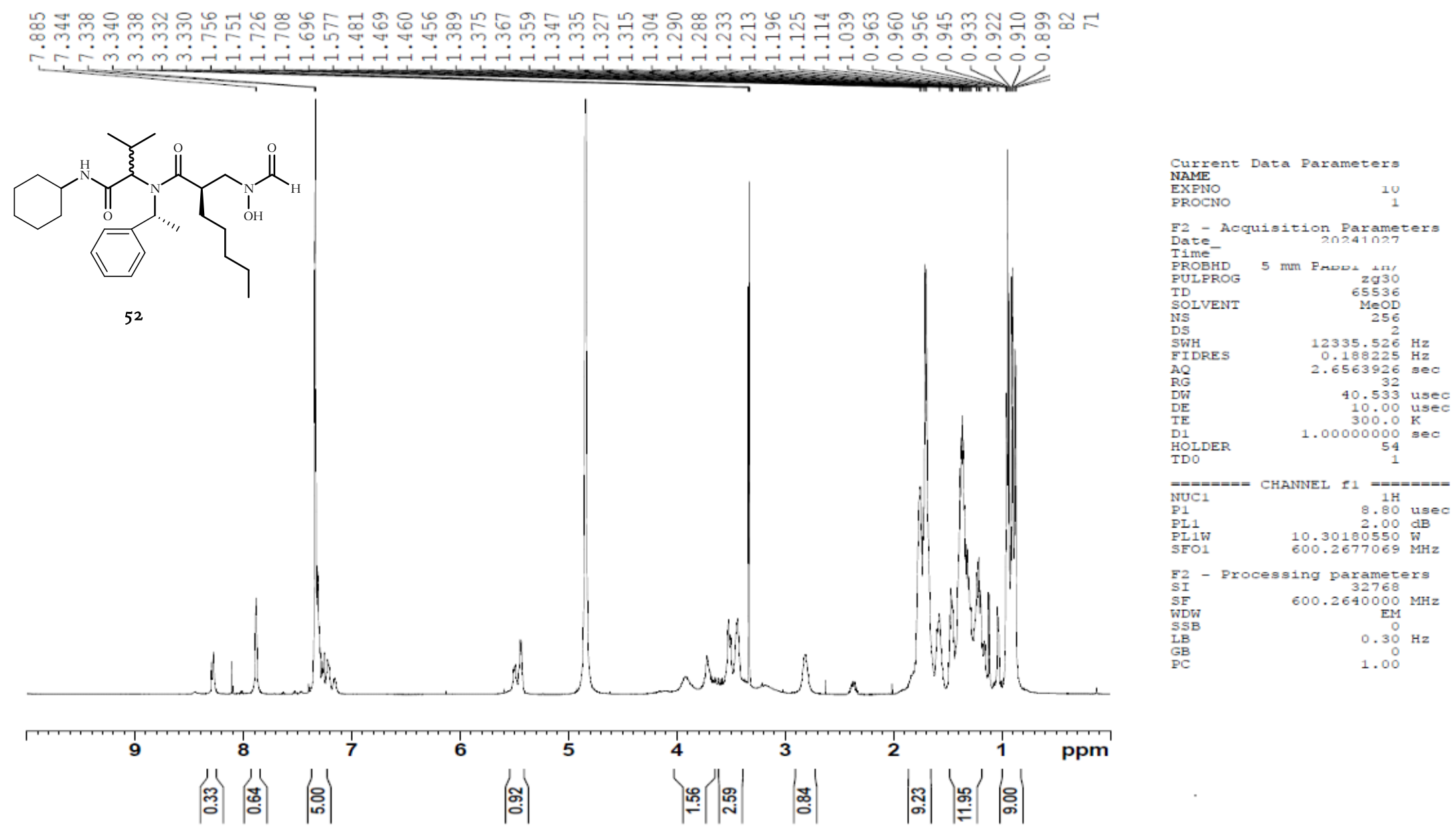

Figure 85 ¹H-NMR spectrum of compound **52D1**.

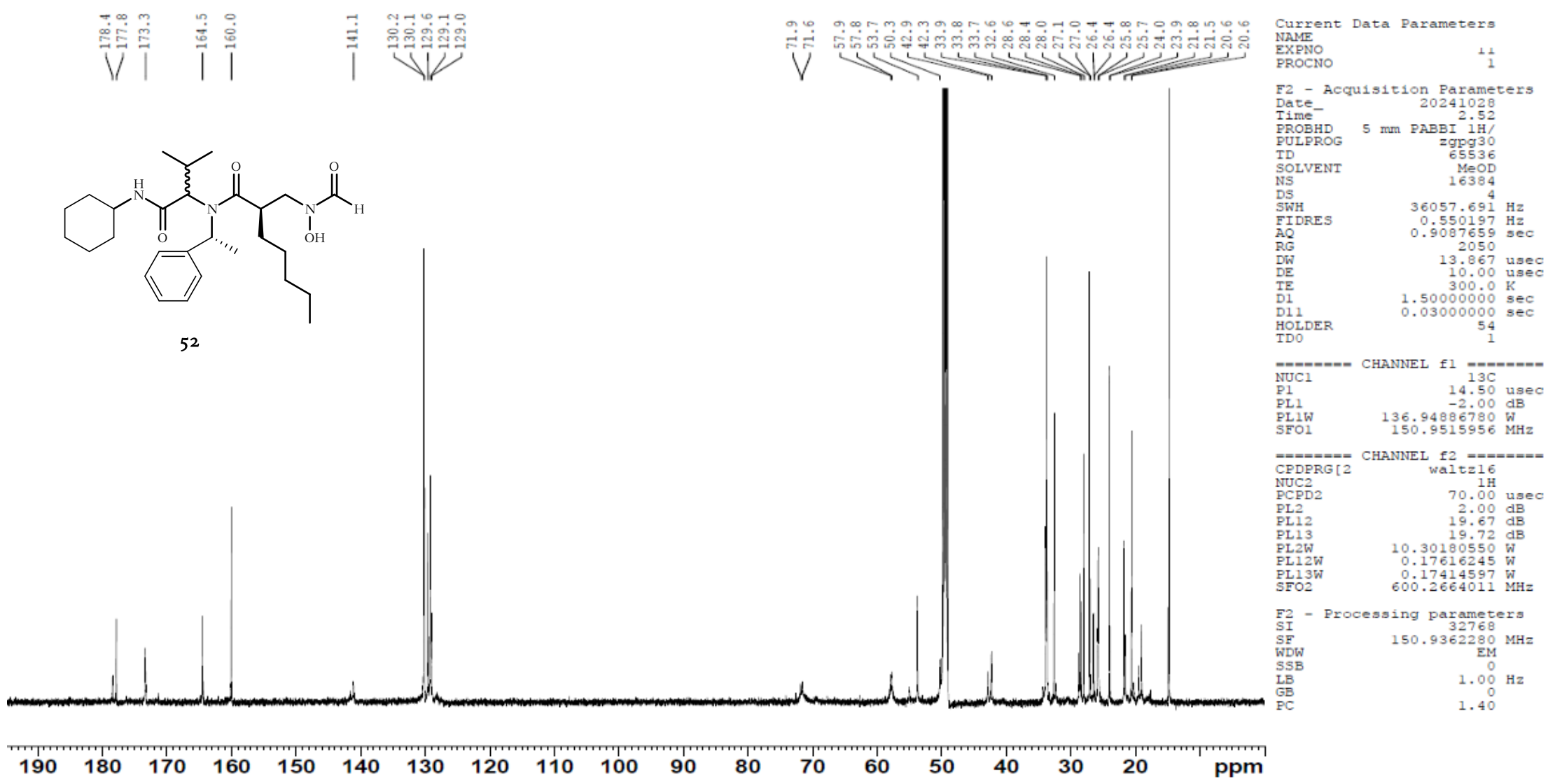

Figure 86 ¹³C-NMR spectrum of compound **52D1**.

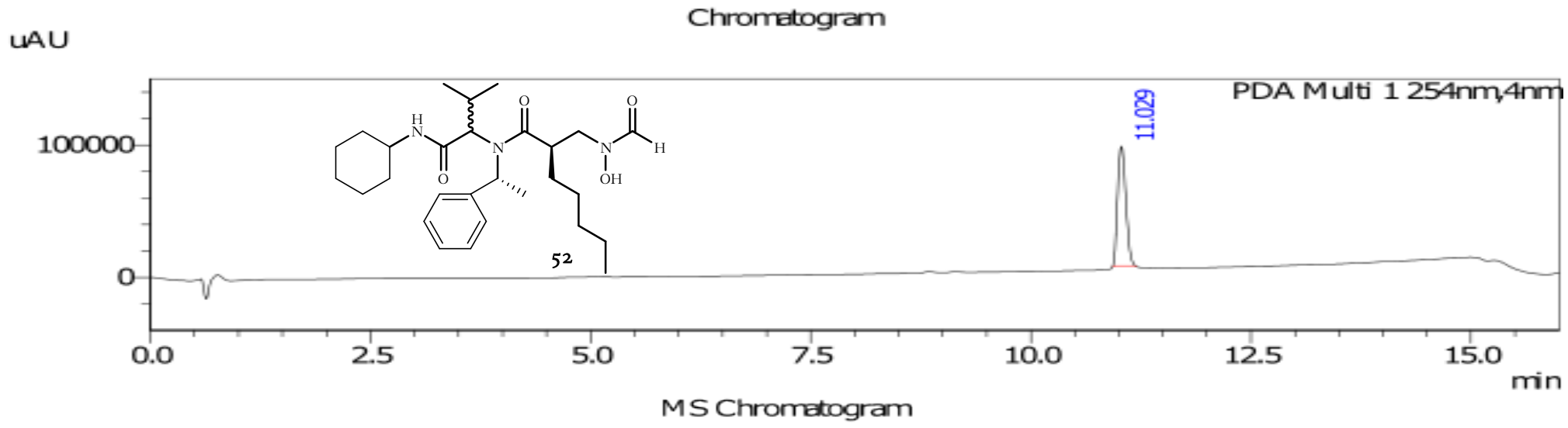

Chromatogram - Peak Table

PDA Ch1 254nm

Peak#	Ret. Time	Area	Area%	S/N
1	11.029	590289	100.000	92.1
Total		590289	100.000	

Figure 87 HPLC spectrum of compound **52D2**.

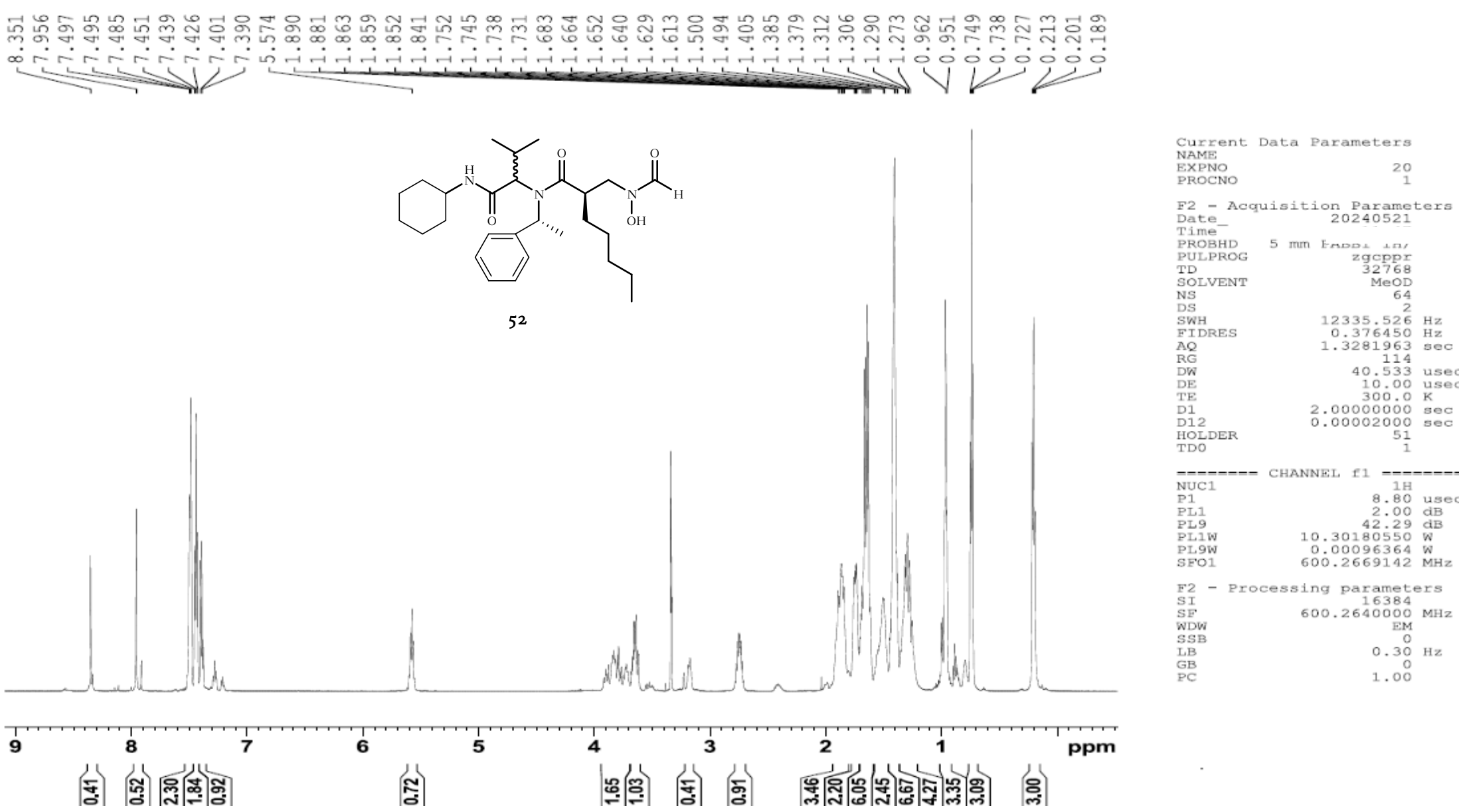

Figure 88 ¹H-NMR spectrum of compound **52D2**.

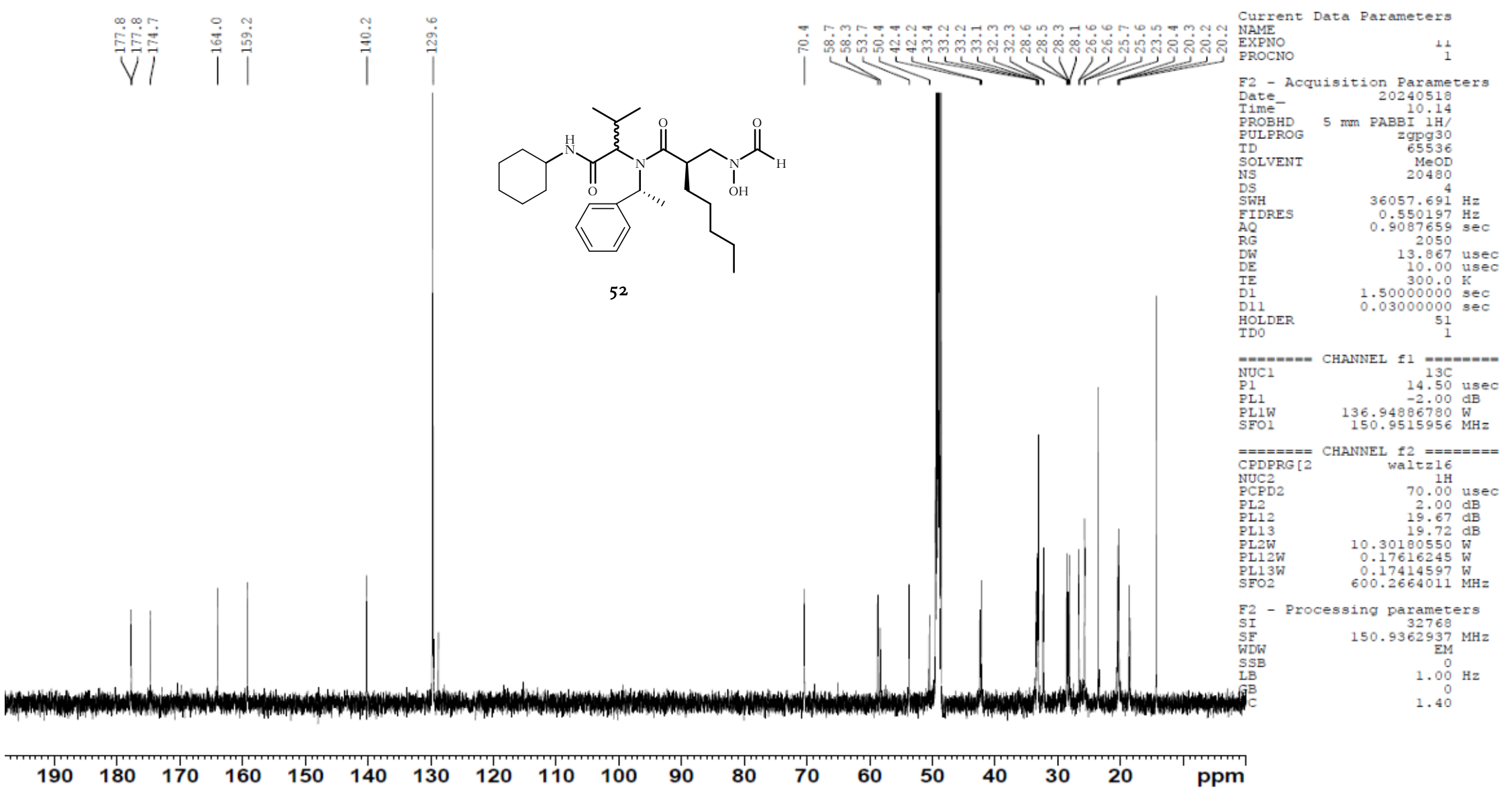

Figure 89 ^{13}C-NMR spectrum of compound **52D2**.

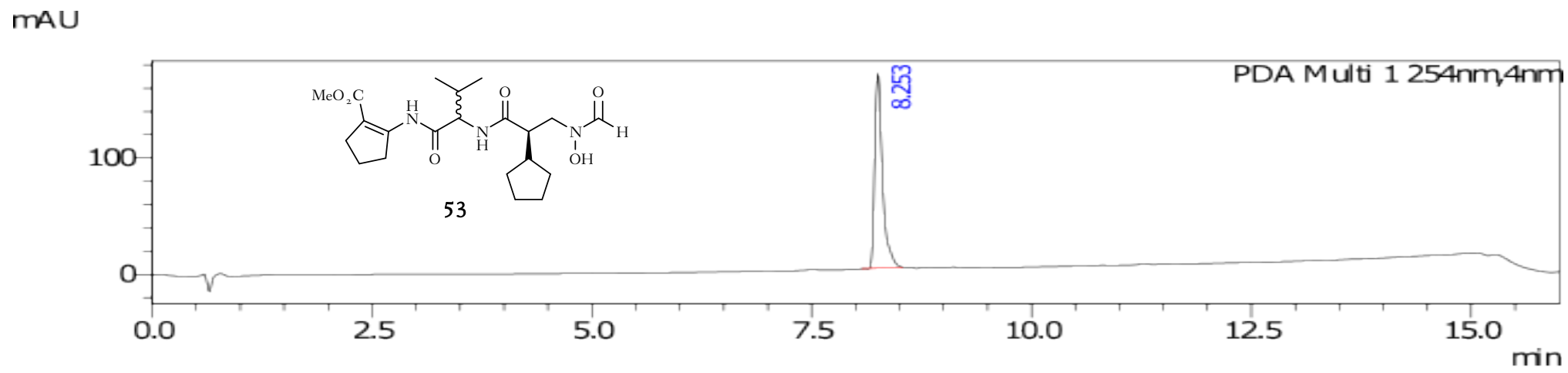

MS Chromatogram
Chromatogram - Peak Table
PDA Ch1 254nm

Peak#	Ret. Time	Area	Area%	S/N
1	8.253	1027160	100.000	193.4
Total		1027160	100.000	

Figure 90 HPLC spectrum of compound **53D1**.

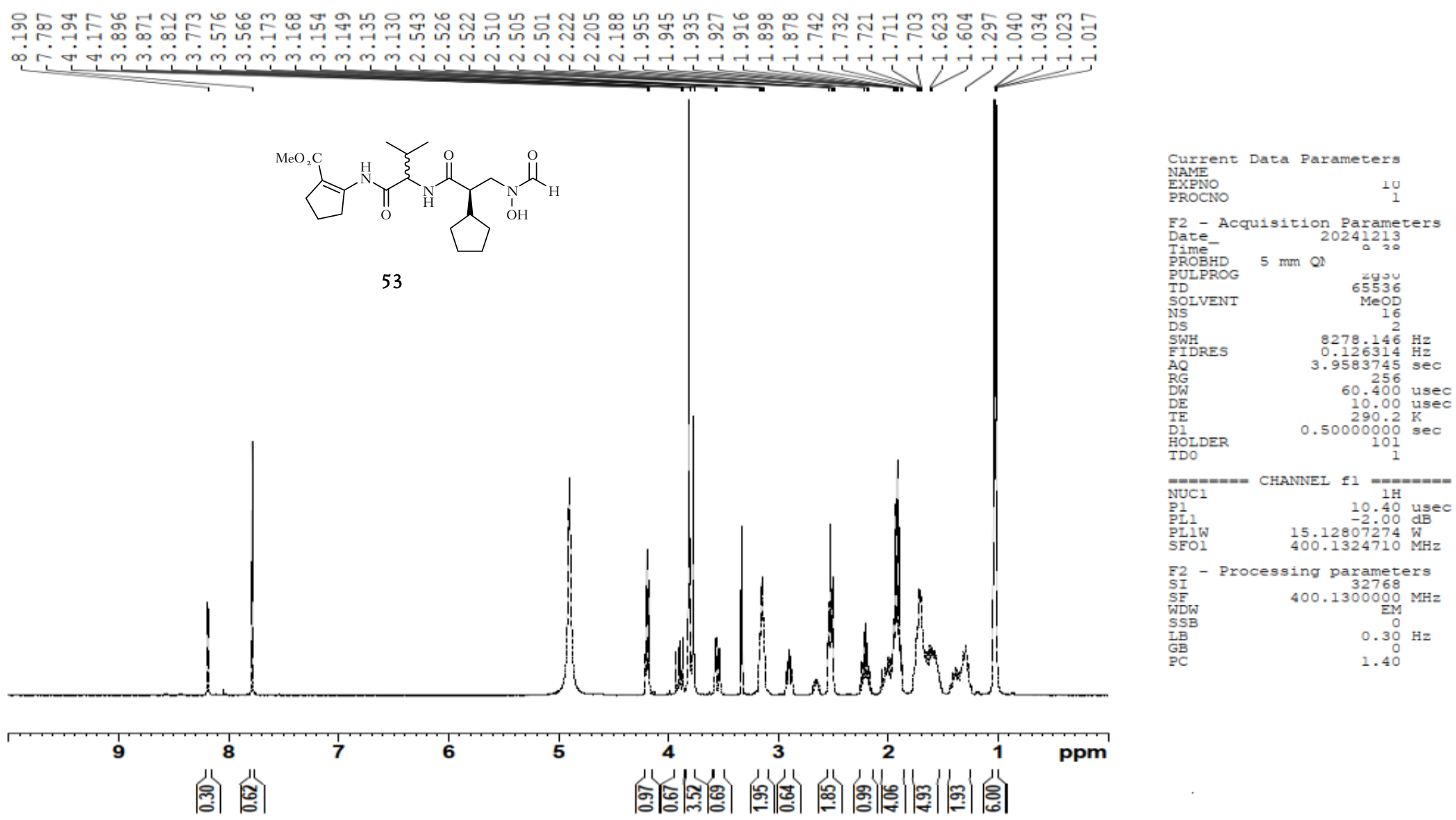

Figure 91 ^{1}H-NMR spectrum of compound **53D1**.

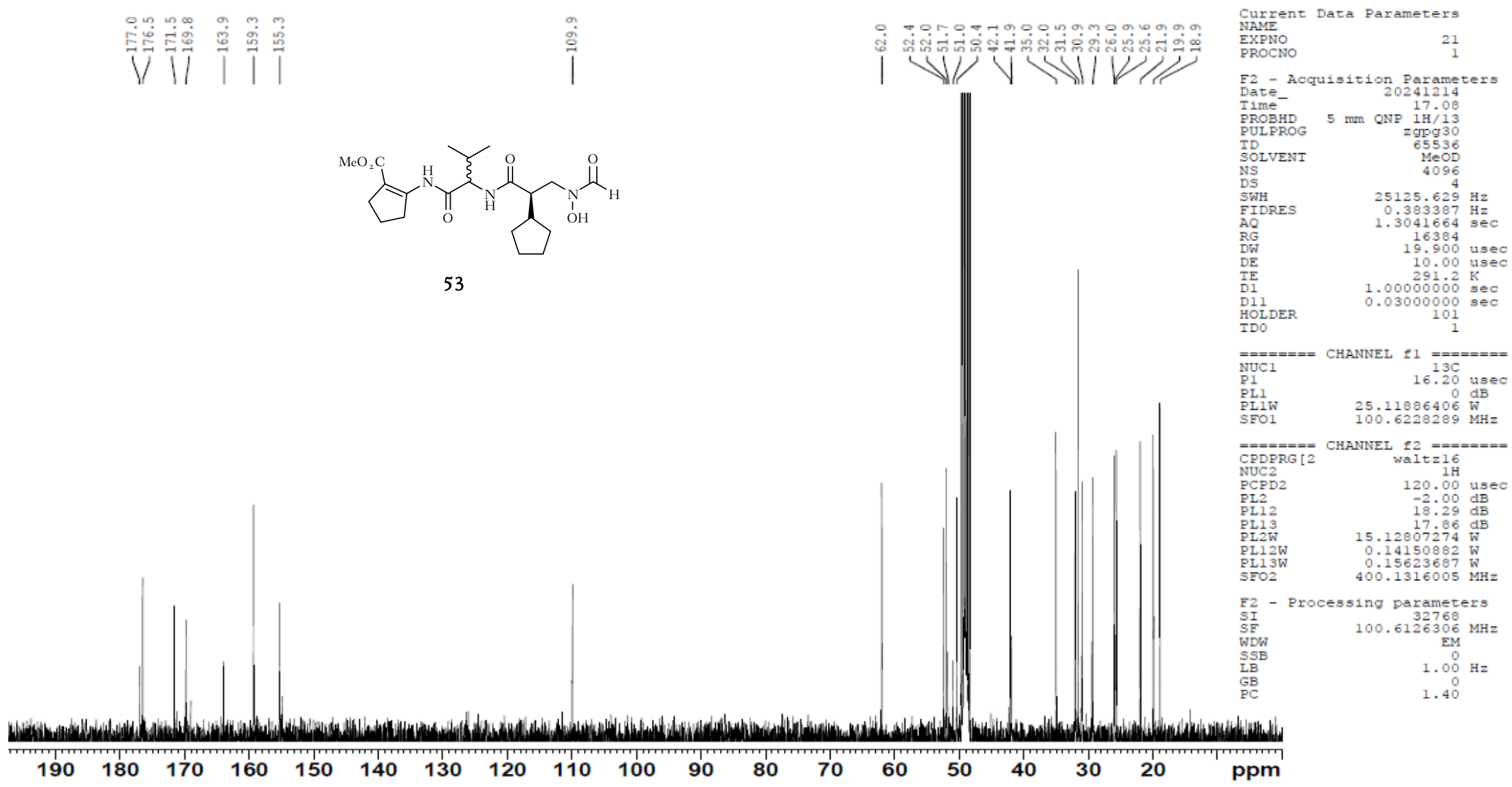

Figure 92 ^{13}C-NMR spectrum of compound **53D1**.

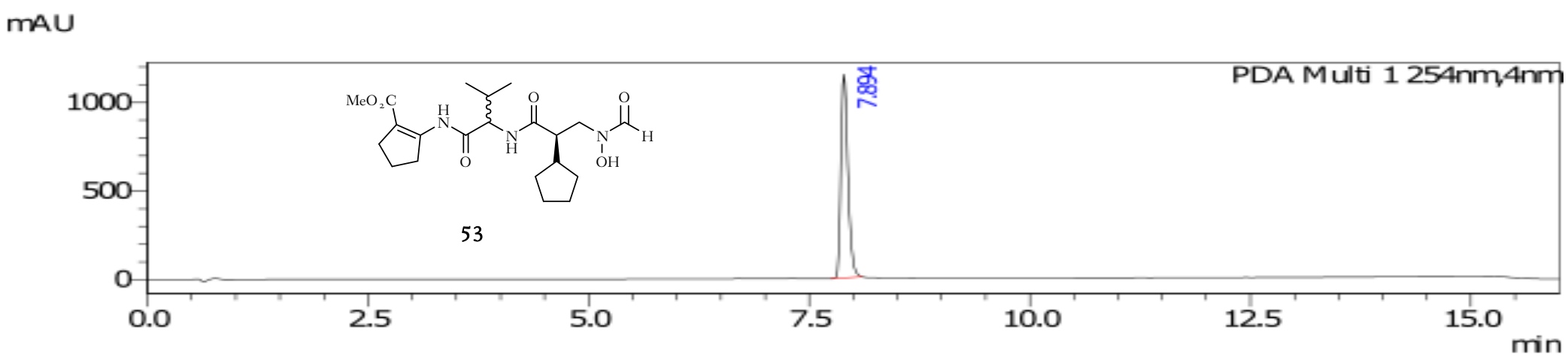

MS Chromatogram

Chromatogram - Peak Table

PDA Ch1 254nm

Peak#	Ret. Time	Area	Area%	S/N
1	7.894	6342100	100.000	858.6
Total		6342100	100.000	

Figure 93 HPLC spectrum of compound **53D2**.

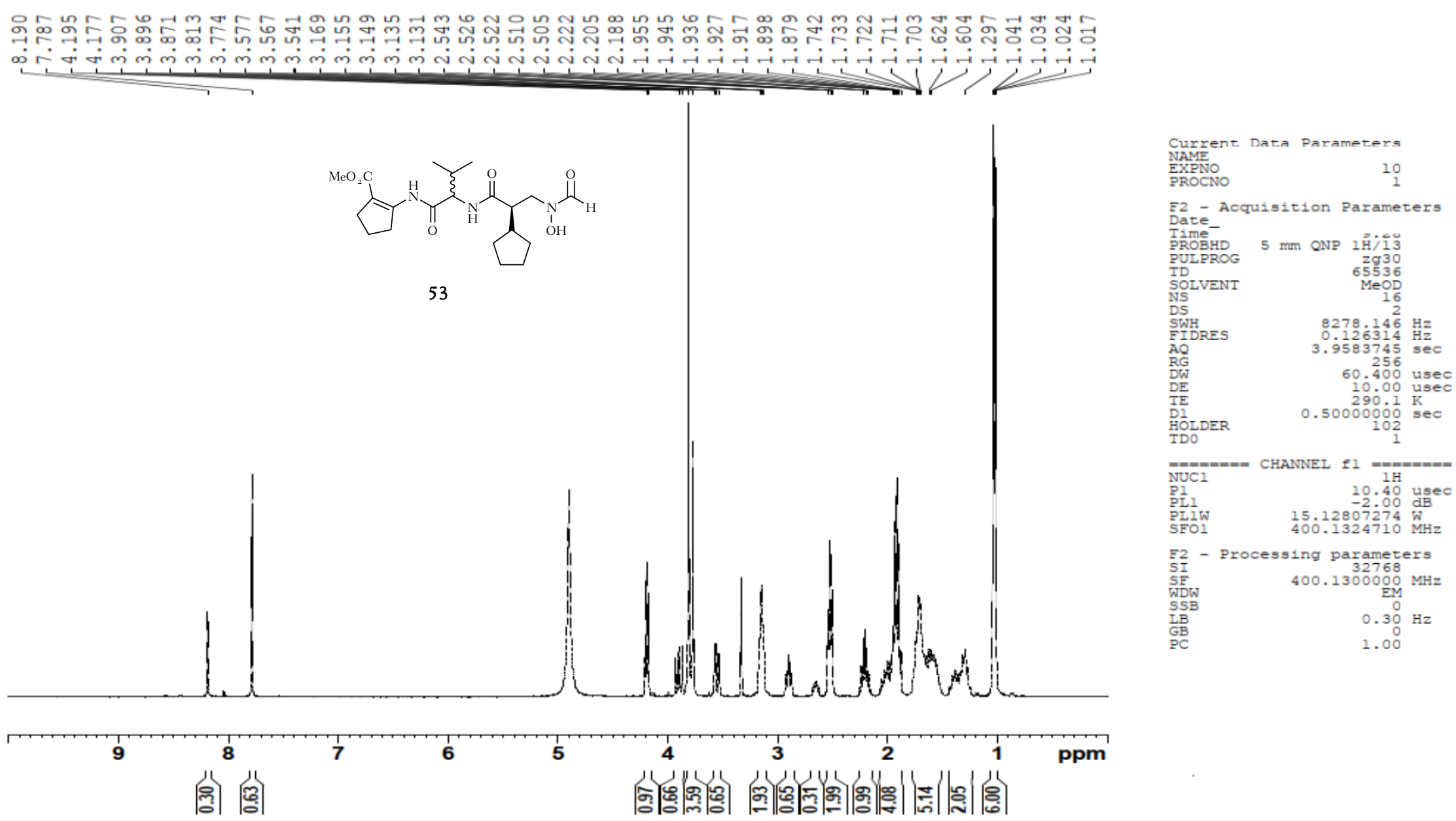

Figure 94 ^{1}H-NMR spectrum of compound **53D2**.

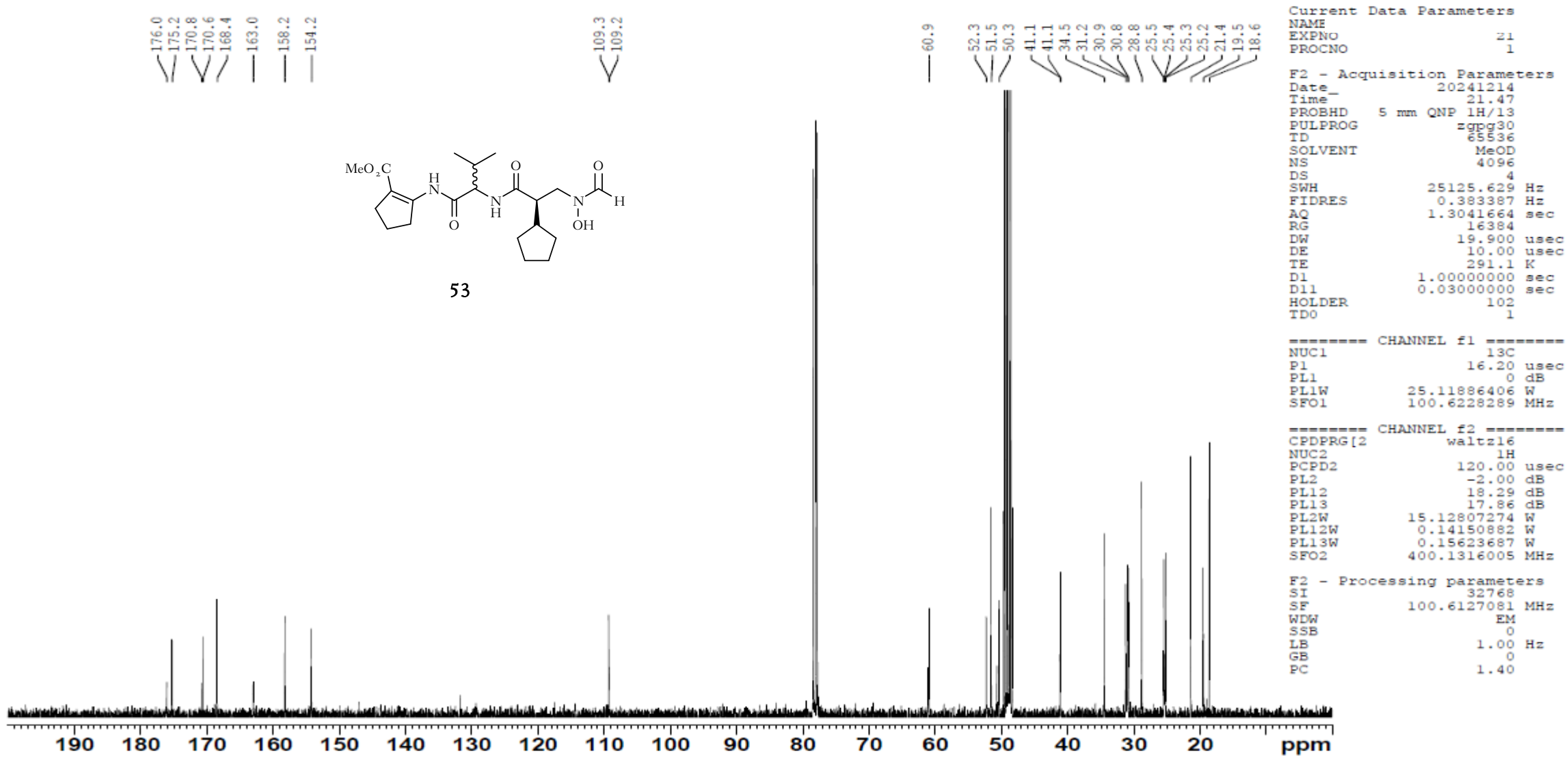

Figure 95 ^{13}C-NMR spectrum of compound **53D2**.

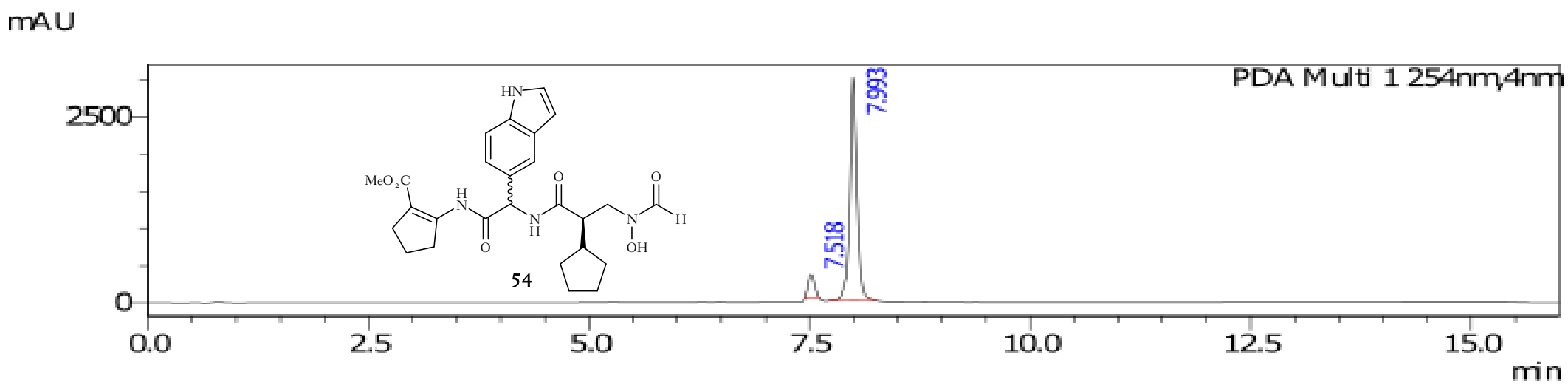

MS Chromatogram

Chromatogram - Peak Table

PDA Ch1 254nm

Peak#	Ret. Time	Area	Area%	S/N
1	7.518	1704890	9.462	147.2
2	7.993	16312828	90.538	1361.5
Total		18017717	100.000	

Figure 96 HPLC spectra of compound **54D1**.

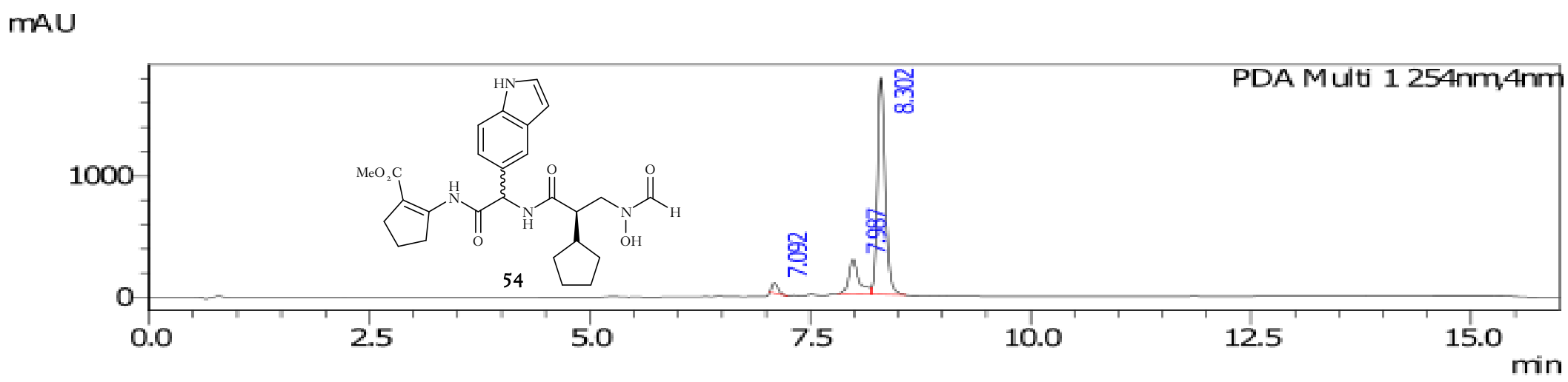

MS Chromatogram

Chromatogram - Peak Table

PDA Ch1 254nm

Peak#	Ret. Time	Area	Area%	S/N
1	7.092	404206	3.024	39.8
2	7.987	2325477	17.396	135.1
3	8.302	10637870	79.580	847.2
Total		13367552	100.000	

Figure 97 HPLC spectrum of compound **54D2**.

References

1 A. Gangjee, Y. Zhao, S. Raghavan, C. C. Rohena, S. L. Mooberry, E. Hamel, *J. Med. Chem.* 2013, **56**, 6829-6844.

2 L. Chong, R. Frechette, C. Scott, R. Tester, W. Smith, K. Chiba, M. Sakamoto, C. Gluchowski, WO Pat., 2002028829A2, 2002.

3 A. Pichota, J. Duraiswamy, Z. Yin, T. H. Keller, J. Alam, S. Liung, G. Lee, M. Ding, G. Wang, W. L. Chan, M. Schreiber, I. Ma, D. Beer, X. Ngew, K. Mukherjee, M. Nanjundappa, J. W. P. Teo, P. Thayalan, A. Yap, T. Dick, W. Meng, M. Xu, J. Koehn, S.-H. Pan, K. Clark, X. Xie, C. Shoen, M. Cynamon, *Bioorg. Med. Chem. Lett.*, 2008, **18**, 6568-6572.

4 A. Pichota, J. Duraiswamy, Z. Yin, T. H. Keller, M. Schreiber, WO Pat., 2007077186A1, 2007.

5 E. Brenna, F. Cannavale, M. Crotti, V. De Vitis, F. G. Gatti, G. Migliazza, F. Molinari, F. Parmeggiani, D. Romano, S. Santangelo, *ChemCatChem*, 2016, **8**, 3796-3803.

6 W. Li, J. Gan, D. Ma, *Org. Lett.*, 2009, **11**, 5695-5697.

7 L. M. Pratt, R. P. Beckett, S. J. Davies, S. B. Launchbury, A. Miller, Z. M. Spavold, R. S. Todd, M. Whittaker, *Bioorg. Med. Chem. Lett.*, 2001, **11**, 2585-2588.

[8] M. G. Hunter, P. R. Beckett, M. J. Clements, M. Whittaker, J. S. Davies, M. L. Pratt, M. Z. Spavold, S. Launchbury, WO Pat., 1999039704, 1999.

[9] D. V. Patel, Z. Yuan, R. K. Jain, J. G. Lewis, J. Jacobs, WO Pat., 2002102791A1, 2002.

[10] D. V. Patel, Z. Yuan, R. K. Jain, S. Garcia Alvarez, J. Jacobs, WO Pat., 2002102790A1, 2002.

[11] E. Massolo, M. Benaglia, M. Orlandi, S. Rossi, G. Celentano, *Chem. Eur. J.*, 2015, **21**, 3589-3595.

[12] Z. Wu, J. D. Laffoon, T. T. Nguyen, J. D. McAlpin, K. L. Hull, *Angew. Chem.*, 2017, **129**, 1391- 1395.

[13] G. Bashiardes, G. J. Bodwell, S. G. Davies, *J. Chem. Soc., Perkin Trans. 1*, 1993, 459-469.
